# Desulfurizative Fluorination of *N*‐CF_3_ Thioformamides for the Efficient Synthesis of *N*(CF_3_)(CF_2_H) Amines with Enhanced Stability

**DOI:** 10.1002/anie.202506154

**Published:** 2025-05-02

**Authors:** Gina Wycich, Jaime Ponce‐de‐León, Linhao Liu, Franziska Schoenebeck

**Affiliations:** ^1^ Institute of Organic Chemistry RWTH Aachen University Landoltweg 1 52074 Aachen Germany

**Keywords:** Desulfurization, Fluorination, Fluorine, *N*‐Difluoromethyl amines, Robustness

## Abstract

With poor metabolic stability being a major cause of failure in drug development, there is a pressing need for strategic molecular modifications to optimize for desired properties and function. *N*‐substitution has emerged as a powerful approach, with *N*‐CF_3_ amines previously demonstrating enhanced lipophilicity and reduced susceptibility to oxidation, albeit inherent instability to hydrolysis. This report discloses the further evolution of this motif—the introduction of an additional *N*‐difluoromethyl unit, resulting in an extraordinary 2000‐fold increase in stability. We present the first general synthetic strategy for accessing *N*(CF_3_)(CF_2_H) amines. The method relies on an operationally simple desulfurization–fluorination strategy of *N*‐CF_3_ thioformamides and is characterized by broad functional group tolerance.

## Graphical abstract

We present the first general synthetic strategy for accessing *N*(CF_3_)(CF_2_H) amines, which display a 2000‐fold increase in stability over *N*‐CF_3_ amines. The compounds are synthesized through a desulfurization–fluorination of *N*‐CF_3_ thioformamides.

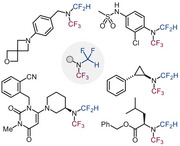

Approximately 84% of all approved drugs contain at least one nitrogen atom,^[^ ^1^, ^2^ ^]^ often in the form of amines, which are fundamental to drug development due to their critical roles in drug–receptor interactions and pharmacokinetics derived from their basicity and hydrogen bonding capacity. However, these properties also make amines vulnerable, and poor metabolic stability accounts for approximately 30%–40% of failures in drug development, with *N*‐oxidation and *N*‐dealkylation in nitrogen‐containing drugs being major contributors.^[^ ^3^ ^]^ There is hence a significant interest to modify the *N*‐substitution to enhance metabolic stability and tailor it toward desired properties and function. Owing to fluorine's well‐established stability‐enhancing and property‐modulating effects,^[^ ^4^, ^5^, ^6^ ^]^ the *N*‐trifluoromethylation of amines^[^ ^7^, ^8^, ^9^ ^]^ (as well as that of other *N*‐containing functional groups)^[^ ^10^, ^11^, ^12^, ^13^, ^14^, ^15^, ^16^, ^17^, ^18^, ^19^ ^]^ has drawn significant interest in recent years as a potential strategy to decrease the propensity for oxidation or dealkylation at nitrogen while also enhancing cell permeability (i.e., lipophilicity).^[^ ^20^ ^]^ Beyond stability, the ability to fundamentally change the properties of nitrogen, paired with the large number of existing amine‐containing drugs, could fuel the discovery of a variety of new drug candidates (or agrochemicals, materials, and more).^[^ ^21^, ^22^, ^23^, ^24^, ^25^ ^]^ That said, while *N*‐CF_3_ amines were indeed found to have enhanced lipophilicity and stability toward oxidation, they displayed insufficient robustness to hydrolysis under metabolically relevant conditions.^[^ ^26^ ^]^ To combat these challenges and enable new functions, it is therefore imperative to identify novel and improved motifs as well as provide efficient synthetic methodology for their straightforward access (Figure 1a).

**Figure 1:**
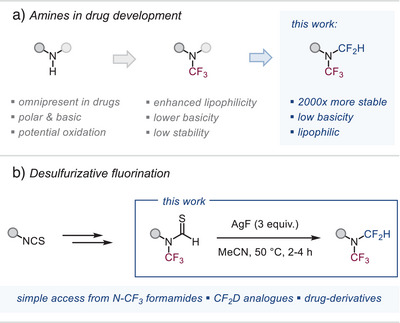
Importance of amines and their further modification.

To this end, we considered the introduction of an additional difluoromethyl group as a potentially advantageous modification. The R*N*(CF_3_)(CF_2_H) motif should similarly be protected toward oxidation or dealkylation as *N*‐CF_3_ amines, and the additional electron‐deficient difluoromethyl group might render the unit overall more robust.^[^ ^27^ ^]^ The difluoromethyl also uniquely presents an H‐bonding donor, which neither *N‐*CF_3_ amines nor their close relatives possess,^[^ ^28^, ^29^ ^]^ but is critical for receptor recognition and drug efficacy. Moreover, our calculation of the log*P* value,^[^ ^30^, ^31^ ^]^ which is a measure of lipophilicity,^[^ ^32^ ^]^ of Ph*N*(CF_3_)(CF_2_H) versus Ph*N*(CF_3_)(Me) indicated very similar values for both, deviating only by 0.03 (see Figure 2c). These results suggest that R*N*(CF_3_)(CF_2_H) could indeed be a superior structural unit to R_2_ *N*(CF_3_), possessing analogous lipophilicity, lack of oxidation or dealkylation while offering an H‐bonding unit and potentially greater robustness. However, there is a lack of general, safe and functional‐group‐tolerant methodology to access the R*N*(CF_3_)(CF_2_H) motif, which is likely also a contributing factor to why it has not been studied for its enabling potential to date. Indeed, only a single example of this moiety has ever been made (in low 20% yield) by reaction of methyl *N*‐aryl‐*N*‐formyldithiocarbamate with highly toxic gaseous SF_4_.^[^ ^33^ ^]^

**Figure 2:**
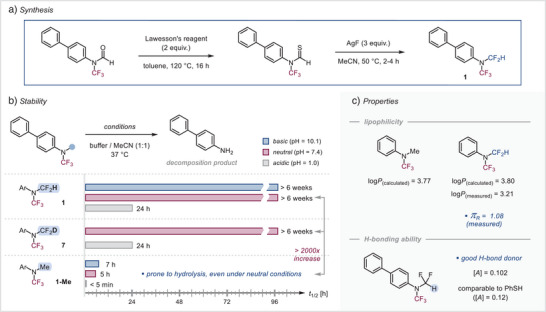
Evaluation of stability and properties of *N*(CF_3_)(CF_2_H) amines and analogues.

In light of the power of silver fluoride to enable effective desulfurizative fluorination of C═S bonds,^[^ ^7^, ^34^, ^35^, ^36^, ^37^ ^]^ we envisioned that a promising precursor to unlock broad access to R*N*(CF_3_)(CF_2_H) could be an *N*‐CF_3_ thioformamide, i.e., R*N*(CF_3_)─C═S(H) (see Figure 1b). While such a motif has never been made (nor has desulfurizative fluorination been demonstrated for it), we recently developed a straightforward synthesis of structurally diverse *N*‐CF_3_ formamides.^[^ ^18^ ^]^ We envisioned that the formamides could in a single step be transformed to their thioformamide counterparts with, e.g., Lawesson's reagent. Indeed, for the biphenyl *N*‐CF_3_ formamide, this approach proved viable (Figure 2a). To our delight, the subsequent exposure of the corresponding thioformamide with AgF (2 equiv) at 50 °C in MeCN for 2 h led to the formation of *N*(CF_3_)(CF_2_H) amine **1** in 66% yield.^[^ ^38^ ^]^ Increasing the amount of AgF to 3 equiv and the concentration from 0.01 to 0.2 M led to the quantitative formation of **1**, which was isolated as a colorless, bench‐stable solid in 98% yield. The use of DCM as solvent instead of MeCN did not affect the yield. Since the only by‐products of this transformation are insoluble salts (Ag_2_S and unreacted AgF), the product can be easily obtained by simple filtration over silica and solvent evaporation. With access to an *N*(CF_3_)(CF_2_H) amine unlocked, the stability of the compound was investigated next. For the corresponding R_2_ *N*‐CF_3_ amines, Cox et al. observed their rapid decomposition in aqueous media at room temperature.^[^ ^26^ ^]^ Moreover, the half‐life of some drug‐derived *N*‐CF_3_ amines was found to be less than one day regardless of the pH of the solution. Therefore, we set out to determine the stability of *N*(CF_3_)(CF_2_H) amines in different aqueous media under analogous conditions.

To this end, we subjected compound **1** to a 1:1 MeCN/buffer solution under a range of different pH media (Figure 2b). At pH 7.4 and more basic pH 10, *N*(CF_3_)(CF_2_H) amine **1** proved to be fully stable for more than 6 weeks (i.e., the time we tested) at room temperature or at 37 °C. For comparison, the *N*‐CF_3_ amine **1‐Me** that bears a methyl group in place of the CF_2_H and is otherwise structurally identical, showed 87% decomposition within just 24 h at 37 °C and neutral pH in the analogous buffer solution. As such, the additional CF_2_H has a tremendous effect on the overall stability.

Even under strongly acidic conditions (pH 1) at 37 °C, 49% of the compound remained after 24 h (and needed one week for full consumption), whereas the corresponding *N*(CF_3_)(Me) amine **1‐Me** was nearly fully decomposed under these conditions in 5 min.

We also prepared the corresponding deuterated analogue of **1**, i.e., (biphenyl)*N*(CF_3_)(CF_2_D) (**7**), which proved to be as stable as the nondeuterated counterpart (see Figure 2b).

Our experimental determination of the Hansch–Leo parameter of Ph*N*(CF_3_)(CF_2_H) (**2**) gave a value of π_R_ = 1.08, slightly higher than that of the OCF_3_ group (π_R_ = 1.04).^[^ ^39^ ^]^

In light of the promising stabilities, the H‐bonding ability of the new motif was evaluated next. To this end, we employed Abraham's method,^[^ ^40^ ^]^ which quantifies the H‐bonding ability through the proton chemical shift deviation observed when measured in CDCl_3_ versus DMSO‐*d*_6_ and subsequent calculation of the corresponding [*A*] value.^[^ ^41^ ^]^ Following this approach, an [*A*] = 0.10 was determined for the newly synthesized *N*(CF_3_)(CF_2_H) amine **1**, which suggests a pronounced H‐bonding capability, comparable to that of thiophenol ([*A*] = 0.12) and stronger than that of aniline ([*A*] = 0.07).^[^ ^42^ ^]^ As such, the *N*(CF_3_)(CF_2_H) amines should function as similarly good H‐bonding motifs as thiols or amines, which is highly promising for molecular recognition, including drug–receptor interactions.

With these highly promising properties identified, we next explored the generality of our synthetic approach and whether highly functional examples could be synthesized in this manner (Scheme 1a). We observed that both aromatic and aliphatic *N*‐CF_3_ thioformamides reacted smoothly with AgF to the desired *N*(CF_3_)(CF_2_H) amines in excellent yields. Neither the presence of electron‐donating (aryl, alkyl, or methoxy, **1**–**4**) nor electron‐withdrawing groups (ester, halogens, **5**, **6**) at the aromatic ring influenced the reaction outcome. Moreover, nitrogen moieties were well tolerated, which are of importance in a medicinal context. *N*–Boc protected piperidine product (**8**) was obtained in quantitative yield, and similar results were gathered for the synthesis of novel *N*(CF_3_)(CF_2_H) amino acid‐based building blocks derived from phenylalanine and leucine (**9**, **10**). The latter was isolated on a gram scale with all stereochemical integrity retained. Functionalization in the benzylic position was also feasible (**11**). Heterocycle cores with high relevance in drugs or agrochemicals, such as carbazole and thiophene, were also subjected to our conditions and gave the desired product in good yields (**13**, **14**).

**Scheme 1:**
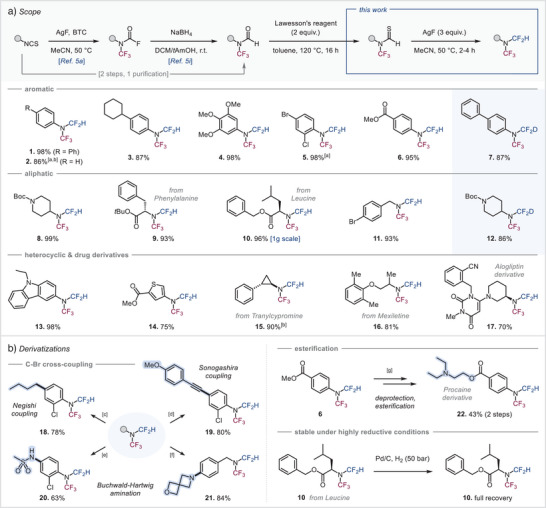
Scope of *N‐*CF_3_ thioformamide desulfurization–fluorination. Reaction conditions: *N*‐CF_3_ thioformamide (1 equiv), AgF (3 equiv), MeCN [0.2 M] at 50 °C for 2–4 h. a) in DCM; b) ^19^F NMR yield vs. internal standard; c) **5** (1 equiv), *n*‐BuZnCl (2 equiv), [Pd(μ‐I)(P*^t^* Bu_3_)]_2_ (2.5 mol%), toluene, r.t., 10 min; d) **5** (1 equiv), 1‐ethynyl‐4‐methoxybenzene (1.3 equiv), PdCl_2_(PPh_3_)_2_ (5 mol%), PPh_3_ (10 mol%), CuI (1.5 equiv), DMF, 80 °C, 16 h; e) **5** (1 equiv), methanesulfonamide (1.2 equiv), [Pd(allyl)Cl]_2_ (1 mol%), *t*BuXPhos (4 mol%), K_2_CO_3_ (2 equiv), 2‐Me‐THF, 80 °C, 16 h; f) **11** (1 equiv), 2‐oxa‐6‐azaspiro[3.3]heptane (1.0 equiv), Pd(OAc)_2_ (20 mol%), P*^t^* Bu_3_ (20 mol%), Cs_2_CO_3_ (1.5 equiv), toluene, 105 °C, 18 h; g) Step 1: **6** (1 equiv), NaOH (2 equiv), THF/H_2_O (4:1), 80 °C, 1 h; Step 2: 2‐(diethylamino)ethan‐1‐ol (1.5 equiv), DCC (1.5 equiv), DMAP (0.1 equiv), DCM, r.t., 24 h (yield over 2 steps). BTC = bis(trichloromethyl) carbonate.

Furthermore, we explored the applicability of our method to synthesize *N*(CF_3_)(CF_2_H) derivatives of commercial drug molecules and obtained the *N*(CF_3_)(CF_2_H) analogues of tranylcypromine (**15**), an antidepressant, and mexiletine (**16**), which is employed for treating irregular heartbeats. Also, the *N*(CF_3_)(CF_2_H) analogue of the structurally more complex drug alogliptin (antidiabetic) **17** was synthesized in 70% yield, highlighting the generality and compatibility of our synthetic approach with diverse functionalities. Employment of deuterated *N*‐CF_3_ formamides^[^ ^18^ ^]^ delivers the corresponding *N*(CF_3_)(CF_2_D) deutero‐analogues (**7**, **12**), without any marked impact by the deuterium on the reaction efficiency and yield.

With the protocol to access *N*(CF_3_)(CF_2_H) amines established, we then investigated the compatibility of this moiety with common derivatization protocols (Scheme 1b). Diverse cross‐coupling reactions were feasible, such as alkylation, alkynylation, and amination under typical Negishi, Sonogashira, and C–N cross‐coupling conditions (**18**–**21**), which underscores the stability of the *N*(CF_3_)(CF_2_H) moiety toward highly basic, nucleophilic, metal‐catalyzed, and high temperature conditions. A derivative of procaine (**22**), a local anesthetic, was obtained in moderate yield by a two‐step strategy comprising an ester deprotection under basic conditions followed by an acidic work‐up, and then alcohol condensation starting from *N*(CF_3_)(CF_2_H) amine (**6**). Notably, while the amino acid derivative **10** did not deliver an anticipated de‐benzylation even under 50 bar H_2_ atmosphere over Pd/C, the *N*(CF_3_)(CF_2_H) moiety equally remained untouched under these high‐pressure conditions and the starting material was fully recovered with no signs of decomposition. These results reinforce the above stability tests and suggest high chemical robustness toward a wide array of synthetic manipulations.

In summary, we have developed an efficient, mild, and practical synthesis of *N*(CF_3_)(CF_2_H) amines via desulfurization–fluorination of *N*‐CF_3_ thioformamides. The method is characterized by operational simplicity and broad scope. Comprehensive stability assessments reveal that *N*(CF_3_)(CF_2_H) amines maintain their integrity for weeks across neutral and basic pH ranges, and also exhibit acceptable half‐lives under highly acidic conditions. Their high robustness is further underscored through compatibility with various chemical postfunctionalizations. With enhanced hydrogen bonding and comparable lipophilicity to *N*(CF_3_)(Me) amines paired with 2000 times higher stability, we anticipate numerous applications of this promising new scaffold in drug discovery and beyond.

## Conflict of Interests

The authors declare no conflict of interest.

## Supporting information



Supporting Information

## Data Availability

The data that support the findings of this study are available in the Supporting Information of this article.
