# Supplementary material for: Desulfurizative Fluorination of N‐CF3 Thioformamides for the Efficient Synthesis of N(CF3)(CF2H) Amines with Enhanced Stability
Source: Angew Chem Int Ed Engl. 2025 May 2;64(26):e202506154. doi: 10.1002/anie.202506154 (PMC12184305; doi:10.1002/anie.202506154)
Supplement: Supplementary file 1 — Supporting Information [file ANIE-64-e202506154-s001.pdf]

Contents

1. General Experimental Details 3

2. Optimization of Reaction Conditions 4

2.1 Desulfurization-fluorination reaction 4

3. Synthesis of *N*(CF_3)(CF_2H) and *N*(CF_3)(CF_2D) amines 5

3.1 General Procedure for the formation of *N*(CF_3)(CF_2H) amines (GP1) 5

3.2 Characterization Data of *N*(CF_3)(CF_2H) and *N*(CF_3)(CF_2D) amines 5

3.3 Derivatization reactions 12

4. Starting Material Synthesis 16

4.1 Synthesis of *N*–trifluoromethyl (deutero)thioformamides 16

4.1.1 General Procedure 2 (GP2) 16

4.1.2 Characterization Data of *N*–trifluoromethyl (deutero)thioformamides (S1-S17) 16

4.2 Synthesis of isothiocyanates 23

4.2.1 General Procedure 3 (GP3) 23

4.2.2 Characterization Data of isothiocyanates 23

4.3 Synthesis of *N*–trifluoromethyl carbamoyl fluorides 25

4.3.1 General Procedure 4 (GP4) 25

4.3.2 Characterization Data of *N*-trifluoromethyl carbamoyl fluorides 25

4.4 Synthesis of *N*–trifluoromethyl (deutero)formamides 27

4.4.1 General Procedure 5 (GP5) 27

4.4.2 Characterization Data of *N*-trifluoromethyl (deutero)formamides 27

5. Synthesis of *N*-CF_3 amine (1-Me) 30

6. Attempts to access other *N*-CF_3 and *N*-CF_2H functionalities 32

6.1 Unsuccessful attempts of desulfurization-fluorination protocols 32

6.2 Unsuccessful attempts of sulfurization reactions with Lawesson's reagent 32

7. Stability tests 33

7.1.1 Stability Study in buffer solution/MeCN 1:1 at pH 1.0, 7.4 and 10.1 33

8. Determination of Hansch-Leo parameter (π_R) 35

9. [*A*] value determination (Abrahams NMR method) 36

10. Enantioretention Study **37**

11. Computational details **40**

11.1 Computed log*P* values 40

11.1.1 XYZ coordinates of computed structures 41

12. NMR Spectra **43**

12.1 Products 43

12.2 Starting materials 76

12.2.1 *N*-CF_3 (deutero)thioformamides 76

12.2.2 Isothiocyanates 102

12.2.3 *N*-CF_3 carbamoyl fluoride 105

12.2.4 *N*-CF_3 (deutero)formamides 113

12.2.5 *N*-methyl-*N*-(trifluoromethyl)-[1,1'-biphenyl]-4-amine (1-Me) 128

13. References **130**

1. General Experimental Details

All reagents and starting materials were purchased from Sigma-Aldrich, Alfa Aesar, abcr, TCI, Fluorochem, BLDpharm or Acros Organics and used as received unless otherwise stated. Anhydrous toluene was obtained using an Innovative Technology PS-MD-5 solvent purification system. Solvents used in work up and purification were distilled prior to use. All the reactions were carried out in oven dried glassware.

Thin layer chromatography (TLC) was performed on Macherey Nagel ALUGRAM Xtra SIL G UV254 aluminium plates with unmodified silica and visualized either under UV light or stained with $KMnO_4$. Flash column chromatography was performed with silica gel (0.04 – 0.063 mm particle size) purchased from Macherey Nagel. Analytical HPLC of chiral compounds was performed on an Agilent 1260 Series instrument using chiral stationary phases (details on page S37 ff.). Stability studies were performed on the same HPLC instrument using an Agilent Poroshell 120 (50 x 4.6 mm) column.

All the ^{1}H, ^{13}C and ^{19}F NMR spectra were recorded on Bruker Avance Neo 600, Varian VNMRS 600, or Varian VNMRS 400 spectrometers at ambient temperature (unless otherwise specified). Chemical shifts (δ) are reported in parts per million (ppm) and were referenced either to residual solvent peak ($CDCl_3$, CD_2Cl_2, $C_6D_5CD_3$ for ^{1}H and ^{13}C spectra) or by the instrument internally after locking and shimming to the deuterated solvent (for ^{19}F). Coupling constants (*J*) are given in Hertz (Hz). Multiplicities of signals in ^{1}H, ^{19}F, and ^{13}C NMR were designated as s (singlet), d (doublet), dd (doublet of doublets), dt (doublet of triplets), ddd (doublet of doublets of doublets), t (triplet), td (triplet of doublets), q (quartet), p (quintet), h (sextet), hept (septet), and m (multiplet).

Gas chromatography coupled with mass spectrometry (GC-MS) was performed on an Agilent Technologies 5975 series MSD mass spectrometer under electron ionization (EI) mode coupled with an Agilent Technologies 7820A gas chromatograph employing an Agilent 19091s-433 HP-5MS column (30 m × 0.250 µm × 0.250 µm). High-resolution mass spectrometry (HRMS) was performed using a Thermo Scientific LTQ Orbitrap XL spectrometer (ESI), or Bruker Maxis II LC-MS-System (APCI). IR spectra were recorded on a Spectrum 100 spectrometer with a UATR Diamond/KRS-5 crystal with attenuated total reflectance (ATR).

2. Optimization of Reaction Conditions

2.1 Desulfurization-fluorination reaction

Under argon atmosphere, an amber-colored 4 mL vial was charged with the *N*–CF_3 thioformamide and solvent was added. Subsequently, AgF was added to the solution and the vial was closed tightly. The reaction mixture was stirred at 50 °C. After the indicated time, the vial was allowed to cool to room temperature, then internal standard was added, and conversion was determined by quantitative ^{19}F NMR.

Table S1. Optimization table for desulfurization-fluorination reaction.

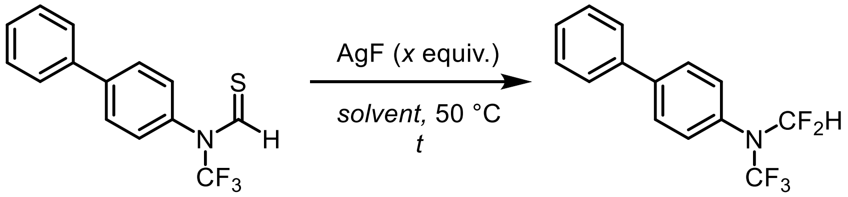

Entry	AgF (equiv.)	Solvent	time (h)	Conc. (M)	Scale (mmol)	Yield (%) [a]
1	2	MeCN	2	0.01	0.05	66
2	3	MeCN	2	0.01	0.05	71
3	2	DCM	2	0.01	0.05	94
4	3	DCM	2	0.01	0.05	90
5	3	MeCN	2	0.05	0.05	64
6	3	MeCN	1	0.2	0.05	63
7	3	MeCN	2	0.2	0.05	Quant.
8	3	MeCN	4	0.2	0.05	Quant.
9	3	MeCN	4	0.2	0.2	98 [b]

[a] Reaction mixture was analyzed by quantitative ^{19}F NMR; [b] Isolated yield.

3. Synthesis of *N*(CF_3)(CF_2H) and *N*(CF_3)(CF_2D) amines

3.1 General Procedure for the formation of *N*(CF_3)(CF_2H) amines (GP1)

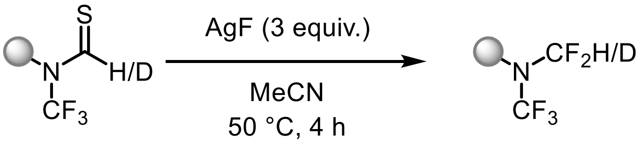

Under argon atmosphere an amber-colored 4 mL vial was charged with the *N*–CF_3 thioformamide (usually on a 0.20 mmol scale) and 1 mL MeCN was added [0.2 M]. Subsequently, AgF (3 equiv.) was added to the solution and the vial was closed tightly. The reaction mixture was stirred at 50 °C for 4h. Afterwards the reaction mixture was allowed to cool to ambient temperature. The crude mixture was filtered over a microscale column (glass pipette) filled with silica, eluting with 20 mL Et_2O/Pentane (1:1) to yield the *N*–bisfluoromethylated amine after evaporation of all volatiles without further purification.

Deuterated compounds (**7**, **12**) were obtained from the deuterated thioformamides following the general procedure.

3.2 Characterization Data of *N*(CF_3)(CF_2H) and *N*(CF_3)(CF_2D) amines

N-(difluoromethyl)-*N*-(trifluoromethyl)-[1,1'-biphenyl]-4-amine (1)

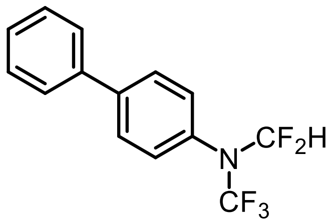

The reaction was performed on a 0.2 mmol scale according to GP1 and the title product was obtained after filtration over silica gel (20 mL, 50% Et_2O/*n*-pentane) as a white solid (56.2 mg, 0.196 mmol, 98%).
***R**$_f$* = 0.88 (10% Et_2O/*n*-pentane). **^{1}H NMR** (400 MHz, Methylene Chloride-*d*$_2$) δ 7.69 (d, *J* = 7.9 Hz, 2H), 7.62 (d, *J* = 7.6 Hz, 2H), 7.48 (dd, *J* = 3.5, 3.5 Hz, 4H), 7.44 – 7.35 (m, 1H), 6.75 (t, *J* = 59.9 Hz, 1H). **^{19}F NMR** (376 MHz, Methylene Chloride-*d*$_2$) δ -56.66 (t, *J* = 6.2 Hz, 3F), -93.96 (dq, *J* = 60.1, 6.2 Hz, 2F). **^{13}C{^{1}H} NMR** (151 MHz, Methylene Chloride-*d*$_2$) δ 143.4, 140.3, 131.3, 131.2, 129.5, 128.6, 128.5, 127.7, 121.3 (q, *J* = 258.9 Hz), 111.0 (t, *J* = 244.7 Hz). **IR** (neat, cm^{-1}): 3040, 2924, 2321, 2107, 1724, 1487, 1288, 1112, 1006, 949, 740, 696. **HRMS** (APCI) calculated for $C_{14}H_{10}NF_5$: 287.0728 $[M]^+$, found: 287.0735.

N-(difluoromethyl)-N-(trifluoromethyl)aniline (2)

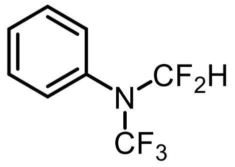

The reaction was performed on a 0.2 mmol scale according to GP1 using DCM as solvent and the title product was obtained after filtration over silica gel (20 mL, 50% Et_2O/*n*-pentane) as a colorless oil (22.0 mg, 0.104 mmol, 52%; 86% ^{1}H NMR yield, using 4-trifluoromethylanisole as an internal standard). *Note*: Given the high volatility of this compound, impurities identified as pentane are present in the isolated product.
***R**$_f$* = 0.57 (*n*-pentane). **^{1}H NMR** (400 MHz, Methylene Chloride-*d*$_2$) δ 7.50 – 7.43 (m, 3H), 7.42 – 7.38 (m, 2H), 6.71 (t, *J* = 60.1 Hz, 1H). **^{19}F NMR** (376 MHz, Methylene Chloride-*d*$_2$) δ -56.66 (t, *J* = 6.4 Hz, 3F), -

93.98 (dq, J = 60.1, 6.4 Hz, 2F). **^{13}C{^{1}H} NMR** (151 MHz, Methylene Chloride-d_2) δ 132.2, 130.8, 130.3, 129.8, 121.1 (q, J = 257.6 Hz), 110.8 (td, J = 244.8, 3.6 Hz). **IR** (neat, cm^{-1}): 2923, 2854, 2315, 2174, 2101, 1740, 1460, 1373, 1215, 875, 740. **HRMS** (APCI) calculated for $C_8H_6NF_5$: 211.0415 [M]$^+$, found: 211.0414.

4-cyclohexyl-*N*-(difluoromethyl)-*N*-(trifluoromethyl)aniline (3)

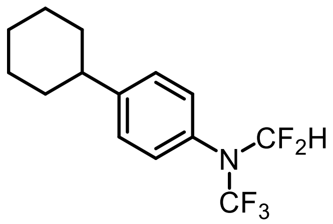

The reaction was performed on a 0.2 mmol scale according to GP1 and the title product was obtained after filtration over silica gel (20 mL, 50% Et_2O/*n*-pentane) colorless oil (51.0 mg, 0.173 mmol, 87%).
Rf = 0.78 (10% Et_2O/*n*-pentane). **^{1}H NMR** (600 MHz, Methylene Chloride-d_2) δ 7.31 (brs, 4H), 6.69 (t, J = 60.2 Hz, 1H), 2.64 – 2.53 (m, 1H), 1.93 – 1.82 (m, 4H), 1.79 – 1.73 (m, 1H), 1.48 – 1.38 (m, 4H), 1.34 – 1.24 (m, 1H). **^{19}F NMR** (376 MHz, Methylene Chloride-d_2) δ -56.76 (t, J = 6.3 Hz, 3F), -93.94 (dq, J = 60.3, 6.2 Hz, 2F). **^{13}C{^{1}H} NMR** (151 MHz, Methylene Chloride-d_2) δ 150.8, 130.8, 129.7, 128.4, 121.3 (q, J = 258.5 Hz), 113.8 – 108.1 (m), 44.8, 34.9, 27.3, 26.6. **IR** (neat, cm^{-1}): 2927, 2855, 2319, 2115, 1906, 1728, 1513, 1425, 1293, 1122, 1027, 952, 758, 715. **HRMS** (APCI) calculated for $C_{14}H_{16}NF_5$: 293.1196 [M]$^+$, found: 293.1197.

***N*-(difluoromethyl)-3,4,5-trimethoxy-*N*-(trifluoromethyl)aniline (4)**

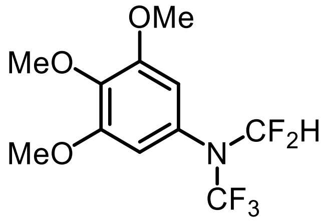

The reaction was performed on a 0.2 mmol scale according to GP1 and the title product was obtained after filtration over silica gel (20 mL, 50% Et_2O/*n*-pentane) as a pale-yellow solid (59.0 mg, 0.196 mmol, 98%).
Rf = 0.74 (20% EtOAc/*n*-pentane). **^{1}H NMR** (600 MHz, Methylene Chloride-d_2) δ 6.67 (t, J = 60.1 Hz, 1H), 6.61 (s, 2H), 3.84 (s, 6H), 3.81 (s, 3H). **^{19}F NMR** (564 MHz, Methylene Chloride-d_2) δ -56.81 (t, J = 6.4 Hz, 3F), -94.26 (dq, J = 60.1, 6.4 Hz, 2F). **^{13}C{^{1}H} NMR** (151 MHz, Methylene Chloride-d_2) δ 154.0, 139.9, 127.4, 121.1 (q, J = 258.7 Hz), 111.0 (tq, J = 244.8, 3.1 Hz), 108.3, 60.9, 56.6. **IR** (neat, cm^{-1}): 3305, 3020, 2939, 2844, 2288, 2112, 1985, 1814, 1714, 1596, 1507, 1459, 1419, 1368, 1278, 1232, 1175, 1119, 990, 890, 834,781, 742, 665. **HRMS** (ESI) calculated for $C_{11}H_{12}O_3NF_5$: 324.0630 [M+Na]$^+$, found: 324.0626.

4-bromo-2-chloro-*N*-(difluoromethyl)-*N*-(trifluoromethyl)aniline (5)

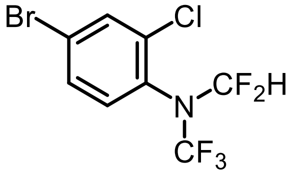

The reaction was performed on a 0.2 mmol scale according to GP1 using DCM as solvent and the title product was obtained after filtration over silica gel (20 mL, 50% Et_2O/*n*-pentane) as a colorless oil (33.0 mg, 0.101 mmol, 52%).
Rf = 0.73 (*n*-pentane). **^{1}H NMR** (600 MHz, Methylene Chloride-d_2) δ 7.75 (d, J = 2.2 Hz, 1H), 7.53 (dd, J = 8.5, 2.2 Hz, 1H), 7.40 (d, J = 8.5 Hz, 1H), 6.86 – 6.58 (m, 1H). **^{19}F NMR** (564 MHz, Methylene Chloride-d_2) δ -57.22 (t, J = 5.7 Hz, 3F), -88.94 (ddq, J = 216.3, 65.2, 5.5 Hz, 1F), -101.74 (ddq, J = 215.8, 54.7, 5.9 Hz, 1F). **^{13}C{^{1}H} NMR** (151 MHz, Methylene Chloride-d_2) δ 138.2, 134.3, 134.0, 131.5, 128.6, 125.4, 120.6 (qd, J = 259.8, 4.9 Hz), 110.5 (m). **IR** (neat, cm^{-1}):

3097, 2928, 2311, 2127, 1979, 1900, 1731, 1580, 1479, 1422, 1378, 1330, 1289, 1204, 1128, 1030, 953, 872, 806, 755, 720, 695. **HRMS** (APCI) calculated for $C_8H_4NBrClF_5$: 322.9130 $[M]^+$, found: 322.9137.

methyl 4-((difluoromethyl)(trifluoromethyl)amino)benzoate (6)

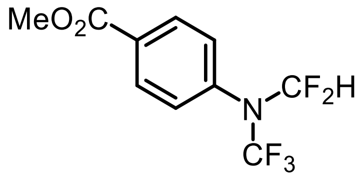

The reaction was performed on a 0.2 mmol scale according to GP1 and the title product was obtained after filtration over silica gel (20 mL, 50% Et_2O/*n*-pentane) as a colorless oil (51.1 mg, 0.19 mmol, 95%). ***R$_f$*** = 0.17 (2% EtOAc/*n*-pentane). **^{1}H NMR** (600 MHz, Methylene Chloride-*d_2*) δ 8.11 (m, 2H), 7.48 (d, *J* = 8.5 Hz, 2H), 6.73 (t, *J* = 59.8 Hz, 1H), 3.92 (s, 3H). **^{19}F NMR** (564 MHz, Methylene Chloride-*d_2*) δ -56.40 (t, *J* = 6.2 Hz, 3F), -93.97 (dq, *J* = 59.8, 6.2 Hz, 2F). **^{13}C{^{1}H} NMR** (151 MHz, Methylene Chloride-*d_2*) δ 166.3, 136.2, 132.0, 131.0, 130.3, 120.9 (q, *J* = 260.0 Hz), 110.7 (tq, *J* = 245.6, 3.4 Hz), 52.7. **IR** (neat, cm^{-1}): 3007, 2957, 2325, 2099, 1933, 1727, 1612, 1512, 1436, 1278, 1194, 1111, 1026, 953, 863, 831, 808, 763, 733, 658. **HRMS** (APCI) calculated for $C_{10}H_8NO_2F_5$: 269.0470 $[M]^+$, found: 269.0471.

***N*-(difluoromethyl-*d*)-*N*-(trifluoromethyl)-[1,1'-biphenyl]-4-amine (7)**

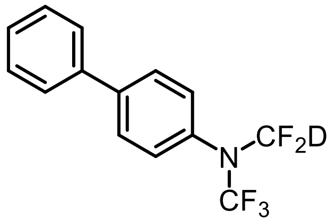

The reaction was performed on a 0.1 mmol scale according to GP1 and the title product was obtained after filtration over silica gel (20 mL, 50% Et_2O/*n*-pentane) as a white solid (25.2 mg, 0.087 mmol, 87%). ***R$_f$*** = 0.88 (10% Et_2O/*n*-pentane). **^{1}H NMR** (400 MHz, Methylene Chloride-*d_2*) δ 7.68 (d, *J* = 8.5 Hz, 2H), 7.64 – 7.59 (m, 2H), 7.47 (ddd, *J* = 7.2, 2.9, 2.9 Hz, 4H), 7.44 – 7.35 (m, 1H), 6.82 (d, *J* = 60.2 Hz, 0.14H). **^{19}F NMR** (376 MHz, Methylene Chloride-*d_2*) δ -56.69 (t, *J* = 6.2 Hz), -93.96 (dq, *J* = 60.1, 6.1 Hz), -94.63 – 94.78 (m). **^{13}C{^{1}H} NMR** (151 MHz, Methylene Chloride-*d_2*) δ 143.4, 140.3, 131.3, 131.2, 129.5, 128.6, 128.5, 127.7, 121.3 (q, *J* = 258.7 Hz), 110.4 (t, *J* = 248.9 Hz). **IR** (neat, cm^{-1}): 2924, 2189, 2098, 1974, 1737, 1488, 1335, 1291, 1114, 953, 740, 693. **HRMS** (APCI) calculated for $C_{14}H_9{}^2HNF_5$: 288.0791 $[M]^+$, found: 288.0795.

***tert*-butyl 4-((difluoromethyl)(trifluoromethyl)amino)piperidine-1-carboxylate (8)**

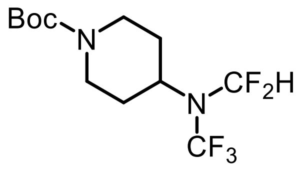

The reaction was performed on a 0.2 mmol scale according to GP1 (reaction time = 2 h) and the title product was obtained after filtration over silica gel (20 mL, 50% Et_2O/*n*-pentane) colorless oil (62.8 mg, 0.197 mmol, 99%). ***R$_f$*** = 0.59 (10% Et_2O/*n*-pentane). **^{1}H NMR** (400 MHz, Methylene Chloride-*d_2*) δ 6.51 (t, *J* = 59.3 Hz, 1H), 4.30 – 4.10 (m, 2H), 3.54 (t, *J* = 11.9 Hz, 1H), 2.70 (s, 2H), 1.93 – 1.69 (m, 4H), 1.43 (s, 9H). **^{19}F NMR** (376 MHz, Methylene Chloride-*d_2*) δ -54.36 (t, *J* = 6.4 Hz, 3F), -95.34 (dq, *J* = 59.6, 6.7 Hz, 2F). **^{13}C{^{1}H} NMR** (151 MHz, Methylene Chloride-*d_2*) δ 154.8, 122.5 (qt, *J* = 259.4, 2.6 Hz), 112.1 (tq, *J* = 242.2, 4.8 Hz), 80.0, 53.3, 44.4 (br), 32.1, 28.6. **IR** (neat, cm^{-1}): 2976, 2939, 2866, 2326, 2086, 1920, 1775, 1691, 1424, 1361, 1293, 1166, 1109, 1012, 891, 768, 726, 668. **HRMS** (ESI) calculated for $C_{12}H_{19}O_2N_2F_5$: 341.1259 $[M+Na]^+$, found: 341.1253.

tert-butyl N-(difluoromethyl)-N-(trifluoromethyl)-L-phenylalaninate (9)

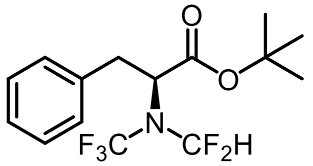

The reaction was performed on a 0.2 mmol scale according to GP1 and the title product was obtained after filtration over silica gel (20 mL, 50% Et_2O/*n*-pentane) colorless oil (62.9 mg, 0.185 mmol, 93%).

***R*$_f$** = 0.54 (10% Et_2O/*n*-pentane). **^{1}H NMR** (600 MHz, Methylene Chloride-*d*$_2$) δ 7.35 – 7.29 (m, 2H), 7.28 – 7.19 (m, 3H), 6.51 (td, *J* = 58.4, 2.7 Hz, 1H), 4.23 (t, *J* = 7.8 Hz, 1H), 3.35 (dd, *J* = 14.3, 7.4 Hz, 1H), 3.02 (dd, *J* = 14.4, 7.9 Hz, 1H), 1.42 (s, 9H). **^{19}F NMR** (376 MHz, Methylene Chloride-*d*$_2$) δ -54.77 (t, *J* = 7.4 Hz, 3F), -94.11 (dq, *J* = 58.3, 7.3 Hz, 1F), -94.93 (dq, *J* = 58.2, 8.0 Hz, 1F). **^{13}C{^{1}H} NMR** (151 MHz, Methylene Chloride-*d*$_2$) δ 168.9, 137.1, 129.9, 129.0, 127.5, 122.1 (q, *J* = 260.1 Hz), 111.3 (tm, *J* = 244.7 Hz), 83.4, 58.6, 37.6, 27.9. **IR** (neat, cm^{-1}): 3032, 2930, 1737, 1448, 1279, 1125, 1030, 968, 842, 747, 699. **HRMS** (ESI) calculated for $C_{15}H_{18}O_2NF_5$: 362.1150 [M+Na]$^+$, found: 362.1157.

benzyl N-(difluoromethyl)-N-(trifluoromethyl)-L-leucinate (10)

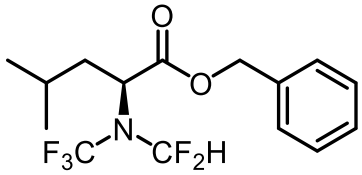

The reaction was performed on a 3.0 mmol scale according to GP1. The title product was obtained after filtration over silica gel (20 mL, DCM) in technical purity as a slightly yellow oil (979.0 mg, 2.88 mmol, 96% [~90% purity]). NMR spectra were obtained from a fraction purified by column chromatography (5% Et_2O/*n*-pentane).

***R*$_f$** = 0.35 (5% Et_2O/*n*-pentane). **^{1}H NMR** (600 MHz, Methylene Chloride-*d*$_2$) δ 7.47 – 7.24 (m, 5H), 6.56 (t, *J* = 58.5 Hz, 1H), 5.19 (s, 2H), 4.21 (t, *J* = 7.0 Hz, 1H), 1.94 – 1.81 (m, 1H), 1.81 – 1.66 (m, 2H), 0.96 (dd, *J* = 12.2, 6.4 Hz, 6H). **^{19}F NMR** (565 MHz, Methylene Chloride-*d*$_2$) δ -54.81, -92.96 (ddq, *J* = 214.5, 59.2, 6.8 Hz), -95.50 (ddq, *J* = 214.7, 58.1, 7.9 Hz). **^{13}C{^{1}H} NMR** (151 MHz, Methylene Chloride-*d*$_2$) δ 170.9, 136.0, 129.2, 129.0, 128.7, 122.1 (q, *J* = 261.5, 260.8 Hz), 111.4 (t, *J* = 243.2 Hz), 68.1, 40.0, 25.1, 22.9, 21.7. **IR** (neat, cm^{-1}): 3037, 2962, 2876, 2325, 2089, 1945, 1747, 1446, 1282, 1116, 1027, 804, 736, 696. **HRMS** (ESI) calculated for $C_{15}H_{18}O_2NF_5$: 362.1150 [M+Na]$^+$, found: 362.1135.

N-(4-bromobenzyl)-N-(difluoromethyl)-1,1,1-trifluoromethanamine (11)

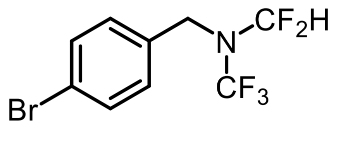

The reaction was performed on a 0.2 mmol scale according to GP1 and the title product was obtained after filtration over silica gel (20 mL, 50% Et_2O/*n*-pentane) as a colorless oil (56.4 mg, 0.185 mmol, 93%).

***R*$_f$** = 0.39 (*n*-pentane). ^{1}**H NMR** (400 MHz, Methylene Chloride-*d*$_2$) δ 7.50 (dd, *J* = 8.3, 1.4 Hz, 2H), 7.23 (d, *J* = 8.2 Hz, 2H), 6.62 (td, *J* = 60.0, 1.2 Hz, 1H), 4.33 (s, 2H). **^{19}F NMR** (376 MHz, Methylene Chloride-*d*$_2$) δ -59.31 (t, *J* = 5.5 Hz, 3F), -97.63 (dq, *J* = 60.2, 5.5 Hz, 2F). **^{13}C{^{1}H} NMR** (151 MHz, Methylene Chloride-*d*$_2$) δ 136.0, 132.1, 129.8, 122.3 (qt, *J* = 259.1, 3.9 Hz), 122.1, 111.5 (tq, *J* = 243.7, 4.6 Hz), 43.8. **IR** (neat, cm^{-1}): 3039, 2929, 2320, 2113, 2006, 1893, 1717, 1596, 1490, 1433, 1343, 1296, 1235, 1140, 1109, 1074, 1004, 934, 835, 789, 722, 668. **HRMS** (APCI) calculated for $C_9H_7BrNF_5$: 302.9677 [M]$^+$, found: 302.9680.

tert-butyl 4-((difluoromethyl-d)(trifluoromethyl)amino)piperidine-1-carboxylate (12)

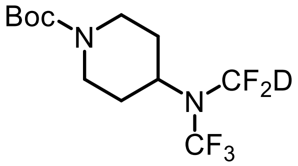

The reaction was performed on a 0.087 mmol scale according to GP1 and the title product was obtained after filtration over silica gel (20 mL, 50% Et_2O/n-pentane) as a colorless oil (24.2 mg, 0.76 mmol, 86%, 88% deuteration). R_f = 0.59 (10% Et_2O/n-pentane). **^{1}H NMR** (600 MHz, Methylene Chloride-d_2) δ 6.51 (t, J = 59.4 Hz, 0.12 H), 4.19 (s, 2H), 3.52 (s, 1H), 2.70 (s, 2H), 1.93 – 1.72 (m, 4H), 1.43 (s, 9H). **^{19}F NMR** (376 MHz, Methylene Chloride-d_2) δ -54.37, -95.31 (dq, J = 59.4, 6.0 Hz), -95.98 – -96.06 (m). **^{13}C{^{1}H} NMR** (151 MHz, Methylene Chloride-d_2) δ 154.2, 121.9 (q, J = 259.5, 259.1 Hz), 111.5 (t), 79.4, 52.7, 31.5, 29.7, 28.0. **IR** (neat, cm^{-1}): 2975, 2937, 2864, 2289, 2092, 1928, 1691, 1426, 1362, 1292, 1167, 1111, 1017, 968, 939, 887, 861, 767, 719. **HRMS** (APCI) calculated for $C_{12}H_{18}{}^2HO_2N_2F_5$: 262.0725 [M-$C_4H_9$]$^+$, found: 262.0730.

N-(difluoromethyl)-9-ethyl-N-(trifluoromethyl)-9H-carbazol-3-amine (13)

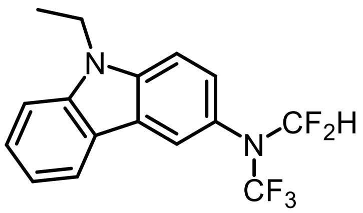

The reaction was performed on a 0.2 mmol scale according to GP1 and the title product was obtained after filtration over silica gel (20 mL, 50% Et_2O/n-pentane) as a dark yellow oil (59.0 mg, 0.18 mmol, 98%). R_f = 0.42 (5% EtOAc/n-pentane). **^{1}H NMR** (600 MHz, Methylene Chloride-d_2) δ 8.16 (d, J = 1.9 Hz, 1H), 8.13 (d, J = 7.7 Hz, 1H), 7.54 (ddd, J = 8.3, 7.1, 1.2 Hz, 1H), 7.51 – 7.46 (m, 3H), 7.29 (ddd, J = 7.9, 7.1, 1.0 Hz, 1H), 6.82 (t, J = 60.4 Hz, 1H), 4.39 (q, J = 7.3 Hz, 2H), 1.44 (t, J = 7.3 Hz, 3H). **^{19}F NMR** (564 MHz, Methylene Chloride-d_2) δ -56.77 (t, J = 6.6 Hz, 3F), -93.80 (dq, J = 60.4, 6.6 Hz, 2F). **^{13}C{^{1}H} NMR** (151 MHz, Methylene Chloride-d_2) δ 141.0, 140.6, 128.2, 126.9, 123.7, 123.4, 122.8, 122.7, 121.5 (q, J = 257.8 Hz), 121.0, 119.9, 111.1 (tq, J = 243.4, 3.4 Hz), 109.4, 109.3, 38.2, 14.0. **IR** (neat, cm^{-1}): 3056, 2979, 2935, 2281, 2117, 1794, 1723, 1599, 1484, 1424, 1309, 1230, 1117, 1022, 954, 887, 839, 806, 747, 724, 666. **HRMS** (APCI) calculated for $C_{16}H_{13}N_2F_5$: 328.0993 [M]$^+$, found: 328.0993.

methyl 4-((difluoromethyl)(trifluoromethyl)amino)thiophene-2-carboxylate (14)

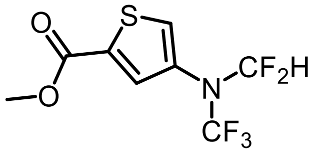

The reaction was performed on a 0.2 mmol scale according to GP1 (reaction time = 2 h) and the title product was obtained after filtration over silica gel (20 mL, 50% Et_2O/n-pentane) as a yellow oil (41.3 mg, 0.150 mmol, 75%). R_f = 0.24 (10% Et_2O/n-pentane). **^{1}H NMR** (600 MHz, Methylene Chloride-d_2) δ 7.74 (s, 1H), 7.67 (s, 1H), 6.69 (t, J = 60.0 Hz, 1H), 3.89 (s, 3H). **^{19}F NMR** (376 MHz, Methylene Chloride-d_2) δ -58.32 (t, J = 5.7 Hz, 3F), -95.49 (dq, J = 60.0, 5.5 Hz, 2F). **^{13}C{^{1}H} NMR** (151 MHz, Methylene Chloride-d_2) δ 162.1, 134.3, 133.5, 132.8, 129.5, 120.7 (q, J = 259.6 Hz), 113.0 – 107.7 (m), 52.9. **IR** (neat, cm^{-1}): 3112, 2958, 2852, 2276, 1902, 1719, 1546, 1453, 1385, 1254, 1127, 1033, 866, 744, 658. **HRMS** (ESI) calculated for $C_8H_6O_2NF_5S$: 297.9932 [M+Na]$^+$, found: 297.9935.

trans-N-(difluoromethyl)-2-phenyl-N-(trifluoromethyl)cyclopropan-1-amine (15)

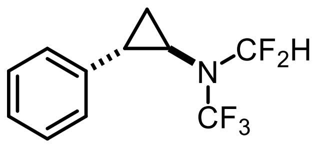

The reaction was performed on a 0.1 mmol scale according to GP1 in DCM due to the volatility of this product. The title product was obtained after filtration over silica gel (20 mL, DCM) as a volatile colorless oil (90% ^{1}H NMR yield, using 4-trifluoromethylanisole as an internal standard).

R_f = 0.83 (5% Et_2O/*n*-pentane). **^{1}H NMR** (400 MHz, Methylene Chloride-*d_2*) δ 7.31 (t, *J* = 7.6 Hz, 2H), 7.27 – 7.20 (m, 1H), 7.15 (d, *J* = 7.5 Hz, 2H), 6.52 (td, *J* = 59.5, 1.8 Hz, 1H), 2.65 – 2.55 (m, 1H), 2.43 (ddd, *J* = 10.1, 6.8, 3.1 Hz, 1H), 1.59 – 1.43 (m, 1H), 1.34 (q, *J* = 6.8 Hz, 1H). **^{19}F NMR** (376 MHz, Methylene Chloride-*d_2*) δ -59.78 (t, *J* = 6.4 Hz, 3F), -98.70 (dt, *J* = 59.4, 6.2 Hz, 2F). **^{13}C{^{1}H} NMR** (151 MHz, Methylene Chloride-*d_2*) δ 139.7, 129.1, 127.2, 127.1, 122.8 (q, *J* = 261.2 Hz), 113.0 (t, *J* = 245.6 Hz), 32.1, 24.0, 14.4. **IR** (neat, cm^{-1}): 3468, 3019, 2929, 2864, 1717, 1613, 1284, 1216, 1114, 1047, 758, 669. **HRMS** (APCI) calculated for $C_{11}H_{10}NF_5$: 251.0728 $[M]^+$, found: 251.0727.

N-(difluoromethyl)-1-(2,6-dimethylphenoxy)-N-(trifluoromethyl)propan-2-amine (16)

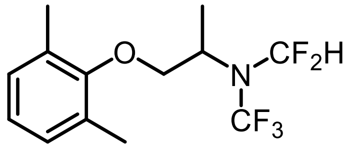

The reaction was performed on a 0.1 mmol scale according to GP1 and the title product was obtained after filtration over silica gel (20 mL, 50% Et_2O/*n*-pentane) as a yellow oil (24.2 mg, 0.081 mmol, 81%).

R_f = 0.69 (10% Et_2O/*n*-pentane). **^{1}H NMR** (600 MHz, Methylene Chloride-*d_2*) δ 7.01 (d, *J* = 7.4 Hz, 2H), 6.93 (t, *J* = 7.4 Hz, 1H), 6.64 (t, *J* = 59.1 Hz, 1H), 4.05 (q, *J* = 6.4 Hz, 1H), 3.84 (d, *J* = 5.7 Hz, 2H), 2.26 (s, 6H), 1.56 (d, *J* = 7.0 Hz, 3H). **^{19}F NMR** (376 MHz, Methylene Chloride-*d_2*) δ -54.89 (t, *J* = 7.3 Hz), -94.34 (ddq, *J* = 216.9, 60.2, 7.0 Hz), -95.73 (ddq, *J* = 216.5, 57.6, 7.7 Hz). **^{13}C{^{1}H} NMR** (151 MHz, Methylene Chloride-*d_2*) δ 155.5, 131.4, 129.5, 124.7, 122.5 (q, *J* = 259.2 Hz), 112.2 (ddd, *J* = 245.2, 241.6, 3.7 Hz), 74.5, 50.6, 17.0, 16.4. **IR** (neat, cm^{-1}): 2928, 2323, 2111, 1923, 1715, 1473,1314, 1199, 1138, 1002, 770. **HRMS** (APCI) calculated for $C_{13}H_{16}NF_5O$: 180.1388 $[M+H-C_2F_5]^+$, found: 180.1386. *Note*: The compound was found to be unstable under all HRMS conditions tested and fragmented with all used methods including APCI, ESI and different GC-MS methods.

(R)-2-((6-(3-((difluoromethyl)(trifluoromethyl)amino)piperidin-1-yl)-3-methyl-2,4-dioxo-3,4-dihydropyrimidin-1(2H)-yl)methyl)benzonitrile (17)

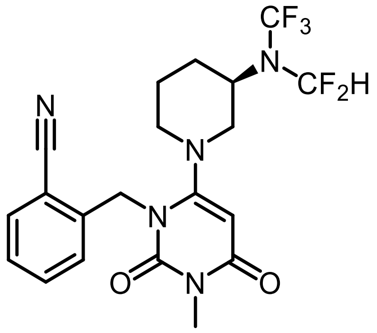

The reaction was performed on a 0.1 mmol scale according to GP1 employing 6 equiv. of AgF. The title product was obtained after column chromatography (75% EtOAc/*n*-pentane) as a colorless oil (33.1 mg, 0.070 mmol, 70%). *Note*: Impurities identified as the formamide starting material are present in the isolated product, quantified as 15% by ^{1}H NMR. The yield has been corrected accordingly. A pure sample for analytic measurements was obtained after further purification by preparative thin layer chromatography (70% EtOAc/*n*-pentane).

R$_f$ = 0.53 (70% EtOAc/*n*-pentane). **^{1}H NMR** (600 MHz, Methylene Chloride-d$_2$) δ 7.69 (dd, *J* = 7.7, 1.0 Hz, 1H), 7.56 (ddd, *J* = 7.8, 7.8, 1.2 Hz, 1H), 7.39 (dd, *J* = 7.6, 7.6 Hz, 1H), 7.15 (d, *J* = 7.9 Hz, 1H), 6.48 (t, *J* = 59.3 Hz, 1H), 5.38 (s, 1H), 5.29 (d, *J* = 15.9 Hz, 1H), 5.19 (d, *J* = 16.0 Hz, 1H), 3.54 (tt, *J* = 11.8, 3.7 Hz, 1H), 3.25 (s, 3H), 3.18 – 3.13 (m, 1H), 3.08 (d, *J* = 13.3 Hz, 1H), 2.83 (t, *J* = 11.3 Hz, 1H), 2.52 (td, *J* = 12.1, 2.0 Hz, 1H), 2.08 (d, *J* = 12.6 Hz, 1H), 1.87 – 1.64 (m, 3H). **^{19}F NMR** (376 MHz, Methylene Chloride-*d*$_2$) δ -54.36 (t, *J* = 5.5 Hz, 3F), -95.03 (dq, *J* = 59.0, 5.5 Hz, 2F). **^{13}C{^{1}H} NMR** (151 MHz, Methylene Chloride-*d*$_2$) δ 163.2, 159.6, 153.0, 141.3, 133.7, 133.7, 128.4, 127.2, 122.1 (q, *J* = 262.0 Hz), 117.6, 111.8 (td, *J* = 243.3, 5.2 Hz), 111.4, 91.7, 56.0, 52.2, 51.4, 46.6, 29.6, 28.2, 25.3. **IR** (neat, cm^{-1}): 2923, 2855, 2275, 2226, 2165, 2080, 2044, 2007, 1979, 1950, 1704, 1652, 1440, 1358, 1302, 1221, 1102, 1021, 977, 809, 763, 729. **HRMS** (ESI) calculated for $C_{20}H_{20}N_5F_5O_2$: 480.1429 [M+Na]$^+$, found: 480.1438.

3.3 Derivatization reactions

4-butyl-2-chloro-*N*-(difluoromethyl)-*N*-(trifluoromethyl)aniline (18)

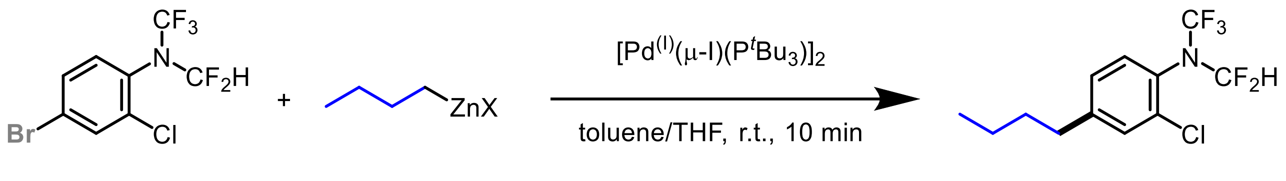

4 17, 78%

To an oven-dried 4 mL vial with a septum cap under Ar atmosphere were added a solution of *n*-butyl magnesium chloride (2M in Et_2O, 0.20 mL, 0.2 mmol, 2 equiv.), $ZnCl_2$ (1M in THF, 0.44 mL, 0.44 mmol, 2.2 equiv.) and LiCl (0.5M in THF, 0.88 mL, 0.44 mmol, 2.2 equiv.) and stirred for 15 minutes. This mixture was subsequently added dropwise over 10 minutes to a stirred solution of 4-bromo-2-chloro-*N*–(difluoromethyl)–*N*–(trifluoromethyl)aniline (63.5 mg, 0.20 mmol, 1 equiv.), and $[Pd(\mu\text{-}I)(P^tBu_3)]_2$ (4.4 mg, 0.005 mmol, 2.5 mol%) in 0.8 mL of anhydrous toluene. After completion of the addition the reaction mixture was stirred for an additional 10 minutes in air. Thereafter, the reaction mixture was diluted with 1 mL of pentane and the formed precipitate was removed by filtration through a short plug of silica. The filtrate was concentrated in vacuo and the crude was then purified by flash column chromatography, eluting with pentane. The title product was isolated as a colorless oil (46.0 mg, 0.15 mmol, 78%).

R R_f = 0.76 (*n*-pentane). **^{1}H NMR** (600 MHz, Methylene Chloride-d_2) δ 7.41 (d, *J* = 8.1 Hz, 1H), 7.39 (d, *J* = 2.0 Hz, 1H), 7.19 (dd, *J* = 8.1, 2.0 Hz, 1H), 6.81 – 6.57 (m, 1H), 2.67 – 2.62 (m, 2H), 1.66 – 1.58 (m, 2H), 1.37 (h, *J* = 7.4 Hz, 2H), 0.94 (t, *J* = 7.4 Hz, 3H). **^{19}F NMR** (564 MHz, Methylene Chloride-d_2) δ -57.12 (t, *J* = 5.9 Hz, 3F), -88.84 (ddq, *J* = 215.8, 64.7, 6.0 Hz, 1F), -101.32 (ddq, *J* = 215.4, 55.6, 6.1 Hz, 1F). **^{13}C{^{1}H} NMR** (151 MHz, Methylene Chloride-d_2) δ 147.9, 136.5, 132.9, 130.9, 128.3, 126.8, 120.9 (qd, *J* = 258.9, 4.5 Hz), 110.9 (m), 35.5, 33.5, 22.7, 14.0. **IR** (neat, cm^{-1}): 2934, 2867, 2326, 2087, 1604, 1564, 1498, 1463, 1426, 1331, 1293, 1207, 1162, 1125, 1029, 955, 879, 765, 704. **HRMS** (APCI) calculated for $C_{12}H_{13}NClF_5$: 301.0651 $[M]^+$, found: 301.0661.

2-chloro-*N*-(difluoromethyl)-4-((4-methoxyphenyl)ethynyl)-*N*-(trifluoromethyl)aniline (19)

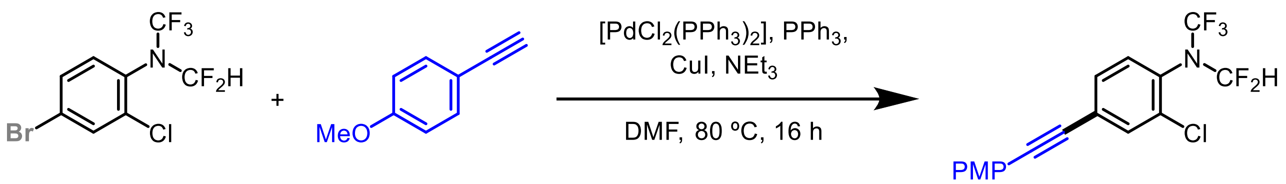

4 18, 80%

Inside an argon-filled glovebox, an oven-dried 4 mL vial was charged with 4-bromo-2-chloro-*N*–difluoromethyl)–*N*–(trifluoromethyl)aniline (63.9 mg, 0.2 mmol, 1 equiv.), $PdCl_2(PPh_3)_2$ (7.0 mg, 0.01 mmol, 5 mol%), PPh_3 (5.3 mg, 0.02 mmol, 10 mol%), CuI (3.8 mg, 0.02 mmol, 1.5 equiv.) and DMF (2 mL). To this mixture, Et_3N (57.0 µL, 0.56 mmol, 2.8 equiv.) and 1-ethynyl-4-methoxybenzene (34 mg, 0.26 mmol, 1.3 equiv.) were added. The reaction mixture was stirred at 80 °C for 16 h. Then the reaction

mixture was allowed to cool to room temperature and water (10 mL) was added, and the mixture was extracted with Et_2O (3 x 5 mL). The organic phase was dried over $MgSO_4$, concentrated in vacuo and purified by flash column chromatography, eluting with 2.5% EtOAc/pentane. The title product was isolated as a colorless solid (58.8 mg, 0.16 mmol, 80%).

R_f = 0.46 (2.5% EtOAc/*n*-pentane). **^{1}H NMR** (600 MHz, Methylene Chloride-d_2) δ 7.70 (s, 1H), 7.54 – 7.47 (m, 4H), 6.94 – 6.89 (m, 2H), 6.84 – 6.60 (m, 1H), 3.83 (s, 3H). **^{19}F NMR** (564 MHz, Methylene Chloride-d_2) δ -56.99 (t, *J* = 5.8 Hz, 3F), -88.84 (ddq, *J* = 216.3, 64.8, 5.9 Hz, 1F), -101.35 (ddq, *J* = 215.4, 55.1, 6.1 Hz, 1F). **^{13}C{^{1}H} NMR** (151 MHz, Methylene Chloride-d_2) δ 160.8, 137.0, 133.8, 133.5, 133.1, 130.9, 128.7, 128.0, 120.8 (qd, *J* = 259.3, 4.0 Hz), 114.6, 114.6, 110.8 (m), 93.1, 85.9, 55.7. **IR** (neat, cm^{-1}): 3009, 2938,2841, 2319, 2225, 2090, 1992, 1887, 1596, 1511, 1428, 1336, 1288, 1249, 1205, 1127, 1028, 955, 890, 831, 809, 766, 704. **HRMS** (APCI) calculated for $C_{17}H_{11}ONClF_5$: 375.0444 $[M]^+$, found: 375.0440.

_N_-(3-chloro-4-((difluoromethyl)(trifluoromethyl)amino)phenyl)methanesulfonamide (20)

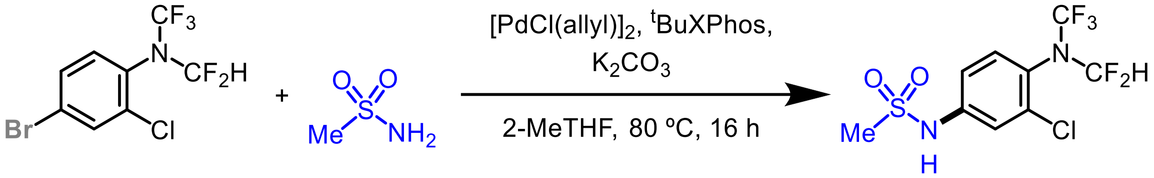

4 **19**, 63%

Inside an argon-filled glovebox, an oven-dried 4 mL vial was charged with $[Pd(allyl)Cl]_2$ (0.8 mg, 0.002 mmol, 1 mol%), ^{t}BuXPhos (3.4 mg, 0.008 mmol, 4 mol%) and K_2CO_3 (55.2 mg, 0.4 mmol, 2 equiv.) and methanesulfonamide (22.9 mg. 0.24 mmol, 1.2 equiv.). To this mixture, a solution of 4-bromo-2-chloro-*N*–(difluoromethyl)–*N*–(trifluoromethyl)aniline (60.2 mg, 0.19 mmol, in 2-Me-THF (0.8 mL) was added. The reaction mixture was stirred at 80 °C for 16 h. Then, the reaction mixture was allowed to cool to room temperature, filtered over a short pad of celite and the filtrate was evaporated to dryness. The crude was purified by flash column chromatography by directly loading it onto silica, eluting with 30% EtOAc/*n*-pentane. The title product was isolated as a colorless solid (39.3 mg, 0.12 mmol, 63%).

R_f = 0.38 (30% EtOAc/*n*-pentane). **^{1}H NMR** (600 MHz, Methylene Chloride-d_2) δ 7.49 (d, *J* = 8.7 Hz, 1H), 7.44 (d, *J* = 2.6 Hz, 1H), 7.19 (dd, *J* = 8.7, 2.6 Hz, 1H), 7.00 (brs, 1H), 6.83 – 6.59 (m, 1H), 3.12 (s, 3H). **^{19}F NMR** (564 MHz, Methylene Chloride-d_2) δ -57.26 (t, *J* = 5.8 Hz, 3F), -88.97 (ddq, *J* = 215.8, 64.8, 5.6 Hz, 1F), -101.53 (ddq, *J* = 215.8, 55.3, 5.9 Hz, 1F). **^{13}C{^{1}H} NMR** (151 MHz, Methylene Chloride-d_2) δ 140.3, 138.3, 134.3, 125.4, 120.9, 120.7 (dq, *J* = 259.6, 5.3 Hz), 118.2, 110.7 (m), 40.5. **IR** (neat, cm^{-1}): 3247, 3032, 2937, 2855, 2325, 2078, 1997, 1917, 1721, 1607, 1500, 1420, 1290, 1222, 1119, 1051, 1016, 953, 856, 836, 751, 705, 661. **HRMS** (ESI) calculated for $C_9H_8N_2ClF_5O_2S$: 360.9807 $[M+Na]^+$, found: 360.9803.

***N*-(4-(2-oxa-6-azaspiro[3.3]heptan-6-yl)benzyl)-*N*-(difluoromethyl)-1,1,1-trifluoromethanamine (21)**

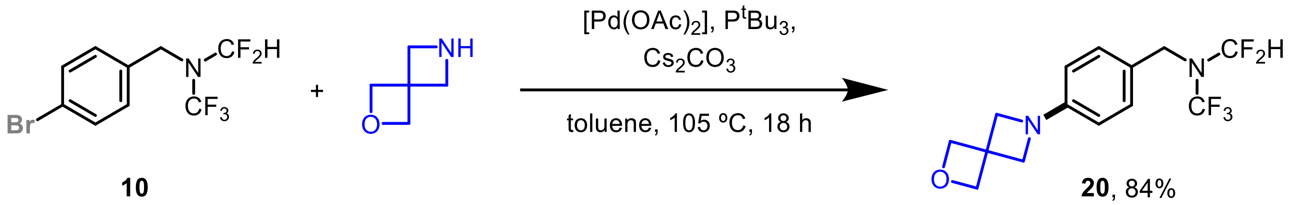

10 **20**, 84%

Inside an argon-filled glovebox, an oven-dried 4 mL vial was charged with $Pd(OAc)_2$ (7.7 mg, 0.034 mmol, 20 mol%), P^tBu_3 (6.9 mg, 0.034 mmol, 20 mol%) and Cs_2CO_3 (83.0 mg, 0.25 mmol, 1.5 equiv.). A solution of *N*-(4-bromobenzyl)-*N*-(difluoromethyl)-1,1,1-trifluoromethanamine (52.1 mg, 0.17 mmol, 1.0 equiv.) in 1 mL toluene and a solution of 2-oxa-6-azaspiro[3.3]heptane (25.4 mg, 0.25 mmol, 1.5 equiv.) in 1 mL toluene were subsequently added. The reaction mixture was stirred at 105 °C for 18 h. The reaction mixture was allowed to cool to room temperature and directly loaded onto the column for purification by flash column chromatography, eluting with 50% Et_2O/pentane. The title product was isolated as a colorless solid (45.9 mg, 0.14 mmol, 84%). *Note*: Traces of impurities identified as OP^tBu_3 are present in the isolated product, quantified as 2.5% by ^1H NMR. The yield has been corrected accordingly.

R_f = 0.39 (50% Et_2O/*n*-pentane). **^{1}H NMR** (600 MHz, Methylene Chloride-*d*$_2$) δ 7.17 (m, 2H), 6.55 (t, *J* = 60.1 Hz, 1H), 6.43 (m, 2H), 4.79 (s, 4H), 4.26 (s, 2H), 4.01 (s, 4H). **^{19}F NMR** (564 MHz, Methylene Chloride-*d*$_2$) δ -58.73 (t, *J* = 5.8 Hz, 3F), -97.16 (dq, *J* = 60.0, 5.7 Hz, 2F). **^{13}C{^{1}H} NMR** (151 MHz, Methylene Chloride-*d*$_2$) δ 151.5, 129.2, 125.4, 122.4 (qt, *J* = 259.7, 3.7 Hz), 111.9, 111.7 (tq, *J* = 243.0, 4.7 Hz), 81.4, 62.0, 44.4, 39.5. **IR** (neat, cm^{-1}): 2931, 2868, 2321, 2074, 1878, 1727, 1613, 1520, 1464, 382, 1297, 1243, 1160, 1101, 999, 969, 821, 792, 722, 674. **HRMS** (APCI) calculated for $C_{14}H_{15}ON_2F_5$: 323.1177 $[M+H]^+$, found: 323.1177.

2-(diethylamino)ethyl 4-((difluoromethyl)(trifluoromethyl)amino)benzoate (22)

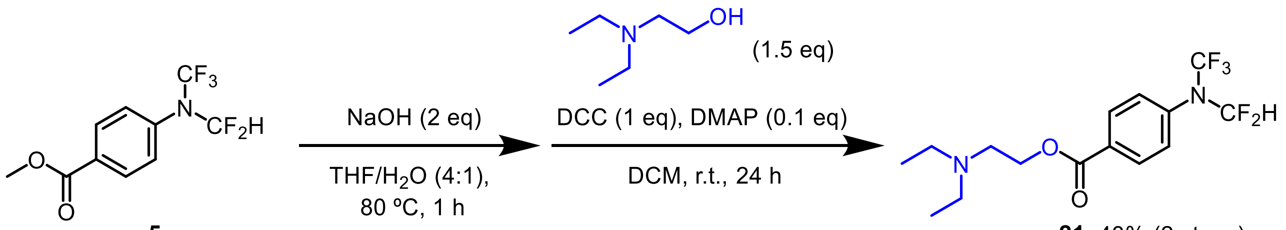

5 **21**, 43% (2 steps)

Step 1: A solution of NaOH (16 mg, 0.4 mmol, 2.1 equiv.) in 0.25 mL of water was added to a 4-mL vial containing a solution of methyl 4-((difluoromethyl)(trifluoromethyl)amino)benzoate (50.0 mg, 0.19 mmol, 1.0 equiv.) in 1 mL THF. The vial was sealed with a screw cap and heated under stirring at 80 °C for 1 h. The obtained mixture was then allowed to cool to room temperature. The crude was diluted with EtOAc (15 mL) and washed with HCl (0.1 M aqueous solution, 15 mL). The organic layer was separated, dried over $MgSO_4$, filtered and evaporated to dryness. The residue obtained was analyzed by NMR, showing full conversion of starting material, and was used without further purification for the next step.

^{1}H NMR (400 MHz, Methylene Chloride-d_2) δ 10.93 (s, 1H), 8.20 (d, *J* = 8.3 Hz, 2H), 7.53 (d, *J* = 8.3 Hz, 2H), 6.76 (t, *J* = 59.7 Hz, 1H). **^{19}F NMR** (376 MHz, Methylene Chloride-d_2) δ -56.37 (t, *J* = 5.8 Hz, 3F), -93.97 (dq, *J* = 59.8, 6.4 Hz, 2F).

Step 2: The residue obtained in step 1 was dissolved in 1 mL of DCM. Then, DMAP (2.5 mg, 0.02 mmol, 10 mol%), DCC (62 mg, 0.3 mmol, 1.6 equiv.) and 2-(diethylamino)ethan-1-ol (40 µL, 0.3 mmol, 1.6 equiv.) were added. The mixture was stirred for 24 h at ambient temperature and then solvent was evaporated. The crude was purified by column chromatography, eluting with 30% EtOAc/pentane. The title product was isolated as a colorless solid (27.5 mg, 0.08 mmol, 44% over two steps). Traces of impurities identified as DCC are present in the isolated product, quantified as 4.2% by ^{1}H NMR. The yield has been corrected accordingly.

R_f = 0.31 (30% EtOAc/*n*-pentane). **^{1}H NMR** (600 MHz, Methylene Chloride-d_2) δ 8.11 (m, 2H), 7.48 (d, *J* = 8.5 Hz, 2H), 6.73 (t, *J* = 59.8 Hz, 1H), 4.37 (t, *J* = 6.1 Hz, 2H), 2.82 (t, *J* = 6.1 Hz, 2H), 2.60 (q, *J* = 7.1 Hz, 4H), 1.04 (t, *J* = 7.1 Hz, 6H). **^{19}F NMR** (564 MHz, Methylene Chloride-d_2) δ -56.39 (t, *J* = 6.2 Hz, 3F), -93.95 (dq, *J* = 59.9, 6.3 Hz, 2F). **^{13}C{^{1}H} NMR** (151 MHz, Methylene Chloride-d_2) δ 165.7, 136.1, 132.2, 131.0, 130.3, 110.7 (t, *J* = 246.2 Hz), 64.3, 51.5, 48.1, 12.3. *Note*: Despite increased number of scans the signal of the CF_3 group could not be observed in ^{13}C NMR. **IR** (neat, cm^{-1}): 2971, 2931, 2851, 2813, 2324, 2092, 1933, 1723, 1614, 1575, 1511, 1434, 1380, 1323, 1273, 1197, 1120, 1024, 953, 862, 764, 733, 658. **HRMS** (APCI) calculated for $C_{15}H_{19}O_2N_2F_5$: 355.1430 $[M+H]^+$, found: 355.1434.

4. Starting Material Synthesis

Isothiocyanate,[1] *N*–trifluoromethylcarbamoyl fluoride,[1] and *N*–trifluoromethyl formamide,[2] starting materials were synthesized as reported previously. For all reactions that were performed with AgF, a reaction temperature of 50 °C was chosen to avoid any concerns regarding the AgF quality as discussed in reference [3].

4.1 Synthesis of *N*–trifluoromethyl (deutero)thioformamides

4.1.1 General Procedure 2 (GP2)

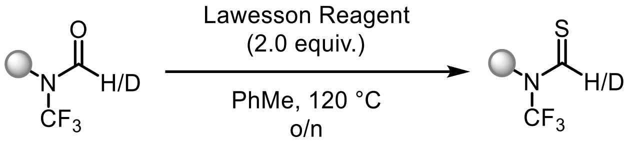

Under argon atmosphere a 20 mL vial was charged with the *N*–CF_3 formamide (usually on a 1.00 mmol scale) and 5 mL toluene were added [0.2 M]. Subsequently Lawesson's reagent (2.0 equiv.) was added to the solution and the vial was tightly closed with a screw cap. The reaction mixture was stirred overnight at 120 °C. Afterwards the solvent was evaporated, and the remaining crude material was purified via column chromatography to yield the corresponding *N*–CF_3 thioformamides.

Deuterated compounds (**S7**, **S12**) were obtained from the deuterated formamides following the general procedure.

4.1.2 Characterization Data of *N*–trifluoromethyl (deutero)thioformamides (S1-S17)

***N*-([1,1'-biphenyl]-4-yl)-*N*-(trifluoromethyl)methanethioamide (S1)**

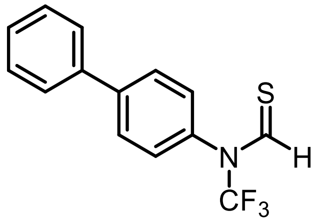

The reaction was performed on a 1.0 mmol scale according to GP2 and the title product was obtained after column chromatography (5% Et_2O/*n*-pentane) as a yellow solid (253 mg, 0.86 mmol, 86%).
R_f = 0.68 (5% Et_2O/*n*-pentane). **^{1}H NMR** (600 MHz, Chloroform-*d*) δ 10.21 (s, 1H), 7.73 (d, *J* = 8.4 Hz, 2H), 7.61 (d, *J* = 7.5 Hz, 2H), 7.47 (dd, *J* = 7.6, 7.6 Hz, 2H), 7.40 (t, *J* = 7.4 Hz, 1H), 7.34 (d, *J* = 8.3 Hz, 2H). **^{19}F NMR** (376 MHz, Chloroform-*d*) δ -57.82. **^{13}C{^{1}H} NMR** (151 MHz, Chloroform-*d*) δ 190.7, 143.3, 139.8, 129.4, 129.0, 128.7, 128.2, 127.4. *Note*: Despite an increased number of scans the signal of the CF_3 group could not be observed in ^{13}C NMR. **IR** (neat, cm^{-1}): 2924, 2097, 1722, 1485, 1349, 1218, 1141, 1007, 940, 730, 691. **HRMS** (APCI) calculated for $C_{14}H_{10}NF_3S$: 281.0481 [M]$^+$, found: 281.0486.

N-phenyl-N-(trifluoromethyl)methanethioamide (S2)

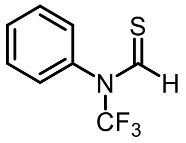

The reaction was performed on a 1.0 mmol scale according to GP2 and the title product was obtained after column chromatography (2% Et_2O/*n*-pentane) as a yellow oil (101.7 mg, 0.50 mmol, 50%).

R_f = 0.20 (2% Et_2O/*n*-pentane). **^{1}H NMR** (600 MHz, Methylene Chloride-*d*$_2$) δ 10.20 (s, 1H), 7.58 – 7.48 (m, 3H), 7.31 – 7.27 (m, 2H). **^{19}F NMR** (376 MHz, Methylene Chloride-*d*$_2$) δ -58.28. **^{13}C{^{1}H} NMR** (151 MHz, Methylene Chloride-*d*$_2$) δ 191.3, 134.9, 130.6, 130.3, 129.5, 120.5 (q, *J* = 264.7 Hz). **IR** (neat, cm^{-1}): 3064, 2102, 1731, 1594, 1493, 1415, 1343, 1216, 1147, 1073, 1017, 943, 893, 772, 695. **HRMS** (APCI) calculated for $C_8H_6NF_3S$: 205.0168 [M]$^+$, found: 205.0168.

(4-cyclohexylphenyl)-N-(trifluoromethyl)methanethioamide (S3)

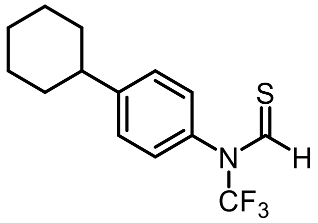

The reaction was performed on a 0.9 mmol scale according to GP2 and the title product was obtained after column chromatography (5% Et_2O/*n*-pentane) as a yellow oil (167 mg, 0.58 mmol, 64%).

R_f = 0.79 (5% Et_2O/*n*-pentane). **^{1}H NMR** (600 MHz, Methylene Chloride-*d*$_2$) δ 10.19 (s, 1H), 7.37 (d, *J* = 8.3 Hz, 2H), 7.18 (d, *J* = 8.1 Hz, 2H), 2.68 – 2.50 (m, 1H), 1.89 (dd, *J* = 27.4, 9.7 Hz, 4H), 1.76 (d, *J* = 12.9 Hz, 1H), 1.44 (h, *J* = 12.8 Hz, 4H), 1.29 (ddq, *J* = 17.6, 8.6, 4.8, 4.1 Hz, 1H). **^{19}F NMR** (376 MHz, Methylene Chloride-*d*$_2$) δ -58.44 (s, 3F). **^{13}C{^{1}H} NMR** (151 MHz, Methylene Chloride-*d*$_2$) δ 191.5, 151.1, 132.6, 129.4, 128.8, 120.7 (q, *J* = 264.6 Hz), 44.8, 34.8, 27.4, 26.6. **IR** (neat, cm^{-1}): 2926, 2853, 2332, 2092, 1904, 1729, 1510, 1415, 1344, 1218, 1148, 1013, 944, 682. **HRMS** (APCI) calculated for $C_{14}H_{16}ONF_3S$: 287.0950 [M]$^+$, found: 286.0872.

N-(trifluoromethyl)-N-(3,4,5-trimethoxyphenyl)methanethioamide (S4)

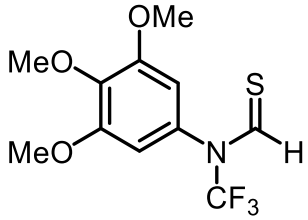

The reaction was performed on a 1.0 mmol scale according to GP2 and the title product was obtained after column chromatography (20% EtOAc/*n*-pentane) as a yellow solid (265.2 mg, 0.90 mmol, 90 %).

R_f = 0.71 (20% EtOAc/*n*-pentane). **^{1}H NMR** (600 MHz, Chloroform-*d*) δ 10.13 (s, 1H), 6.45 (s, 2H), 3.89 (s, 3H), 3.84 (s, 6H). **^{19}F NMR** (564 MHz, Chloroform-*d*) δ -57.93 (s, 3F). **^{13}C{^{1}H} NMR** (151 MHz, Chloroform-*d*) δ 190.6, 154.1, 139.4, 129.7, 120.1 (q, *J* = 265.3 Hz), 106.3, 61.1, 56.4. **IR** (neat, cm^{-1}): 3007, 2939, 2837, 2115, 1805, 1593, 1502, 1456, 1417, 1371, 1302, 1222, 1145, 1118, 999, 962, 902, 872, 827, 773, 716, 657. **HRMS** (ESI) calculated for $C_{11}H_{12}O_3NF_3S$: 318.0382 [M+Na]$^+$, found: 318.0379.

N-(4-bromo-2-chlorophenyl)-N-(trifluoromethyl)methanethioamide (S5)

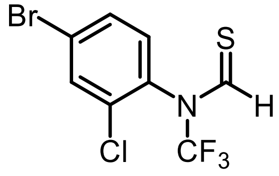

The reaction was performed on a 0.89 mmol scale according to GP2 and the title product was obtained after column chromatography (2% EtOAc/*n*-pentane) as a yellow solid (268 mg, 0.84 mmol, 94%).

R_f = 0.46 (2% EtOAc/*n*-pentane). **^{1}H NMR** (600 MHz, Chloroform-*d*) δ 10.06 (s, 1H), 7.75 (d, *J* = 2.2 Hz, 1H), 7.56 (dd, *J* = 8.5, 2.2 Hz, 1H), 7.17 (d, *J* = 8.5 Hz, 1H).

^{19}**F NMR** (564 MHz, Chloroform-*d*) δ -57.58 (s, 3F). ^{13}**C{^{1}H} NMR** (151 MHz, Chloroform-*d*) δ 189.2, 135.5, 133.9, 131.9, 131.8, 131.4, 125.2, 119.9 (q, *J* = 266.4 Hz). **IR** (neat, cm^{-1}): 3077, 2999, 2100, 1731, 1575, 1474, 1421, 1351, 1151, 1086, 1066, 1015, 943, 910, 875, 822, 796, 734, 667. **HRMS** (APCI) calculated for $C_8H_4NBrClF_3S$: 281.9194 [M-Cl]$^+$, found: 281.9192.

methyl 4-(*N*-(trifluoromethyl)methanethioamido)benzoate (S6)

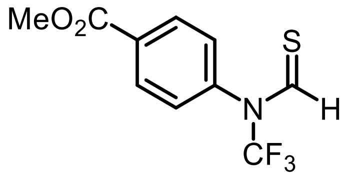

The reaction was performed on a 1.0 mmol scale according to GP2 and the title product was obtained after column chromatography (2% EtOAc/*n*-pentane) as a yellow liquid (189 mg, 0.72 mmol, 72%).

R$_f$ = 0.14 (2% EtOAc/*n*-pentane). ^{1}**H NMR** (600 MHz, Chloroform-*d*) δ 10.16 (s, 1H), 8.19 (m, 2H), 7.35 (d, *J* = 8.5 Hz, 2H), 3.95 (s, 3H). ^{19}**F NMR** (564 MHz, Chloroform-*d*) δ -57.48 (s, 3F). ^{13}**C{^{1}H} NMR** (151 MHz, Chloroform-*d*) δ 190.4, 165.9, 138.4, 132.0, 131.3, 129.4, 120.0 (q, *J* = 265.9 Hz), 52.6. **IR** (neat, cm^{-1}): 3005, 2954, 2846, 2326, 2109, 1926, 1726, 1607, 1508, 1416, 1345, 1277, 1223, 1152, 1108, 1015, 943, 891, 859, 829, 775, 712. **HRMS** (ESI) calculated for $C_{10}H_8NO_2F_3S$: 286.0120 [M+Na]$^+$, found: 286.0125.

***N*-([1,1'-biphenyl]-4-yl)-*N*-(trifluoromethyl)methanethioamide-*d* (S7)**

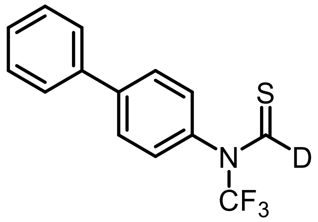

The reaction was performed on a 0.2 mmol scale according to GP2 and the title product was obtained after column chromatography (5% Et_2O/*n*-pentane) as a yellow solid (33.5 mg, 0.118 mmol, 59%, 92% deuteration).

R$_f$ = 0.68 (5% Et_2O/*n*-pentane). ^{1}**H NMR** (600 MHz, Methylene Chloride-*d$_2$*) δ 10.24 (s, 0.08H), 7.76 (d, *J* = 8.4 Hz, 2H), 7.65 (d, *J* = 7.4 Hz, 2H), 7.49 (dd, *J* = 7.7, 7.7 Hz, 2H), 7.42 (t, *J* = 7.4 Hz, 1H), 7.37 (d, *J* = 8.3 Hz, 2H). ^{19}**F NMR** (564 MHz, Methylene Chloride-*d$_2$*) δ -58.29 (s, 3F). ^{13}**C{^{1}H} NMR** (151 MHz, Methylene Chloride-*d$_2$*) δ 191.2 (dd, *J* = 55.1, 30.1 Hz), 143.7, 140.1, 134.1, 130.0, 129.5, 129.1, 128.6, 127.8, 120.6 (q, *J* = 265.1 Hz). **IR** (neat, cm^{-1}): 2926, 1730, 1484, 1347, 1297, 1215, 1140, 940, 894, 730, 690. **HRMS** (APCI) calculated for $C_{14}H_9{}^2HNF_3S$: 283.0622 [M+H]$^+$, found: 283.0621.

***tert*-butyl 4-(*N*-(trifluoromethyl)methanethioamido)piperidine-1-carboxylate (S8)**

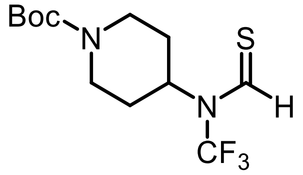

The reaction was performed on a 1.0 mmol scale according to GP2 and the title product was obtained after column chromatography (10% Et_2O/*n*-pentane) as a yellow oil (79.4 mg, 0.250 mmol, 25%).

R$_f$ = 0.59 (10% Et_2O/*n*-pentane). ^{1}**H NMR** (600 MHz, Methylene Chloride-*d$_2$*) δ 9.87 (s, 1H), 5.28 (brs, 1H), 4.22 (brs, 2H), 2.76 (brs, 2H), 1.89 (brs, 4H), 1.44 (s, 9H). ^{19}**F NMR** (376 MHz, Methylene Chloride-*d$_2$*) δ -55.55 (s, 3F). ^{13}**C{^{1}H} NMR** (151 MHz, Methylene Chloride-*d$_2$*) δ 190.2 (q, *J* = 4.2 Hz), 154.8, 121.9 (q, *J* = 266.8 Hz), 80.1, 56.0, 44.2, 29.4, 28.6. **IR** (neat, cm^{-1}): 2974, 2084, 1694, 1425, 1355, 1317, 1133, 1012, 956, 885, 767, 680. **HRMS** (APCI) calculated for $C_{12}H_{19}O_2N_2F_3S$: 239.0466 [M-C_4H_9O]$^+$, found: 239.0461.

tert-butyl _N_-thioformyl-_N_-(trifluoromethyl)-_L_-phenylalaninate (_S_9)

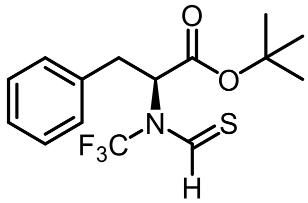

The reaction was performed on a 0.75 mmol scale according to GP2 and the title product was obtained after column chromatography (10% Et_2O/*n*-pentane) as a yellow oil (139 mg, 0.418 mmol, 56%).

***R**$_f$* = 0.54 (10% Et_2O/*n*-pentane). **^{1}H NMR** (600 MHz, Toluene-d_8, 90°C) δ 9.54 – 9.37 (m, 1H), 7.17 – 6.82 (m, 5H, overlap with toluene), 5.72 (brs, 1H), 3.44 (dd, *J* = 14.8, 6.0 Hz, 1H), 3.14 (dd, *J* = 14.9, 9.0 Hz, 1H), 1.26 (s, 9H). **^{19}F NMR** (565 MHz, Toluene-d_8, 90°C) δ -56.18. **^{13}C{^{1}H} NMR** (151 MHz, Methylene Chloride-d_2) δ 190.2, 166.6, 137.2, 129.8, 128.9, 127.4, 121.6 (q), 83.7, 28.0. **IR** (neat, cm^{-1}): 2979, 2327, 2112, 1738, 1433, 1326, 1152, 970, 842, 697. **HRMS** (ESI) calculated for $C_{15}H_{18}O_2NF_3S$: 356.0903 $[M+Na]^+$, found: 356.0906.

benzyl _N_-thioformyl-_N_-(trifluoromethyl)-_L_-leucinate (_S_10)

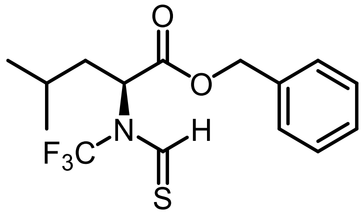

The reaction was performed on a 2 x 2.00 mmol scale according to GP2 and the title product was obtained after column chromatography (5% Et_2O/ *n*-pentane) as a yellow oil (1.1663 g, 3.49 mmol, 87%).

***R**$_f$* = 0.62 (5% Et_2O/ *n*-pentane). **^{1}H NMR** (600 MHz, Methylene Chloride-d_2) δ 9.89 (s, 1H), 7.42 – 7.26 (m, 5H), 5.94 (s, 1H), 5.17 (dd, *J* = 12.0, 6.6 Hz, 2H), 2.16 (ddd, *J* = 14.4, 8.8, 5.3 Hz, 1H), 1.88 (ddd, *J* = 14.4, 9.2, 5.0 Hz, 1H), 1.77 – 1.57 (m, 1H), 0.96 (d, *J* = 6.5 Hz, 3H), 0.96 (d, *J* = 6.7 Hz, 3H). **^{19}F NMR** (376 MHz, Methylene Chloride-d_2) δ -55.99 (s, 3F). **^{13}C{^{1}H} NMR** (151 MHz, Methylene Chloride-d_2) δ 190.6, 168.8, 135.8, 129.1, 129.0, 128.7, 121.7 (q, *J* = 266.3 Hz), 68.2, 56.4, 38.5, 25.6, 23.2, 22.5. **IR** (neat, cm^{-1}): 2961, 2899, 2324, 2089, 1746, 1432, 1363, 1325, 1265, 1197, 1154, 976, 894, 743, 695. **HRMS** (ESI) calculated for $C_{15}H_{18}O_2NF_3S$: 356.0903 $[M+Na]^+$, found: 356.0895.

_N_-(4-bromobenzyl)-_N_-(trifluoromethyl)methanethioamide (S11)

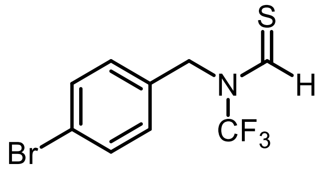

The reaction was performed on a 1.0 mmol scale according to GP2 and the title product was obtained after column chromatography (5% EtOAc/*n*-pentane) as a yellow oil (268 mg, 0.90 mmol, 90%).

***R**$_f$* = 0.65 (5% EtOAc/*n*-pentane). **^{1}H NMR** (600 MHz, Chloroform-*d*) δ 9.96 (s, 1H), 7.46 (d, *J* = 8.5 Hz, 2H), 7.20 (d, *J* = 8.5 Hz, 2H), 5.16 (s, 2H). **^{19}F NMR** (564 MHz, Chloroform-*d*) δ -58.82 (s, 3F). **^{13}C{^{1}H} NMR** (151 MHz, Chloroform-*d*) δ 189.5 (q, *J* = 2.7 Hz), 133.3, 132.0, 129.5, 122.3, 121.1 (q, *J* = 266.0 Hz), 47.2. **IR** (neat, cm^{-1}): 2937, 2332, 2098, 2001, 1905, 1594, 1488, 1452, 1418, 1348, 1305, 1150, 1073, 988, 929, 895, 822, 785, 706, 666. **HRMS** (APCI) calculated for $C_9H_7NBrF_3S$: 296.9429 $[M]^+$, found: 296.9427.

***tert*-butyl 4-(*N*-(trifluoromethyl)methanethioamido-*d*)piperidine-1-carboxylate (S12)**

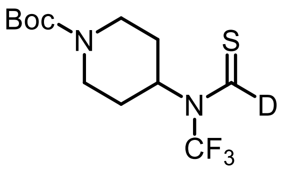

The reaction was performed on a 0.7 mmol scale according to GP2 except for using 1.00 equiv. of Lawesson's reagent. The title product was obtained after column chromatography (10% Et_2O/*n*-pentane) as a yellow oil (32.9 mg, 0.105 mmol, 15%, 90% deuteration).

R R_f = 0.62 (10% Et_2O/*n*-pentane). **^{1}H NMR** (600 MHz, Methylene Chloride-d_2) δ 9.87 (s, 0.10 H), 5.28 (brs, 1H), 4.23 (brs, 2H), 2.77 (brs, 2H), 1.89 (brs, 4H), 1.44 (s, 9H). **^{19}F NMR** (376 MHz, Methylene Chloride-d_2) δ -55.55 (s, 3F). **^{13}C{^{1}H} NMR** (151 MHz, Methylene Chloride-d_2) δ 190.4 – 189.4 (m), 154.9, 121.9 (q, *J* = 266.9 Hz), 80.1, 56.0, 44.3, 29.4, 28.6. **IR** (neat, cm^{-1}): 2974, 2932, 2863, 2324, 2110, 1913, 1694, 1597, 1410, 1362, 1321, 1275, 1242, 1130, 1023, 957, 901, 766, 739, 680. **HRMS** (ESI) calculated for $C_{12}H_{18}{}^2HO_2N_2F_3S$: 336.1074 [M+Na]$^+$, found: 336.1073.

***N*-(9-ethyl-9*H*-carbazol-3-yl)-*N*-(trifluoromethyl)methanethioamide (S13)**

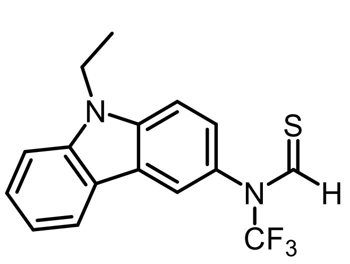

The reaction was performed on a 0.78 mmol scale according to GP2 and the title product was obtained after column chromatography (2% EtOAc/*n*-pentane) as a yellow solid (205 mg, 0.63 mmol, 81%).

R_f = 0.39 (2% EtOAc/*n*-pentane). **^{1}H NMR** (600 MHz, Chloroform-*d*) δ 10.32 (s, 1H), 8.09 (d, *J* = 7.7 Hz, 1H), 8.00 (d, *J* = 2.1 Hz, 1H), 7.55 – 7.50 (m, 2H), 7.44 (d, *J* = 8.2 Hz, 1H), 7.33 (dd, *J* = 8.6, 2.1 Hz, 1H), 7.28 (ddd, *J* = 8.0, 7.0, 1.0 Hz, 1H), 4.38 (q, *J* = 7.3 Hz, 2H), 1.48 (t, *J* = 7.3 Hz, 3H). **^{19}F NMR** (564 MHz, Chloroform-*d*) δ -58.04 (s, 3F). **^{13}C{^{1}H} NMR** (151 MHz, Chloroform-*d*) δ 191.5, 140.7, 140.2, 126.7, 125.9, 125.4, 123.8, 122.5, 121.3, 120.9, 120.5 (q, *J* = 264.6 Hz), 119.7, 109.5, 109.0, 37.9, 14.0. **IR** (neat, cm^{-1}): 3009, 2976, 2117, 1849, 1780, 1698, 1627, 1598, 1470, 1425, 1331, 1222, 1141, 1019, 943, 889, 798, 747, 715, 672. **HRMS** (ESI) calculated for $C_{16}H_{13}N_2F_3S$: 345.0644 [M+Na]$^+$, found: 345.0641.

methyl 4-(*N*-(trifluoromethyl)methanethioamido)thiophene-2-carboxylate (S14)

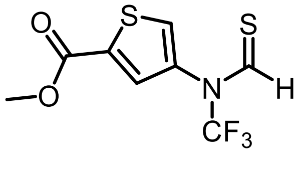

The reaction was performed on a 1.0 mmol scale according to GP2 and the title product was obtained after column chromatography (10% Et_2O/*n*-pentane) as a yellow oil (190 mg, 0.710 mmol, 71%).

R_f = 0.70 (10% Et_2O/*n*-pentane). **^{1}H NMR** (600 MHz, Methylene Chloride-d_2) δ 10.13 (s, 1H), 7.69 (s, 1H), 7.64 (s, 1H), 3.89 (s, 3H). **^{19}F NMR** (376 MHz, Methylene Chloride-d_2) δ -59.11 (s, 3F). **^{13}C{^{1}H} NMR** (151 MHz, Methylene Chloride-d_2) δ 190.4, 161.4, 134.2, 132.0, 131.6, 131.0, 119.6 (q, *J* = 265.6 Hz), 52.4. **IR** (neat, cm^{-1}): 3102, 2954, 2105, 1717, 1448, 1242, 1151, 1076, 973, 888, 783, 723. **HRMS** (APCI) calculated for $C_8H_6O_2NF_3S_2$: 268.9787 [M]$^+$, found: 268.9787.

N-(*trans*-2-phenylcyclopropyl)-N-(trifluoromethyl)methanethioamide (S15)

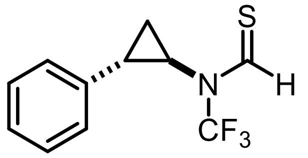

The reaction was performed on a 1.00 mmol scale according to GP2 and the title product was obtained after column chromatography (5% Et_2O/Pent) as a yellow oil (166 mg, 0.67 mmol, 67%).

***R**$_f$* = 0.77 (5% Et_2O/Pent). **^{1}H NMR** (600 MHz, Methylene Chloride-d_2) δ 10.01 (s, 1H), 7.39 – 7.33 (m, 3H), 7.32 – 7.26 (m, 1H), 7.24 (d, J = 7.7 Hz, 3H), 2.99 (s, 1H), 2.60 – 2.38 (m, 2H), 1.67 (dt, J = 10.4, 5.7 Hz, 1H), 1.63 (q, J = 7.5, 7.1 Hz, 1H). **^{19}F NMR** (376 MHz, Methylene Chloride-d_2) δ -57.65 (s, 3F). **^{13}C{^{1}H} NMR** (151 MHz, Methylene Chloride-d_2) δ 191.5, 139.5, 128.9, 127.3, 127.1, 121.5 (q, J = 266.0 Hz), 37.3, 25.9, 16.6. **IR** (neat, cm^{-1}): 3030, 2668, 2327, 2120, 1997, 1943, 1724, 1604, 1499, 1424, 1356, 1318, 1238, 1152, 1012, 975, 892, 776, 743, 692. **HRMS** (APCI) calculated for $C_{11}H_{10}NF_3S$: 245.0481 [M]$^+$, found: 245.0482.

N-(1-(2,6-dimethylphenoxy)propan-2-yl)-N-(trifluoromethyl)methanethioamide (S16)

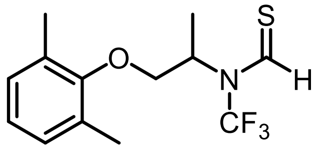

The reaction was performed on a 0.2 mmol scale according to GP2 and the title product was obtained after column chromatography (5% Et_2O/n-pentane) as a yellow oil (34.5 mg, 0.118 mmol, 59%).

***R**$_f$* = 0.65 (5% Et_2O/n-pentane). ^{1}**H NMR** (600 MHz, Methylene Chloride-d_2) δ 9.96 (s, 1H), 7.00 (d, J = 7.5 Hz, 2H), 6.92 (dd, J = 8.0, 6.9 Hz, 1H), 5.79 (brs, 1H), 4.10 (brs, 1H), 3.87 (t, J = 8.2 Hz, 1H), 2.25 (s, 6H), 1.60 (d, J = 7.2 Hz, 3H). **^{19}F NMR** (376 MHz, Methylene Chloride-d_2) δ -55.34 (s, 3F). **^{13}C{^{1}H} NMR** (151 MHz, Methylene Chloride-d_2) δ 191.0, 155.6, 131.3, 129.4, 124.7, 122.1 (q, J = 266.4 Hz), 72.2, 53.2 (br), 16.5, 15.0. **IR** (neat, cm^{-1}): 2928, 1432, 1326, 1201, 1215, 1150, 1097, 1021, 948, 896, 768, 658. **HRMS** (ESI) calculated for $C_{13}H_{16}ONF_3S$: 314.0797 [M+Na]$^+$, found: 314.0795.

(R)-N-(1-(3-(2-cyanobenzyl)-1-methyl-2-oxo-6-thioxo-1,2,3,6-tetrahydropyrimidin-4-yl)piperidin-3-yl)-N-(trifluoromethyl)methanethioamide (S17)

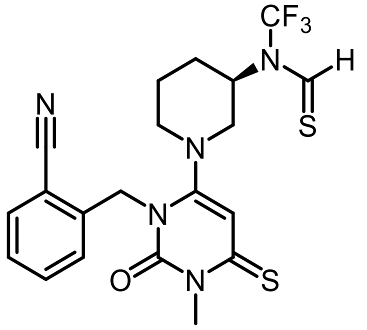

The reaction was performed on a 0.2 mmol scale according to GP2 and the title product was obtained as a yellow solid (83.4 mg, 0.184 mmol, 56 %).

***R**$_f$* = 0.49 (35% EtOAc/ n-pentane). **^{1}H NMR** (600 MHz, Methylene Chloride-d_2) δ 9.82 (s, 1H), 7.70 (d, J = 7.8 Hz, 1H), 7.57 (dd, J = 7.7, 7.7 Hz, 1H), 7.41 (dd, J = 7.6, 7.6 Hz, 1H), 7.19 (d, J = 7.9 Hz, 1H), 6.42 (s, 1H), 5.34 (d, J = 11.7 Hz, 1H), 5.26 (s, 1H), 5.20 (d, J = 15.8 Hz, 1H), 3.67 (s, 3H), 3.29 (d, J = 11.2 Hz, 1H), 3.21 (d, J = 12.9 Hz, 1H), 2.93 (t, J = 11.0 Hz, 1H), 2.72 (t, J = 12.3 Hz, 1H), 2.12 (d, J = 12.5 Hz, 1H), 2.02 – 1.93 (m, 1H), 1.92 (s, 1H), 1.83 – 1.74 (m, 1H). **^{19}F NMR** (565 MHz, Methylene Chloride-d_2) δ -55.25 (s, 3F). **^{13}C{^{1}H} NMR** (151 MHz, Methylene Chloride-d_2) δ 189.7 (q, J = 4.0 Hz), 189.4, 152.8, 150.7, 139.8, 133.3, 133.2, 128.5, 127.4, 121.1 (q, J = 266.9 Hz), 117.1, 111.3, 104.6, 53.4, 52.5, 51.4, 47.0, 35.0, 26.5, 24.5. **IR** (neat, cm^{-1}): 2951, 2855, 2226, 1689, 1529, 1434, 1351, 1319,

1201, 1146, 1076, 1000, 956, 934, 870, 813, 761, 706. **HRMS** (APCI) calculated for $C_{20}H_{20}N_5F_3OS_2$: 467.1061 $[M]^+$, found: 467.1048.

4.2 Synthesis of isothiocyanates

4.2.1 General Procedure 3 (GP3)

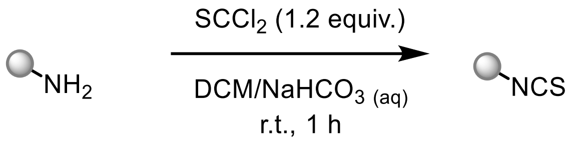

A 100 mL round-bottom flask was charged with the amine or ammonium salt (5 mmol, 1 equiv.), DCM (25 mL) and saturated aqueous $NaHCO_3$ (25 mL). To the biphasic system under strong stirring was slowly added thiophosgene (460 µL, 6 mmol, 1.2 equiv.) at room temperature. After 1 h, the two phases were separated, and the aqueous phase was extracted with DCM (2x). The combined organic phases were dried over $MgSO_4$ and concentrated under reduced pressure. This material was then directly used in the next step without further purification.

4.2.2 Characterization Data of isothiocyanates

Methyl 4-isothiocyanatothiophene-2-carboxylate (S18)

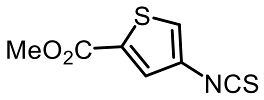

The reaction was performed on a 5.0 mmol scale according to GP3 using methyl 4-aminothiophene-2-carboxylate as substrate, and the title product was obtained as a yellow solid (0.99 g, 5.0 mmol, quant.).

^{1}H NMR (400 MHz, Chloroform-*d*) δ 7.61 (s, 1H), 7.33 (s, 1H), 3.89 (s, 3H). **^{13}C{^{1}H} NMR** (101 MHz, Chloroform-*d*) δ 161.5, 137.0, 133.7, 130.2, 128.2, 125.9, 52.7. **IR** (neat, cm^{-1}): 3094, 2118, 1708, 1439, 1249, 1191, 1070, 865, 784, 738. **HRMS** (ESI): calculated for $C_7H_5NO_2S_2$: 198.9756 [M]$^+$, found 198.9756.

***(trans*-2-isothiocyanatocyclopropyl)benzene (S19)**

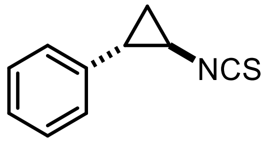

The reaction was performed on a 10.0 mmol scale according to GP3 using trans-2-Phenylcyclopropanamine hydrochloride as substrate, and the title product was obtained as a yellow oil (1.75 g, 10.0 mmol, quant.).

^{1}H NMR (400 MHz, Methylene Chloride-*d*$_2$) δ 7.32 (dd, *J* = 7.3, 7.3 Hz, 2H), 7.24 (t, *J* = 7.3 Hz, 1H), 7.08 (d, *J* = 7.3 Hz, 2H), 3.01 (dt, *J* = 7.5, 3.7 Hz, 1H), 2.47 (ddd, *J* = 10.0, 7.0, 3.2 Hz, 1H), 1.53 (ddd, *J* = 10.2, 6.1, 4.3 Hz, 1H), 1.38 (q, *J* = 7.0 Hz, 1H). **^{13}C{^{1}H} NMR** (151 MHz, Methylene Chloride-*d*$_2$) δ 138.4, 128.9, 128.5, 127.2, 126.5, 34.3, 26.3, 17.3. **IR** (neat, cm^{-1}): 3031, 2096, 1602, 1497, 1455, 1386, 1321, 1191, 1039, 974, 930, 870, 750, 695. **HRMS** (APCI) calculated for $C_{10}H_9NS$: 175.0450 [M]$^+$, found: 175.0450.

(*R*)-2-((6-(3-isothiocyanatopiperidin-1-yl)-3-methyl-2,4-dioxo-3,4-dihydropyrimidin-1(2*H*)-yl)methyl)benzonitrile (*S*20)

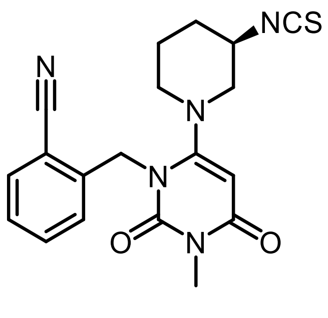

The reaction was performed on a 10.0 mmol scale according to GP3 and the title product was obtained as a colorless solid (3.91 g, 10.0 mmol, quant.).

^{1}H NMR (600 MHz, Chloroform-*d*) δ 7.66 (d, *J* = 7.7 Hz, 1H), 7.54 (dd, *J* = 7.7, 7.7 Hz, 1H), 7.37 (dd, *J* = 7.6, 7.6 Hz, 1H), 7.15 (d, *J* = 7.9 Hz, 1H), 5.37 (s, 1H), 5.27 (s, 2H), 3.84 (s, 1H), 3.27 (s, 3H), 3.11 – 2.86 (s, 3H), 2.80 (s, 1H), 2.04 – 1.74 (m, 3H), 1.67 (s, 1H). **^{13}C{^{1}H} NMR** (151 MHz, Chloroform-*d*) δ 162.9, 159.1, 152.4, 140.4, 135.0, 133.3, 133.3, 128.1, 127.0, 117.2, 111.0, 91.2, 55.5, 52.7, 52.1, 46.3, 30.7, 28.0, 21.7. **IR** (neat, cm^{-1}): 3093, 3053, 2974, 2914, 2850, 2217, 2098, 1672, 1607, 1426, 1372, 1263, 1228, 1097, 1025, 950, 868, 813, 762. **HRMS** (ESI) calculated for $C_{19}H_{19}N_5S$: 404.1152 [M+Na]$^+$, found: 404.1145.

4.3 Synthesis of *N*–trifluoromethyl carbamoyl fluorides

4.3.1 General Procedure 4 (GP4)

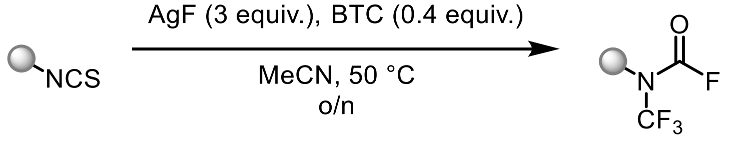

A 20 mL vial was charged with the isothiocyanate (2 mmol, 1 equiv.), AgF (10 mmol, 5 equiv.) and bis(trichloromethyl)carbonate (BTC) (237 mg, 0.8 mmol, 0.4 equiv.). Acetonitrile (10 mL) was quickly added and the vial was sealed (if the isothiocyanate was a liquid or an oil it was added as a solution in MeCN). The mixture was stirred at 50 °C for 15 h. After the indicated time, the crude mixture was added at once to Et_2O (40 mL) and stirred for 10 minutes. The formed solid was removed by filtration over celite and the solvents were then evaporated. The crude material was redissolved in Et_2O and filtered through a pad of silica to remove the last traces of salt byproducts. The *N*–trifluoromethylcarbamoyl fluorides were then obtained in a technical grade purity ranging from 90 to 99% and used without further purification. All previously synthesized *N*–trifluoromethylcarbamoyl fluorides matched the reported data.[1-2]

4.3.2 Characterization Data of *N*-trifluoromethyl carbamoyl fluorides

(4-cyclohexylphenyl)(trifluoromethyl)carbamoyl fluoride (S21)

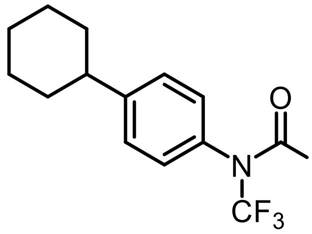

The reaction was performed on a 2.0 mmol scale according to GP4 and the title product was obtained as a yellow oil (567.0 mg, 1.96 mmol, 98%). **^{1}H NMR** (400 MHz, Chloroform-d) δ 7.31 (d, *J* = 7.4 Hz, 2H), 7.22 (d, *J* = 7.8 Hz, 2H), 2.55 (m, 1H), 1.93 – 1.82 (m, 4H), 1.77 (d, *J* = 12.9 Hz, 1H), 1.49 – 1.33 (m, 4H), 1.32 – 1.19 (m, 1H). **^{19}F NMR** (376 MHz, Chloroform-*d*) δ -2.50 (brs, 1F), -56.44 (s, 3F). **^{13}C{^{1}H} NMR** (101 MHz, Chloroform-*d*) δ 150.8, 142.4 (d, *J* = 299.9 Hz), 130.8, 128.4, 128.2, 119.5 (q, *J* = 264.8 Hz), 44.4, 34.4, 26.8, 26.1. **IR** (neat, cm^{-1}): 2928, 1835, 1363, 1325, 1271, 1167, 1000, 963, 755, 687. **HRMS** (ESI): calculated for $C_{14}H_{15}F_4ON$: 312.0987 $[M+Na]^+$, found 312.0982.

benzyl *N*-(fluorocarbonyl)-*N*-(trifluoromethyl)-*L*-leucinate (S22)

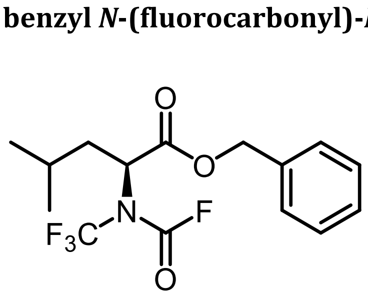

The reaction was performed on a 5 x 2.0 mmol scale according to GP4 and the title product was obtained as a colorless oil (2.24 g, 6.68 mmol, 67%).

^{1}H NMR (600 MHz, Chloroform-*d*) δ 7.41 – 7.32 (m, 5H), 5.22 (s, 2H), 4.71 (s, 1H), 2.05 – 1.91 (m, 2H), 1.72 – 1.64 (m, 1H), 0.99 (d, *J* = 6.7 Hz, 3H), 0.96 (d, *J* = 6.5 Hz, 3H). **^{19}F NMR** (376 MHz, Chloroform-*d*) δ -6.06 (brs, 1F), -54.31 (s, 3F). **^{13}C{^{1}H} NMR** (151 MHz, Chloroform-*d*) δ 168.5, 134.8, 128.8, 128.8, 128.5, 119.9 (d, *J* = 266.8 Hz), 68.3, 58.2, 38.3, 25.0, 23.1, 21.3. **IR** (neat, cm^{-1}): 3036, 2962, 2877, 2326, 2111, 1830, 1749, 1460, 1399, 1343, 1261,

1177, 1023, 968, 892, 748, 697. **HRMS** (ESI) calculated for $C_{15}H_{17}O_3NF_4$: 244.0597 [M-Bn]$^+$, found: 244.0590.

Methyl 4-((fluorocarbonyl)(trifluoromethyl)amino)thiophene-2-carboxylate (S23)

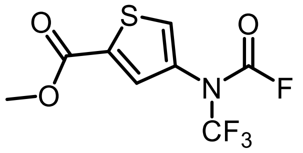

The reaction was performed on a 2.0 mmol scale according to GP4 and the title product was obtained as a pale yellow solid (531.5 mg, 1.96 mmol, 98%). **^{1}H NMR** (400 MHz, Chloroform-*d*) δ 7.67 (s, 1H), 7.62 (s, 1H), 3.85 (s, 3H). **^{19}F NMR** (376 MHz, Chloroform-*d*) δ -3.58 (brs, 1F), -57.41 (s, 3F). **^{13}C{^{1}H} NMR** (101 MHz, Chloroform-*d*) δ 161.4, 141.4 (d, *J* = 301.1 Hz), 134.5, 131.3, 129.7, 118.9 (q, *J* = 265.1 Hz), 52.6. **IR** (neat, cm^{-1}):1827, 1703, 1447, 1329, 1244, 1168, 1078, 989, 954, 745, 705. **HRMS** (ESI): calculated for $C_8H_5F_4NO_3S$: 270.9919 [M+H]$^+$, found 270.9921.

***trans*-2-phenylcyclopropyl)(trifluoromethyl)carbamoyl fluoride (S24)**

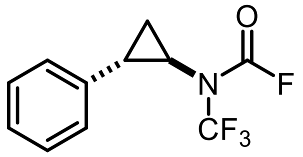

The reaction was performed on a 2.0 mmol scale according to GP4 and the title product was obtained as a pale yellow oil (1.54 g, 6.23 mmol, quant., technical grade purity).

^{1}H NMR (600 MHz, Chloroform-*d*) δ 7.27 – 7.20 (m, 2H), 7.19 – 7.15 (m, 1H), 7.09 (d, *J* = 7.6 Hz, 2H), 2.73 (s, 1H), 2.36 (t, *J* = 8.0 Hz, 1H), 1.49 (q, *J* = 7.1 Hz, 1H), 1.43 (dt, *J* = 16.0, 7.6 Hz, 1H). **^{19}F NMR** (565 MHz, Chloroform-*d*) δ -2.45 (brs, 1F), -55.55 (s, 3F). **^{13}C{^{1}H} NMR** (151 MHz, Chloroform-*d*) δ 143.0 (d, *J* = 303.8 Hz), 137.9, 128.8, 127.2, 126.8, 120.3 (q, *J* = 265.5 Hz), 35.7, 25.9, 16.1. **IR** (neat, cm^{-1}): 3034, 2281, 2110, 1832, 1502, 1372, 1317, 1274, 1169, 1034, 992, 753, 693. **HRMS** (APCI) calculated for $C_{11}H_9ONF_4$: 247.0615 [M]$^+$, found: 247.0616.

(*R*)-(1-(3-(2-cyanobenzyl)-1-methyl-2,6-dioxo-1,2,3,6-tetrahydropyrimidin-4-yl)piperidin-3-yl)(trifluoromethyl)carbamoyl fluoride (S25)

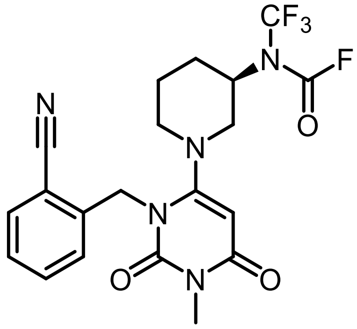

The reaction was performed on a 2.0 mmol scale according to GP4 and the title product was obtained as a pale yellow solid (225 mg, 0.50 mmol, 25%). **^{1}H NMR** (600 MHz, Chloroform-*d*) δ 7.70 (d, *J* = 6.8 Hz, 1H), 7.58 (dd, *J* = 7.2, 7.2 Hz, 1H), 7.40 (dd, *J* = 7.3, 7.3 Hz, 1H), 7.19 (dd, *J* = 18.8, 7.9 Hz, 1H), 5.42 (s, 1H), 5.29 (d, *J* = 16.0 Hz, 1H), 5.20 (d, *J* = 16.0 Hz, 1H), 3.95 (ddd, *J* = 16.0, 9.8, 4.4 Hz, 1H), 3.26 (s, 3H), 3.16 – 3.06 (m, 3H), 2.56 (t, *J* = 11.4 Hz, 1H), 2.13 – 2.04 (m, 1H), 1.89 – 1.84 (m, 1H), 1.78 – 1.68 (m, 1H), 1.36 – 1.25 (m, 1H). **^{19}F NMR** (376 MHz, Chloroform-*d*) δ -1.01 (brs, 1F), -54.55 (s, 3F). **^{13}C NMR** (151 MHz, Chloroform-*d*) δ 163.4, 159.5, 153.0, 141.1, 133.8 (d, *J* = 9.7 Hz), 130.5, 129.0, 128.6, 127.5, 120.4 (q, *J* = 265.8 Hz), 117.7, 111.4, 91.9, 55.9, 53.4, 52.2, 46.8, 28.3, 27.6, 25.0. **IR** (neat, cm^{-1}): 2955, 2226, 1825, 1703, 1650, 1439, 1328, 1219, 1166, 1101, 1016, 984, 949, 760, 698. **HRMS** (APCI) calculated for $C_{20}H_{19}O_3N_5F_4$: 453.1419 [M]$^+$, found: 453.1417.

4.4 Synthesis of *N*–trifluoromethyl (deutero)formamides

4.4.1 General Procedure 5 (GP5)

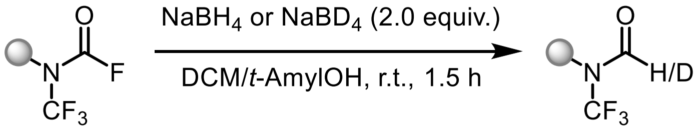

A solution of *N*–CF_3 carbamoyl fluoride (1 equiv.) in DCM was added to a mixture of $NaBH_4$ or $NaBD_4$ (2 equiv.) in ^{t}AmOH (DCM/^{t}AmOH = 1:1). The reaction mixture (0.2 M) was then stirred at room temperature (0.5 h – 2 h) and monitored by TLC until full consumption of starting material. The reaction mixture was then quenched by slow addition of saturated NH_4Cl (aq.) and stirred for an additional 10 min. The organic phase was separated, and the aqueous phase was extracted with DCM (2 x 15 mL). The combined organic phases were dried over $MgSO_4$, concentrated in vacuo and the crude product purified by flash column chromatography.

4.4.2 Characterization Data of *N*-trifluoromethyl (deutero)formamides

N-phenyl-N-(trifluoromethyl)formamide (S26)

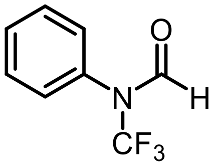

The reaction was performed on a 2.0 mmol scale according to GP5 and the title product was obtained after column chromatography (2.5% EtOAc/*n*-pentane) as a pale yellow oil (210 mg, 1.11 mmol, 56%).

$\boldsymbol{R_f}$ = 0.20 (2.5% EtOAc/*n*-pentane). **^{1}H NMR** (600 MHz, Chloroform-*d*) δ 8.90 (s, 1H), 7.51 – 7.46 (m, 3H), 7.30 – 7.26 (m, 2H). **^{19}F NMR** (565 MHz, Chloroform-*d*) δ -56.31 (s, 3F). **^{13}C{^{1}H} NMR** (151 MHz, Chloroform-*d*) δ 159.1, 130.1, 129.9. 129.1. *Note*: Despite an increased number of scans the signal of the CF_3 group could not be observed in ^{13}C NMR. **IR** (neat, cm^{-1}):3067, 2938, 2252, 1810, 1723, 1594, 1494, 1455, 1340, 1263, 1192, 1144, 1076, 979, 805, 757, 725, 694. **HRMS** (ESI): calculated for $C_{14}H_{16}F_3ON$: 189.0396 [M+Na]$^+$, 189.0395.

N-(4-cyclohexylphenyl)-N-(trifluoromethyl)formamide (S27)

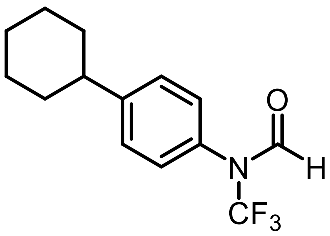

The reaction was performed on a 2.0 mmol scale according to GP5 and the title product was obtained after column chromatography (10% Et_2O/*n*-pentane) as a yellow solid (412 mg, 1.52 mmol, 76%).

$\boldsymbol{R_f}$ = 0.42 (10% Et_2O/*n*-pentane). **^{1}H NMR** (600 MHz, Methylene Chloride-*d*$_2$) δ 8.87 (s, 1H), 7.34 (d, *J* = 8.0 Hz, 2H), 7.19 (d, *J* = 7.9 Hz, 2H), 2.59 (m, 1H), 1.95 – 1.82 (m, 4H), 1.77 (d, *J* = 13.2 Hz, 1H), 1.50 – 1.37 (m, 4H), 1.29 (m, 1H). **^{19}F NMR** (565 MHz, Methylene Chloride-*d*$_2$) δ -56.87 (s, 3F). **^{13}C{^{1}H} NMR** (151 MHz, Methylene Chloride-*d*$_2$) δ 159.4, 150.7, 129.6, 129.2, 128.5, 121.1 (q, *J* = 263.9 Hz), 44.7, 34.7, 27.2, 26.5. **IR** (neat, cm^{-1}):2926, 1715, 1344, 1252, 1202, 1135, 978, 728. **HRMS** (ESI): calculated for $C_{14}H_{16}F_3ON$: 294.1081 [M+Na]$^+$, 294.1076.

N-(4-bromo-2-chlorophenyl)-N-(trifluoromethyl)formamide (S28)

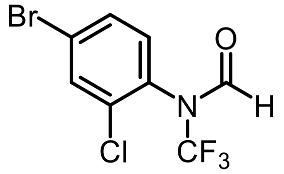

The reaction was performed on a 1.0 mmol scale according to GP5 and the title product was obtained after column chromatography (5% EtOAc/*n*-pentane) as a colorless oil (385 mg, 1.27 mmol, 64%).

R_f = 0.41 (5% EtOAc/*n*-pentane). **^{1}H NMR** (600 MHz, Chloroform-*d*) δ 8.83 (s, 1H), 7.73 (d, *J* = 2.2 Hz, 1H), 7.52 (dd, *J* = 8.5, 2.2 Hz, 1H), 7.19 (d, *J* = 8.5 Hz, 1H). **^{19}F NMR** (564 MHz, Chloroform-*d*) δ -56.24 (s, 3F). **^{13}C{^{1}H} NMR** (151 MHz, Chloroform-*d*) δ 157.9, 135.8, 133.7, 132.2, 131.5, 128.7, 125.1, 120.2 (q, *J* = 264.6 Hz). **IR** (neat, cm^{-1}): 2119, 1900, 1727, 1578, 1476, 1380, 1343, 1256, 1200, 1153, 1085, 1065, 979, 870, 803, 755, 720, 683. **HRMS** (APCI) calculated for $C_8H_4NBrClF_3S$: 265.9428 [M-Cl]$^+$, found: 265.9423.

methyl 4-(N-(trifluoromethyl)formamido)benzoate (S29)

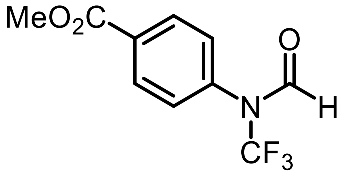

The reaction was performed on a 2.0 mmol scale according to GP5 and the title product was obtained after column chromatography (10% EtOAc/*n*-pentane) as a white solid (306 mg, 1.24 mmol, 62%).

R_f = 0.40 (10% EtOAc/*n*-pentane). **^{1}H NMR** (600 MHz, Chloroform-*d*) δ 8.85 (brs, 1H), 8.14 (m, 2H), 7.35 (d, *J* = 8.5 Hz, 2H), 3.93 (s, 3H). **^{19}F NMR** (564 MHz, Chloroform-*d*) δ -55.93 (s, 3F). **^{13}C{^{1}H} NMR** (151 MHz, Chloroform-*d*) δ 165.9, 158.7, 131.7, 131.1, 129.0, 123.0, 120.4 (q, *J* = 265.4 Hz), 52.6. **IR** (neat, cm^{-1}): 2958, 2122, 1926, 1716, 1605, 1509, 1436, 1344, 1266, 1194, 1142, 1109, 1020, 978, 865, 832, 760, 731, 695. **HRMS** (APCI) calculated for $C_{10}H_8NF_3O_3$: 247.0451 [M]$^+$, found: 247.0445.

benzyl N-formyl-N-(trifluoromethyl)-L-leucinate (S30)

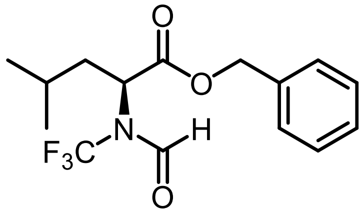

The reaction was performed on a 4 x 2.0 mmol scale according to GP5 and the title product was obtained after column chromatography (5% Et_2O/*n*-pentane) as a colorless oil (1.35 g, 4.25 mmol, 53%).

R_f = 0.68 (10% Et_2O/*n*-pentane). **^{1}H NMR** (400 MHz, Methylene Chloride-*d*$_2$) δ 8.64 (s, 1H), 7.45 – 7.22 (m, 5H), 5.27 – 5.05 (m, 2H), 4.78 – 4.68 (m, 1H), 2.08 – 1.94 (m, 1H), 1.92 – 1.81 (m, 1H), 1.70 – 1.57 (m, 1H), 0.94 (d, *J* = 7.0 Hz, 3H), 0.92 (d, *J* = 6.7 Hz, 3H). **^{19}F NMR** (376 MHz, Methylene Chloride-*d*$_2$) δ -55.59 (s, 3F)**.** **^{13}C{^{1}H} NMR** (151 MHz, Methylene Chloride-*d*$_2$) δ 169.4, 159.4, 135.9, 129.1, 128.9, 128.6, 122.0 (q, *J* = 266.5 Hz), 68.1, 38.2, 25.3, 23.1, 21.6. **IR** (neat, cm^{-1}): 3036, 2961, 2876, 2327, 2078, 1747, 1718, 1459, 1368, 1326, 1259, 1218, 1144, 1026, 968, 920, 738, 697. **HRMS** (APCI) calculated for $C_{15}H_{18}NO_3F_3$: 340.1124 [M+Na]$^+$, found: 340.1131.

tert-butyl 4-(N-(trifluoromethyl)formamido-d)piperidine-1-carboxylate (S31)

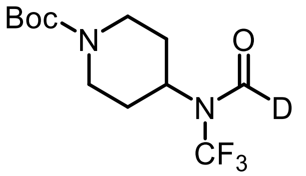

The reaction was performed on a 1.20 mmol scale according to GP5 and the title product was obtained after column chromatography (10% EtOAc/*n*-pentane) as a colorless solid (230 mg, 0.77 mmol, 64%, 89% deuteration).

***R**$_f$* = 0.33 (10% EtOAc/*n*-pentane). **^{1}H NMR** (600 MHz, Chloroform-*d*) δ 4.27 – 4.01 (m, 3H), 2.69 (s, 2H), 2.00 (q, *J* = 13.9, 12.2 Hz, 2H), 1.71 (d, *J* = 11.9 Hz, 2H), 1.42 (s, 9H). **^{19}F NMR** (376 MHz, Chloroform-*d*) δ -55.51 (s, 3F). **^{13}C{^{1}H} NMR** (151 MHz, Chloroform-*d*) δ 158.9, 154.5, 121.7 (q, *J* = 265.6 Hz), 79.9, 52.2, 43.8, 29.37, 28.4. **IR** (neat, cm^{-1}): 2976, 2944, 2871, 2260, 2081, 1982, 1765, 1677, 1425, 1359, 1308, 1253, 1126, 1027, 980, 947, 889, 860, 822, 764, 720, 657. **HRMS** (ESI) calculated for $C_{12}H_{18}{}^2HN_2O_2F_3$: 320.1303 [M+Na]$^+$, found: 320.1305.

N-(9-ethyl-9H-carbazol-3-yl)-N-(trifluoromethyl)formamide (S32)

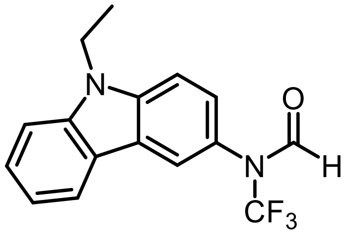

The reaction was performed on a 1.07 mmol scale according to GP5 and the title product was obtained after column chromatography (5% EtOAc/*n*-pentane) as a yellow solid (235 mg, 0.76 mmol, 72%).

***R**$_f$* = 0.39 (5% EtOAc/*n*-pentane). **^{1}H NMR** (600 MHz, Chloroform-*d*) δ 9.03 (s, 1H), 8.09 (d, *J* = 7.8 Hz, 1H), 8.02 (s, 1H), 7.53 (dd, *J* = 7.7, 7.7 Hz, 1H), 7.47 (d, *J* = 8.6 Hz, 1H), 7.44 (d, *J* = 8.2 Hz, 1H), 7.34 (d, *J* = 8.6 Hz, 1H), 7.28 (dd, *J* = 7.4, 7.4 Hz, 1H), 4.37 (q, *J* = 7.3 Hz, 2H), 1.45 (t, *J* = 7.3 Hz, 3H). **^{19}F NMR** (564 MHz, Chloroform-*d*) δ -56.62 (s, 3F). **^{13}C{^{1}H} NMR** (151 MHz, Chloroform-*d*) δ 159.8, 140.6, 140.2, 126.7, 126.1, 123.7, 122.5 (br, 2C), 121.5, 120.8, 119.6, 109.3, 109.0, 37.8, 13.9. *Note*: Despite an increased number of scans the signal of the CF_3 group could not be observed in ^{13}C NMR. **IR** (neat, cm^{-1}): 3051, 2982, 2938, 2895, 2161, 2113, 2063, 2019, 1990, 1936, 1808, 1720, 1629, 1599, 1481, 1324, 1265, 1230, 1133, 979, 937, 792, 749, 690, 658. **HRMS** (APCI) calculated for $C_{16}H_{13}N_2OF_3$: 306.0980 [M]$^+$, found: 306.0972.

Methyl 4-(N-(trifluoromethyl)formamido)thiophene-2-carboxylate (S33)

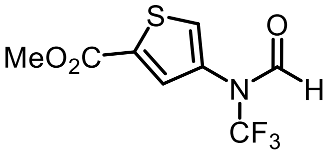

The reaction was performed on a 1.96 mmol scale according to GP5 and the title product was obtained after column chromatography (10% EtOAc /*n*-pentane) as a yellow solid (289 mg, 1.14 mmol, 58%).

***R**$_f$* = 0.57 (10% EtOAc /*n*-pentane). **^{1}H NMR** (400 MHz, Chloroform-*d*) δ 8.81 (brs, 1H), 7.69 (s, 1H), 7.57 (s, 1H), 3.91 (s, 3H).**^{19}F NMR** (376 MHz, Chloroform-*d*) δ -57.28 (s, 3F). **^{13}C{^{1}H} NMR** (151 MHz, Chloroform-*d*) δ 161.5, 158.4, 134.0, 131.7, 130.5, 120.9 (q, *J* = 264.3 Hz), 52.5, 41.9. **IR** (neat, cm^{-1}): 3359, 3106, 2955, 2324, 2096, 1921, 1716, 1545, 1449, 1390, 1257, 1152, 1076, 1013, 865, 789, 747. **HRMS** (ESI): calculated for $C_8H_6F_3NO_3S$: 275.9913 [M+Na]$^+$, found 275.9914.

N-(*trans*-2-phenylcyclopropyl)-N-(trifluoromethyl)formamide (S34)

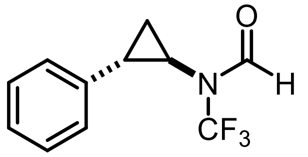

The reaction was performed on a 2.0 mmol scale according to GP5 and the title product was obtained after column chromatography (5% Et_2O/*n*-pentane) as a colorless oil (280 mg, 1.22 mmol, 61%).

***R*$_f$** = 0.57 (5% Et_2O/*n*-pentane). **^{1}H NMR** (600 MHz, Chloroform-*d*) δ 8.59 (s, 1H), 7.26 – 7.19 (m, 2H), 7.18 – 7.11 (m, 3H), 2.55 (s, 1H), 2.30 (t, *J* = 6.5 Hz, 1H), 1.44 – 1.35 (m, 2H). **^{19}F NMR** (565 MHz, Chloroform-*d*) δ -56.87 (s, 3F). **^{13}C{^{1}H} NMR** (151 MHz, Chloroform-*d*) δ 160.1, 138.8, 128.7, 127.1, 127.0, 121.7 (q, *J* = 266.3 Hz), 32.0, 24.3, 14.4. **IR** (neat, cm^{-1}): 3032, 2117, 1830, 1722, 1500, 1355, 1273, 1144, 1090, 997, 913, 789, 746, 696. **HRMS** (APCI) calculated for $C_{11}H_{10}NOF_3$: 229.0709 [M]$^+$, found: 229.0709.

(*R*)-N-(1-(3-(2-cyanobenzyl)-1-methyl-2,6-dioxo-1,2,3,6-tetrahydropyrimidin-4-yl)piperidin-3-yl)-N-(trifluoromethyl)formamide (S35)

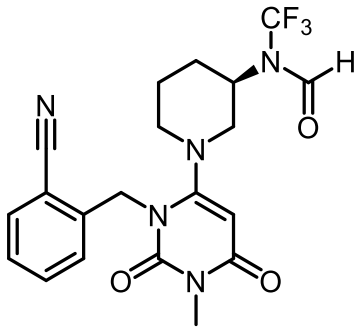

The reaction was performed on a 1.5 mmol scale according to GP1 and the title product was obtained after column chromatography (70% EtOAc/*n*-pentane) as a colorless solid (115 mg, 0.263 mmol, 18%).

***R*$_f$** = 0.41 (70% EtOAc/*n*-pentane) **^{1}H NMR** (600 MHz, Methylene Chloride-*d*$_2$) δ 8.57 (s, 1H), 7.69 (d, *J* = 7.7 Hz, 1H), 7.56 (dd, *J* = 7.2, 7.2 Hz, 1H), 7.39 (dd, *J* = 7.5, 7.5 Hz, 1H), 7.17 (d, *J* = 7.9 Hz, 1H), 5.38 (s, 1H), 5.29 (d, *J* = 16.0 Hz, 1H), 5.21 (d, *J* = 16.0 Hz, 1H), 3.98 (t, *J* = 11.7 Hz, 1H), 3.24 (s, 3F), 3.17 – 3.02 (m, 3H), 2.59 (t, *J* = 11.5 Hz, 1H), 2.15 – 2.04 (m, 1H), 1.98 – 1.90 (m, 1H), 1.87 – 1.80 (m, 1H), 1.76 – 1.64 (m, 1H). **^{19}F NMR** (565 MHz, Methylene Chloride-*d*$_2$) δ -56.69 (s, 3F). **^{13}C NMR** (151 MHz, Methylene Chloride-*d*$_2$) δ 163.1, 159.4, 159.2, 152.9, 141.2, 133.6, 133.5, 128.3, 127.2, 121.8 (q, *J* = 264.1 Hz), 117.5, 111.3, 91.4, 53.4, 52.0, 51.4, 46.6, 28.0, 27.1, 25.0. **IR** (neat, cm^{-1}): 2955, 2855, 2219, 2163, 1956, 1704, 1651, 1438, 1355, 1317, 1251, 1217, 1135, 994, 955, 809, 764, 733, 699. **HRMS** (ESI) calculated for $C_{20}H_{20}N_5O_3F_3Na$: 458.1411 [M+Na]$^+$, found: 458.1400.

5. Synthesis of N-CF_3 amine (1-Me)

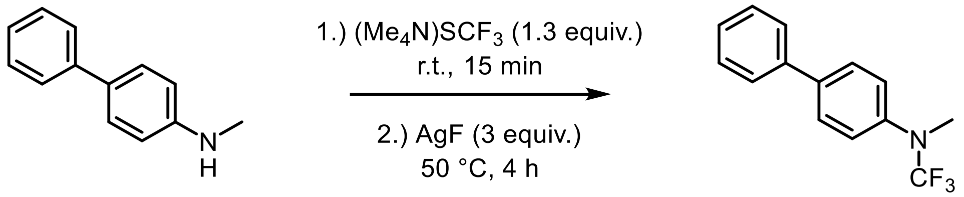

Under argon atmosphere an amber-colored 4 mL vial was charged with $(Me_4N)SCF_3$ (46 mg, 0.26 mmol, 1.3 equiv.). To this, the biphenyl-NHMe (36.6 mg, 0.2 mmol, 1 equiv.) was added as a solution in 1.5 mL MeCN. The mixture was stirred for 15 min at room temperature and subsequently AgF (76 mg, 0.6 mmol, 3 equiv.) was added to the solution and the vial was closed tightly. The reaction mixture was stirred at 50 °C for 4 h. Afterwards the reaction mixture was allowed to cool to ambient temperature. The crude

mixture was filtered over a celite pad eluting with a mixture of pentane and CH_2Cl_2 (1:1). The obtained filtrate was concentrated under reduced pressure to yield the title product as a colorless solid (28.8 mg, 0.115 mmol, 58%).

^{1}H NMR (600 MHz, Chloroform-*d*) δ 7.60 – 7.55 (m, 4H), 7.44 (dd, *J* = 7.8, 7.8 Hz, 2H), 7.35 (t, *J* = 7.4 Hz, 1H), 7.32 (d, *J* = 8.2 Hz, 2H), 3.08 (d, *J* = 1.0 Hz, 3H). **^{19}F NMR** (376 MHz, Methylene Chloride-d_2) δ -60.57 (s, 3F). **^{13}C{^{1}H} NMR** (151 MHz, Chloroform-*d*) δ 142.1, 140.5, 139.2, 129.0, 127.9, 127.5, 127.2, 125.1, 123.6 (q, *J* = 256.1 Hz), 36.4. **HRMS** (ESI) calculated for $C_{14}H_{12}NF_3$: 251.0916 $[M]^+$, found: 251.0913.

6. Attempts to access other *N*-CF_3 and *N*-CF_2H functionalities

6.1 Unsuccessful attempts of desulfurization-fluorination protocols

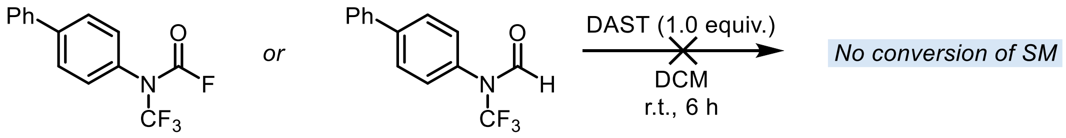

Under argon atmosphere, a solution of DAST (0.5 mmol) in 1.5 mL DCM was added dropwise to a vial containing a solution of the starting material (0.5 mmol) in 1.5 mL DCM. The solution was stirred at room temperature for 6 h. It was quenched with sat. aq. $NaHCO_3$ solution and the organic layer was separated. An aliquot was checked by NMR and GCMS, showing no conversion of the starting material.

6.2 Unsuccessful attempts of sulfurization reactions with Lawesson's reagent

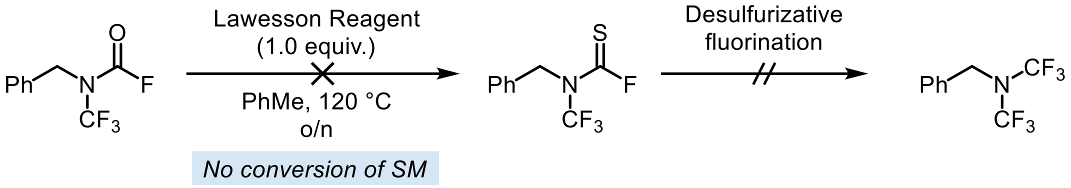

Under argon atmosphere, a 20 mL vial was charged with the *N*–CF_3 carbamoyl fluoride (0.40 mmol scale) and toluene was added [0.2 M]. Subsequently Lawesson's reagent (1.0 equiv.) was added to the solution and the vial was tightly closed with a screw cap. The reaction mixture was stirred overnight at 120 °C. Afterwards the reaction mixture was cooled down and analyzed by NMR and GC-MS. No conversion of starting material or product formation was observed.

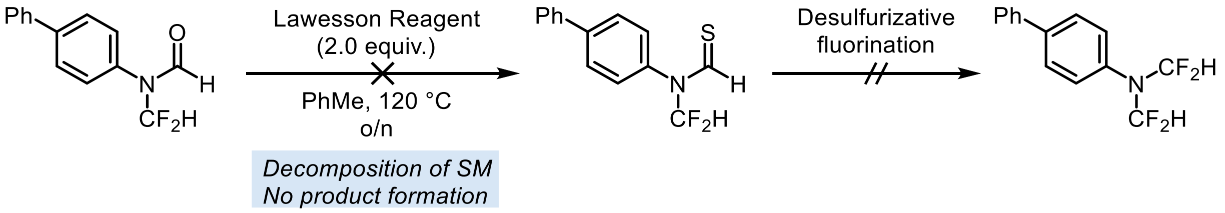

Under argon atmosphere, a 20 mL vial was charged with the *N*–CF_2H formamide (0.15 mmol scale) and toluene was added [0.2 M]. Subsequently Lawesson's reagent (2.0 equiv.) was added to the solution and the vial was tightly closed with a screw cap. The reaction mixture was stirred overnight at 120 °C. Afterwards the reaction mixture was cooled down and analyzed by NMR and GC-MS. Starting material decomposed to different products, one of them identified as the derived isothiocyanate, without formation of the desired *N*–CF_2H thioformamide.

7. Stability tests

7.1.1 Stability Study in buffer solution/MeCN 1:1 at pH 1.0, 7.4 and 10.1

Three buffer solutions were prepared: 0.1 M HCl solution (pH 1.1), 20 mM sodium phosphate buffer (pH 7.4) and 20 mM sodium carbonate buffer (pH 10.1). For the stability study the corresponding compounds (**1**, **6** or **1-Me**) were dissolved in 50 mL of MeCN (1 µmol/mL) and subsequently buffer solution (50 mL) was added to obtain a 1:1 MeCN/buffer mixture. From these stock solutions, 4 mL were transferred to a vial and stirred at the given temperature without any further protection against light or moisture. Aliquots of 300 µL were taken and HPLC analysis was performed after 0 min, 1 h, 7 h, 24 h, 72 h, 1 week, 3 weeks and 6 weeks. The percentage of remaining compound was determined by linear regression from a calibration curve.

Note: Since no internal standard was used, any deviations in concentration caused by evaporation of solvent cannot be accounted for. However, these effects should be negligible at lower temperature (r.t. and 37°C) and minimal also at 70°C given the high boiling points of the used solvents (H_2O/MeCN).

Tables S2-S6 summarize the obtained data. Half-life times $t_{1/2}$ were determined by plotting ln(conc.) versus time and using the slope k determined by linear regression to compute $t_{1/2} = -\frac{\ln(2)}{k}$.

Table S2. Percentage of remaining compound **1** over time under basic conditions.

**Time (h)**		**Remaining compound 1 (%)**		
		pH 10.1, r.t.	pH 10.1, 37°C	pH 10.1, 70°C
**0**		100%	100%	100%
**1**		99%	99%	100%
**7**		102%	102%	101%
**24**	1 day	104%	99%	99%
**72**	3 days	101%	100%	101%
**168**	1 week	103%	105%	101%
**504**	3 weeks	99%	100%	93%
**1008**	6 weeks	102%	101%	83%

Table S3. Percentage of remaining compound **1** over time under neutral conditions.

**Time (h)**		**Remaining compound 1 (%)**		
		pH 7.4, r.t.	pH 7.4, 37°C	pH 7.4, 70°C
**0**		100%	100%	100%
**1**		100%	101%	100%
**7**		101%	102%	102%
**24**	1 day	102%	102%	103%
**72**	3 days	99%	99%	99%
**168**	1 week	101%	100%	102%
**504**	3 weeks	99%	98%	91%
**1008**	6 weeks	103%	101%	86%

Table S4. Percentage of remaining compound **1** over time under acidic conditions.

Time (h)		Remaining compound 1 (%)		
		pH 1.0, r.t.	pH 1.0, 37°C	pH 1.0, 70°C
**0**		100%	100%	100%
**1**		95%	93%	70%
**7**		89%	78%	5%
**24**	1 day	78%	49%	0%
**72**	3 days	55%	11%	
**168**	1 week	27%	0%	
**504**	3 weeks	2%		
**1008**	6 weeks	0%		

Table S5. Percentage of remaining compound **6** over time.

Time (h)		Remaining compound 6 (%)	
		pH 1.0, 37°C	pH 7.4, 37°C
**0**		100%	100%
**1**		95%	99%
**7**		80%	100%
**24**	1 day	53%	99%
**72**	3 days	14%	101%
**168**	7 days	0%	100%

Table S6. Percentage of remaining compound **1-Me** over time.

Time (h)		Remaining compound 1-Me (%)		
		pH 1.0, 37°C	pH 7.4, 37°C	pH 10.1, 37°C
**0**		100%	100%	100%
**1**		0%	78%	79%
**7**			33%	48%
**24**	1 day		13%	11%
**72**	3 days		0%	0%

8. Determination of Hansch-Leo parameter (π_R)

Hansch-Leo parameter was determined following a reported procedure.[4] To a 10 mL flask was added octanol (2 mL), water (2 mL) and the molecule to study (ca. 2 µL). The resulting biphasic mixture was hand-shacked for 5 min and then the flask was centrifuged for 5 min to enable complete phase separation. Using two syringes with needles, a sample was carefully taken from each layer. A small amount of water phase was discarded to ensure all traces of octanol were out of the needle. The needles were carefully dried before transferring the sample into the HPLC vial.

LogP was determined as the logarithm of the ratio between the peak areas of molecules in octanol and water.

The Hansch-Leo parameter (Table S7) was calculated as π_R = logP(molecule) – logP(benzene).

Table S7. Hansch-Leo parameter.

Entry	Compound	logP	π_R
1	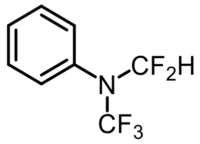	3.21	1.08

9. [*A*] value determination (Abrahams NMR method)

The evaluation of hydrogen bonding acidity properties was performed using Abraham's method.[5] For this purpose, solutions of the corresponding compound were prepared in $CDCl_3$ and DMSO-d_6 and ^1H NMR spectra were recorded. The difference in chemical shift (Δδ, ppm) of the to be investigated proton was determined (($\Delta\delta = \delta(DMSO) - \delta(CDCl_3)$) and the [*A*] value was calculated using the following equation: $[A] = 0.0065 + 0.133\Delta\delta$. Table S8 summarizes the experimental [*A*] values obtained.

Table S8. ^1H NMR chemical shifts and [*A*] values of selected compounds.

Entry	Compound	δ (ppm, $CDCl_3$)	δ (ppm, DMSO-*d6*)	Δδ	[*A*] value
1	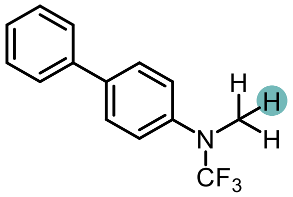	3.09	3.06	0.03	0.010
2	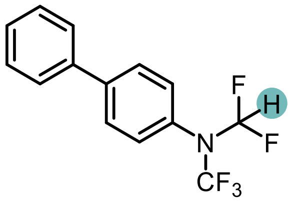	6.71	7.43	072	0.102

10. Enantioretention Study

For compounds **9-L**, **9-D**, **S9-L** and **S9-D** both enantiomers were synthesized separately using the same method described in the experimental section. The spectroscopic data obtained was as expected.

benzyl N-thioformyl-N-(trifluoromethyl)-L-leucinate (S9)

Column: Daicel Chiralcel OJ-H, (250 x 4,6) mm; Mobile phase: n-Hexane/iPrOH 99.5:0.5

Temperature in °C: 30.0; Flow in ml/min: 0.75; 45 min

Racemic HPLC trace of S9

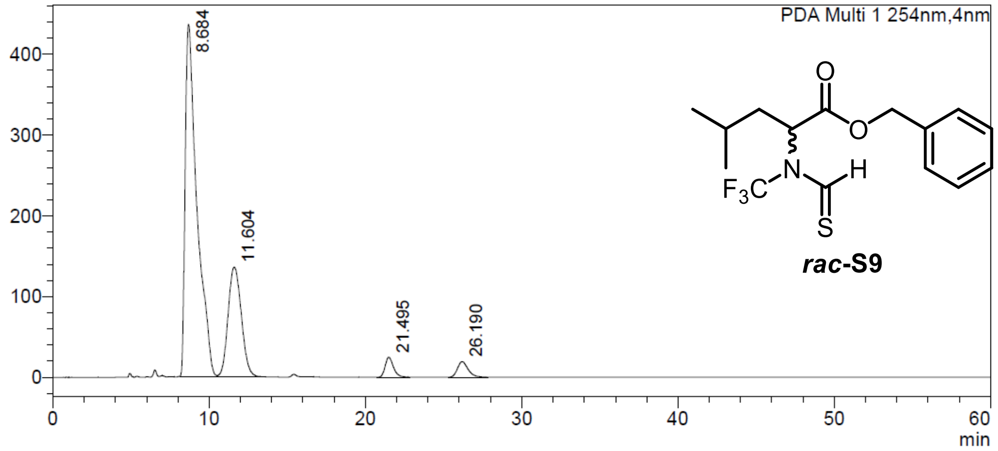

HPLC trace of S9-L

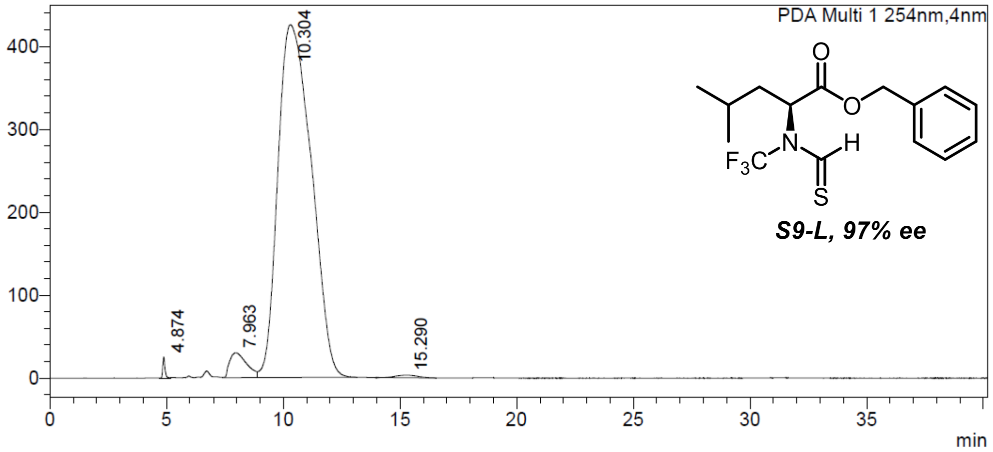

HPLC trace of S9-D

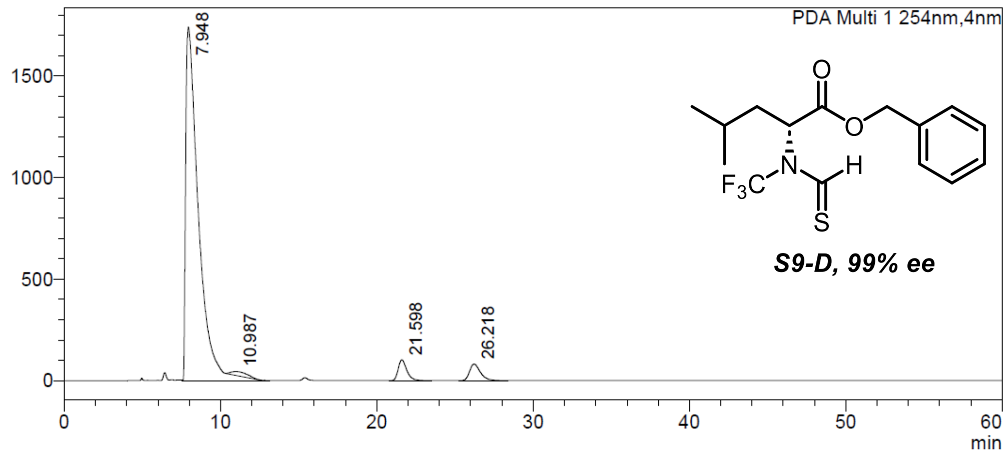

benzyl N-(difluoromethyl)-N-(trifluoromethyl)-L-leucinate (9)

Column: Daicel Chiralcel OJ-H, (250 x 4,6) mm; Mobile phase: n-Hexane/iPrOH 99:1

Temperature in °C: 30.0; Flow in ml/min: 0.75; 60 min.

Racemic HPLC trace of 9-rac

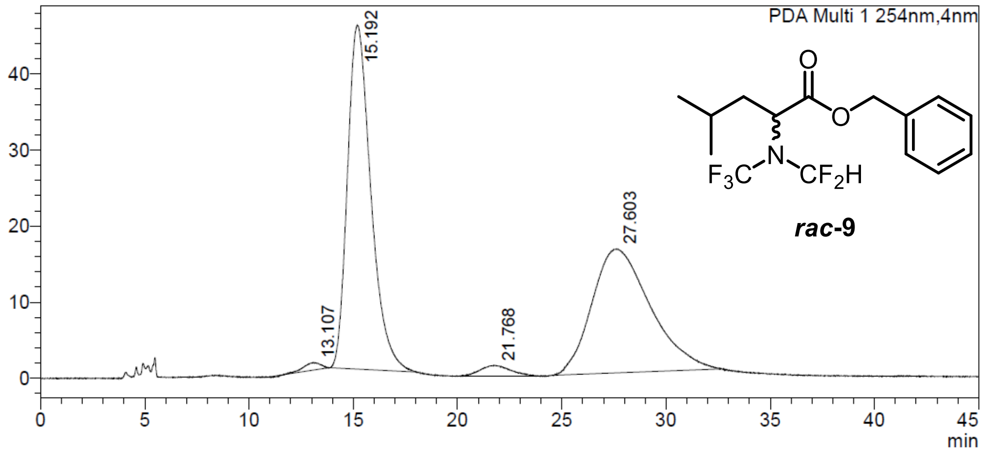

HPLC trace of 9-L

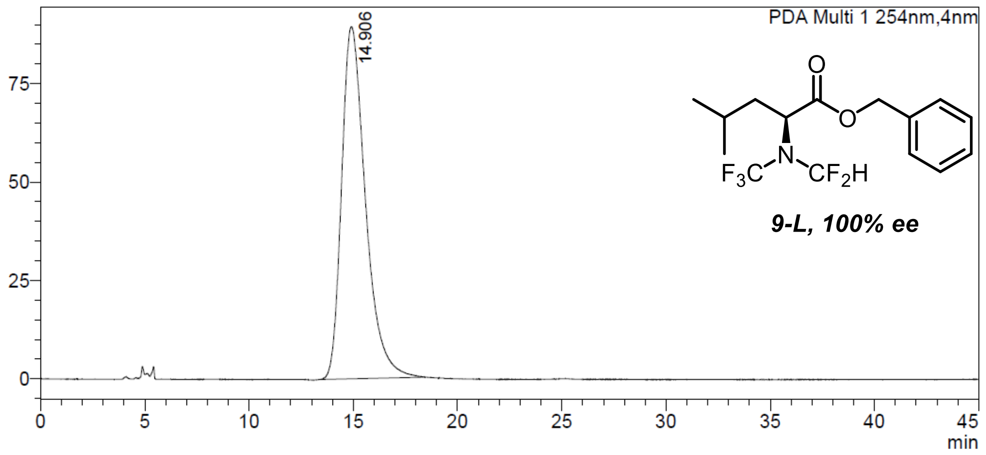

HPLC trace of 9-D

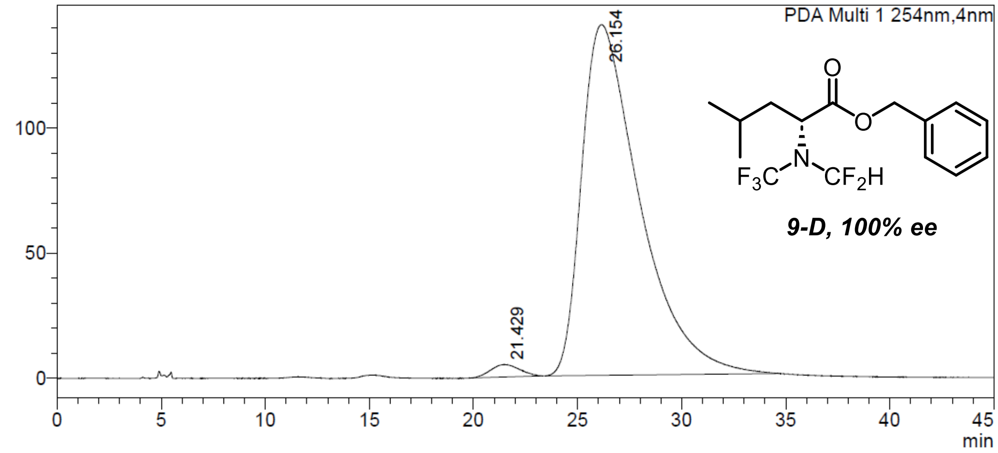

11. Computational details

11.1 Computed log*P* values

The octanol-water partition coefficient (log*P*) was calculated using COSMOtherm.[6] Input structures for COSMOtherm were generated from single-point energy calculations at the BP86/def2-TZVPD level of theory using Turbomole,[7] based on the minimum energy conformer geometries obtained after conformational search with xTB[8]/CREST[9] and further optimization at DFT level using Gaussian 16, Revision A.03.[10] Geometry optimizations were conducted in acetonitrile (SMD solvation) at the M06-2X/6-311++G(d,p) level of theory. Frequencies were calculated at the same level of theory and used to verify the nature of all stationary points as minima (no imaginary frequencies). Table S9 summarizes the computed log*P* values.

Table S9. Computed log*P* values of Ph–N–$CF_xH_{(3-x)}$ derivatives.

Entry	Compound	log*P* (wet octanol)	log*P* (dry octanol)
1	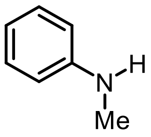	2.02	1.98
2	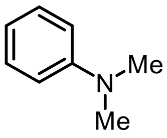	2.63	2.64
3	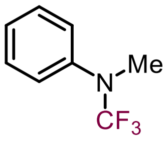	3.70	3.77
4	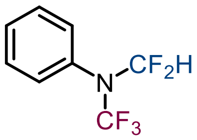	3.76	3.80
5	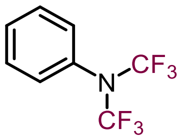	4.28	4.38

11.1.1 XYZ coordinates of computed structures

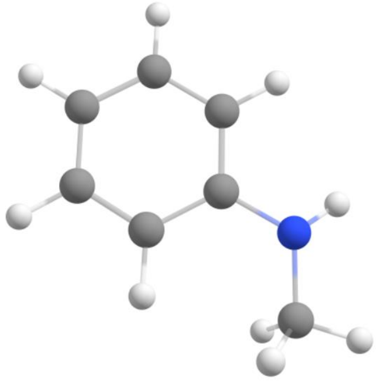

N	1.78432	-0.61622	-0.15579
C	2.81386	0.37111	0.11224
H	2.82310	1.13850	-0.66540
H	2.68017	0.86521	1.08297
H	3.78111	-0.12946	0.10326
C	0.44530	-0.28079	-0.06353
C	-0.52515	-1.29949	-0.00592
C	0.01221	1.05466	-0.05974
C	-1.87471	-0.98897	0.04585
C	-1.34906	1.35056	-0.01235
C	-2.30350	0.34070	0.04082
H	-0.19918	-2.33502	-0.00390
H	0.73314	1.86207	-0.09602
H	-2.60124	-1.79322	0.09182
H	-1.65994	2.38986	-0.01267
H	-3.35944	0.57949	0.08075
H	1.99836	-1.55049	0.16551

Zero-point correction = 0.145937 (Hartree/Particle)
Thermal correction to Energy = 0.153104
Thermal correction to Enthalpy = 0.154049
Thermal correction to Gibbs Free Energy = 0.114744
Sum of electronic and zero-point Energies = -326.716212
Sum of electronic and thermal Energies = -326.709044
Sum of electronic and thermal Enthalpies = -326.708100
Sum of electronic and thermal Free Energies = -326.747405

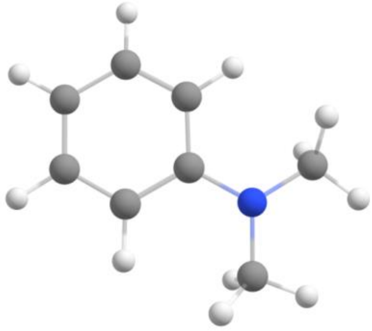

N	-1.56777	-0.00092	-0.20587
C	-2.27800	-1.23645	0.08720
C	-2.28011	1.23580	0.07568
H	-2.10198	1.59613	1.09833
H	-1.98719	2.02125	-0.62324
H	-3.34687	1.06083	-0.05196
H	-1.99727	-2.02357	-0.61500
H	-2.08462	-1.59564	1.10737
H	-3.34631	-1.05961	-0.02333
C	-0.18711	0.00011	-0.09660
C	0.54483	-1.20603	-0.04846
C	0.54406	1.20636	-0.04217
C	1.93294	-1.19554	0.01939
C	1.93228	1.19616	0.02575
C	2.64555	0.00048	0.05321
H	0.03220	-2.15814	-0.06587
H	0.03124	2.15842	-0.05342
H	2.46118	-2.14258	0.05148
H	2.45994	2.14333	0.06348
H	3.72742	0.00064	0.10915

Zero-point correction = 0.174407 (Hartree/Particle)
Thermal correction to Energy = 0.182985
Thermal correction to Enthalpy = 0.183929
Thermal correction to Gibbs Free Energy = 0.140814
Sum of electronic and zero-point Energies = -365.980397
Sum of electronic and thermal Energies = -365.971819
Sum of electronic and thermal Enthalpies = -365.970875
Sum of electronic and thermal Free Energies = -366.013991

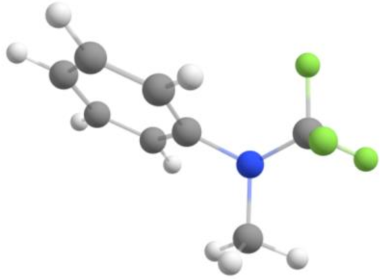

N	-0.76269	0.70605	-0.02852
C	-1.10634	1.71590	0.98828
C	-1.73651	-0.26739	-0.21707
F	-2.90712	0.28631	-0.57529
F	-2.02967	-1.01906	0.88308
H	-0.43964	2.56646	0.86235
H	-0.99519	1.31377	1.99998
H	-2.12993	2.05368	0.83492
F	-1.39015	-1.13401	-1.17507
C	0.61075	0.28128	-0.01631
C	1.55680	1.18084	-0.50745
C	1.01937	-0.95210	0.48986
C	2.90597	0.84877	-0.49313
C	2.37189	-1.28608	0.48134
C	3.31775	-0.38914	-0.00351
H	1.22448	2.13468	-0.90224
H	0.29694	-1.65410	0.88905
H	3.63458	1.55330	-0.87732
H	2.68278	-2.25011	0.86720
H	4.36918	-0.65172	-0.00082

Zero-point correction = 0.150807 (Hartree/Particle)
Thermal correction to Energy = 0.161216
Thermal correction to Enthalpy = 0.162161
Thermal correction to Gibbs Free Energy = 0.113830
Sum of electronic and zero-point Energies = -663.757950
Sum of electronic and thermal Energies = -663.747540
Sum of electronic and thermal Enthalpies = -663.746596
Sum of electronic and thermal Free Energies = -663.794926

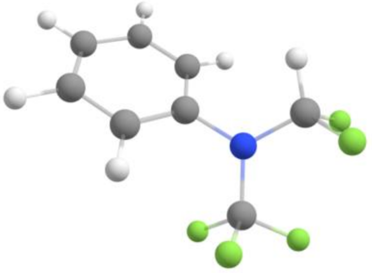

N	0.52114	0.20586	-0.05308

C	1.27878	-0.94547	-0.32235
C	1.17815	1.34861	0.46874
H	0.46006	2.03550	0.90774
F	1.88907	2.00208	-0.49437
F	2.10324	0.99005	1.40499
F	2.50017	-0.61921	-0.75068
F	1.45771	-1.74715	0.74975
F	0.69076	-1.69546	-1.25542
C	-0.91280	0.13031	-0.00069
C	-1.55309	-0.94374	0.61230
C	-1.64736	1.16449	-0.57450
C	-2.94370	-0.98119	0.63678
C	-3.03652	1.13139	-0.51682
C	-3.68743	0.05611	0.08136
H	-0.97898	-1.74117	1.06895
H	-1.13220	1.98260	-1.06582
H	-3.44361	-1.81970	1.10715
H	-3.60784	1.94004	-0.95721
H	-4.77018	0.02593	0.11342

Zero-point correction = 0.135970 (Hartree/Particle)
Thermal correction to Energy = 0.147607
Thermal correction to Enthalpy = 0.148552
Thermal correction to Gibbs Free Energy = 0.096731
Sum of electronic and zero-point Energies = -862.266750
Sum of electronic and thermal Energies = -862.255113
Sum of electronic and thermal Enthalpies = -862.254169
Sum of electronic and thermal Free Energies = -862.305989

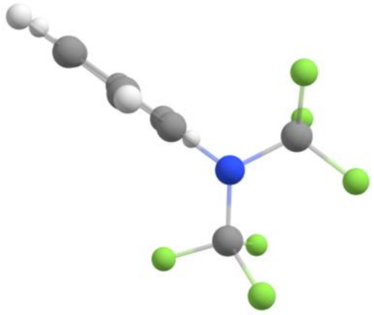

N	-0.52369	0.01141	-0.31518
C	-1.27151	-1.17804	-0.11838
C	-1.14787	1.22951	0.04189
F	-0.60114	2.23818	-0.63340
F	-1.02644	1.53366	1.34686
F	-1.84381	-1.25325	1.09751
F	-2.26600	-1.28790	-1.00432
F	-0.48170	-2.23388	-0.26050
F	-2.45165	1.19136	-0.22612
C	0.91855	-0.02997	-0.14493
C	1.47478	-0.24505	1.11183
C	1.71177	0.16565	-1.26803
C	2.85943	-0.26806	1.24000
C	3.09600	0.14802	-1.12838
C	3.66723	-0.06964	0.12269
H	0.83253	-0.39689	1.97248
H	1.24280	0.32988	-2.23111
H	3.30531	-0.43807	2.21272
H	3.72553	0.30195	-1.99662
H	4.74582	-0.08483	0.22832

Zero-point correction = 0.127019 (Hartree/Particle)
Thermal correction to Energy = 0.139387
Thermal correction to Enthalpy = 0.140331
Thermal correction to Gibbs Free Energy = 0.085925
Sum of electronic and zero-point Energies = -961.523027
Sum of electronic and thermal Energies = -961.510660
Sum of electronic and thermal Enthalpies = -961.509716
Sum of electronic and thermal Free Energies = -961.564122

12. NMR Spectra

12.1 Products

N-(difluoromethyl)-*N*-(trifluoromethyl)-[1,1'-biphenyl]-4-amine (1)

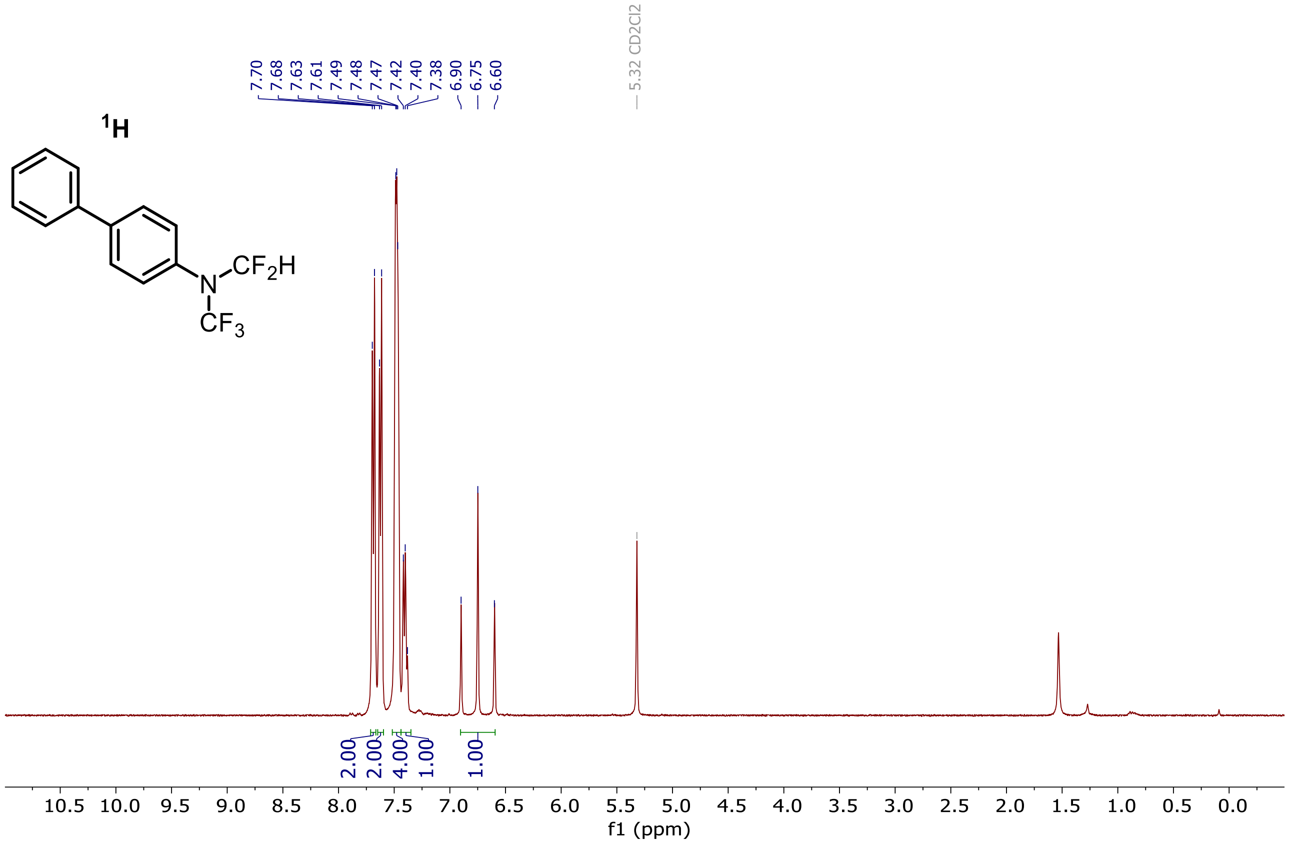

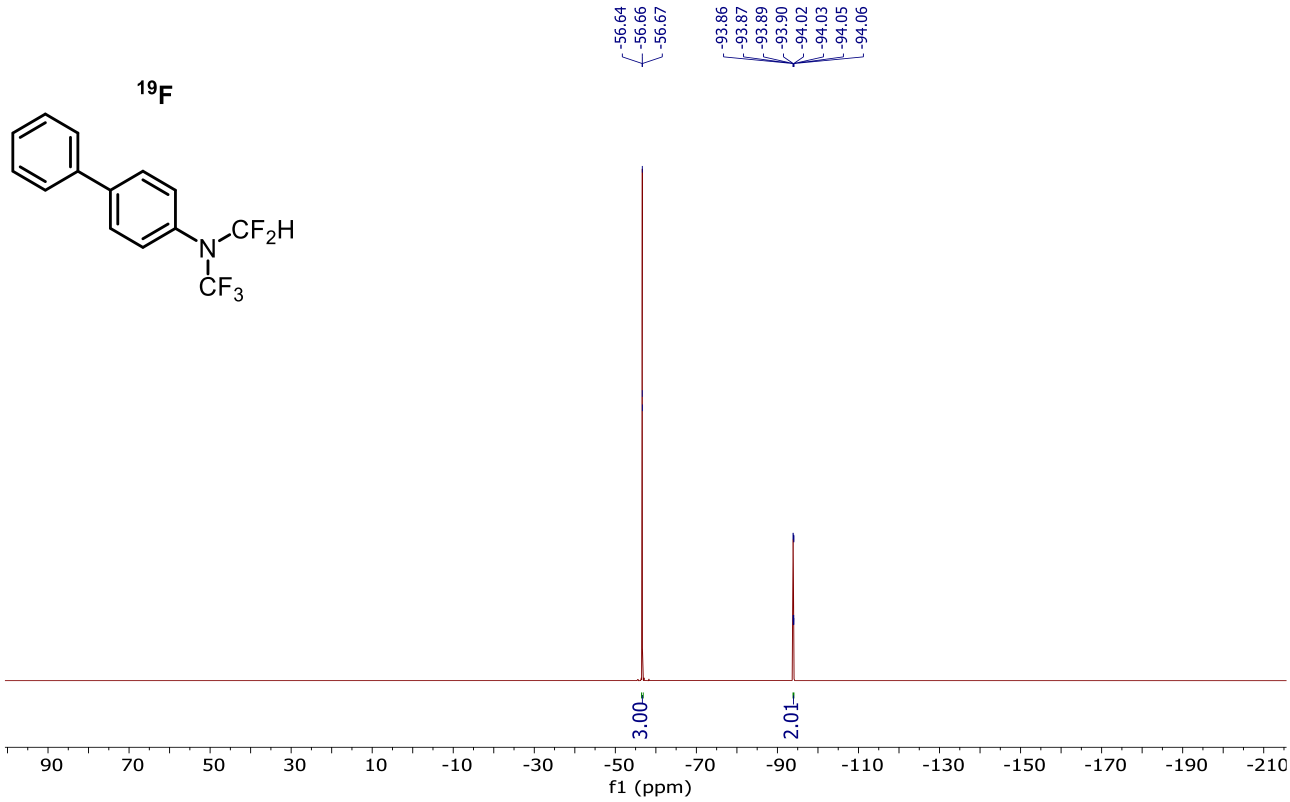

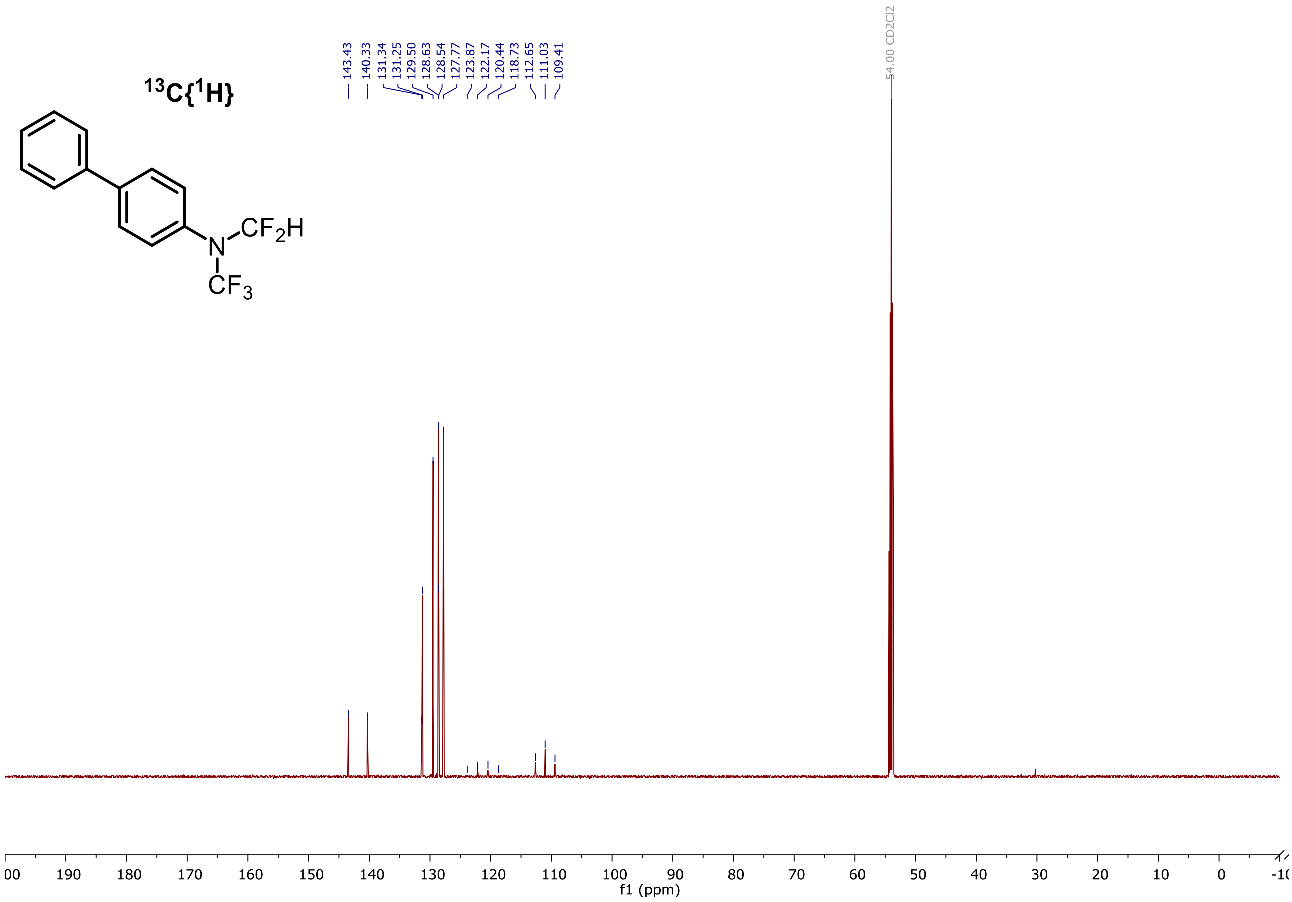

***N*-(difluoromethyl)-*N*-(trifluoromethyl)aniline (2)**

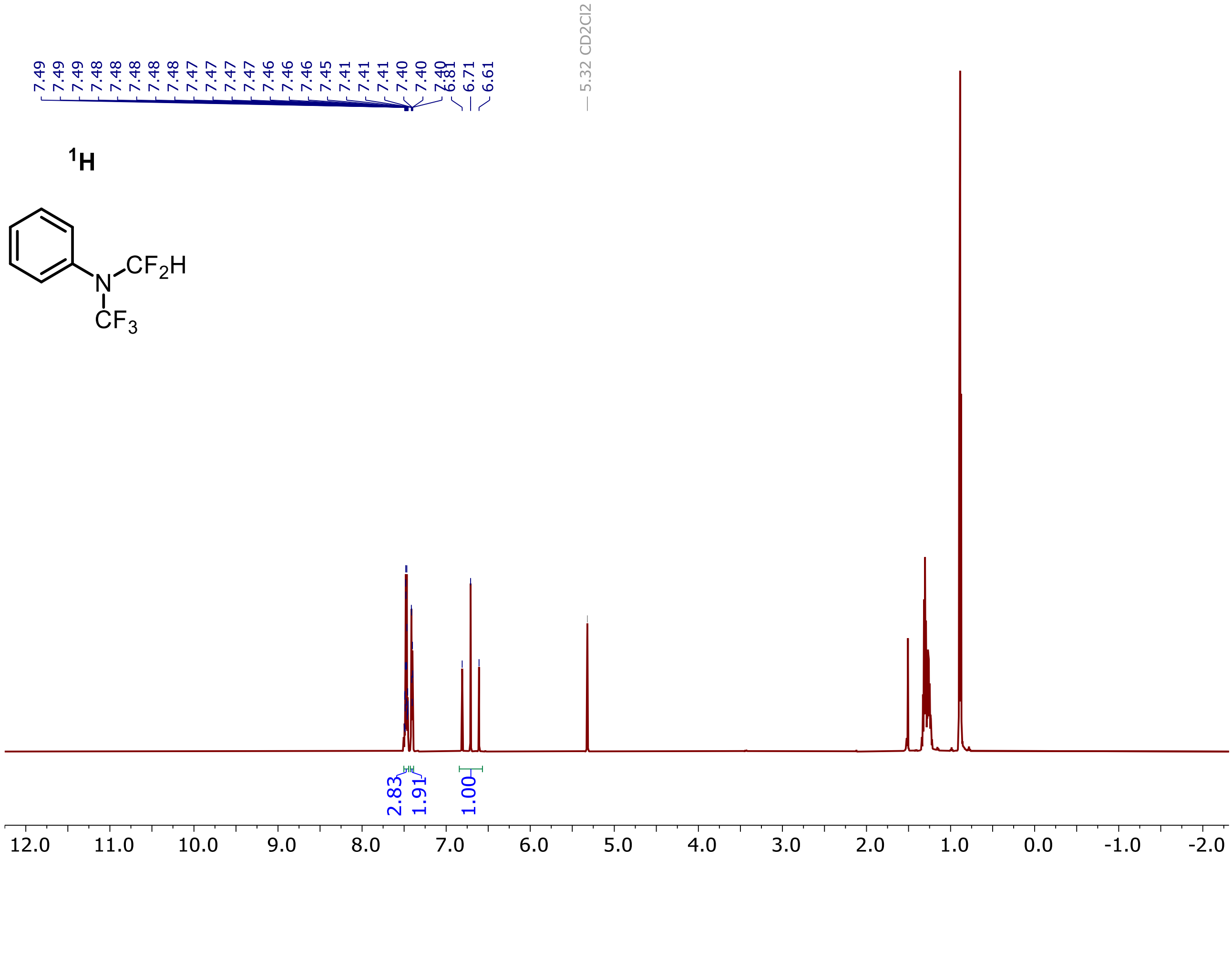

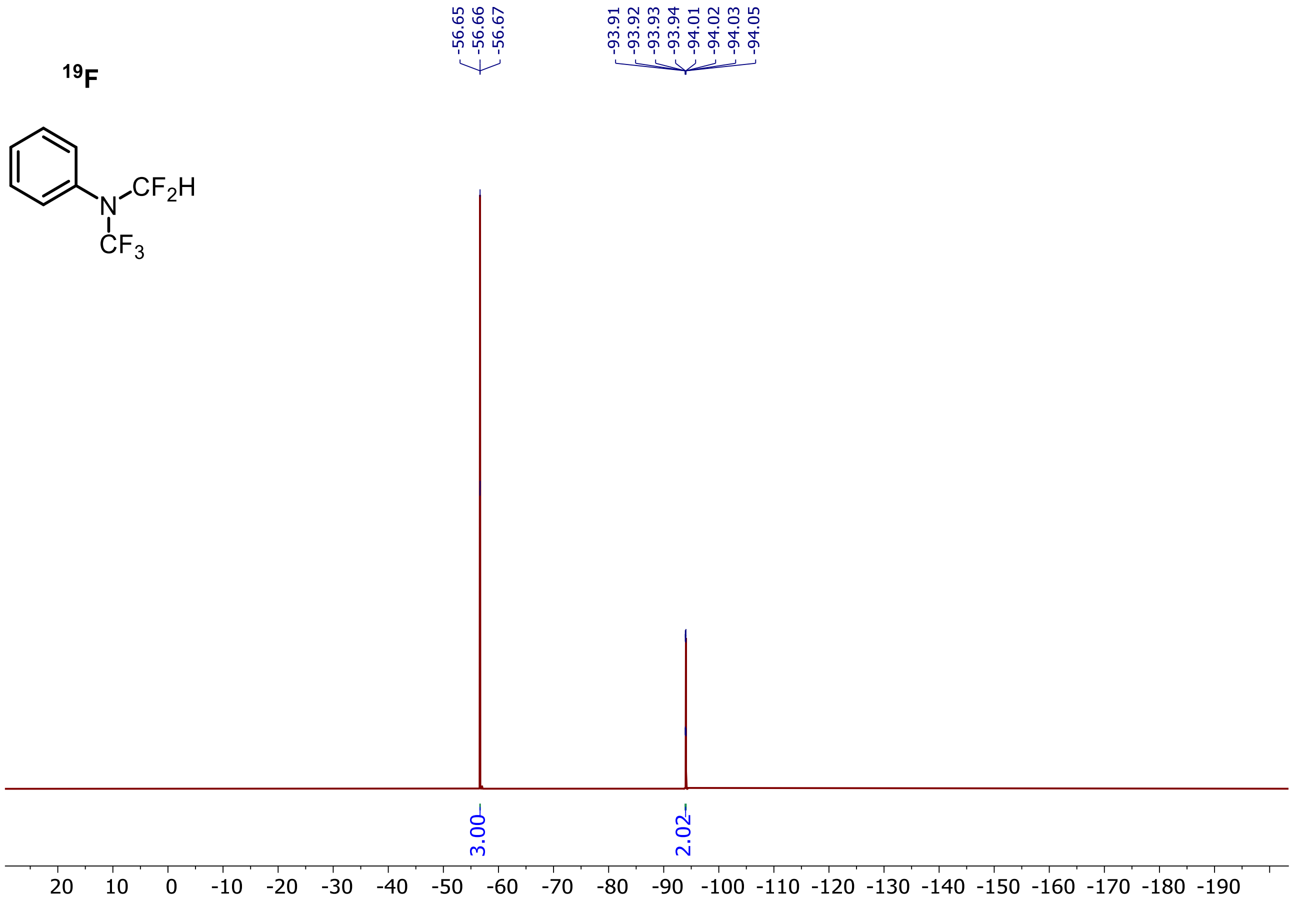

19F
CF2H
N
CF3
-56.65
-56.66
-56.67
-93.91
-93.92
-93.93
-93.94
-94.01
-94.02
-94.03
-94.05
3.00
2.02
20 10 0 -10 -20 -30 -40 -50 -60 -70 -80 -90 -100 -110 -120 -130 -140 -150 -160 -170 -180 -190

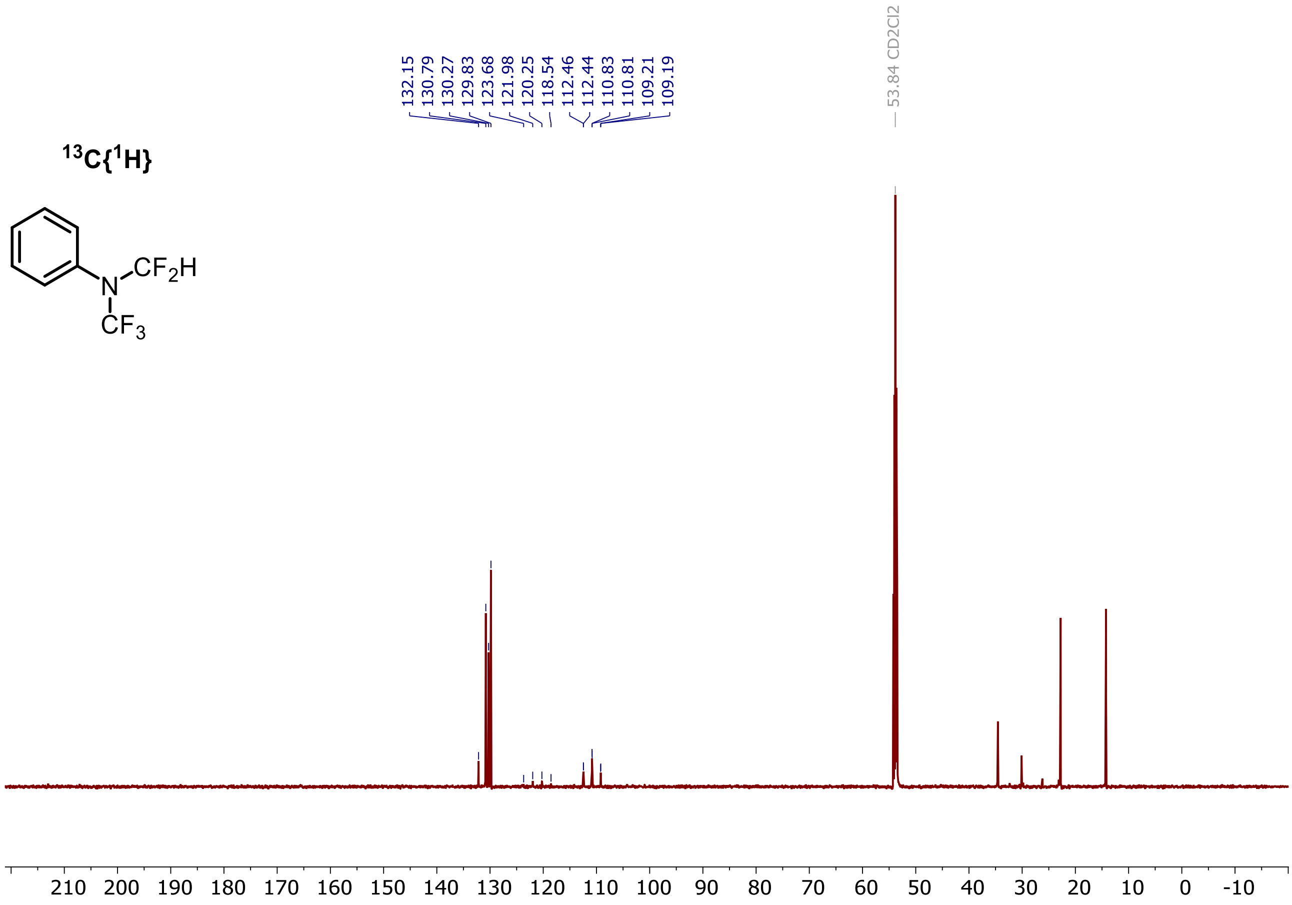

13C{1H}
CF2H
N
CF3
132.15
130.79
130.27
129.83
123.68
121.98
120.25
118.54
112.46
112.44
110.83
110.81
109.21
109.19
53.84 CD2Cl2
210 200 190 180 170 160 150 140 130 120 110 100 90 80 70 60 50 40 30 20 10 0 -10

4-cyclohexyl-*N*-(difluoromethyl)-*N*-(trifluoromethyl)anilinemethyl) (3)

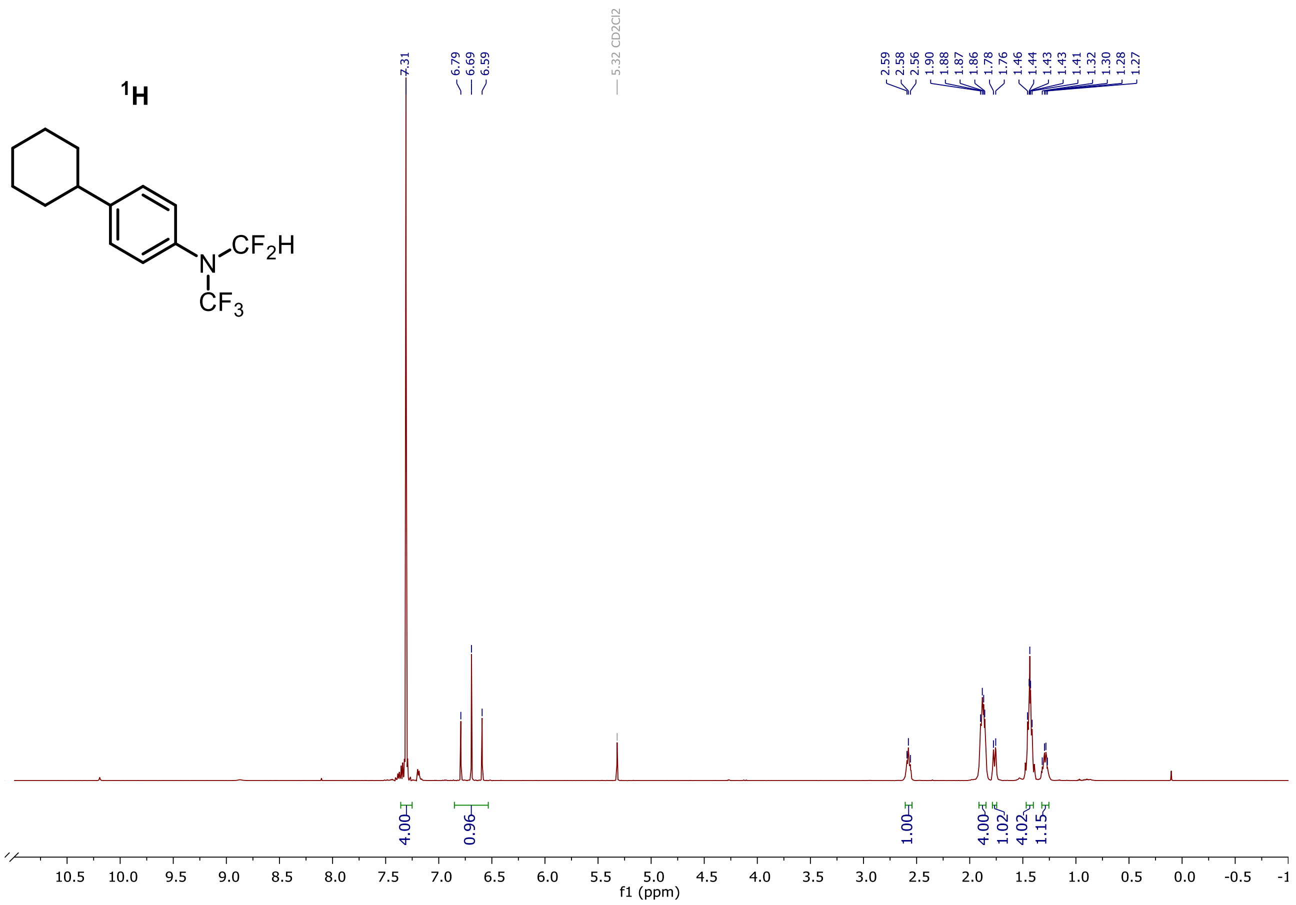

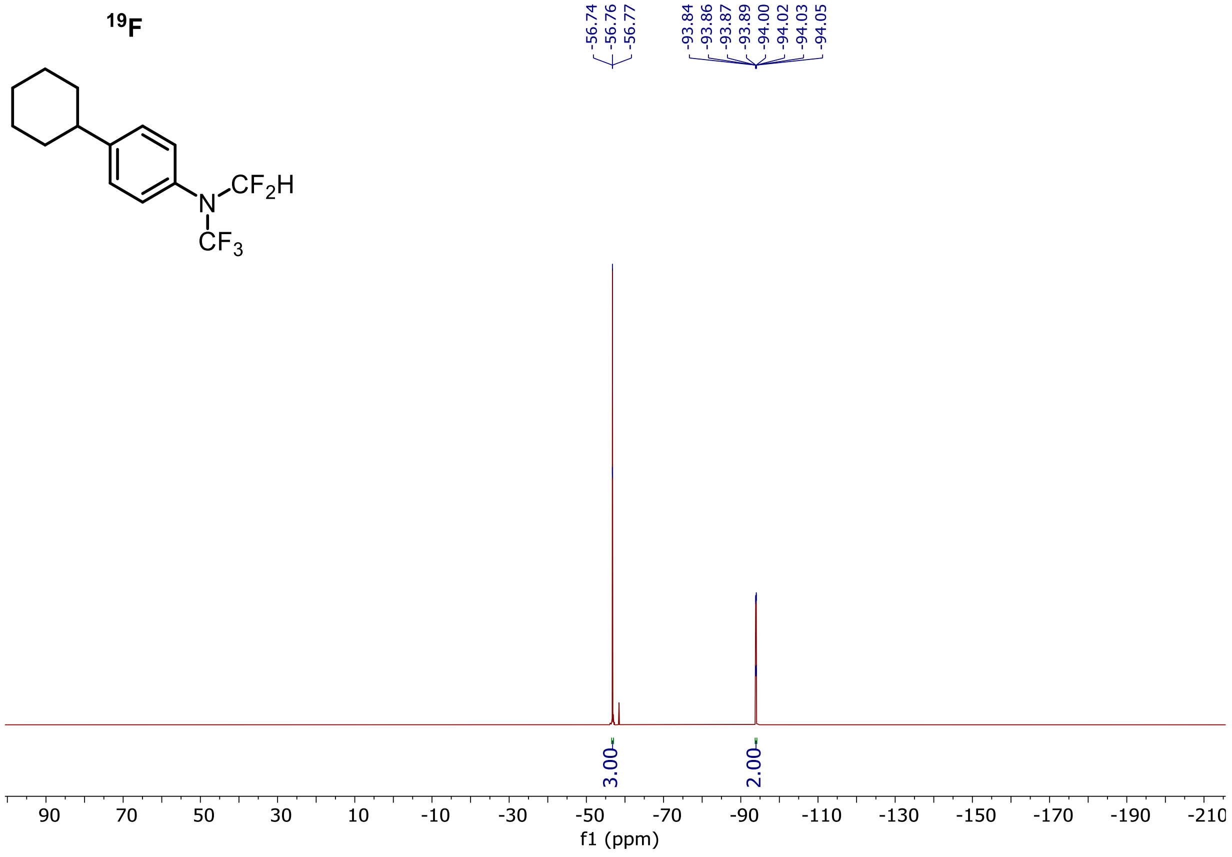

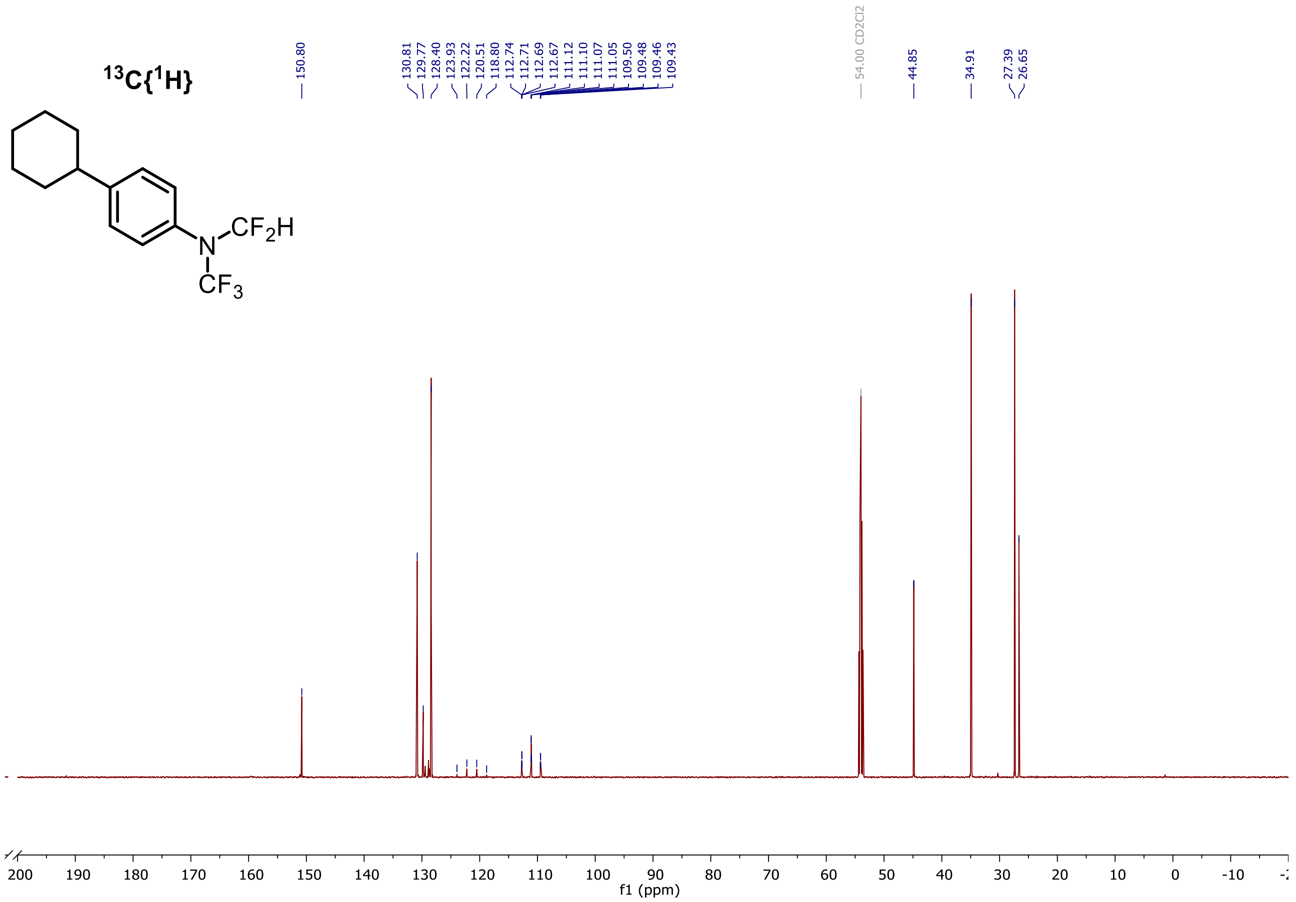

N-(difluoromethyl)-3,4,5-trimethoxy-*N*-(trifluoromethyl)aniline (4)

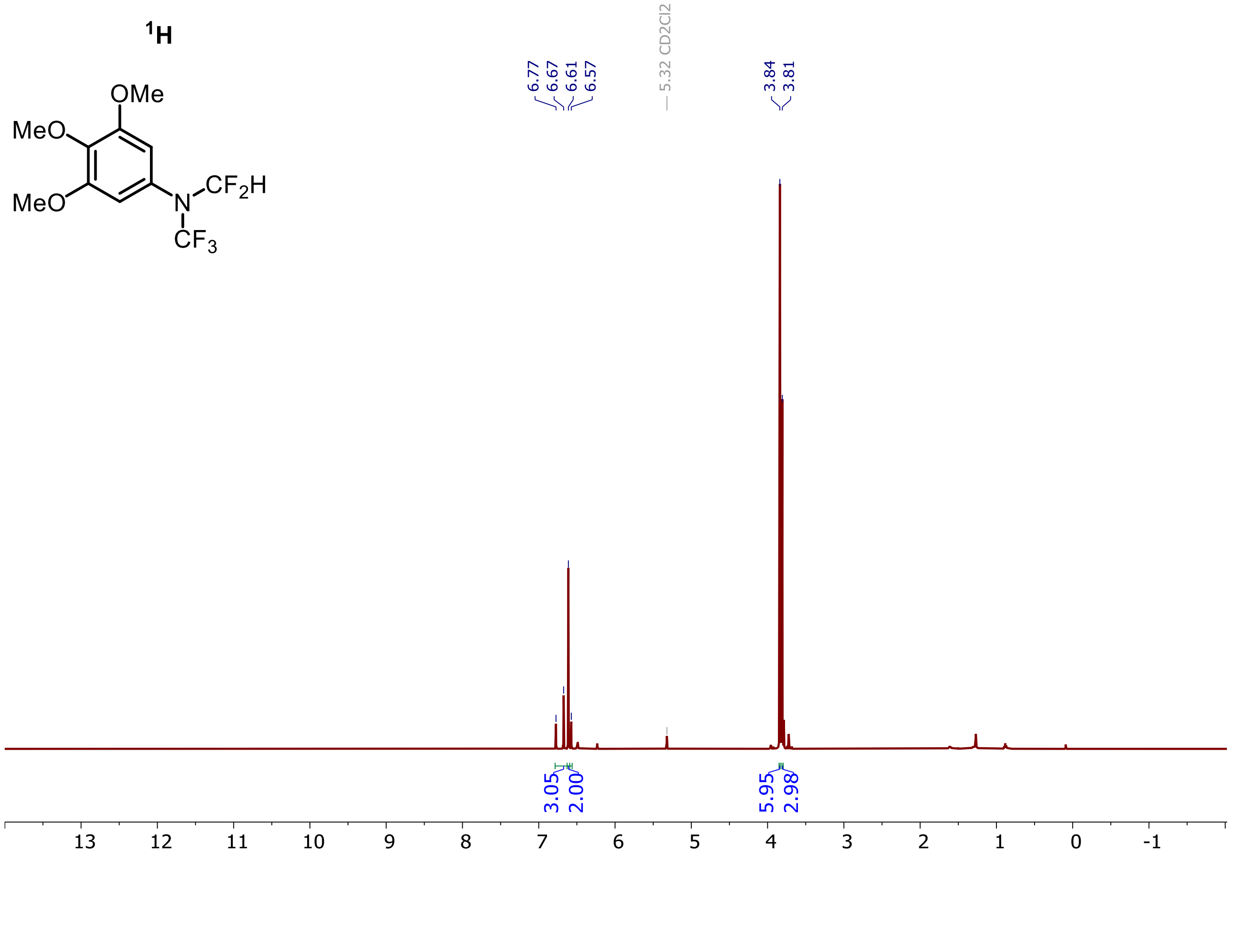

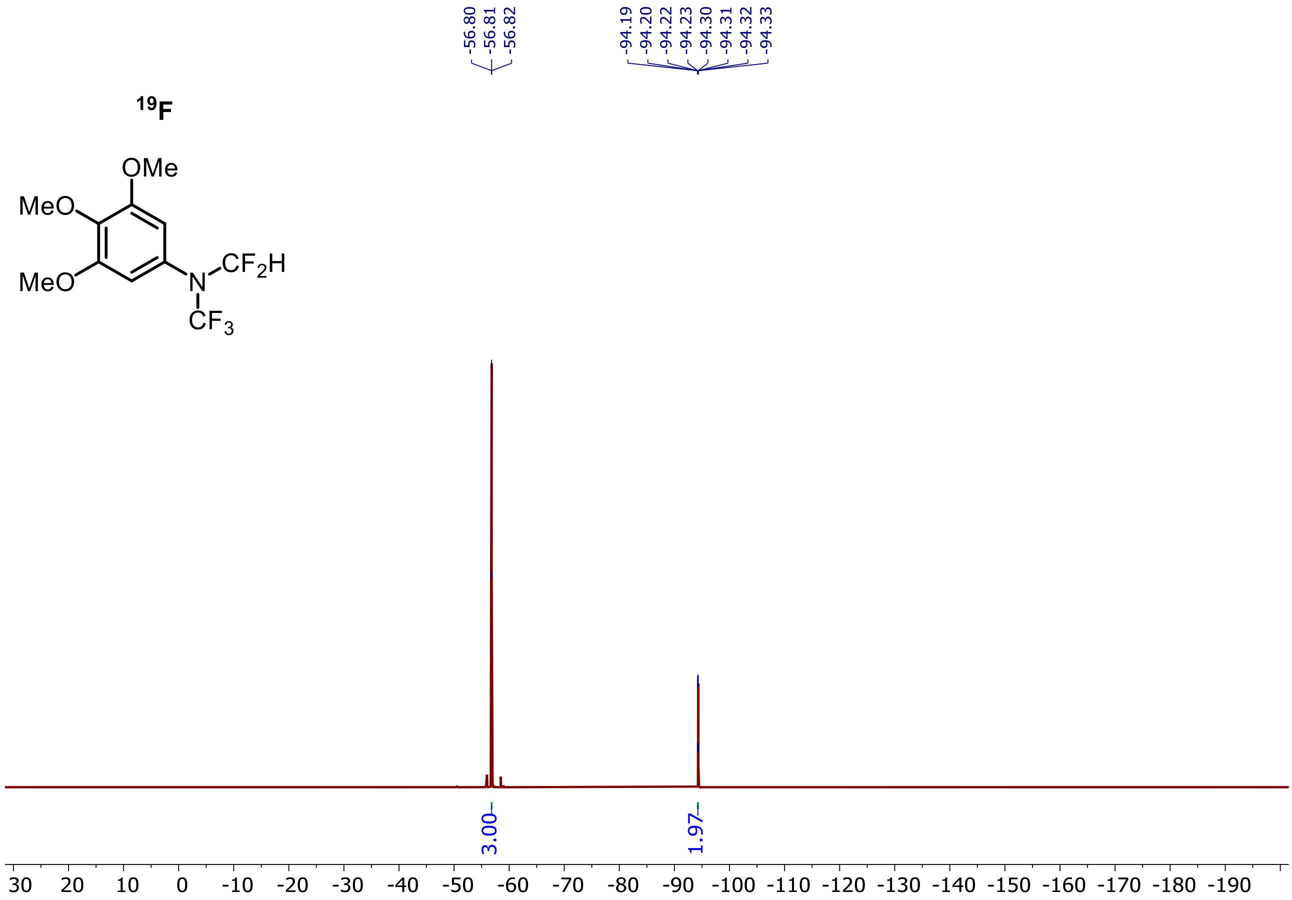
19F
OMe
MeO
MeO
N
CF2H
CF3
-56.80
-56.81
-56.82
-94.19
-94.20
-94.22
-94.23
-94.30
-94.31
-94.32
-94.33
3.00
1.97
30 20 10 0 -10 -20 -30 -40 -50 -60 -70 -80 -90 -100 -110 -120 -130 -140 -150 -160 -170 -180 -190

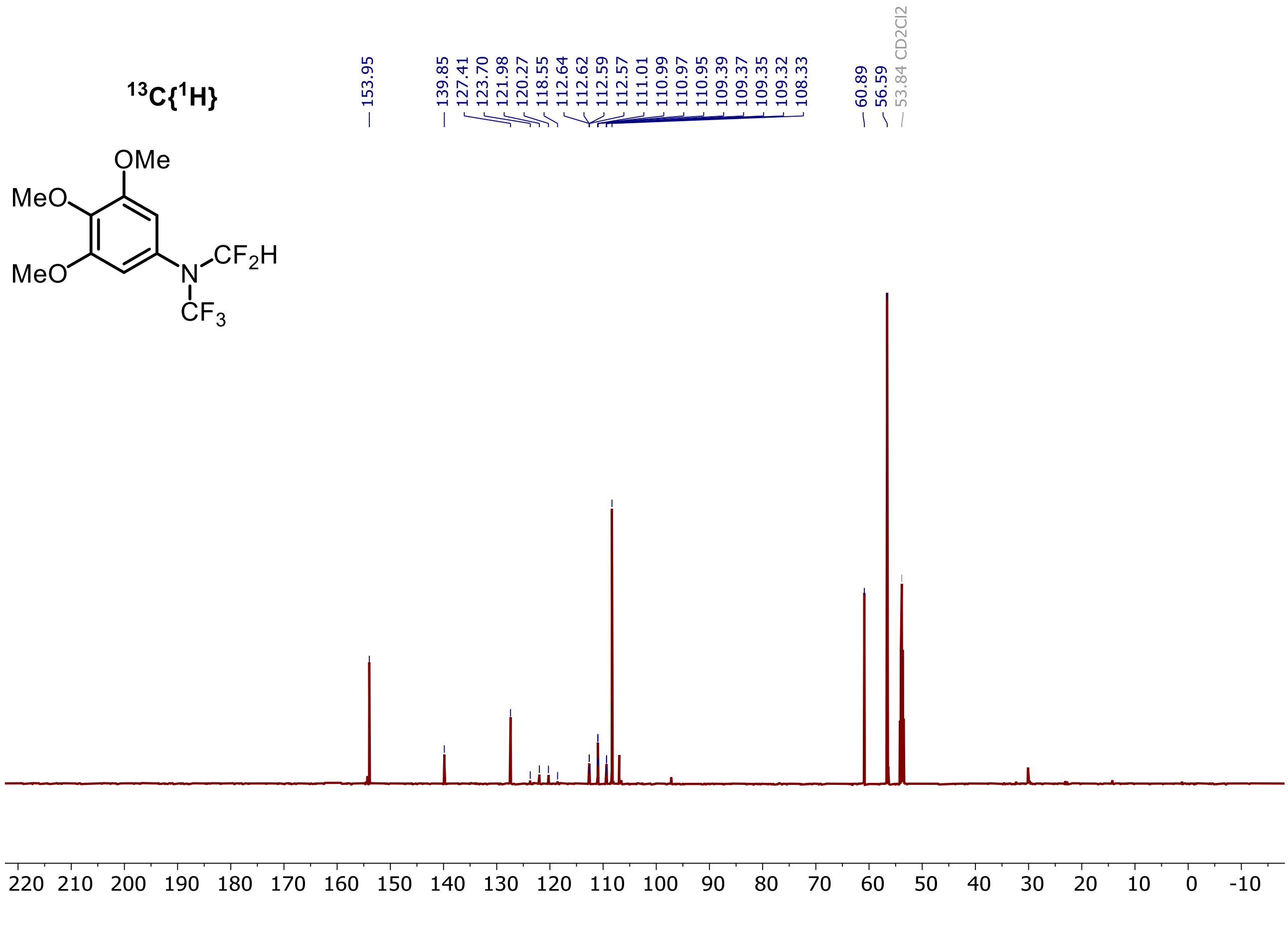
13C{1H}
OMe
MeO
MeO
N
CF2H
CF3
153.95
139.85
127.41
123.70
121.98
120.27
118.55
112.64
112.62
112.59
112.57
111.01
110.99
110.97
110.95
109.39
109.37
109.35
109.32
108.33
60.89
56.59
53.84 CD2Cl2
220 210 200 190 180 170 160 150 140 130 120 110 100 90 80 70 60 50 40 30 20 10 0 -10

4-bromo-2-chloro-*N*-(difluoromethyl)-*N*-(trifluoromethyl)aniline (5)

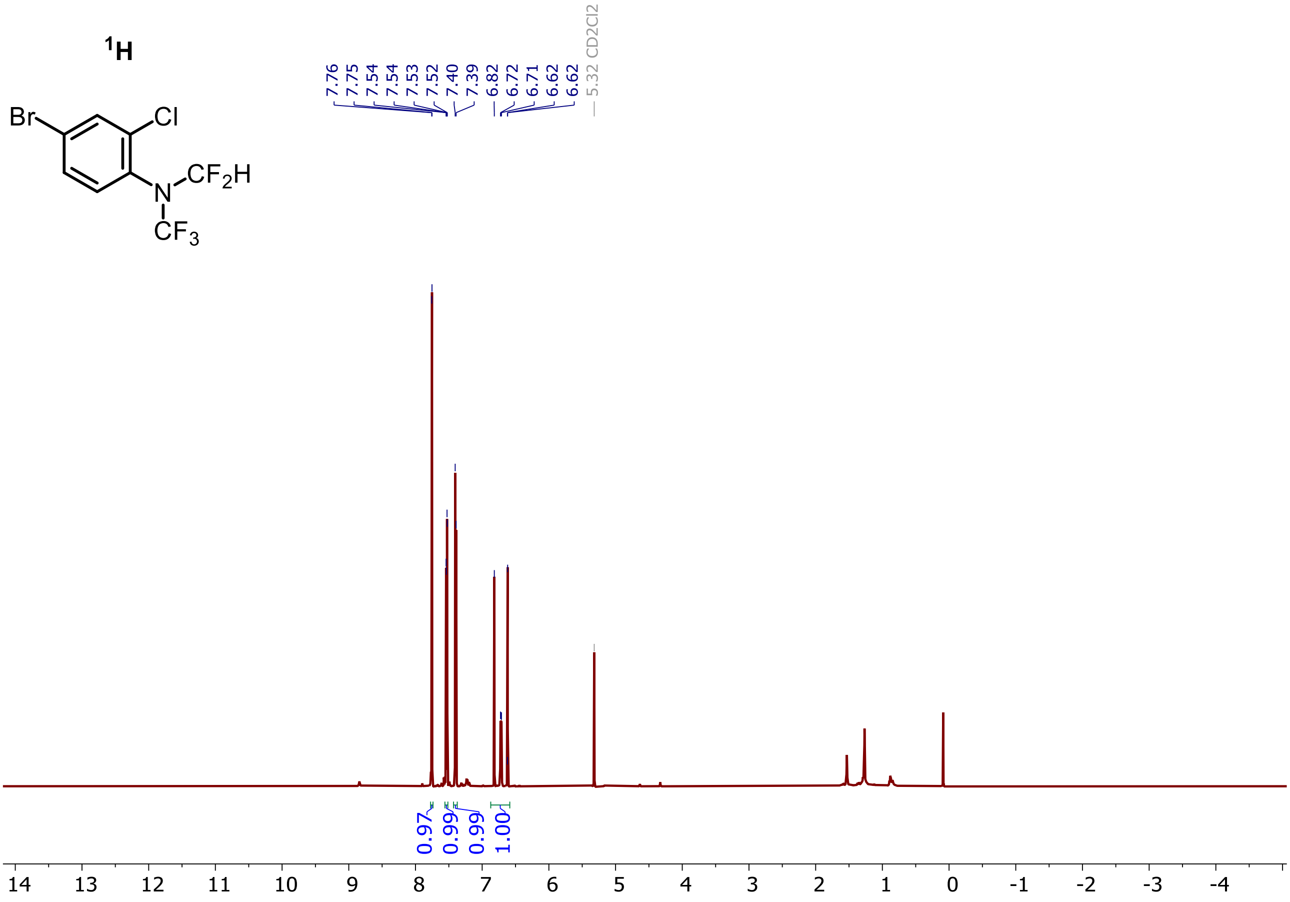

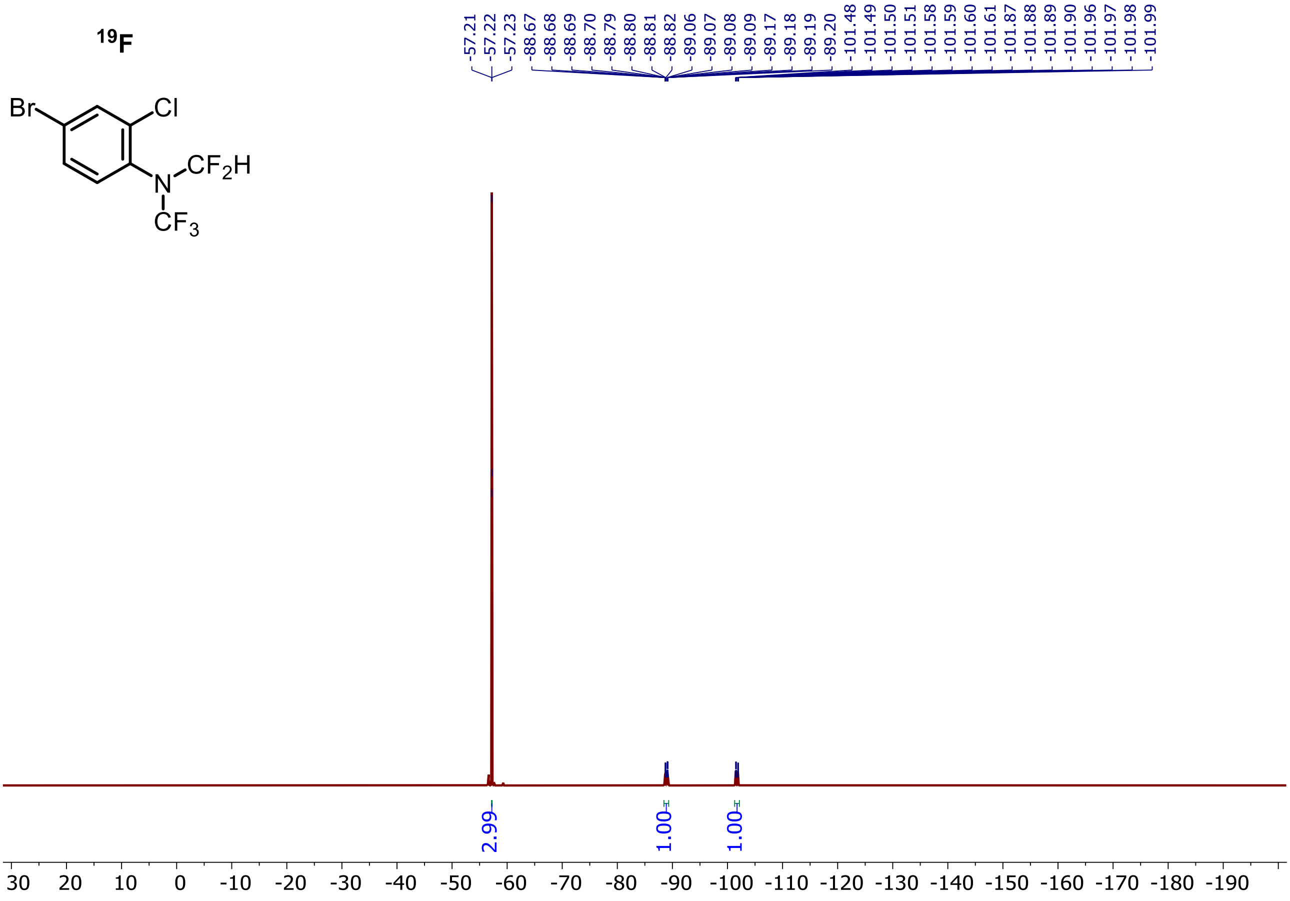

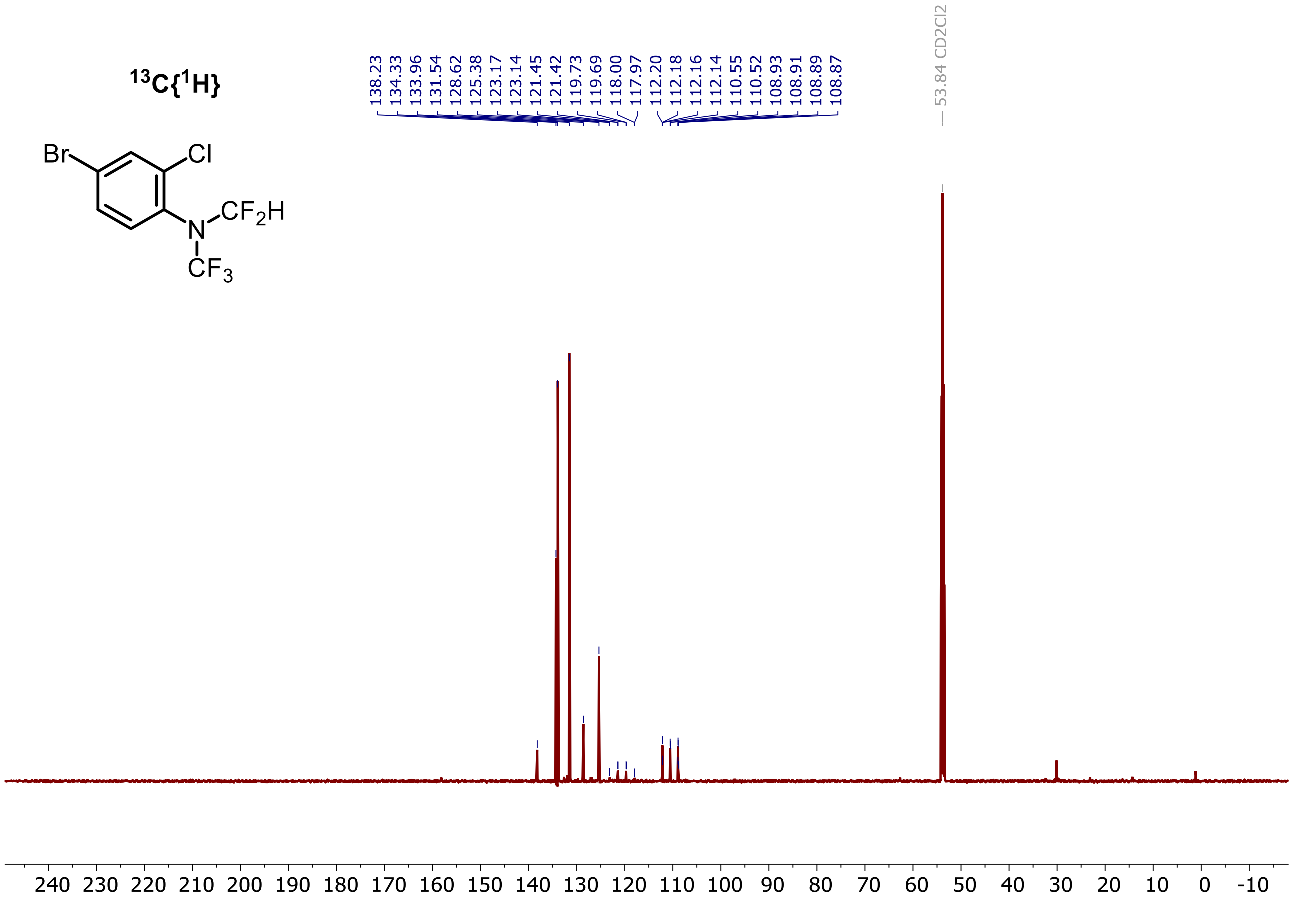

methyl 4-((difluoromethyl)(trifluoromethyl)amino)benzoate (6)

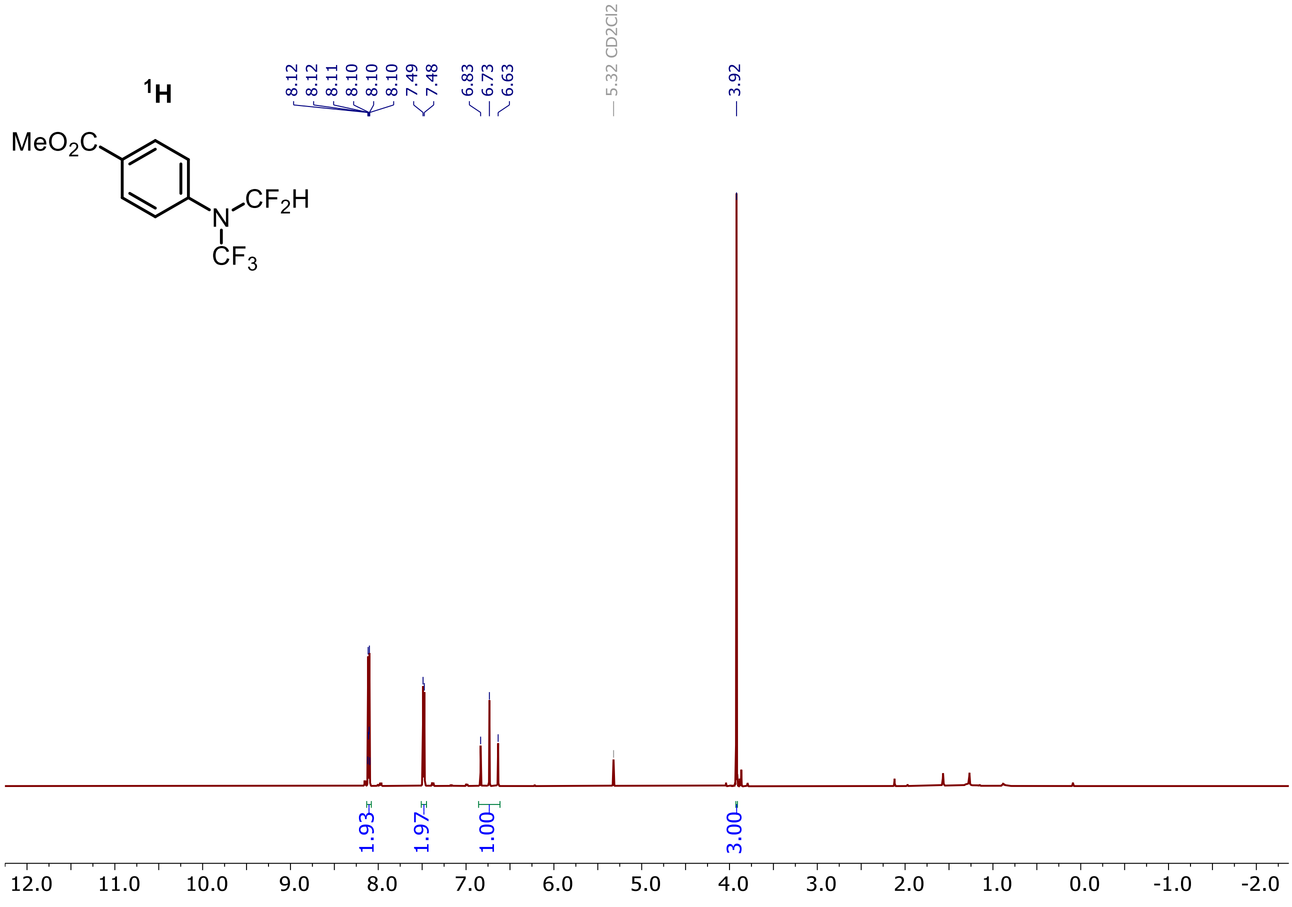

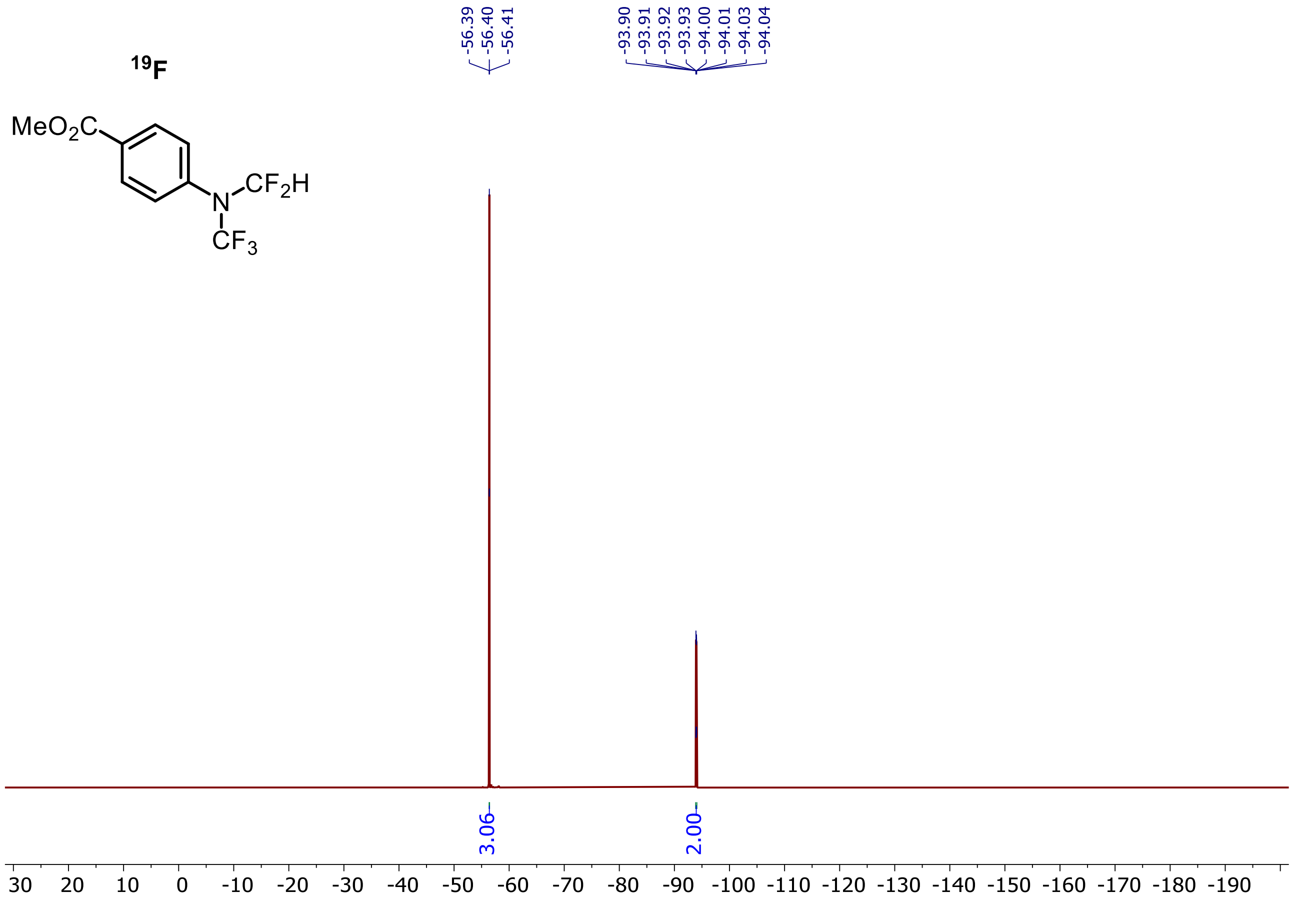
19F
MeO2C
CF2H
N
CF3
-56.39
-56.40
-56.41
-93.90
-93.91
-93.92
-93.93
-94.00
-94.01
-94.03
-94.04
3.06
2.00
30 20 10 0 -10 -20 -30 -40 -50 -60 -70 -80 -90 -100 -110 -120 -130 -140 -150 -160 -170 -180 -190

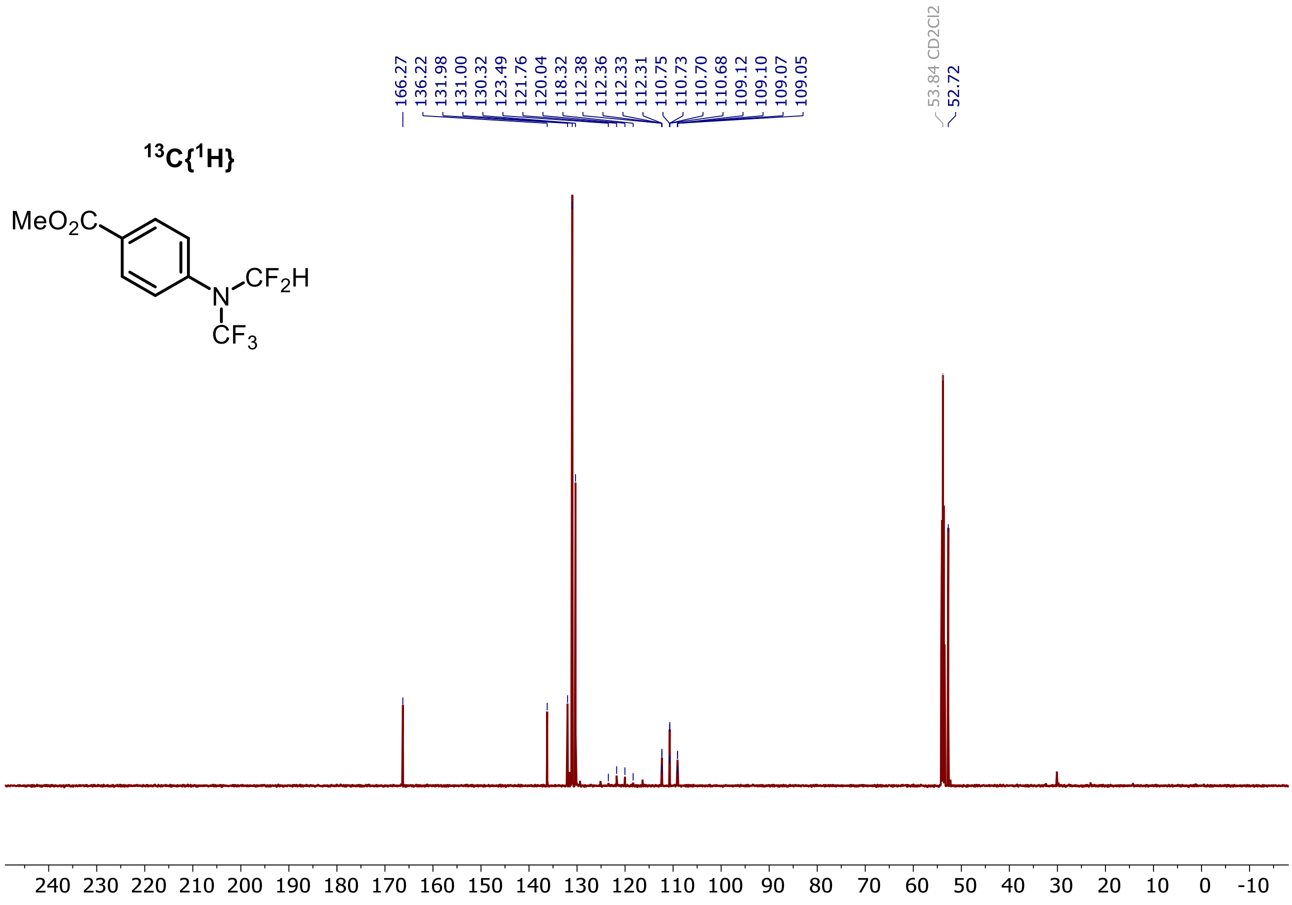
13C{1H}
MeO2C
CF2H
N
CF3
166.27
136.22
131.98
131.00
130.32
123.49
121.76
120.04
118.32
112.38
112.36
112.33
112.31
110.75
110.73
110.70
110.68
109.12
109.10
109.07
109.05
53.84 CD2Cl2
52.72
240 230 220 210 200 190 180 170 160 150 140 130 120 110 100 90 80 70 60 50 40 30 20 10 0 -10

***N*-(difluoromethyl-d)-*N*-(trifluoromethyl)-[1,1'-biphenyl]-4-amine (7)**

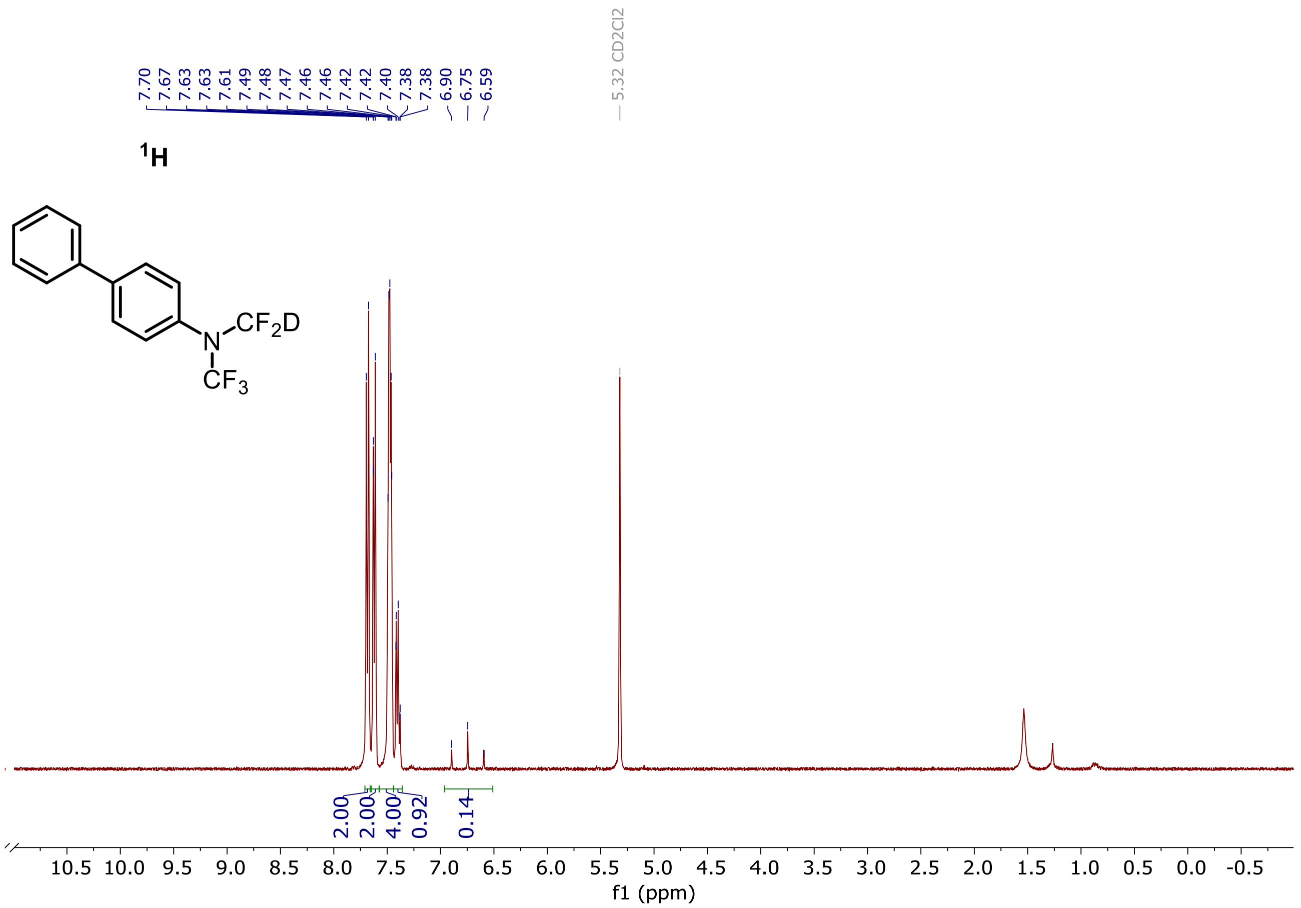

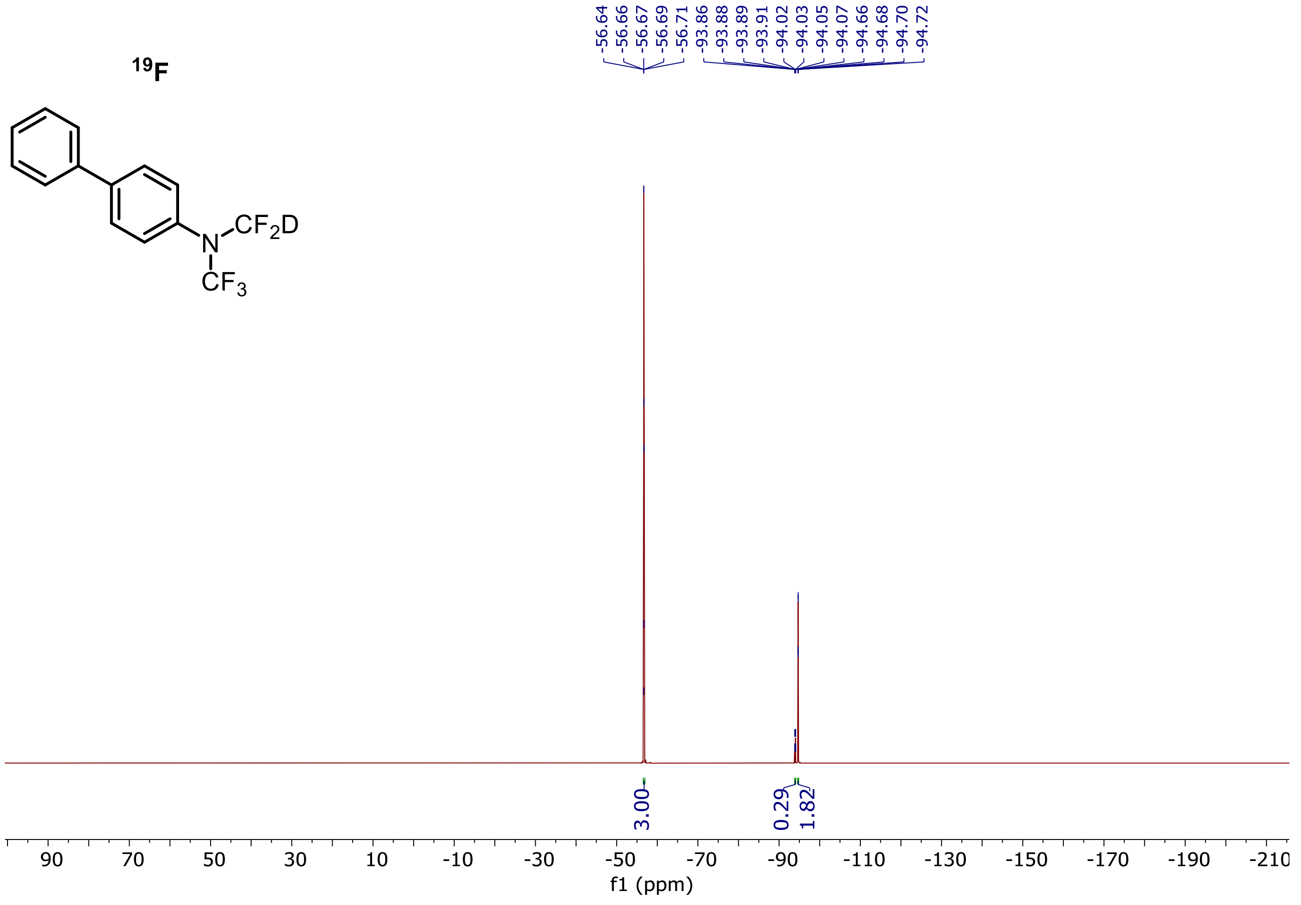

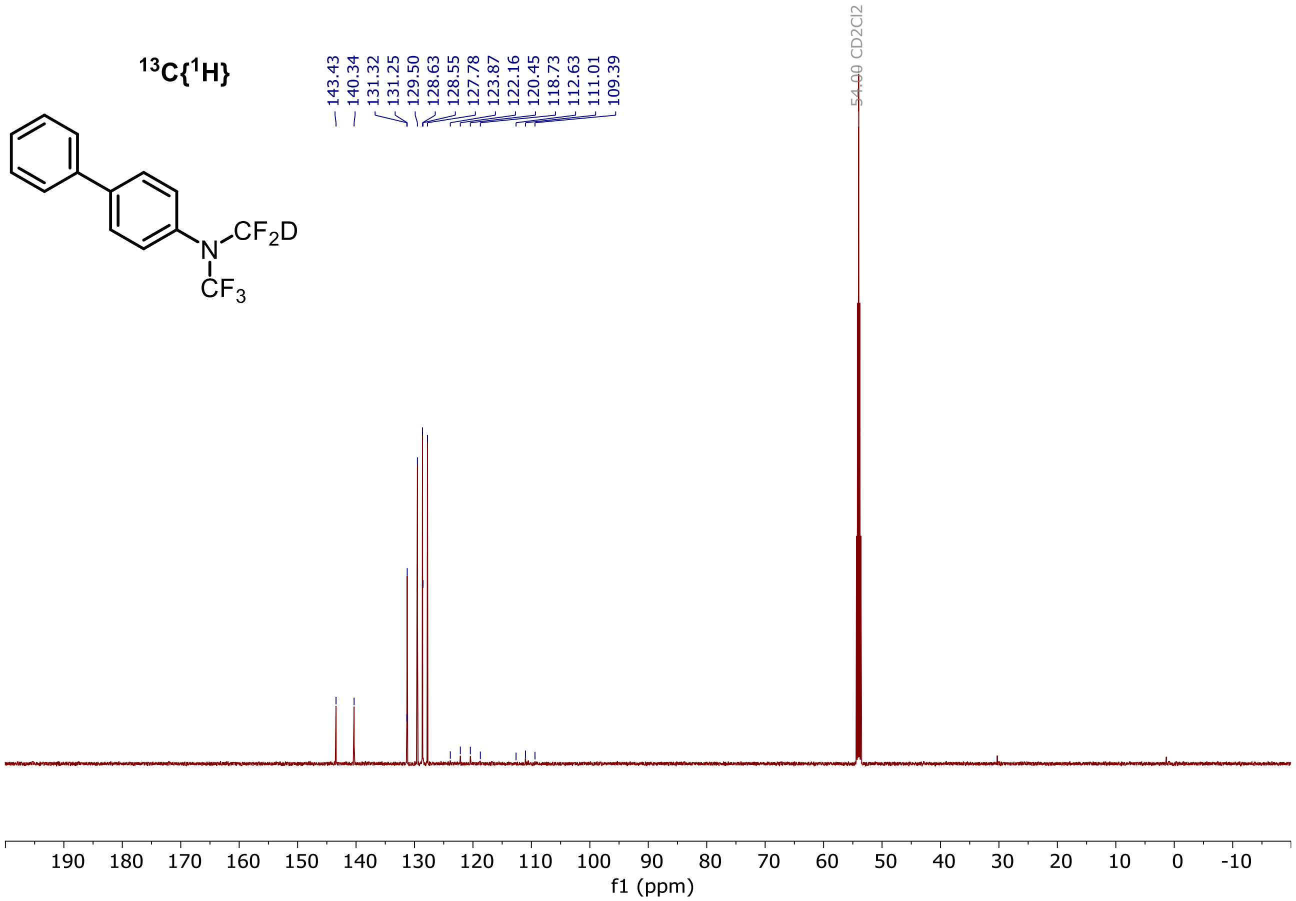

tert-butyl 4-((difluoromethyl)(trifluoromethyl)amino)piperidine-1-carboxylate (8)

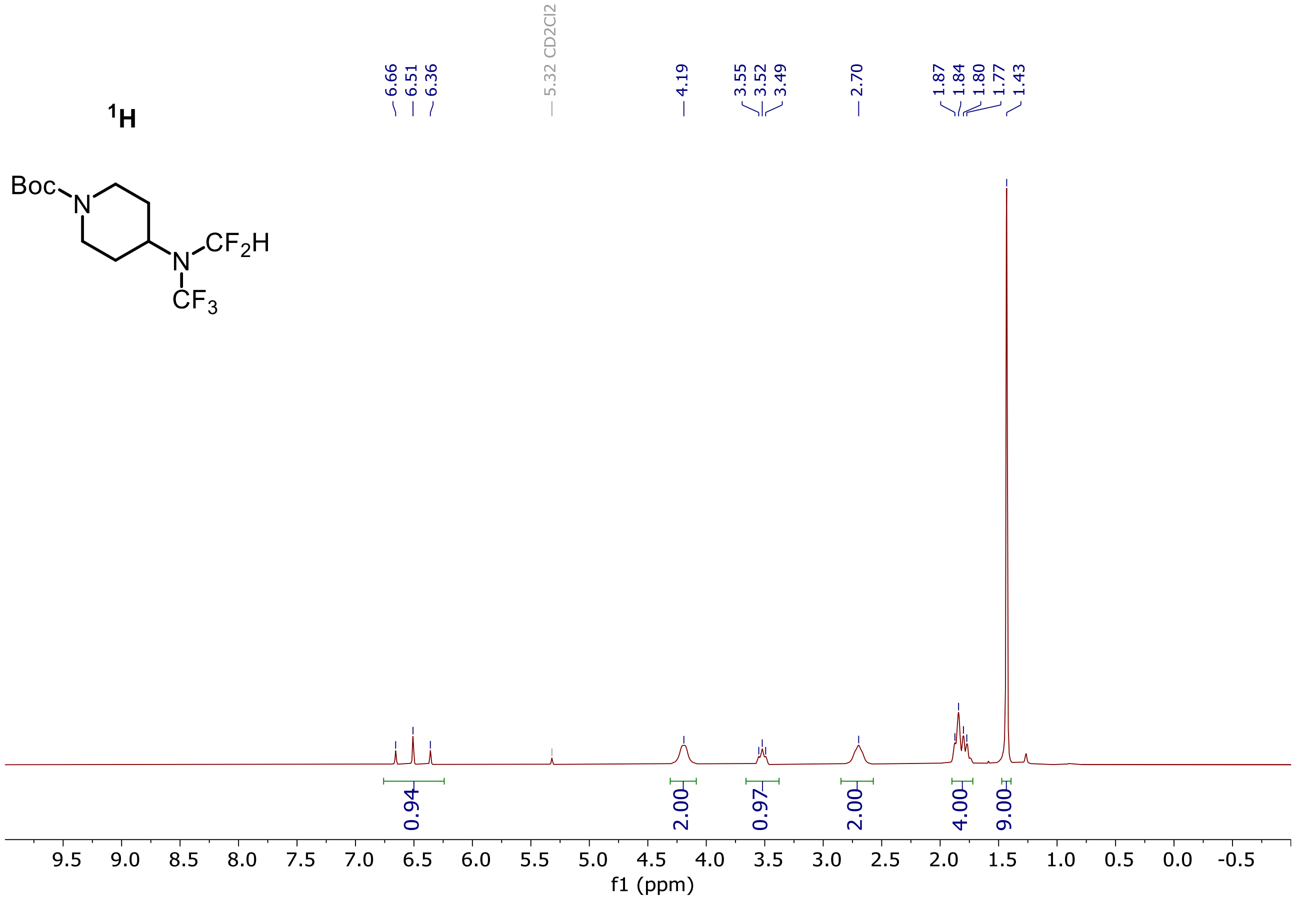

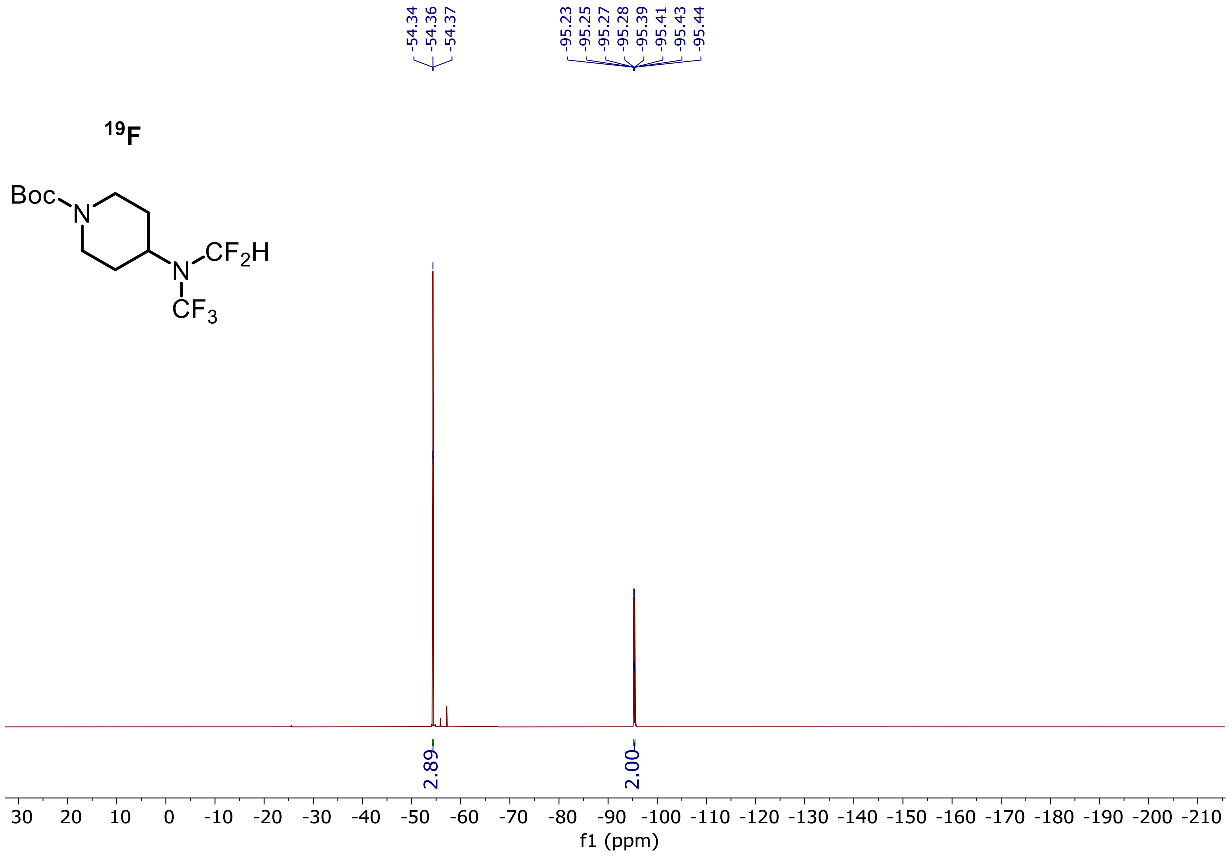
-54.34
-54.36
-54.37
-95.23
-95.25
-95.27
-95.28
-95.39
-95.41
-95.43
-95.44
19F
Boc
N
N
CF2H
CF3
2.89
2.00
30 20 10 0 -10 -20 -30 -40 -50 -60 -70 -80 -90 -100 -110 -120 -130 -140 -150 -160 -170 -180 -190 -200 -210
f1 (ppm)

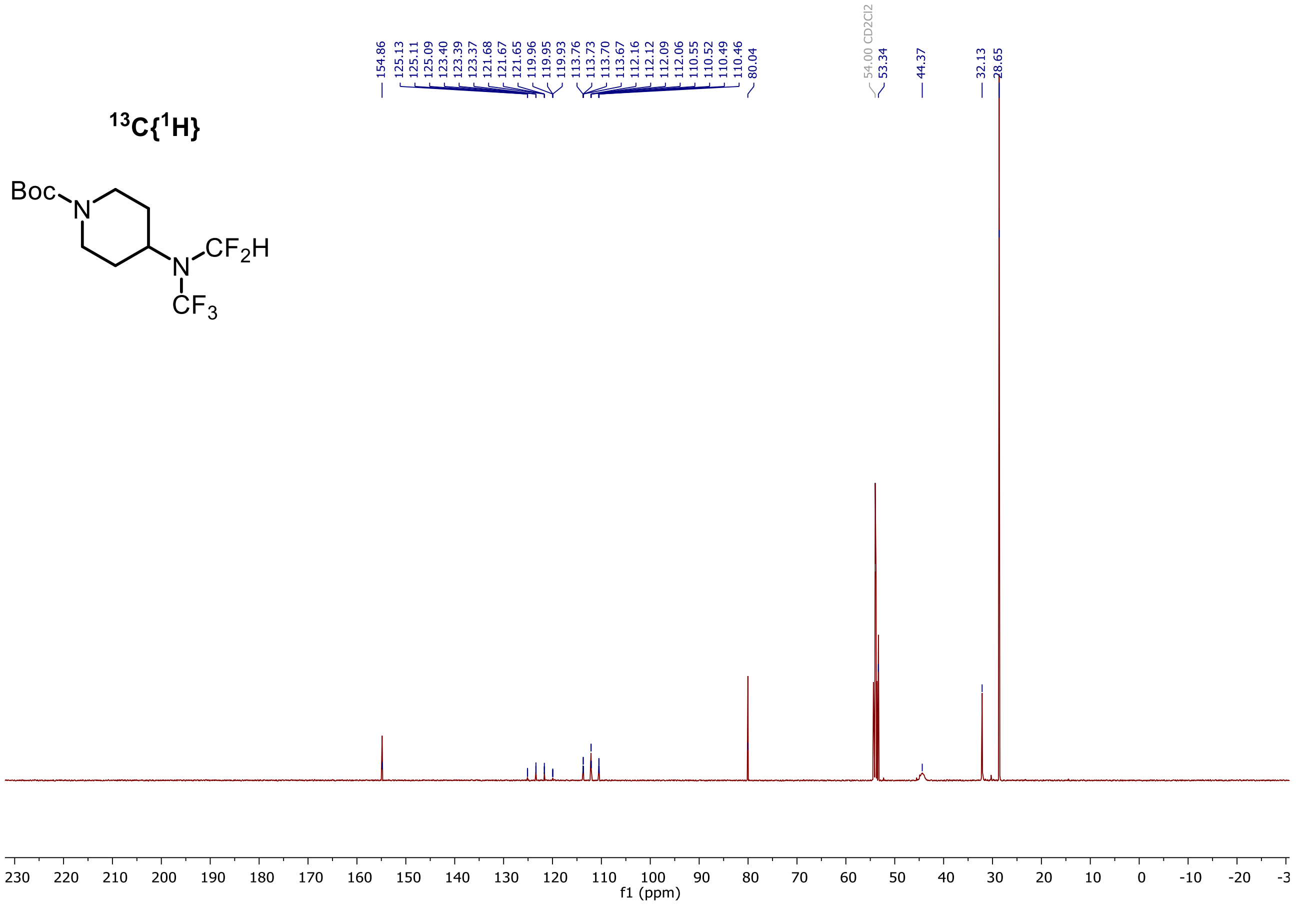
154.86
125.13
125.11
125.09
123.40
123.39
123.37
121.68
121.67
121.65
119.96
119.95
119.93
113.76
113.73
113.70
113.67
112.16
112.12
112.09
112.06
110.55
110.52
110.49
110.46
80.04
54.00 CD2Cl2
53.34
44.37
32.13
28.65
13C{1H}
Boc
N
N
CF2H
CF3
230 220 210 200 190 180 170 160 150 140 130 120 110 100 90 80 70 60 50 40 30 20 10 0 -10 -20 -3
f1 (ppm)

tert-butyl _N_-(difluoromethyl)-_N_-(trifluoromethyl)-_L_-phenylalaninate (9)

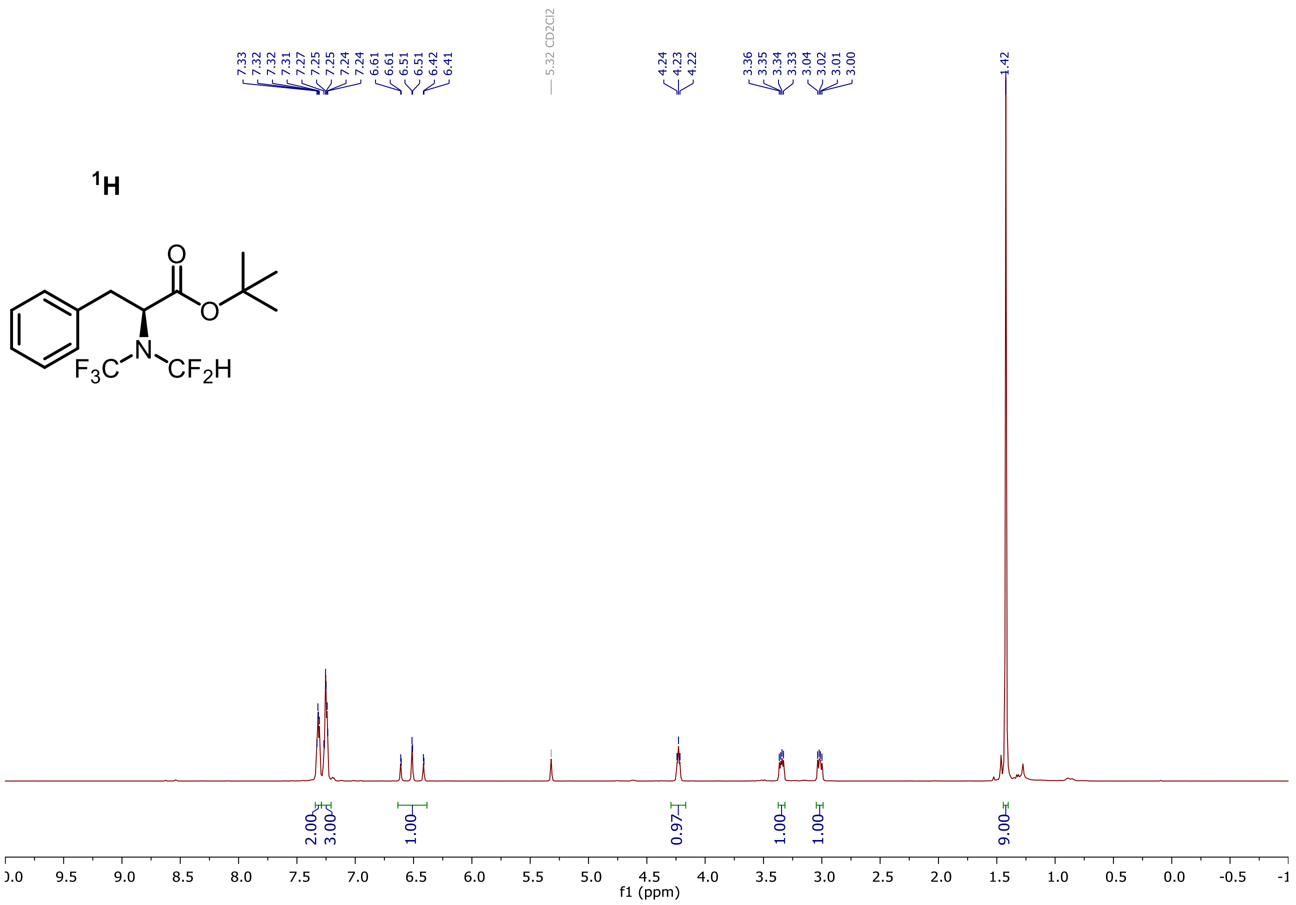

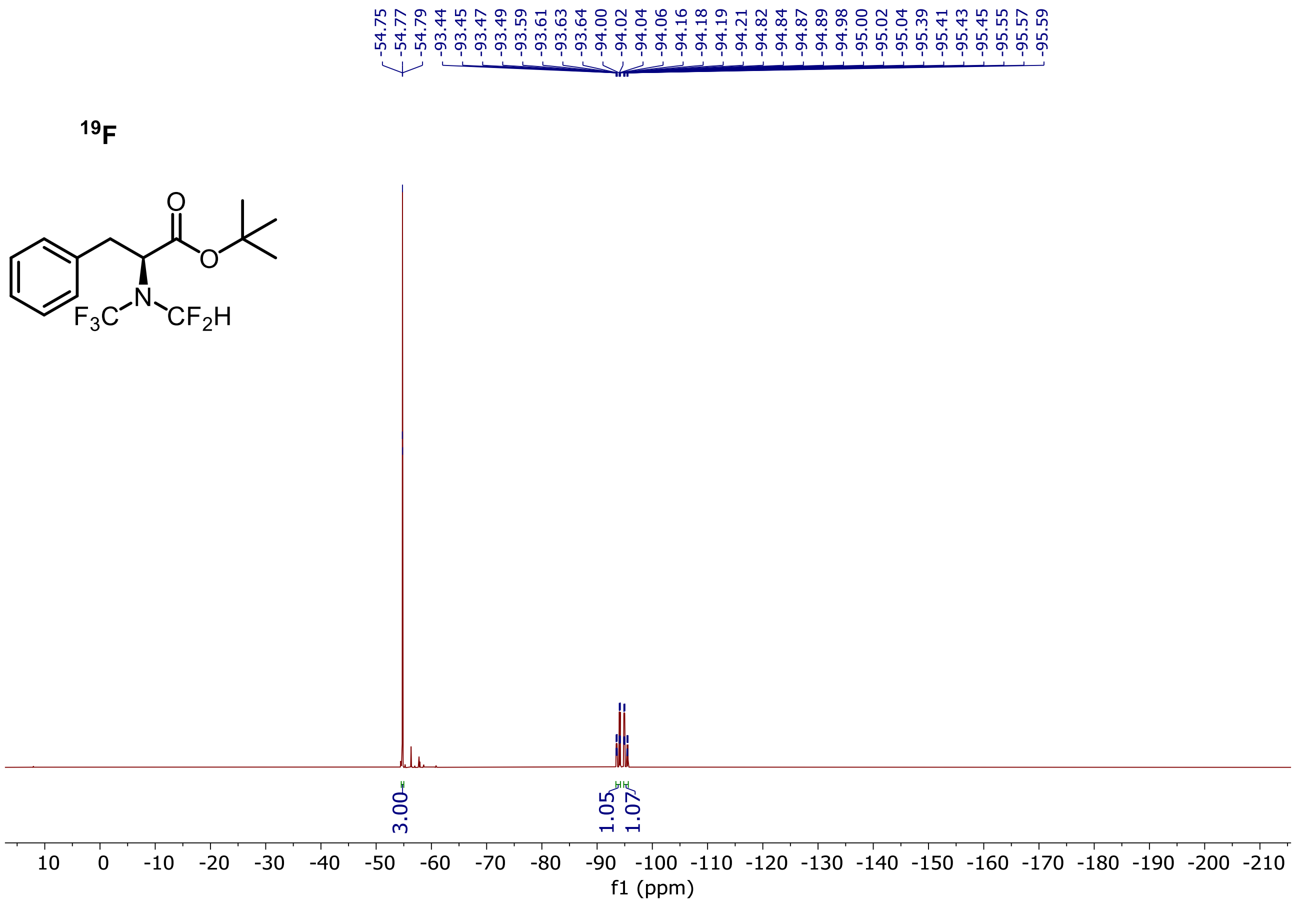

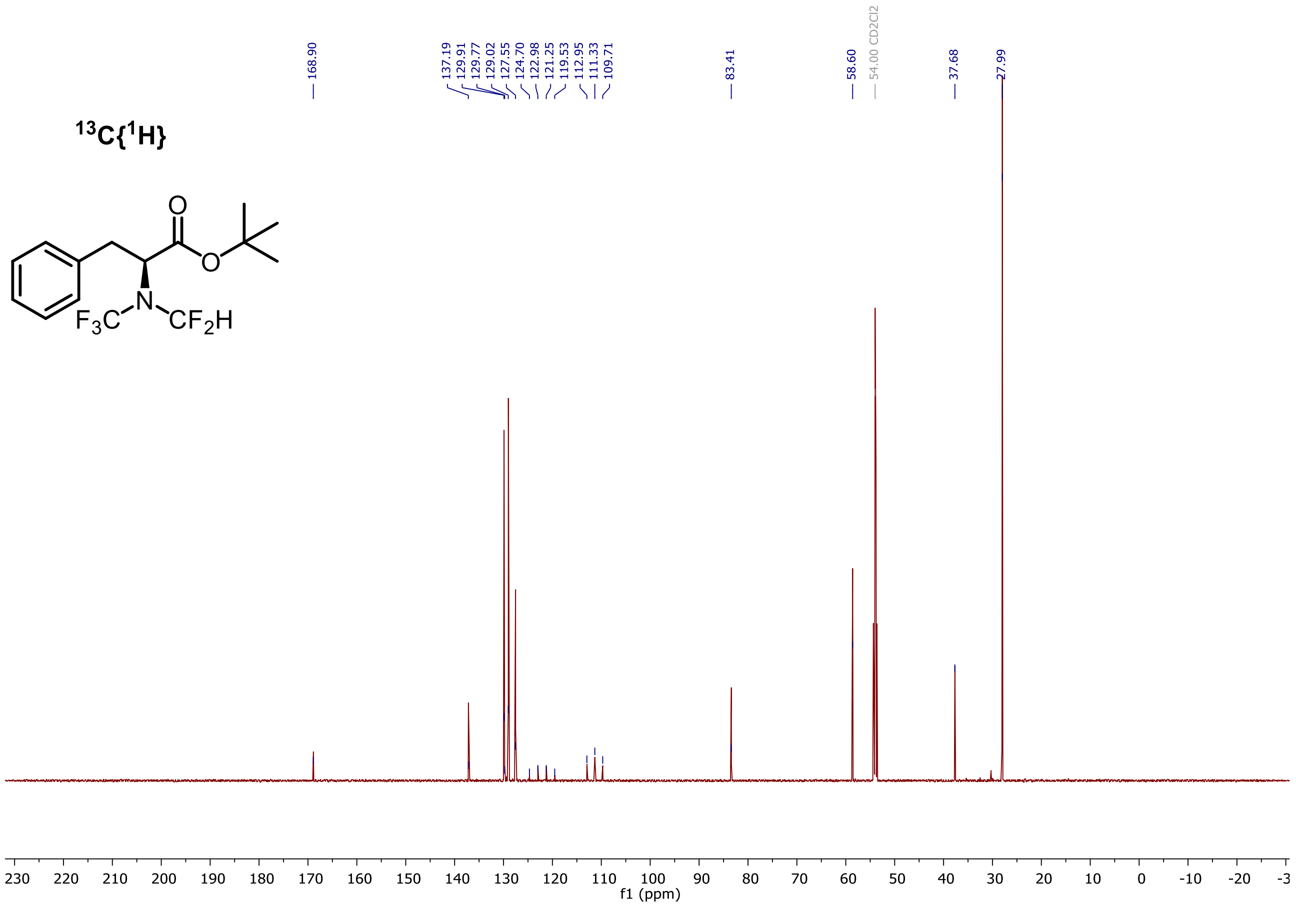

benzyl *N*-(difluoromethyl)-*N*-(trifluoromethyl)-*L*-leucinate (10)

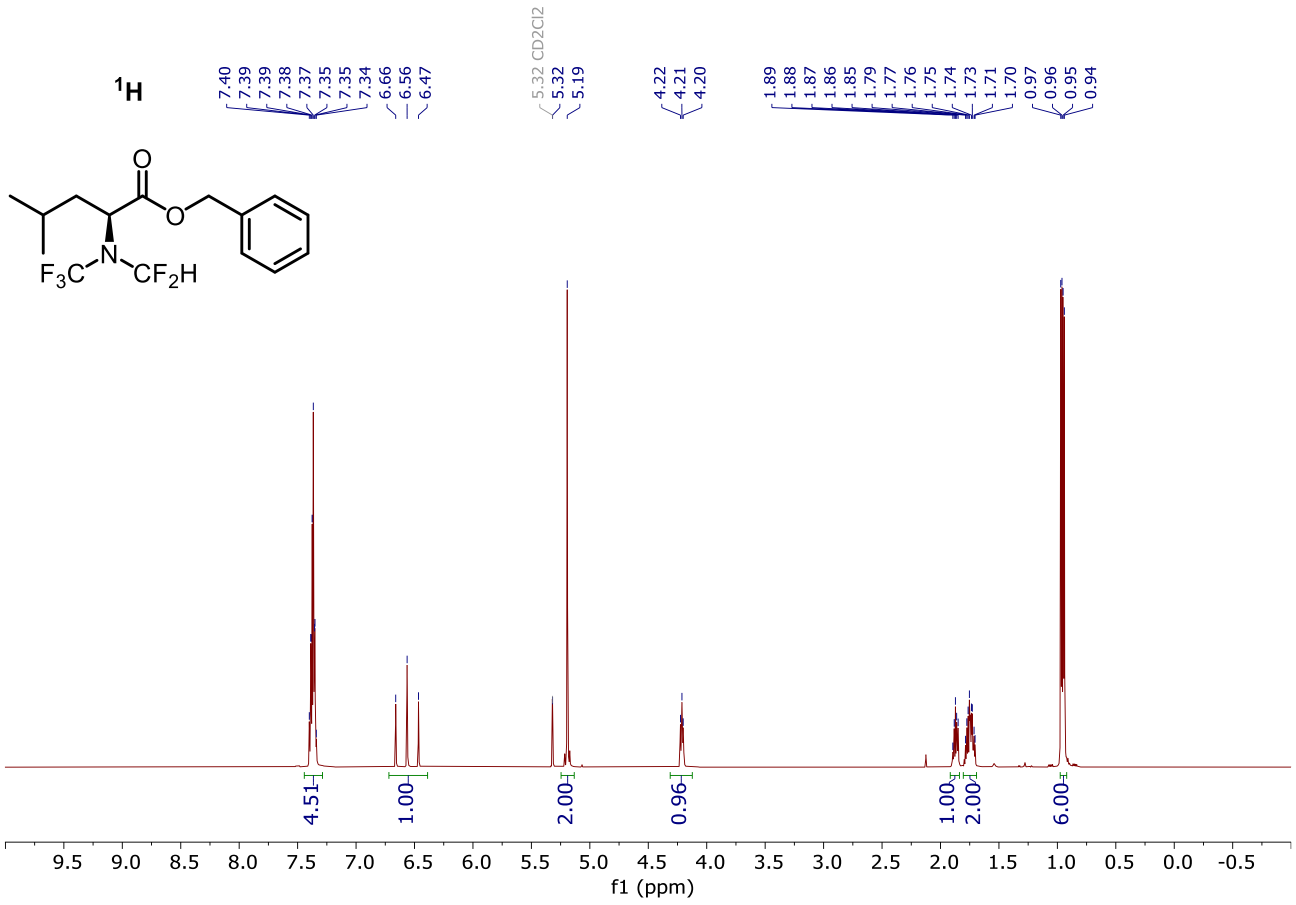

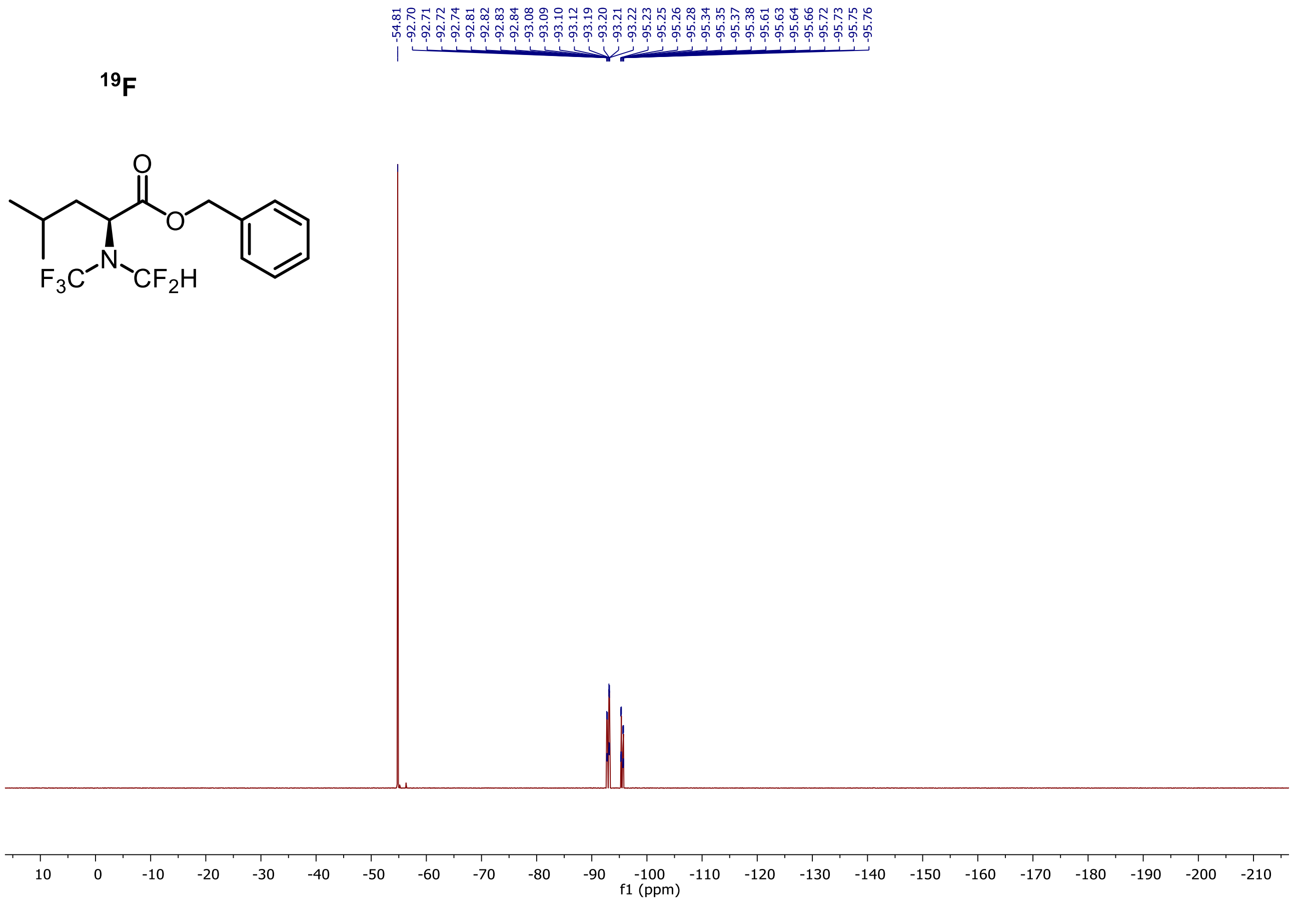
19F
F3C N CF2H
O
O
-54.81
-92.70
-92.71
-92.72
-92.74
-92.81
-92.82
-92.83
-92.84
-93.08
-93.09
-93.10
-93.12
-93.19
-93.20
-93.21
-93.22
-95.23
-95.25
-95.26
-95.28
-95.34
-95.35
-95.37
-95.38
-95.61
-95.63
-95.64
-95.66
-95.72
-95.73
-95.75
-95.76
f1 (ppm)

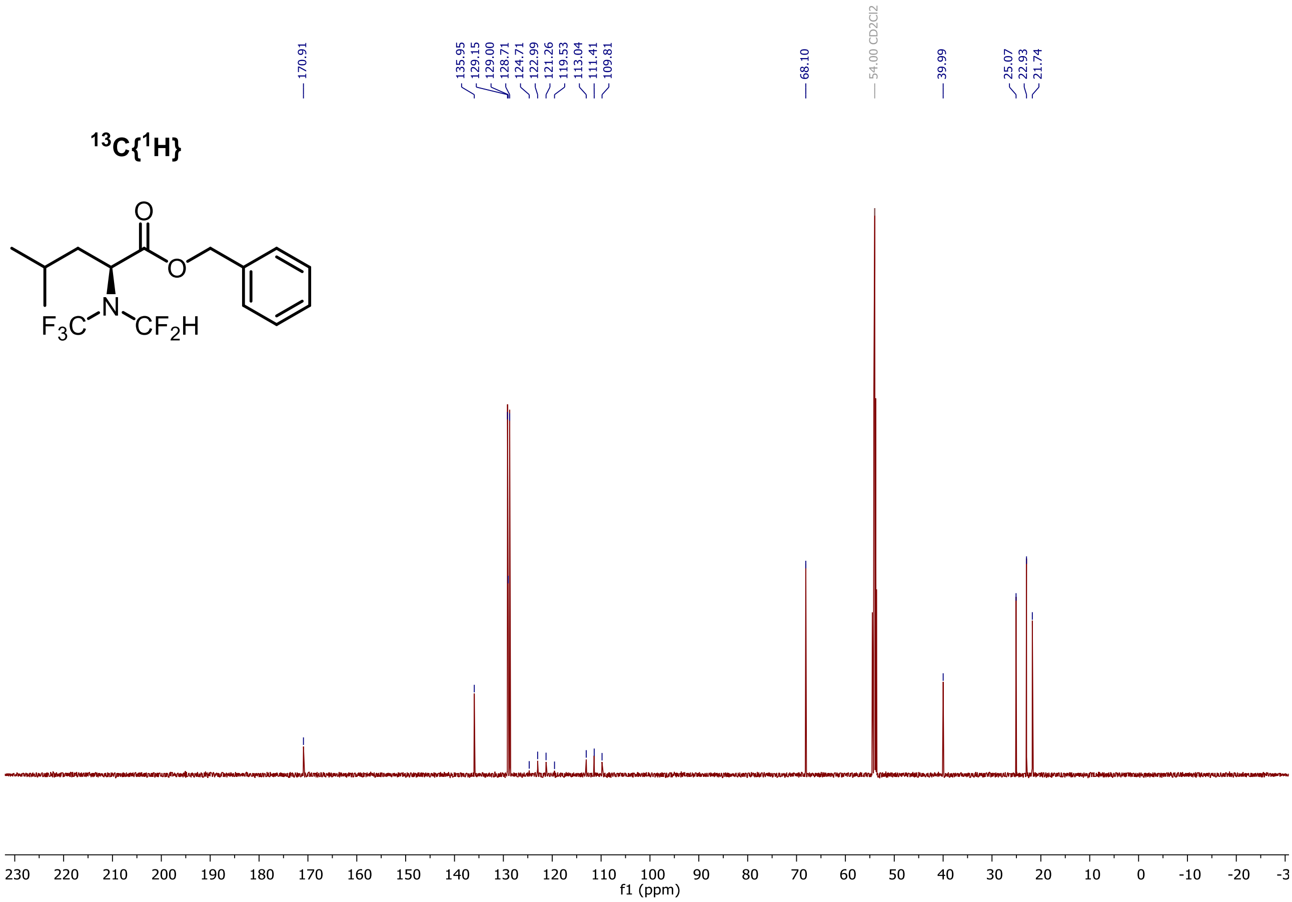
13C{1H}
F3C N CF2H
O
O
170.91
135.95
129.15
129.00
128.71
124.71
122.99
121.26
119.53
113.04
111.41
109.81
68.10
54.00 CD2Cl2
39.99
25.07
22.93
21.74
f1 (ppm)

N-(4-bromobenzyl)-N-(difluoromethyl)-1,1,1-trifluoromethanamine (11)

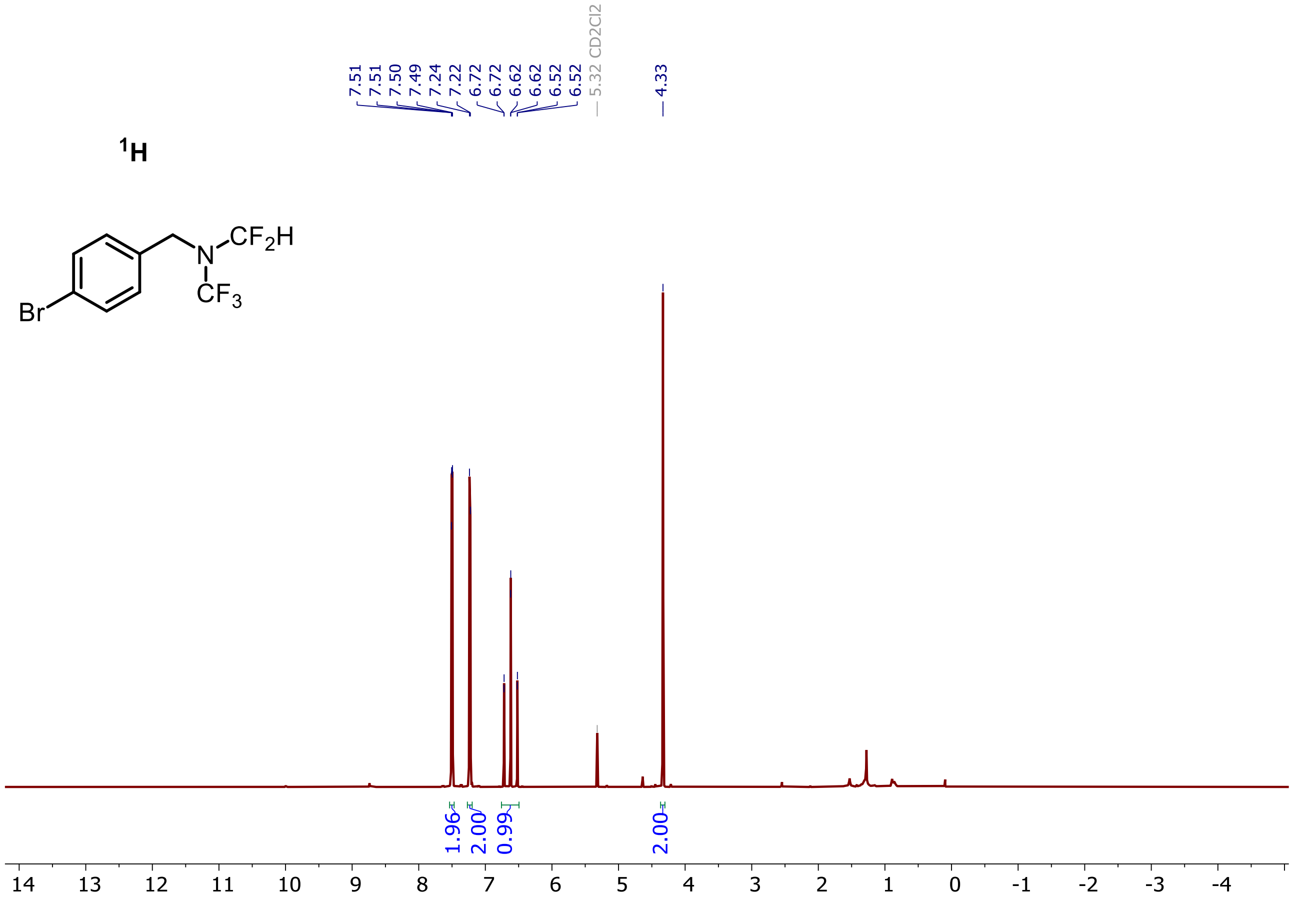

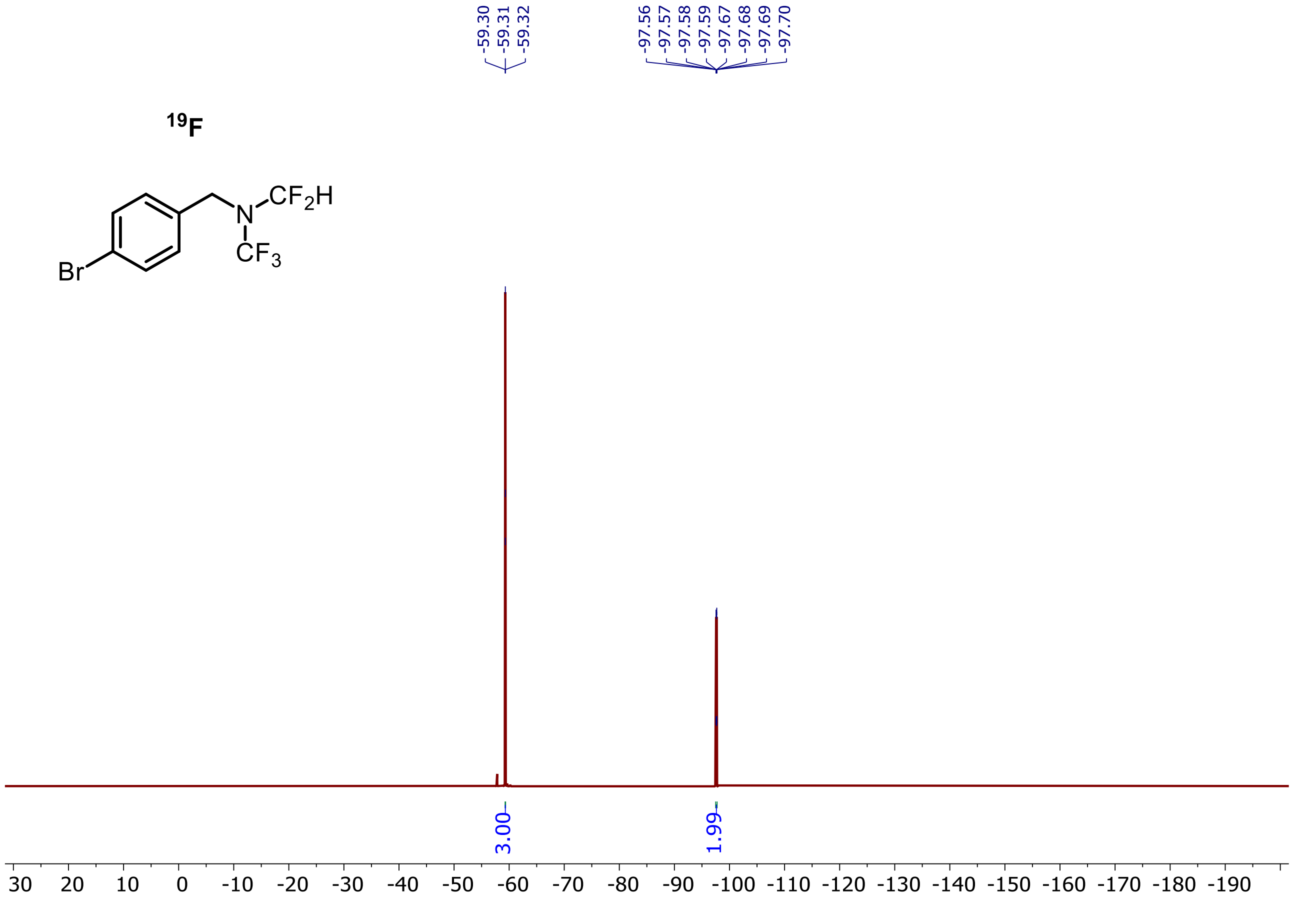

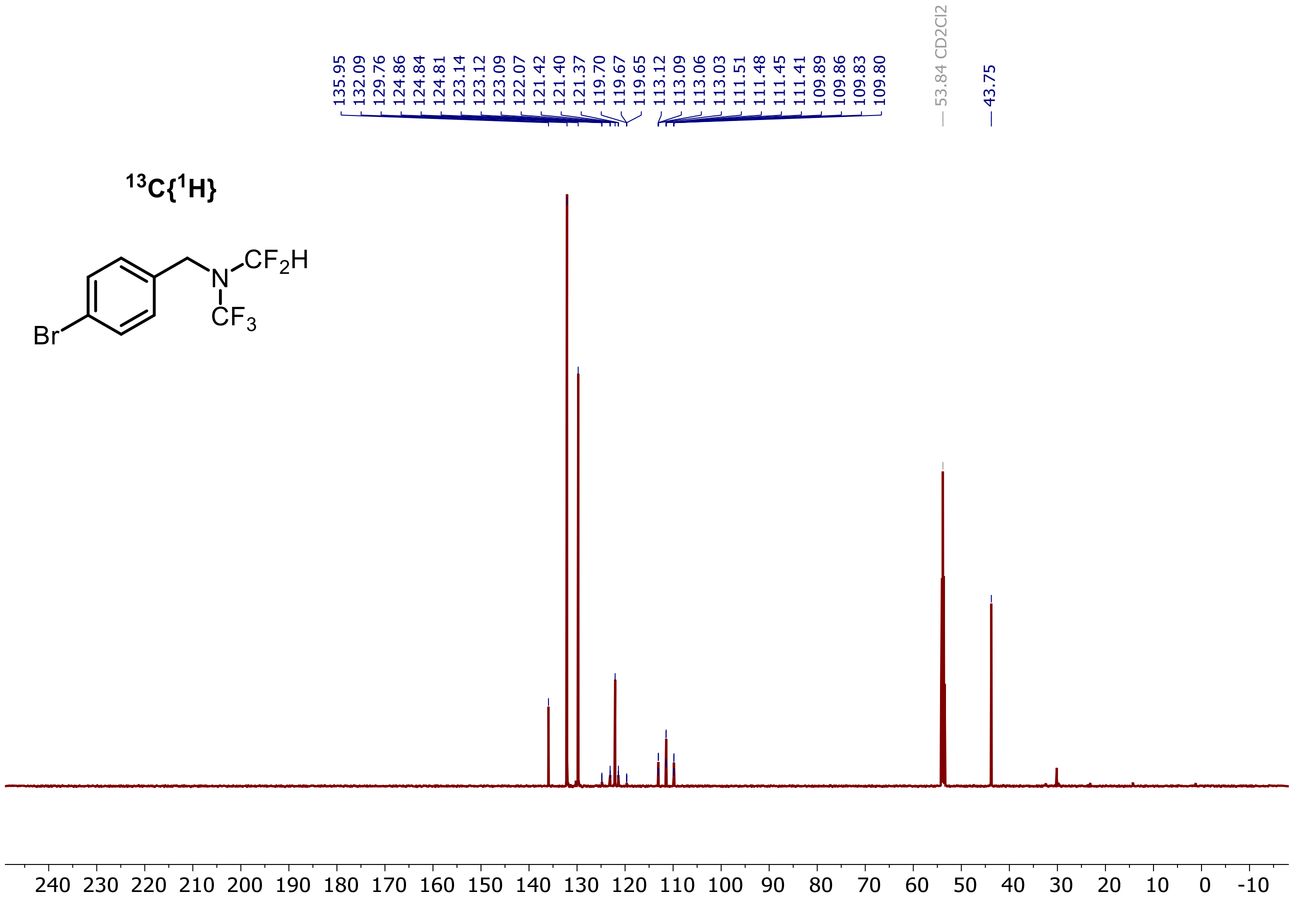

tert-butyl 4-((difluoromethyl-*d*)(trifluoromethyl)amino)piperidine-1-carboxylate (12)

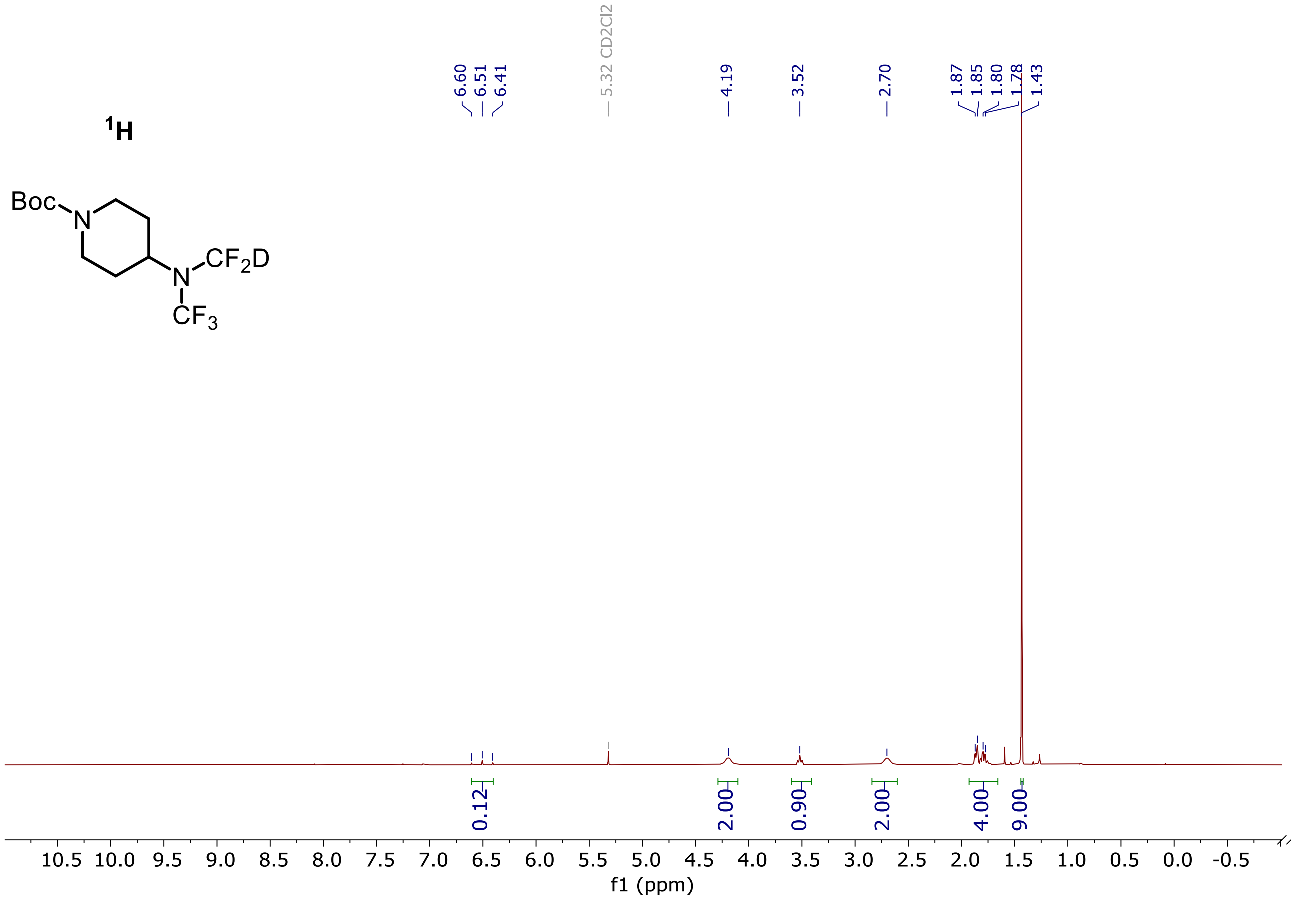

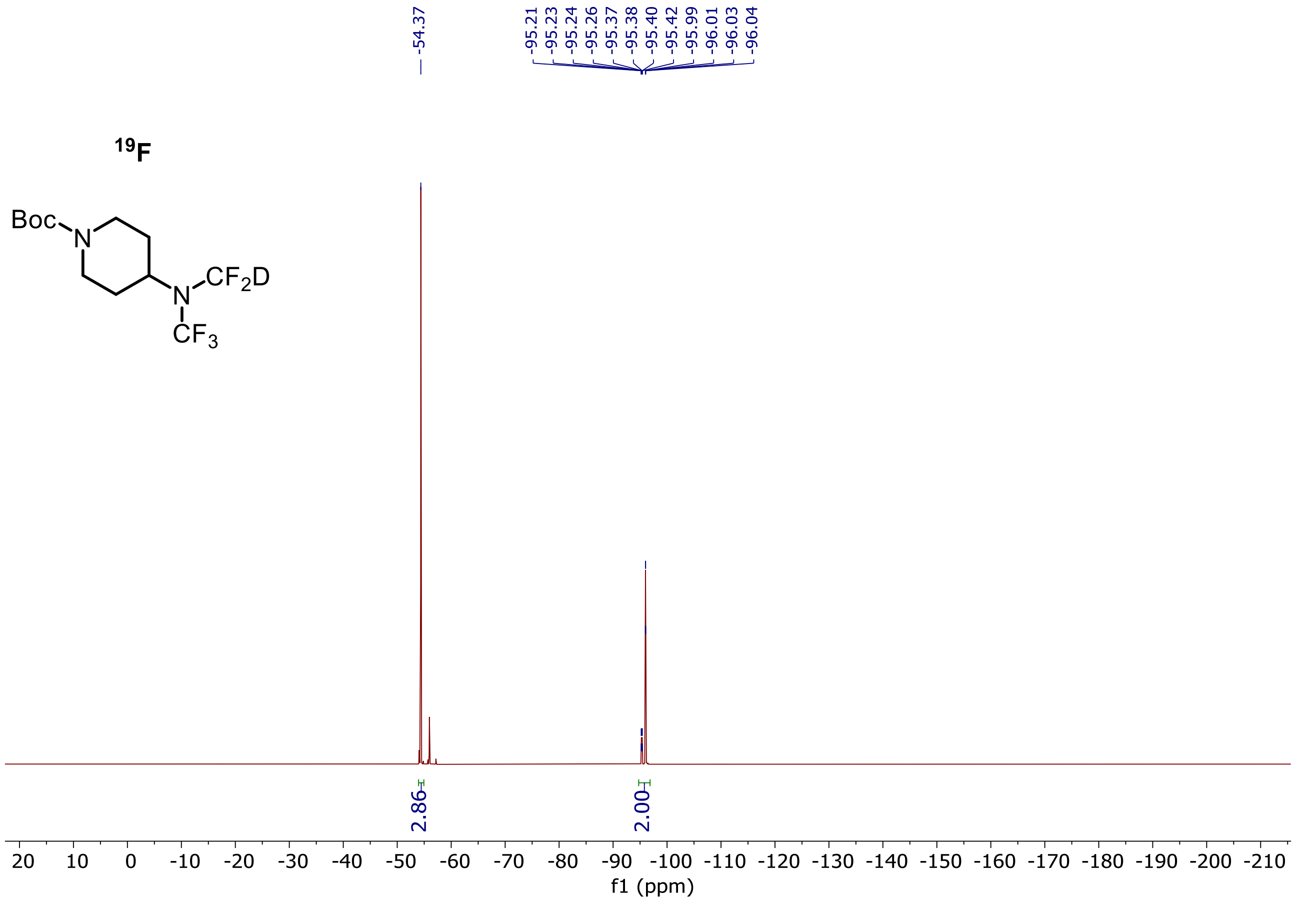
-54.37
-95.21
-95.23
-95.24
-95.26
-95.37
-95.38
-95.40
-95.42
-95.99
-96.01
-96.03
-96.04
19F
Boc
N
N
CF2D
CF3
2.86
2.00
20 10 0 -10 -20 -30 -40 -50 -60 -70 -80 -90 -100 -110 -120 -130 -140 -150 -160 -170 -180 -190 -200 -210
f1 (ppm)

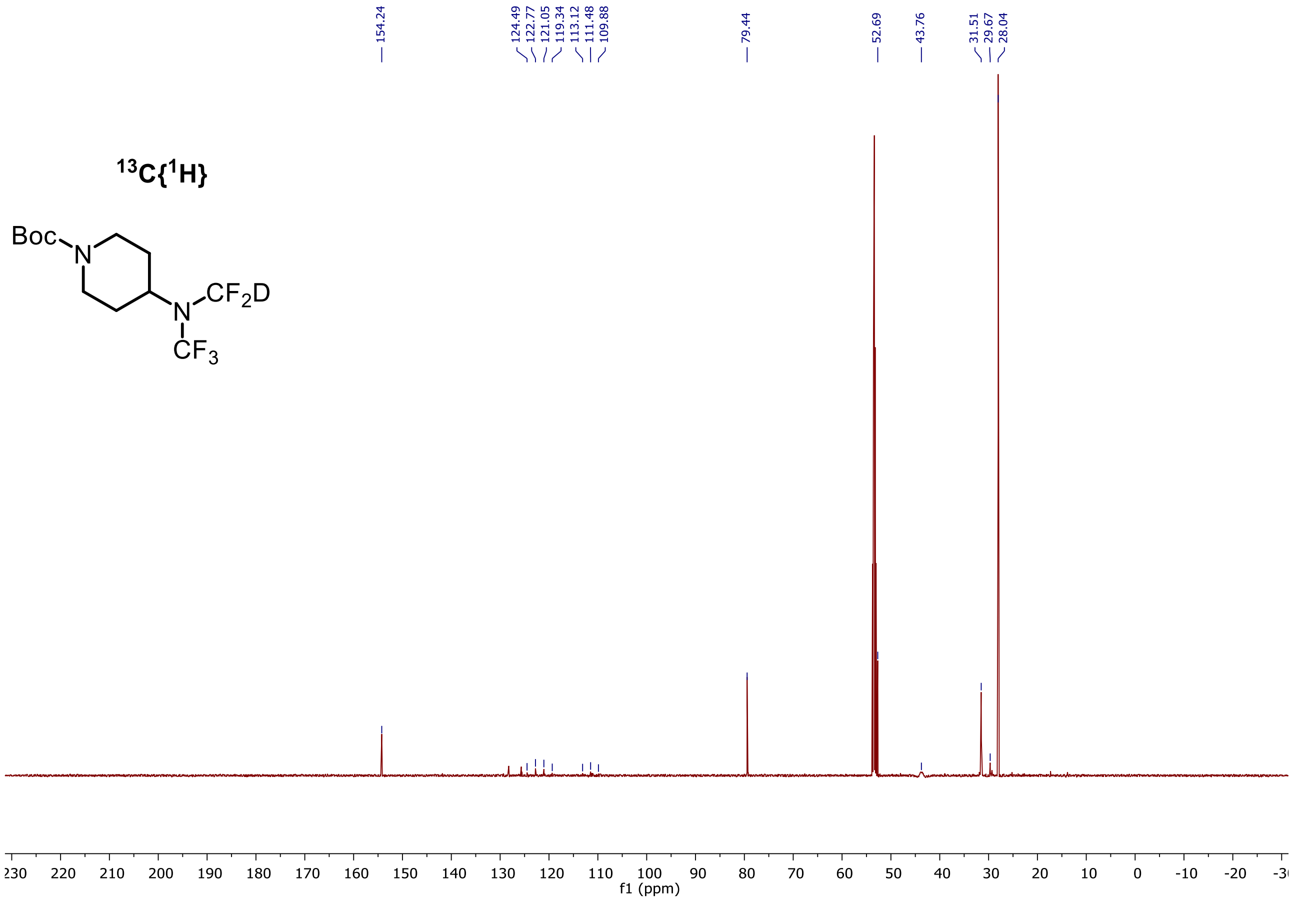
154.24
124.49
122.77
121.05
119.34
113.12
111.48
109.88
79.44
52.69
43.76
31.51
29.67
28.04
13C{1H}
Boc
N
N
CF2D
CF3
220 210 200 190 180 170 160 150 140 130 120 110 100 90 80 70 60 50 40 30 20 10 0 -10 -20
f1 (ppm)

_N_-(difluoromethyl)-9-ethyl-_N_-(trifluoromethyl)-9_H_-carbazol-3-amine (13)

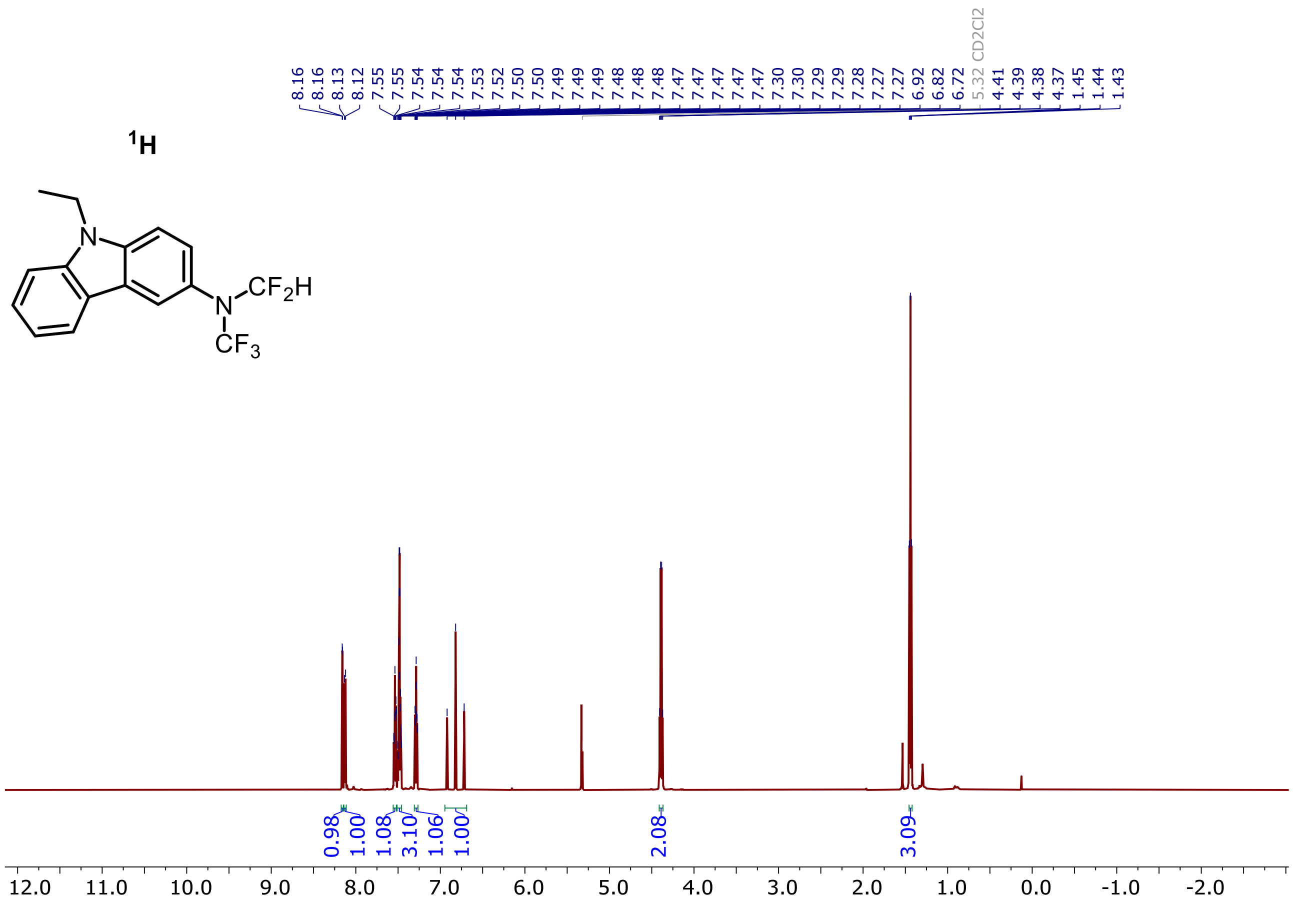

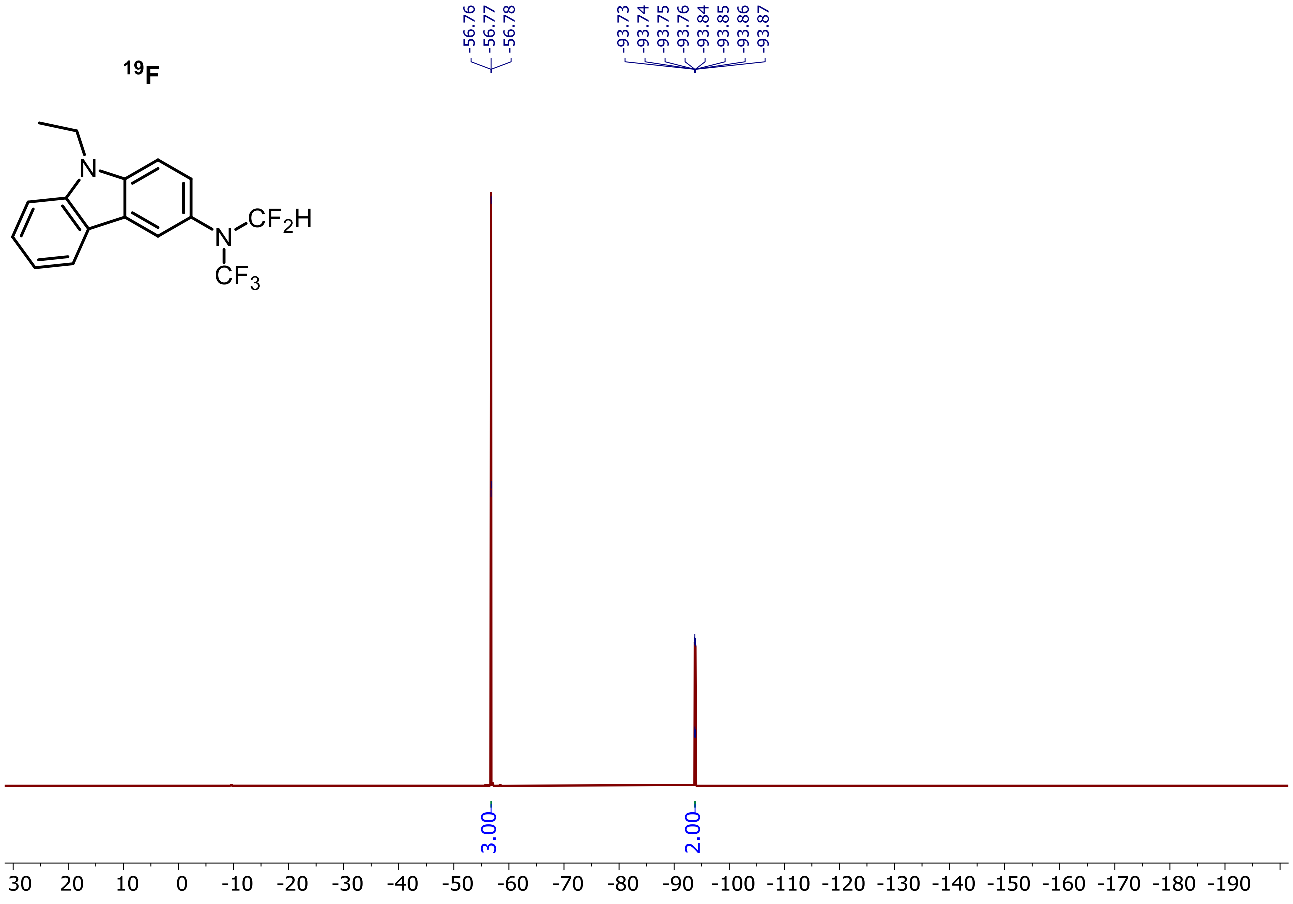

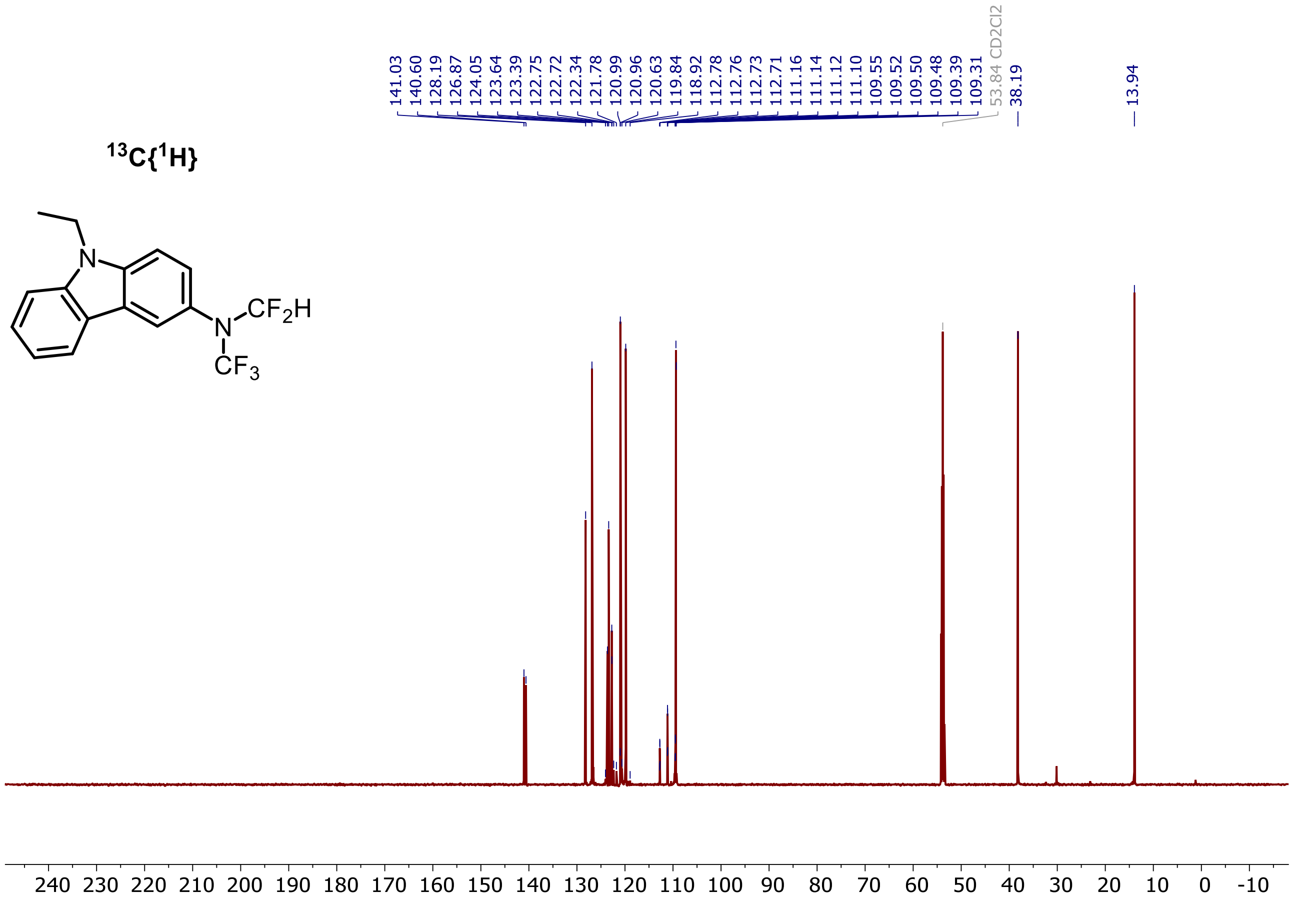

methyl 4-((difluoromethyl)(trifluoromethyl)amino)thiophene-2-carboxylate (14)

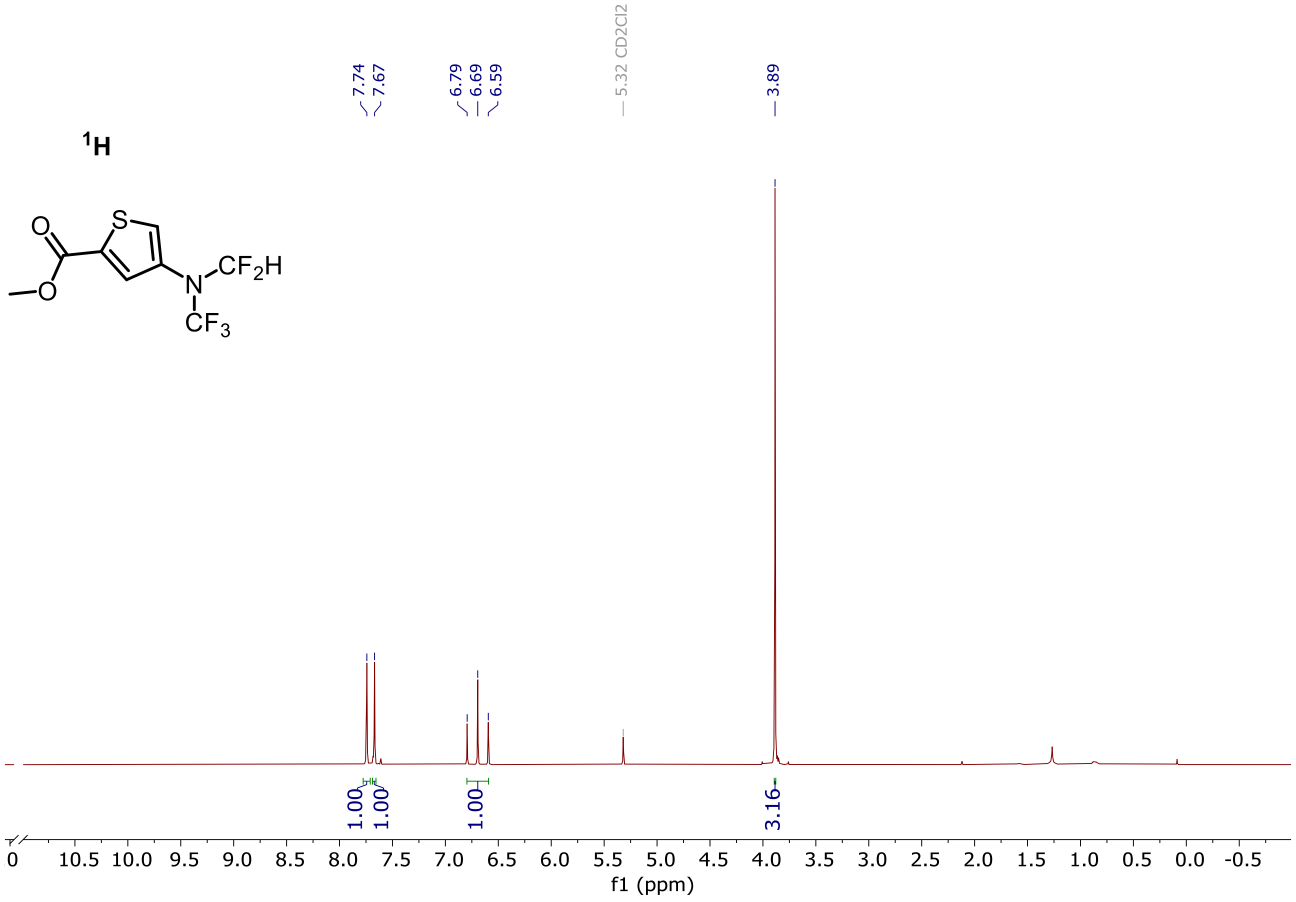

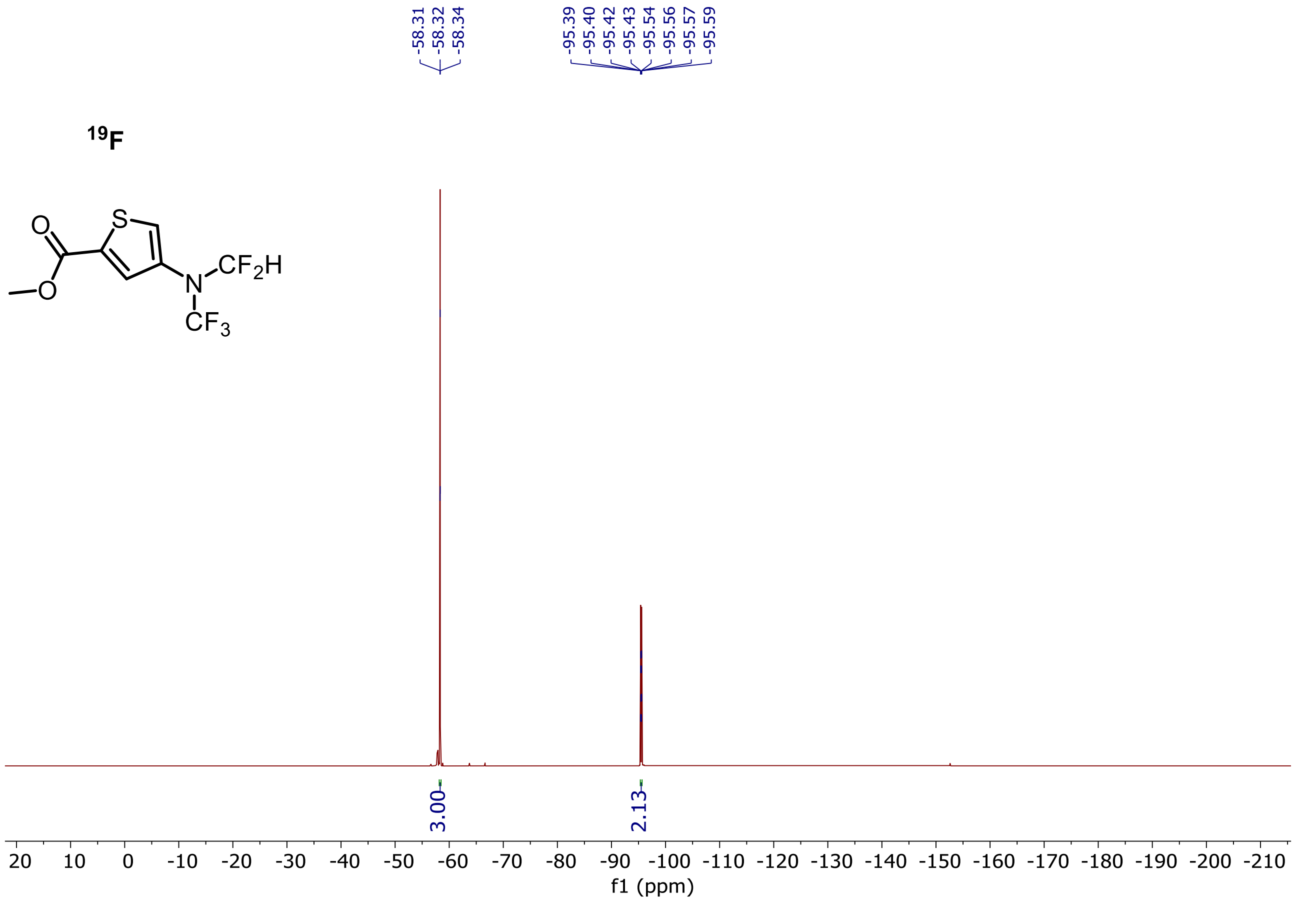
-58.31
-58.32
-58.34
-95.39
-95.40
-95.42
-95.43
-95.54
-95.56
-95.57
-95.59
19F
CF2H
CF3
3.00
2.13
f1 (ppm)

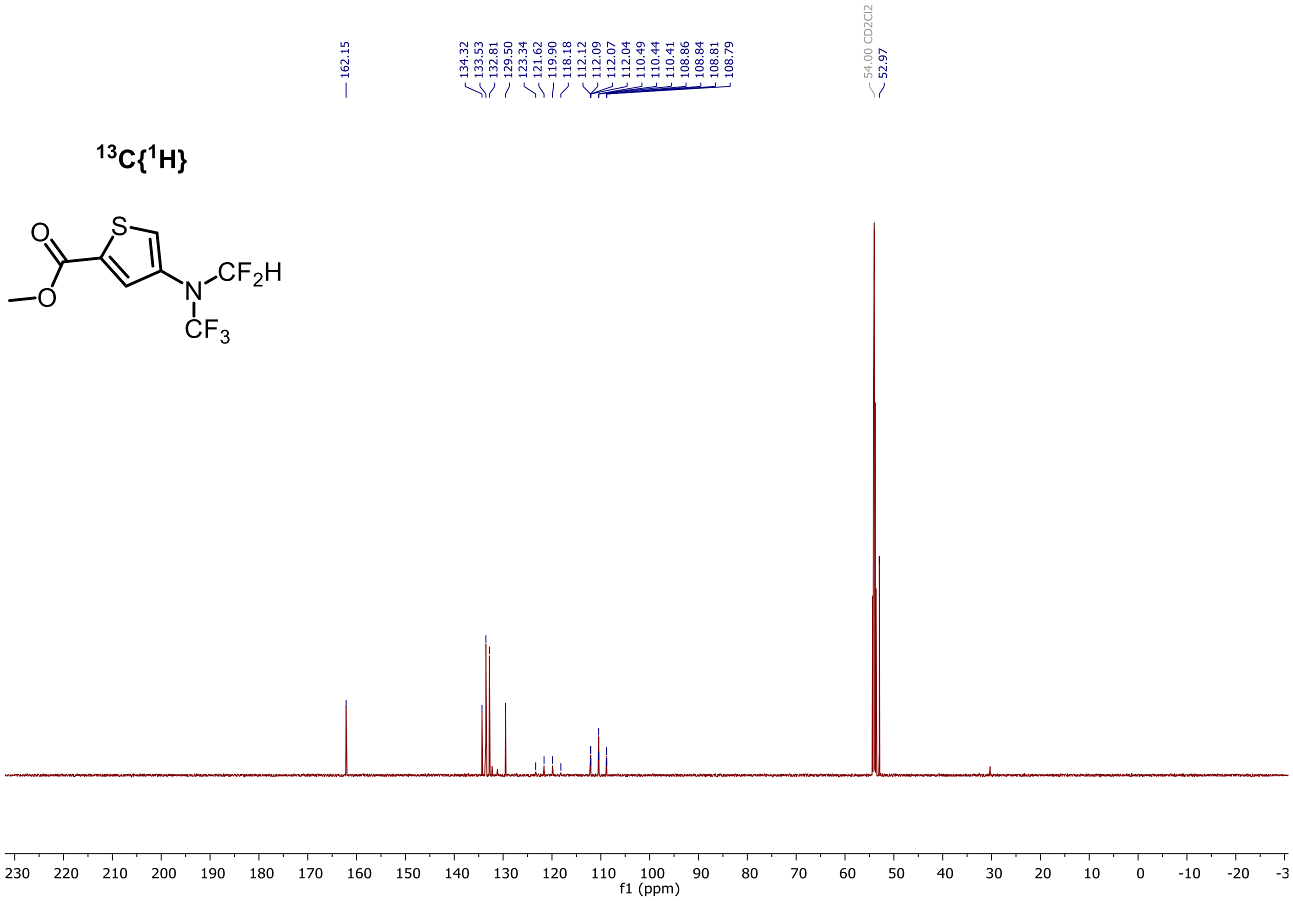
162.15
134.32
133.53
132.81
129.50
123.34
121.62
119.90
118.18
112.12
112.09
112.07
112.04
110.49
110.44
110.41
108.86
108.84
108.81
108.79
54.00 CD2Cl2
52.97
13C{1H}
CF2H
CF3
f1 (ppm)

trans-N-(difluoromethyl)-2-phenyl-_N_-(trifluoromethyl)cyclopropan-1-amine (15)

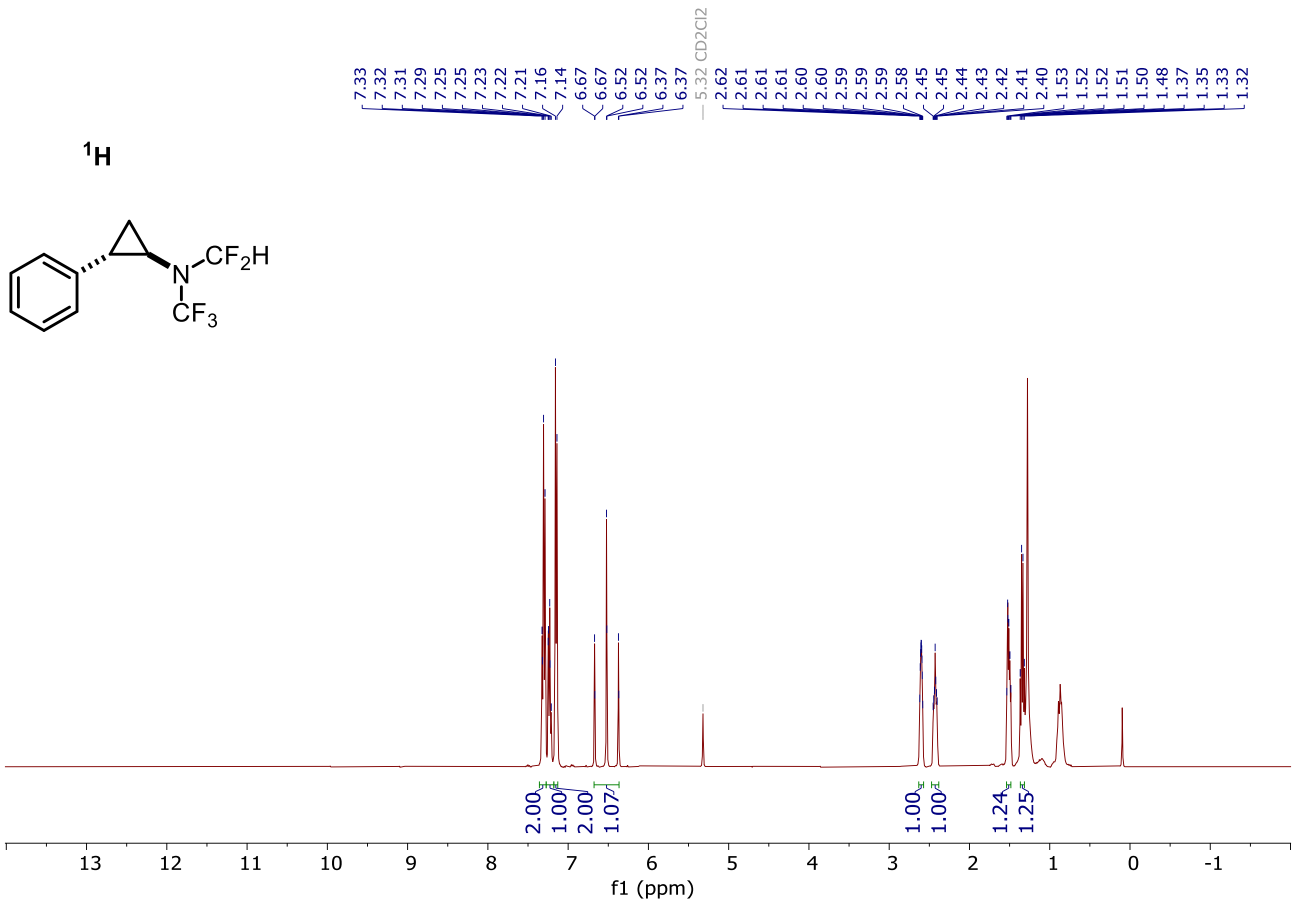

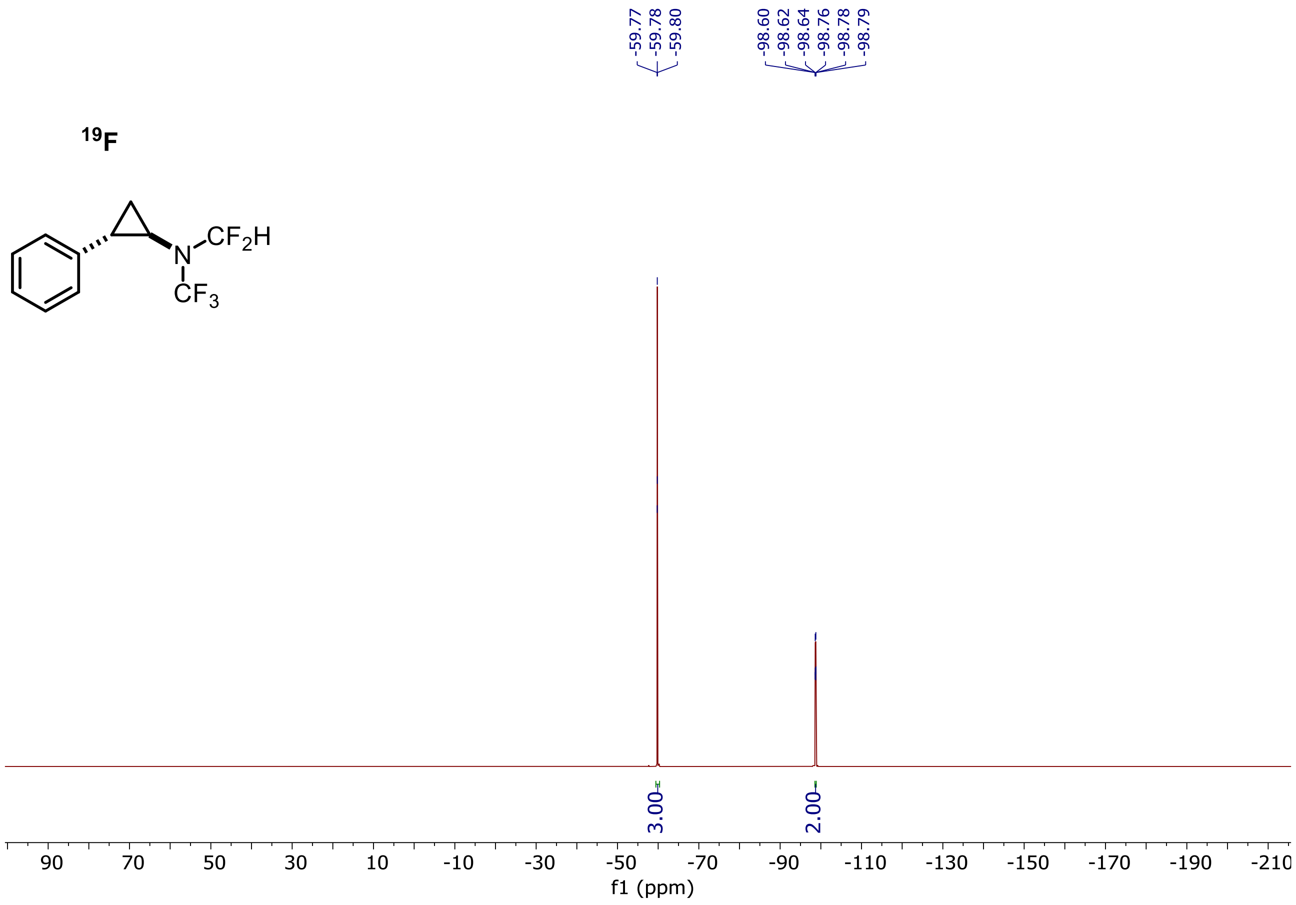

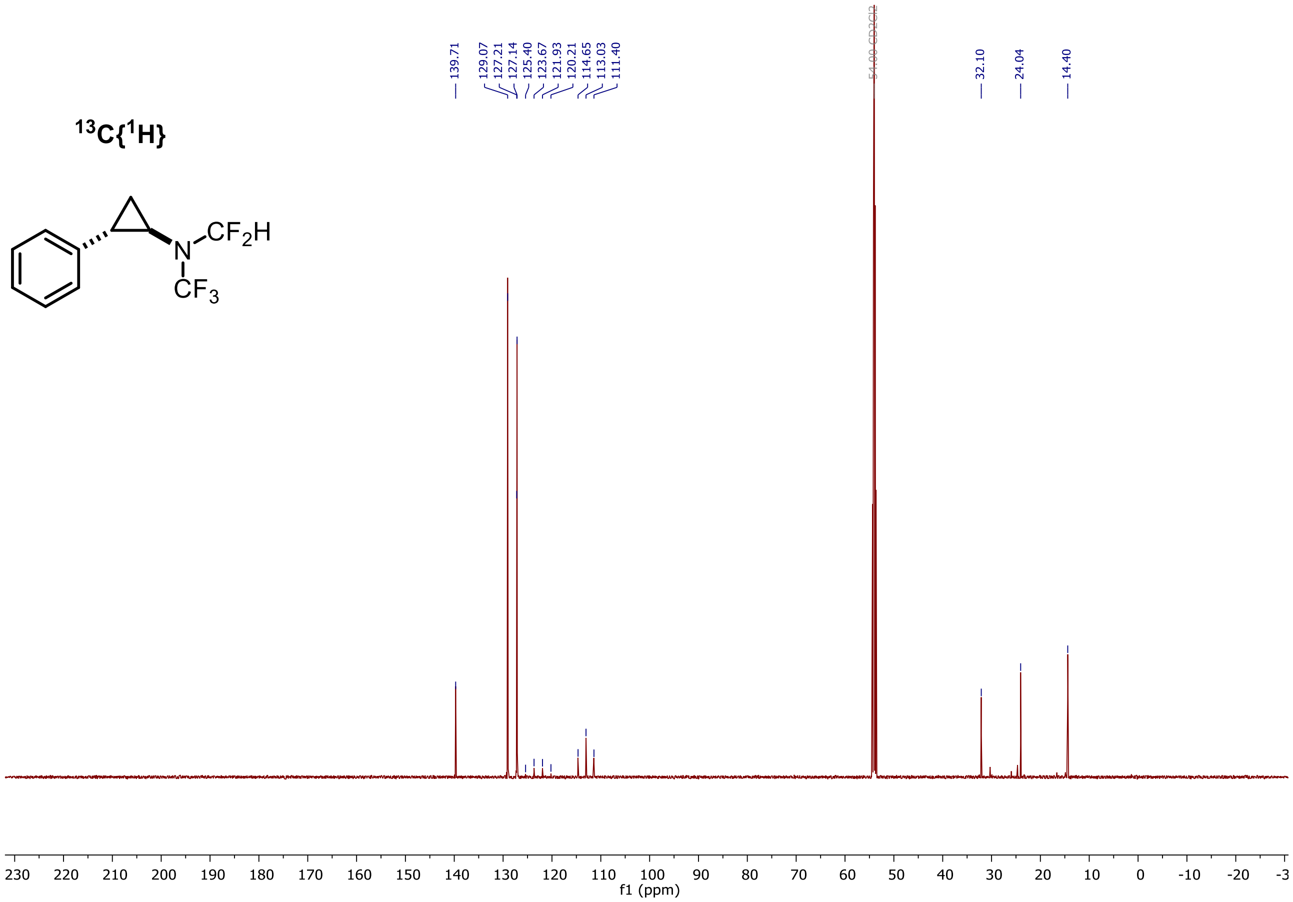

N-(difluoromethyl)-1-(2,6-dimethylphenoxy)-*N*-(trifluoromethyl)propan-2-amine (16)

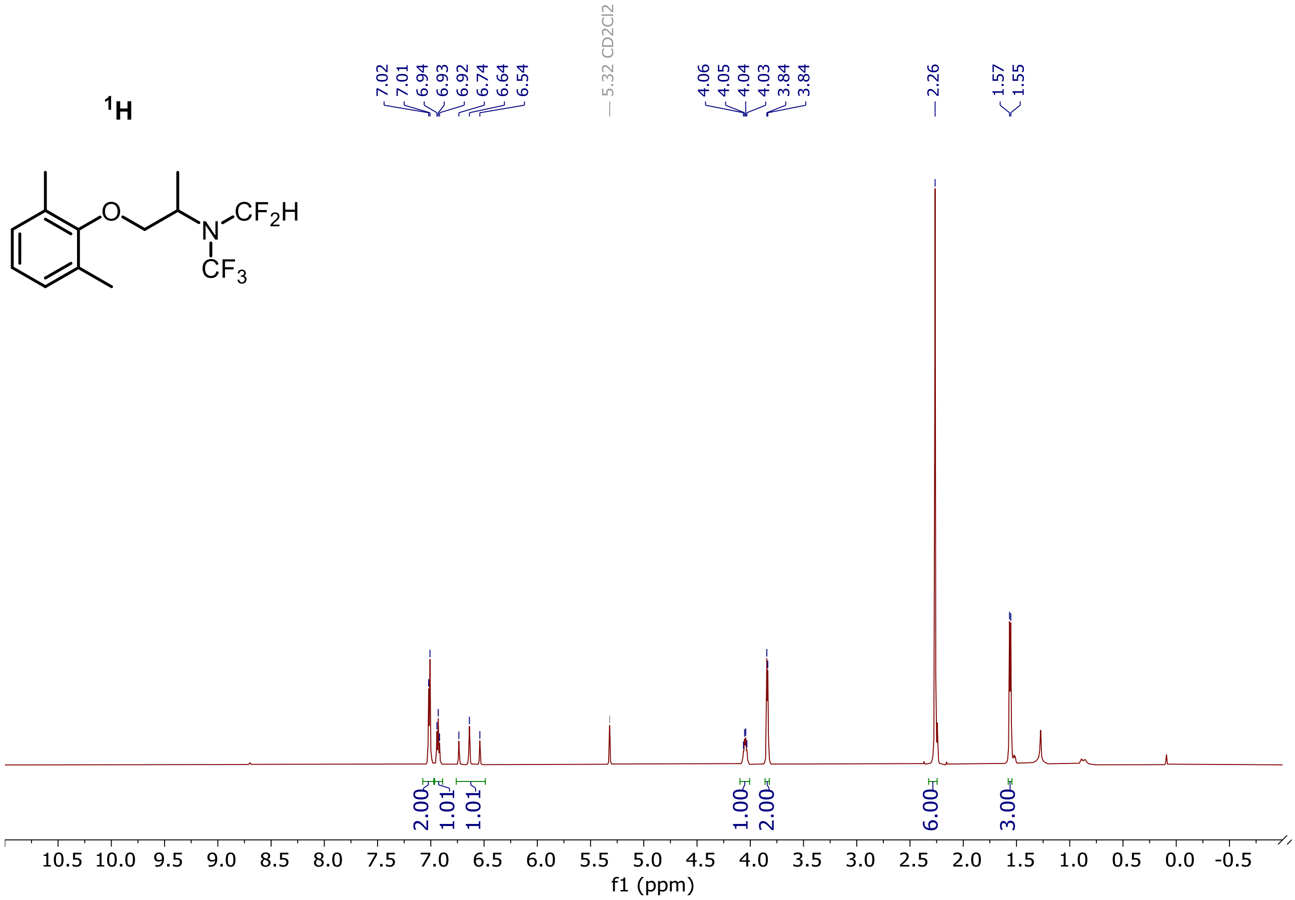

^{19}F

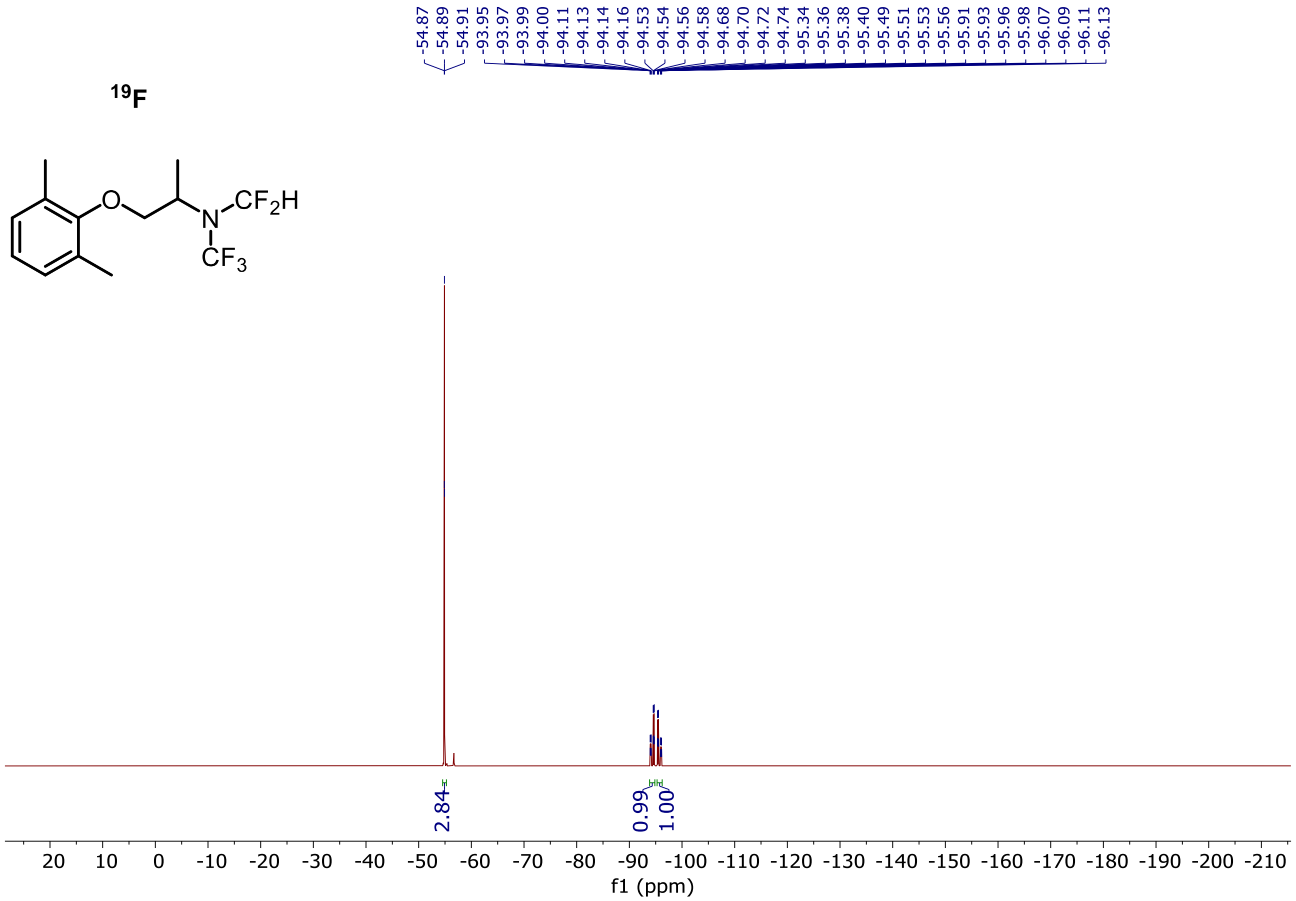

-54.87
-54.89
-54.91
-93.95
-93.97
-93.99
-94.00
-94.11
-94.13
-94.14
-94.16
-94.53
-94.54
-94.56
-94.58
-94.68
-94.70
-94.72
-94.74
-95.34
-95.36
-95.38
-95.40
-95.49
-95.51
-95.53
-95.56
-95.91
-95.93
-95.96
-95.98
-96.07
-96.09
-96.11
-96.13
O
N
CF2H
CF3
2.84
0.99
1.00
20 10 0 -10 -20 -30 -40 -50 -60 -70 -80 -90 -100 -110 -120 -130 -140 -150 -160 -170 -180 -190 -200 -210
f1 (ppm)

$^{13}C\{^{1}H\}$

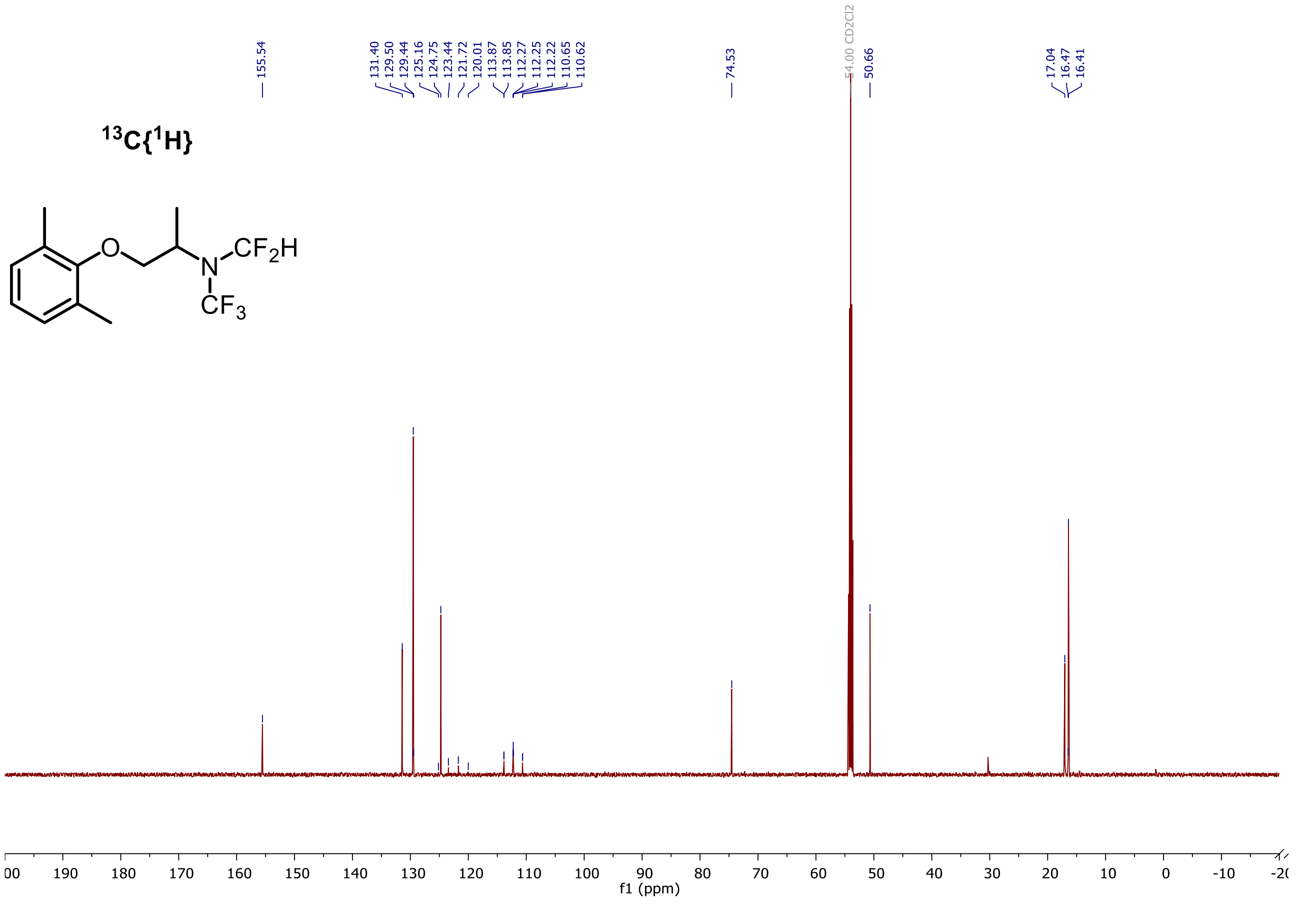

155.54
131.40
129.50
129.44
125.16
124.75
123.44
121.72
120.01
113.87
113.85
112.27
112.25
112.22
110.65
110.62
74.53
54.00 CD2Cl2
50.66
17.04
16.47
16.41
O
N
CF2H
CF3
00 190 180 170 160 150 140 130 120 110 100 90 80 70 60 50 40 30 20 10 0 -10 -2(
f1 (ppm)

(*R*)-2-((6-(3-((difluoromethyl)(trifluoromethyl)amino)piperidin-1-yl)-3-methyl-2,4-dioxo-3,4-dihydropyrimidin-1(2H)-yl)methyl)benzonitrile (17)

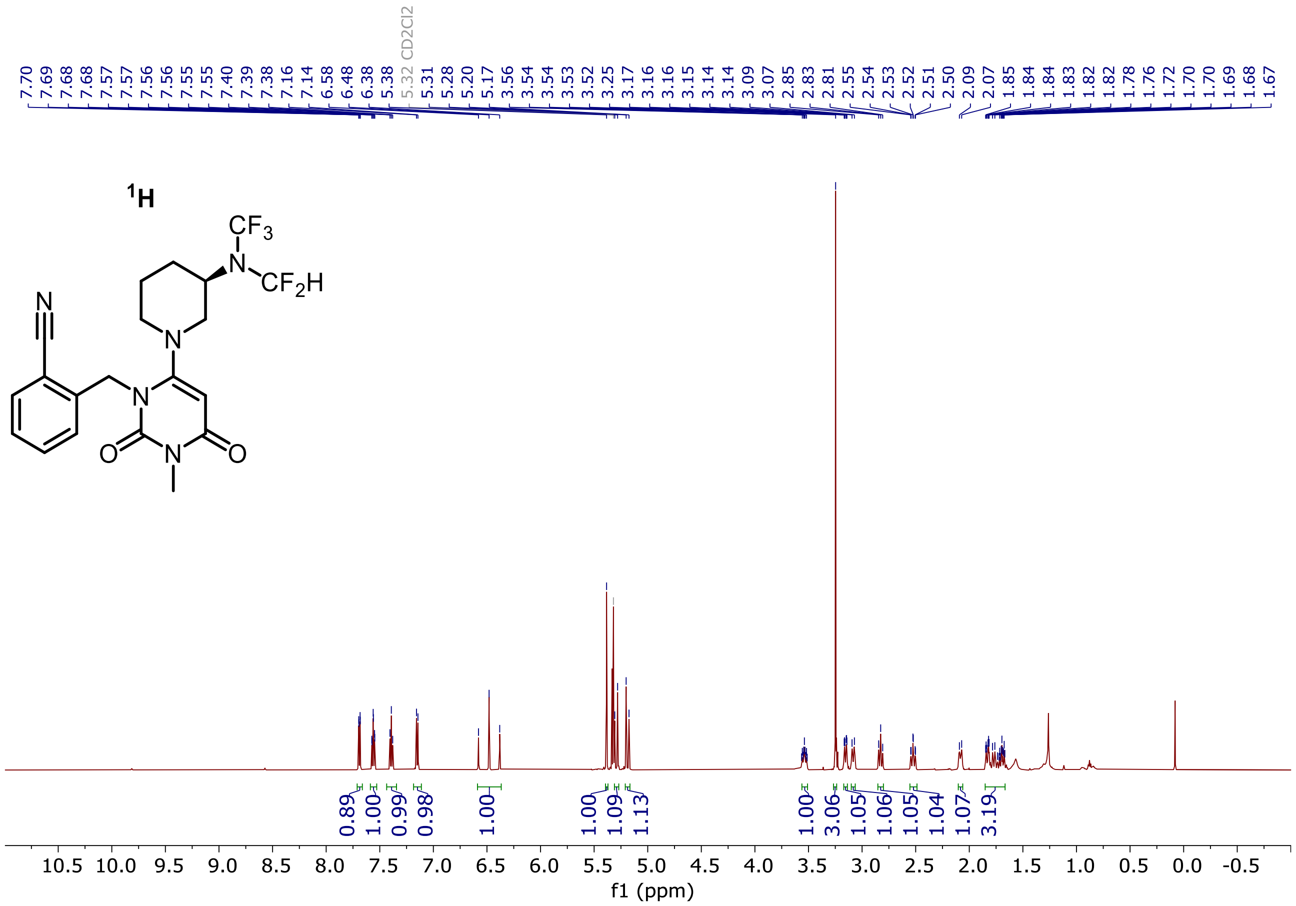

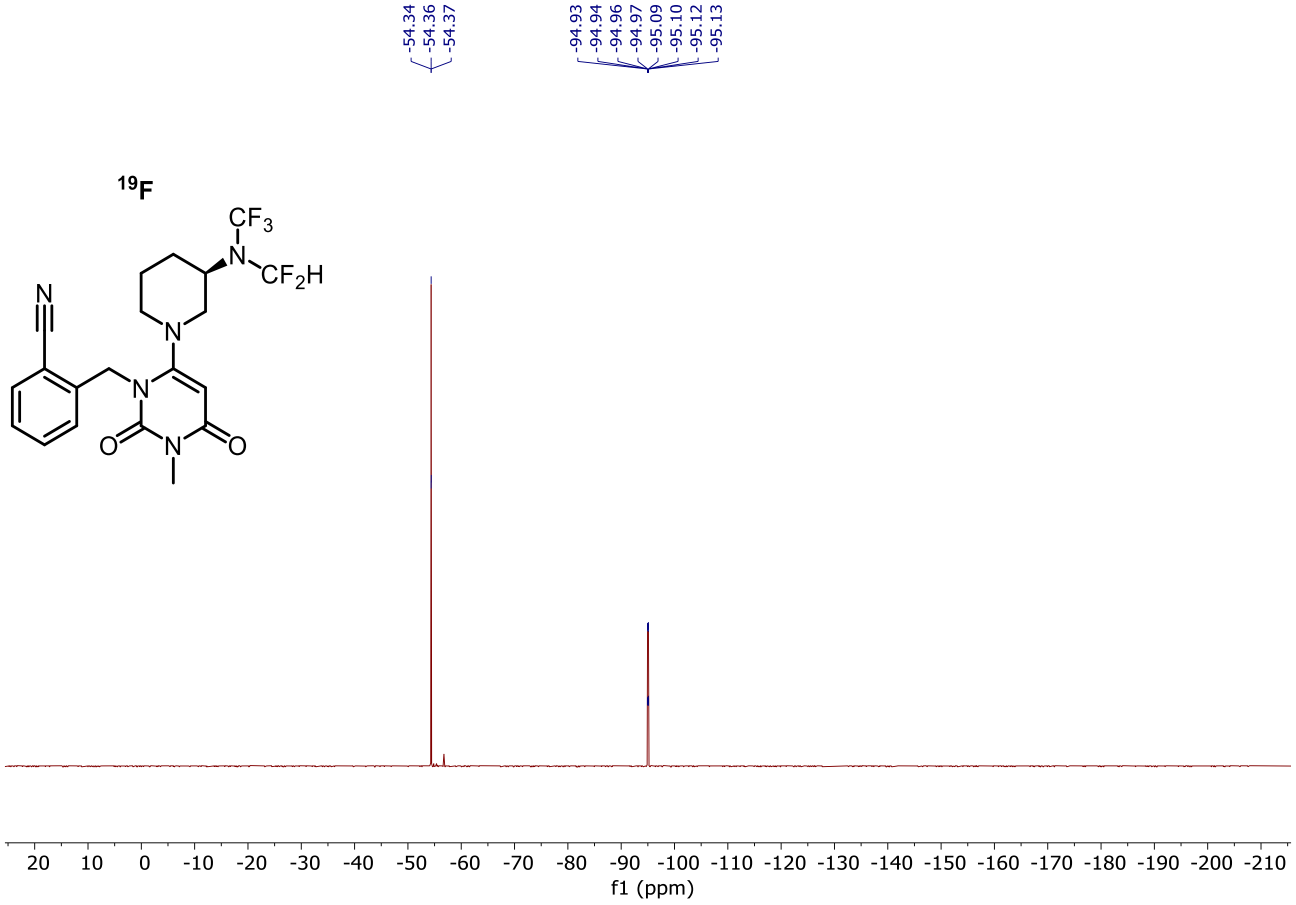

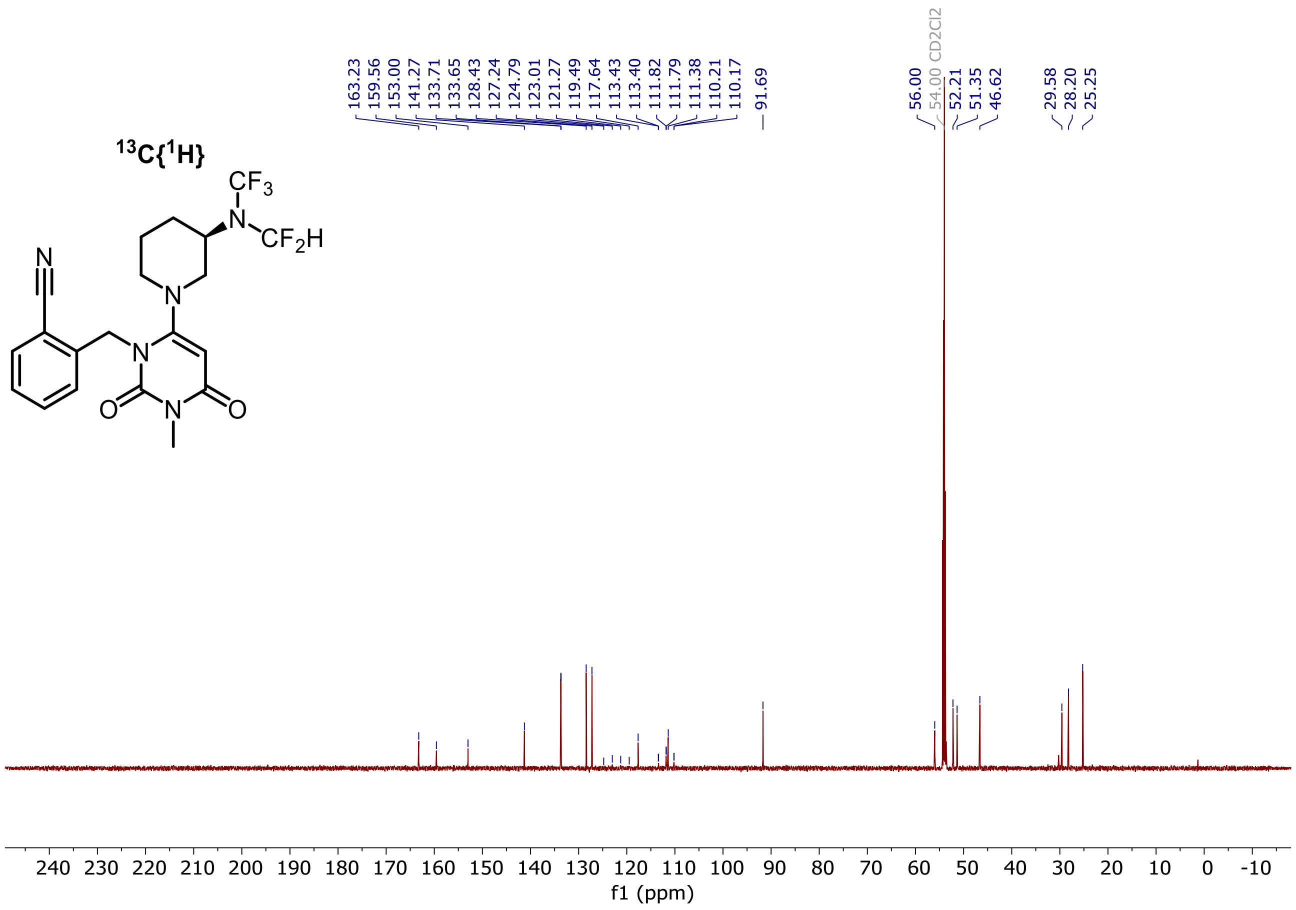

4-butyl-2-chloro-*N*-(difluoromethyl)-*N*-(trifluoromethyl)aniline (18)

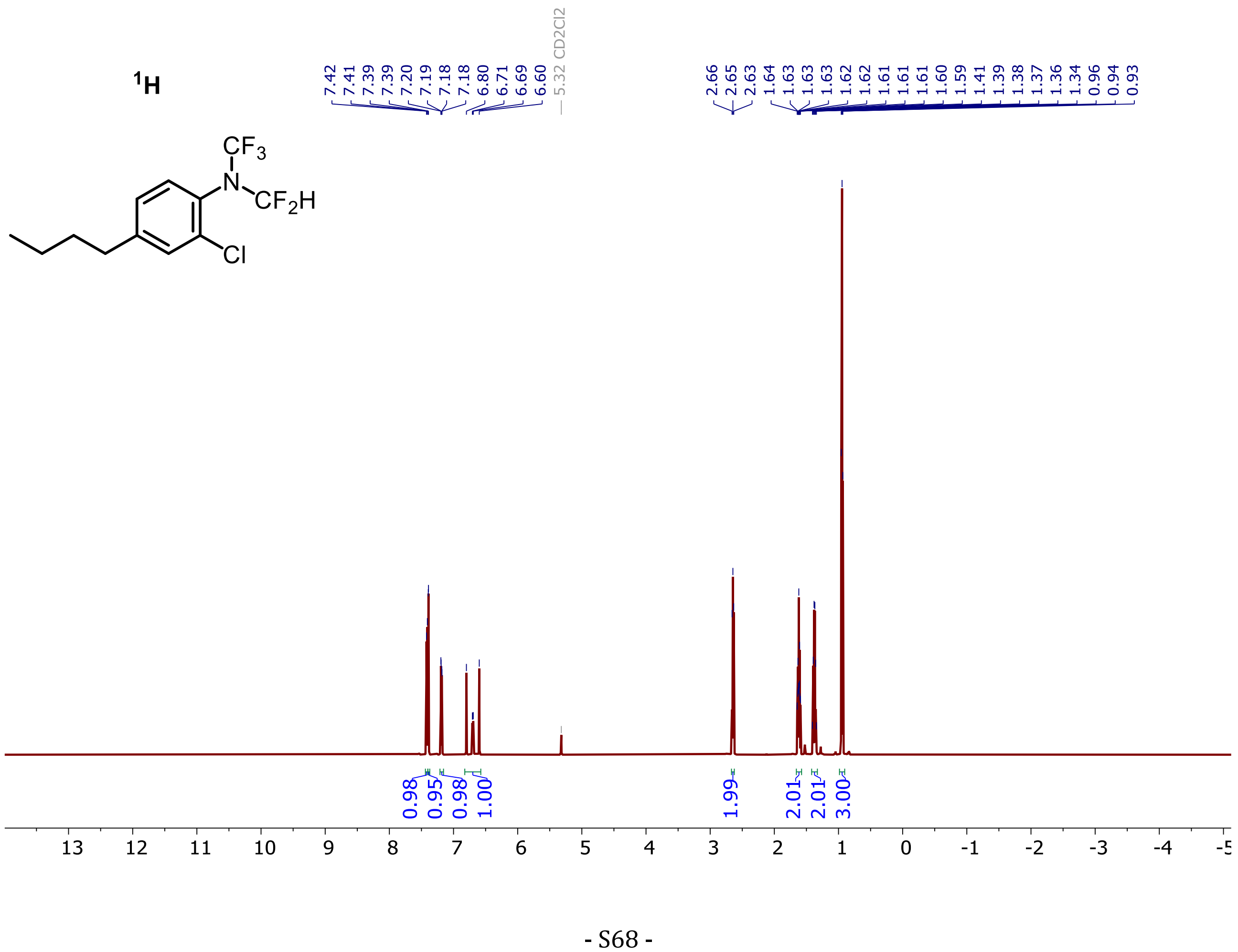

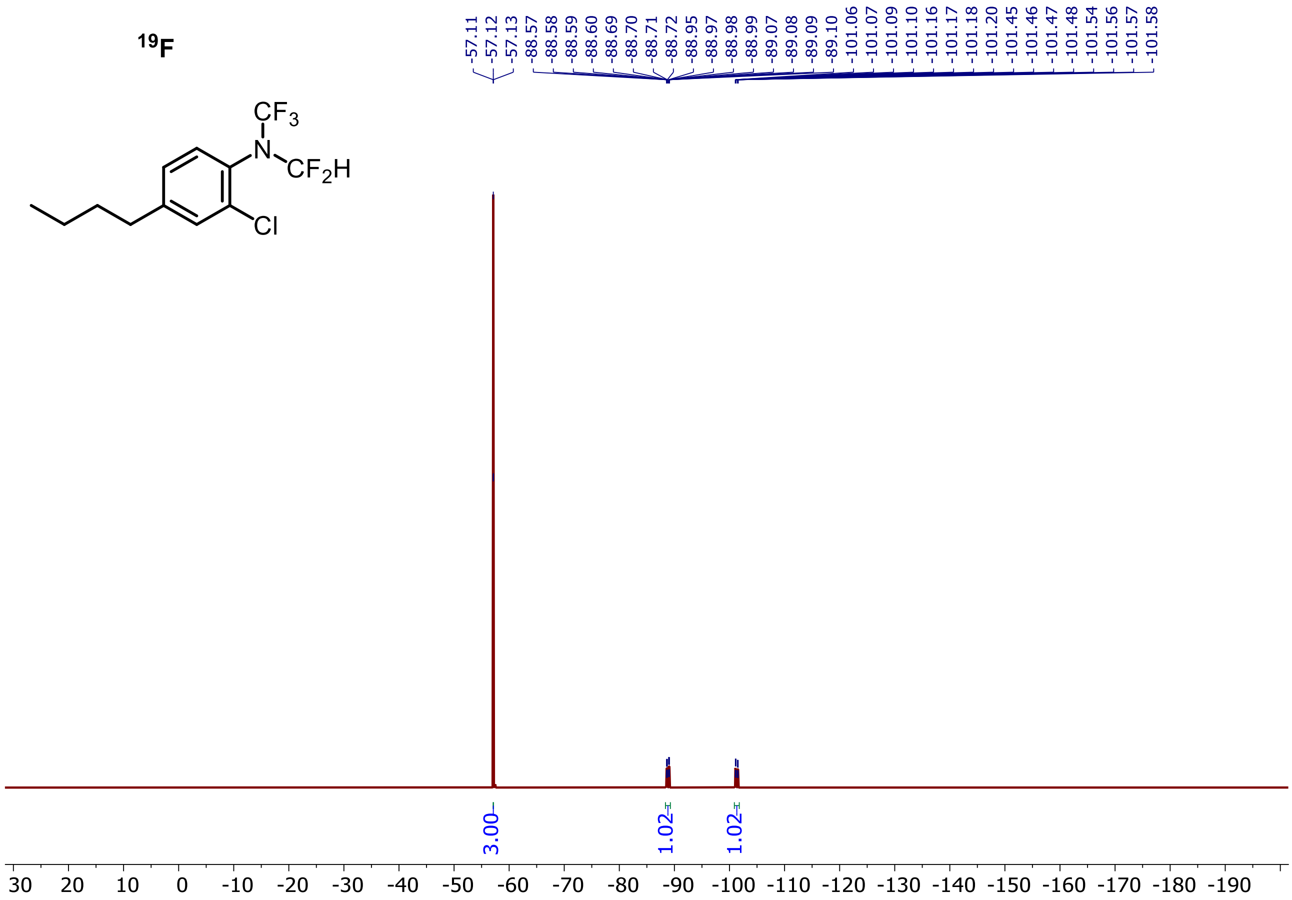

19F
CF3
N
CF2H
Cl
-57.11
-57.12
-57.13
-88.57
-88.58
-88.59
-88.60
-88.69
-88.70
-88.71
-88.72
-88.95
-88.97
-88.98
-88.99
-89.07
-89.08
-89.09
-89.10
-101.06
-101.07
-101.09
-101.10
-101.16
-101.17
-101.18
-101.20
-101.45
-101.46
-101.47
-101.48
-101.54
-101.56
-101.57
-101.58
3.00
1.02
1.02
30 20 10 0 -10 -20 -30 -40 -50 -60 -70 -80 -90 -100 -110 -120 -130 -140 -150 -160 -170 -180 -190

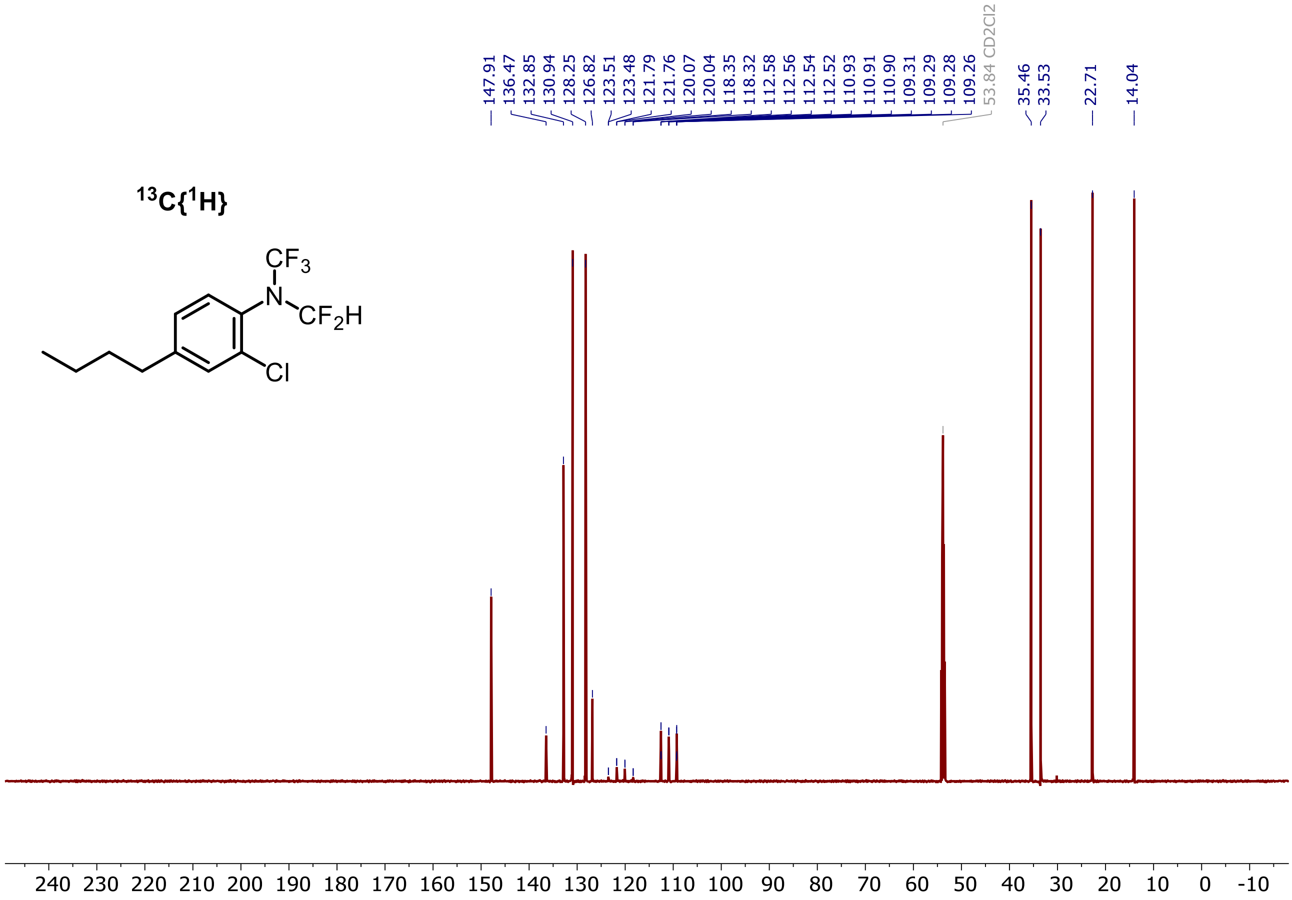

13C{1H}
CF3
N
CF2H
Cl
147.91
136.47
132.85
130.94
128.25
126.82
123.51
123.48
121.79
121.76
120.07
120.04
118.35
118.32
112.58
112.56
112.54
112.52
110.93
110.91
110.90
109.31
109.29
109.28
109.26
53.84 CD2Cl2
35.46
33.53
22.71
14.04
240 230 220 210 200 190 180 170 160 150 140 130 120 110 100 90 80 70 60 50 40 30 20 10 0 -10

2-chloro-*N*-(difluoromethyl)-4-((4-methoxyphenyl)ethynyl)-*N*-(trifluoromethyl)aniline (19)

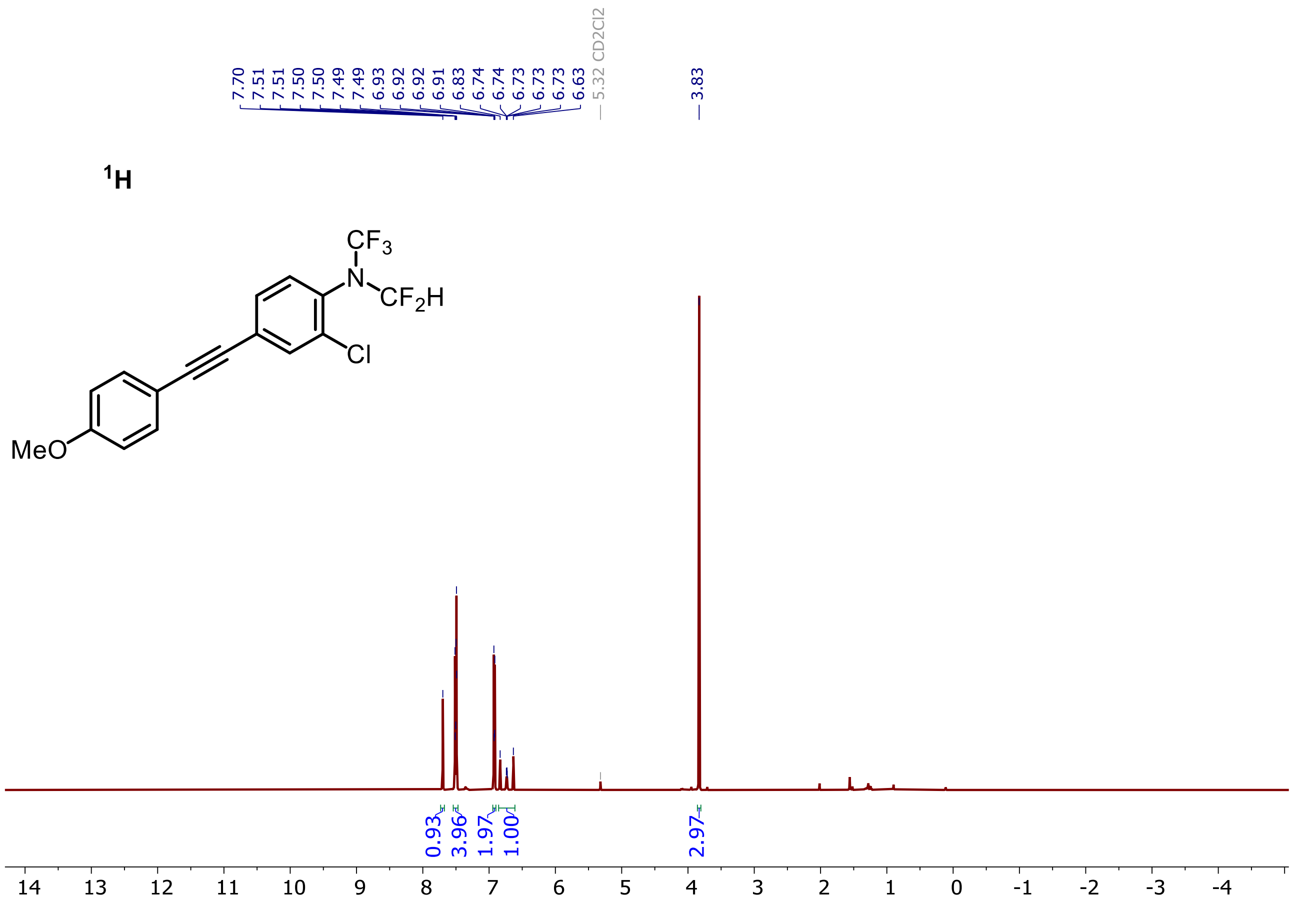

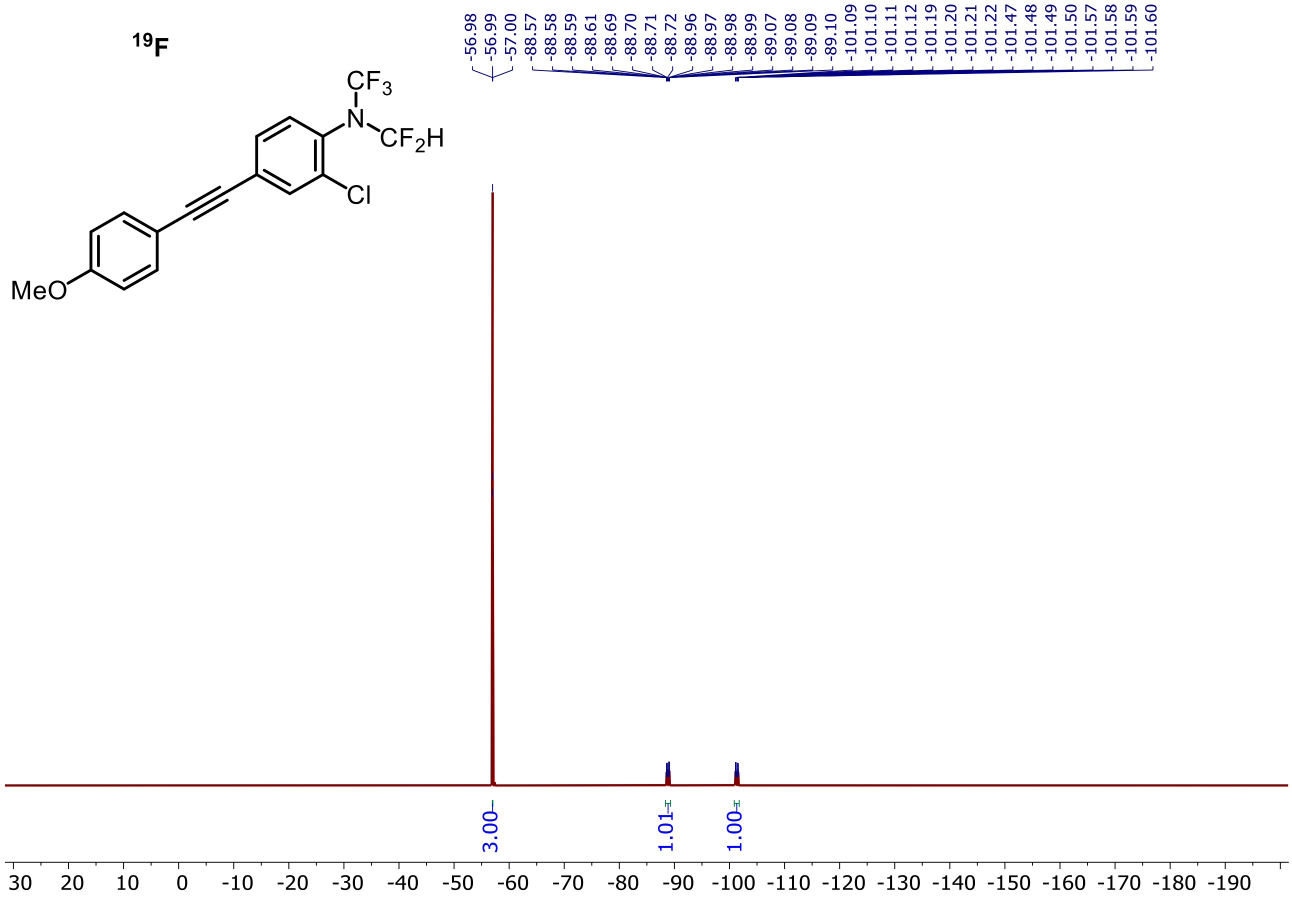

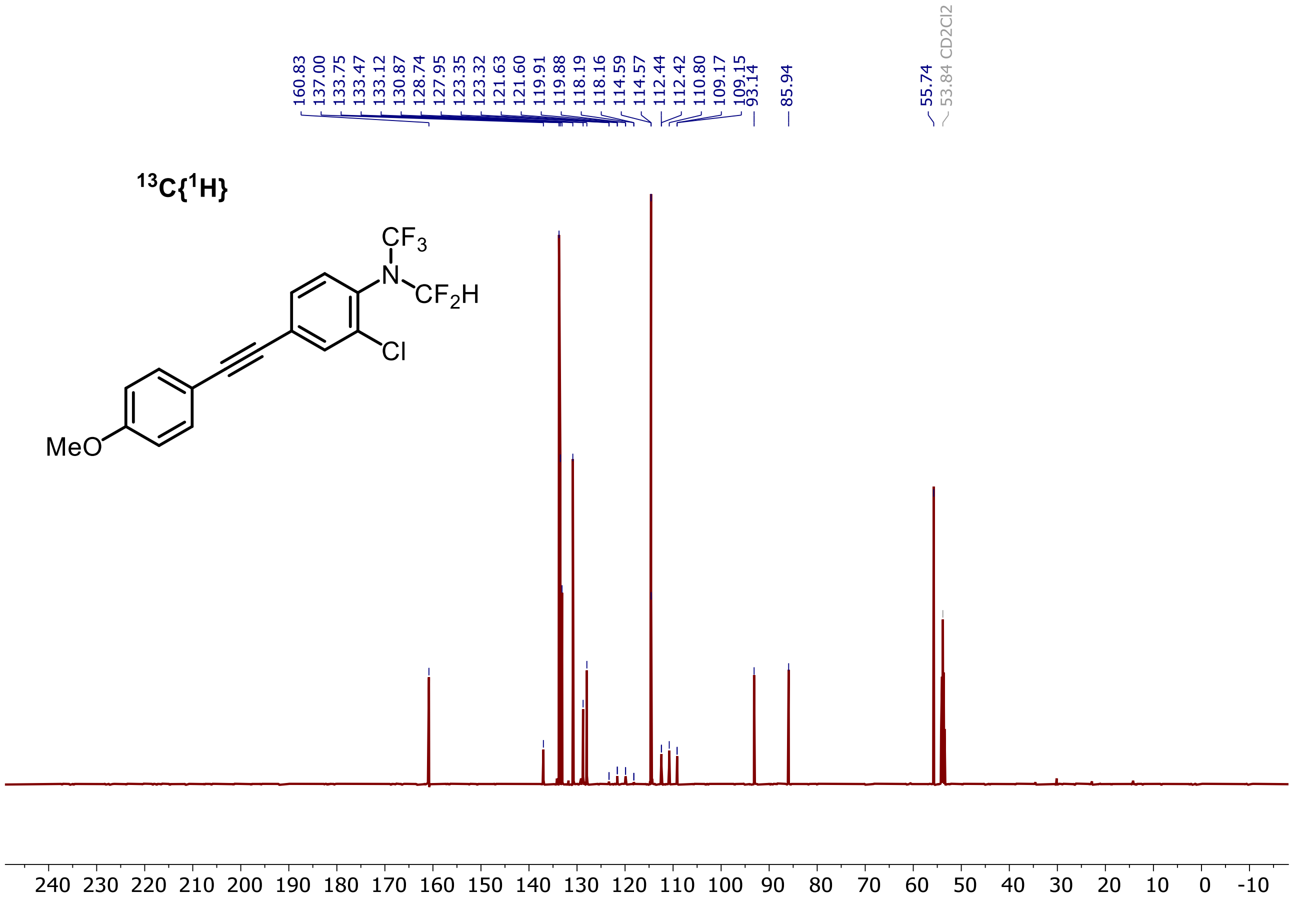

_N_-(3-chloro-4-((difluoromethyl)(trifluoromethyl)amino)phenyl)methanesulfonamide (20)

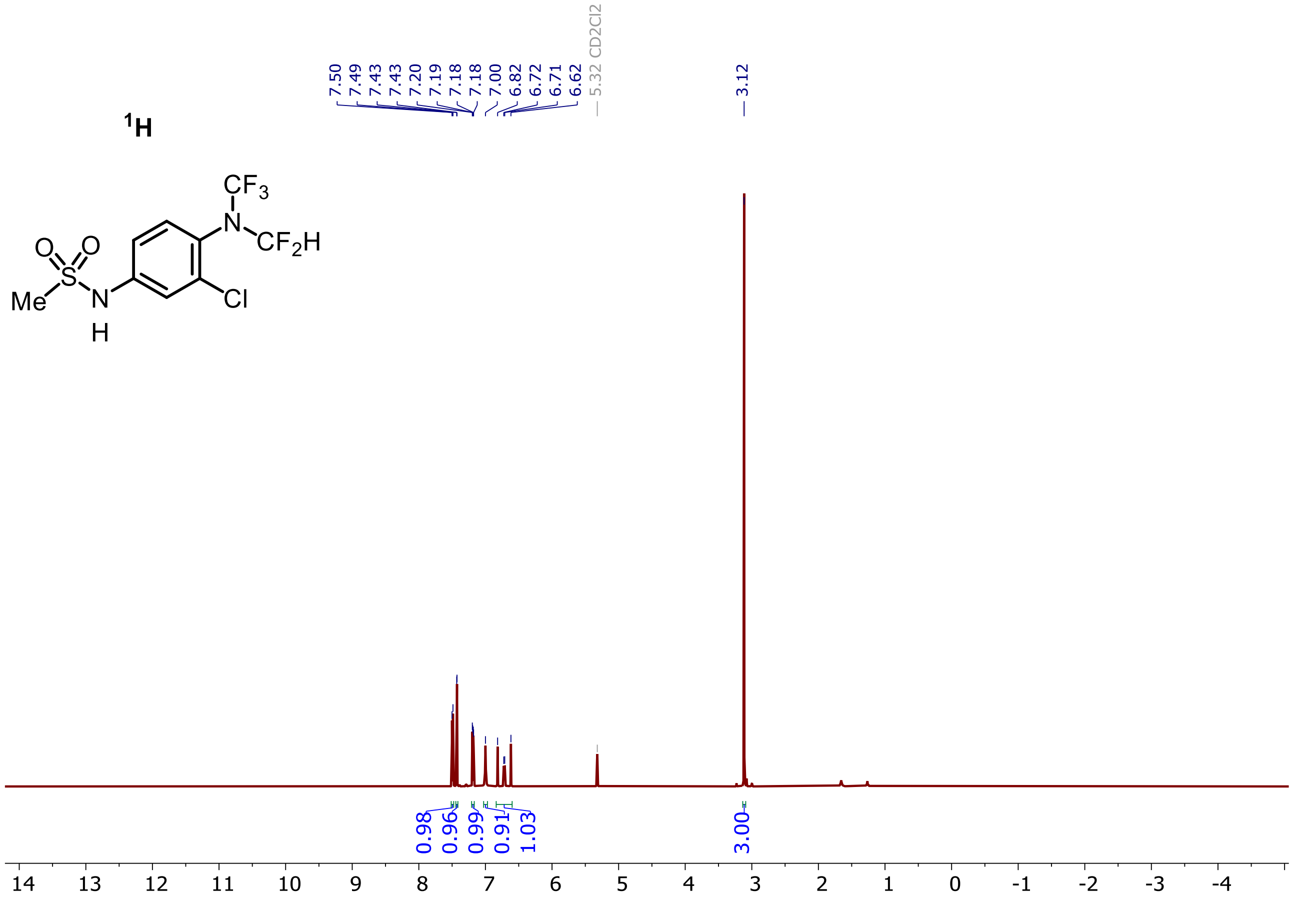

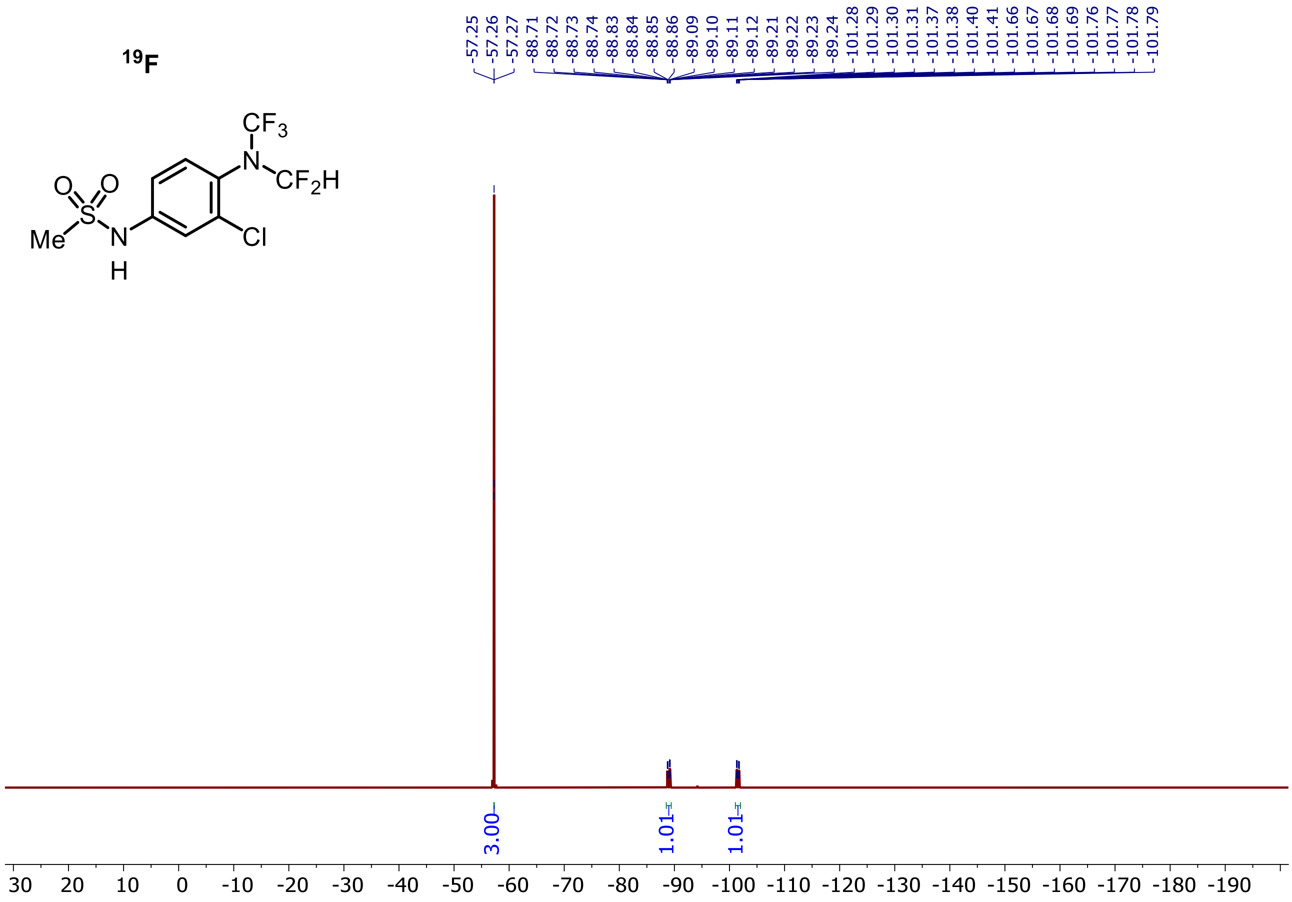
19F
-57.25
-57.26
-57.27
-88.71
-88.72
-88.73
-88.74
-88.83
-88.84
-88.85
-88.86
-89.09
-89.10
-89.11
-89.12
-89.21
-89.22
-89.23
-89.24
-101.28
-101.29
-101.30
-101.31
-101.37
-101.38
-101.40
-101.41
-101.66
-101.67
-101.68
-101.69
-101.76
-101.77
-101.78
-101.79
3.00
1.01
1.01

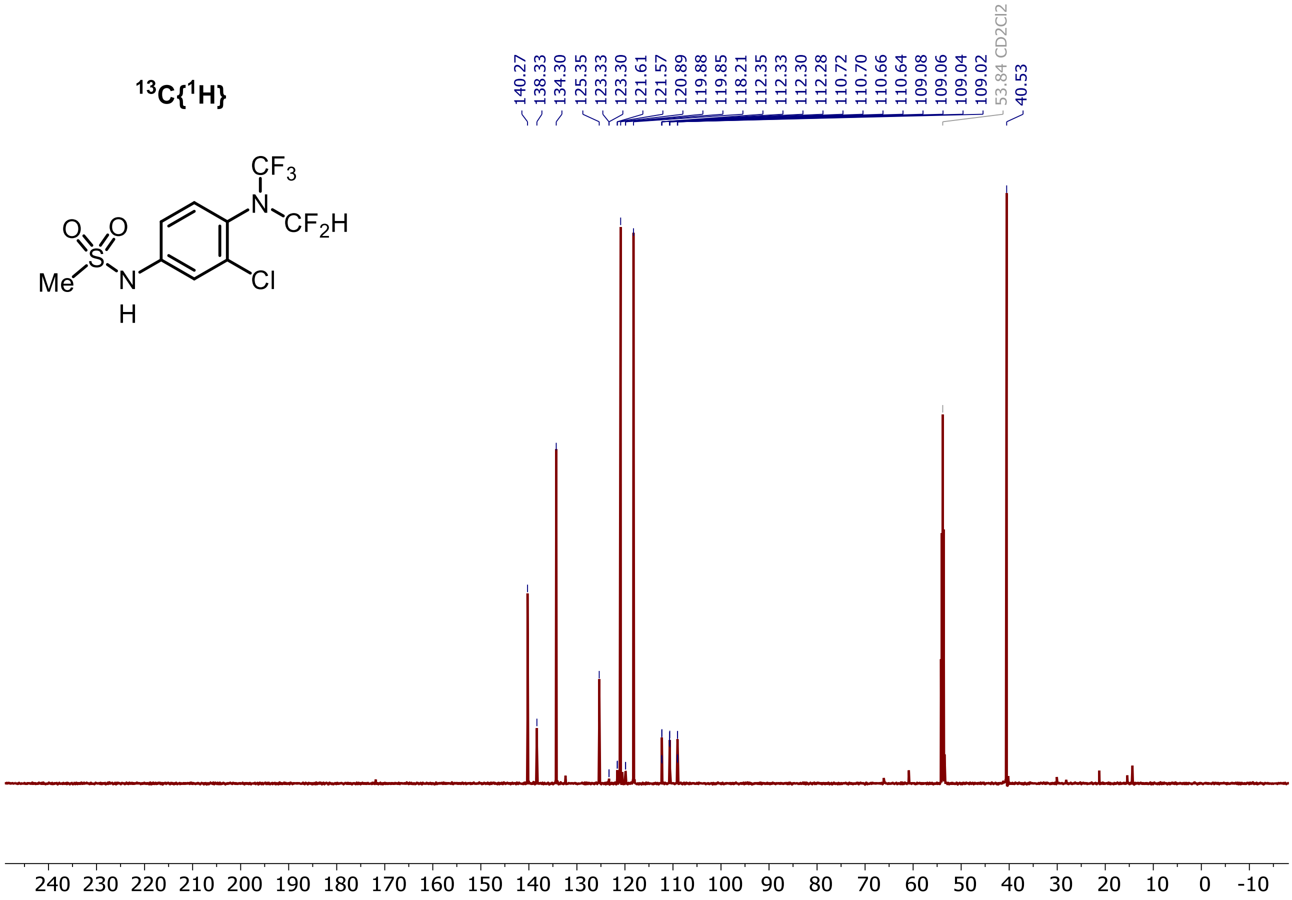
13C{1H}
140.27
138.33
134.30
125.35
123.33
123.30
121.61
121.57
120.89
119.88
119.85
118.21
112.35
112.33
112.30
112.28
110.72
110.70
110.66
110.64
109.08
109.06
109.04
109.02
53.84 CD2Cl2
40.53

_N_-(4-(2-oxa-6-azaspiro[3.3]heptan-6-yl)benzyl)-_N_-(difluoromethyl)-1,1,1-trifluoromethanamine (21)

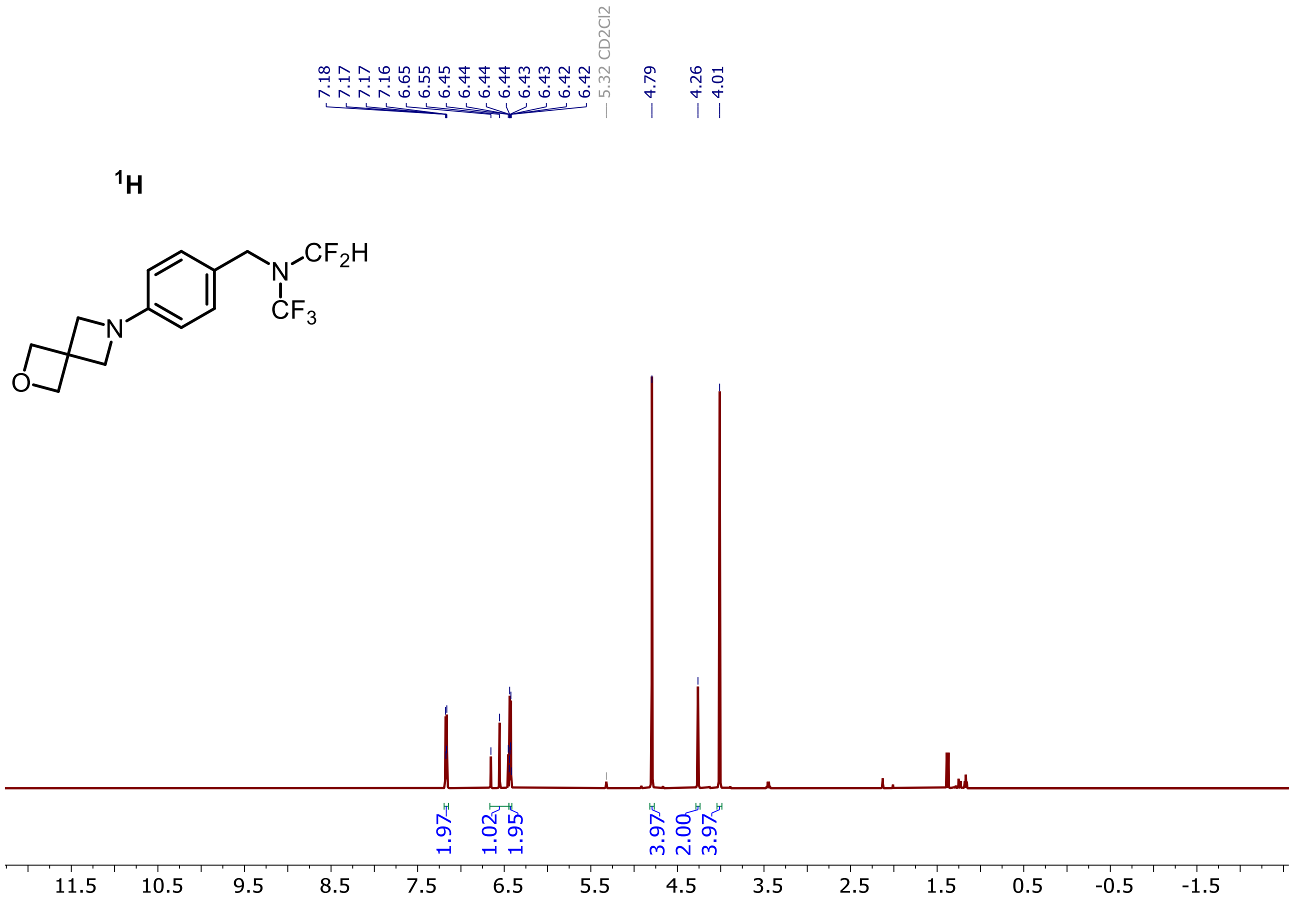

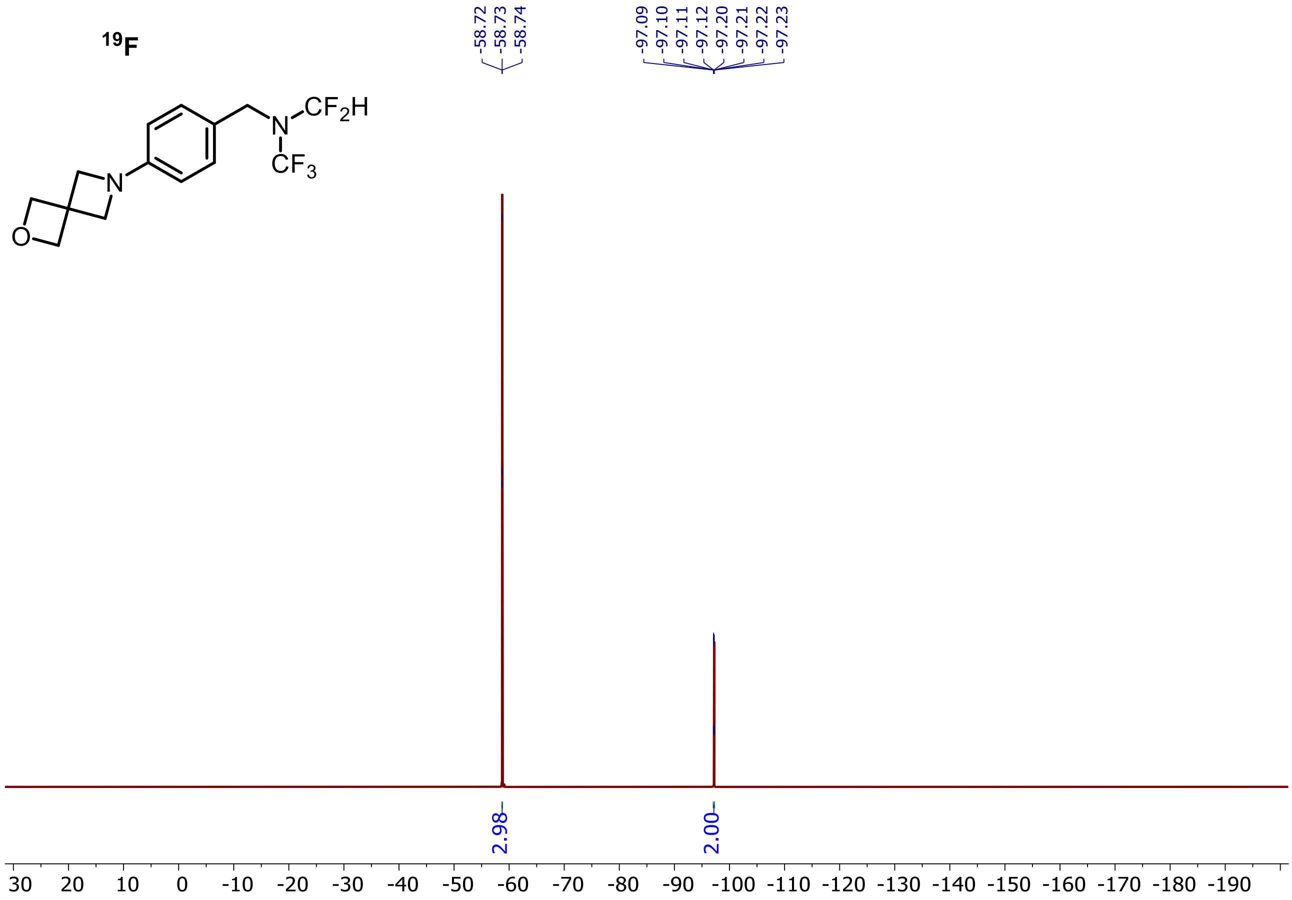

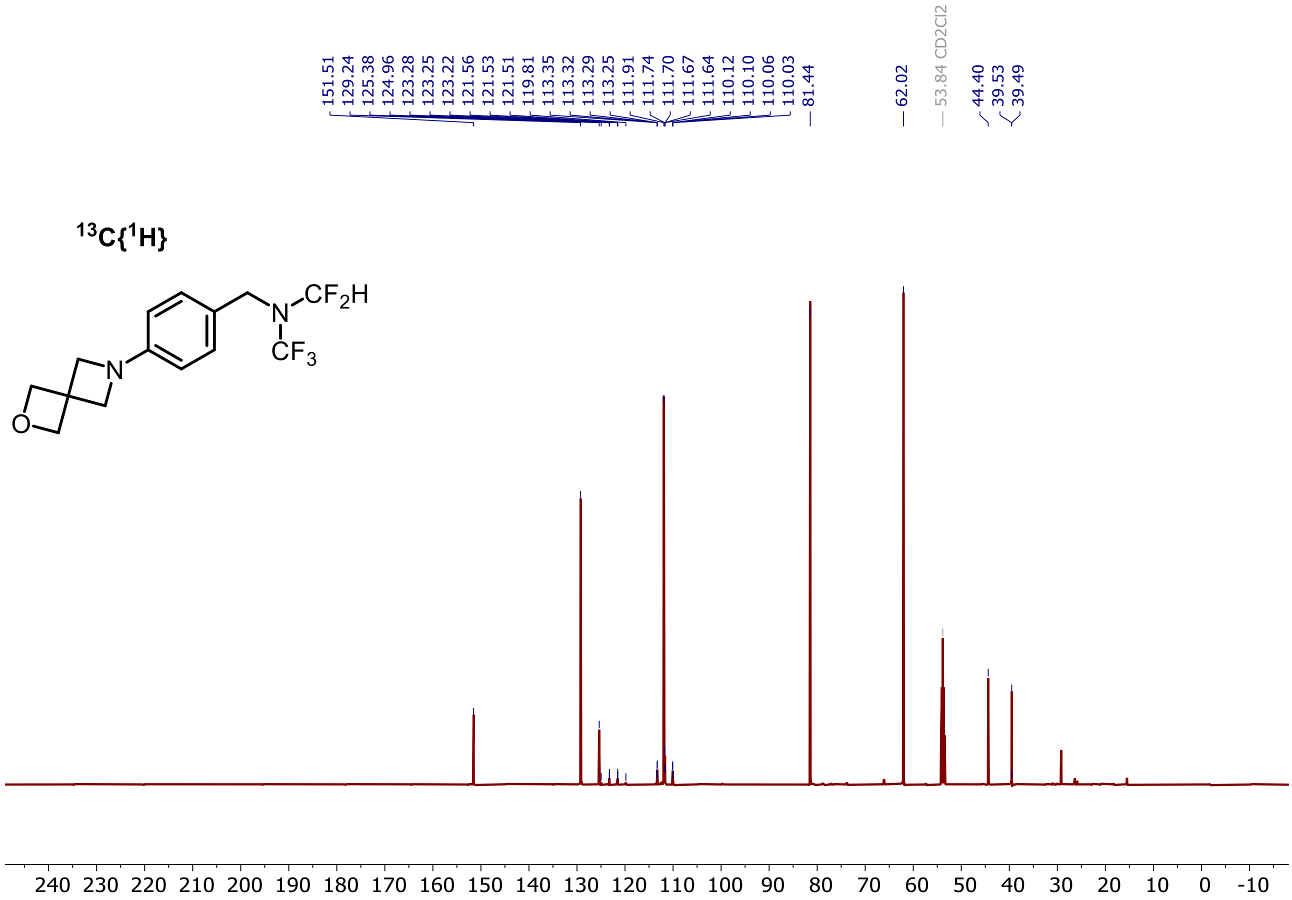

2-(diethylamino)ethyl 4-((difluoromethyl)(trifluoromethyl)amino)benzoate (22)

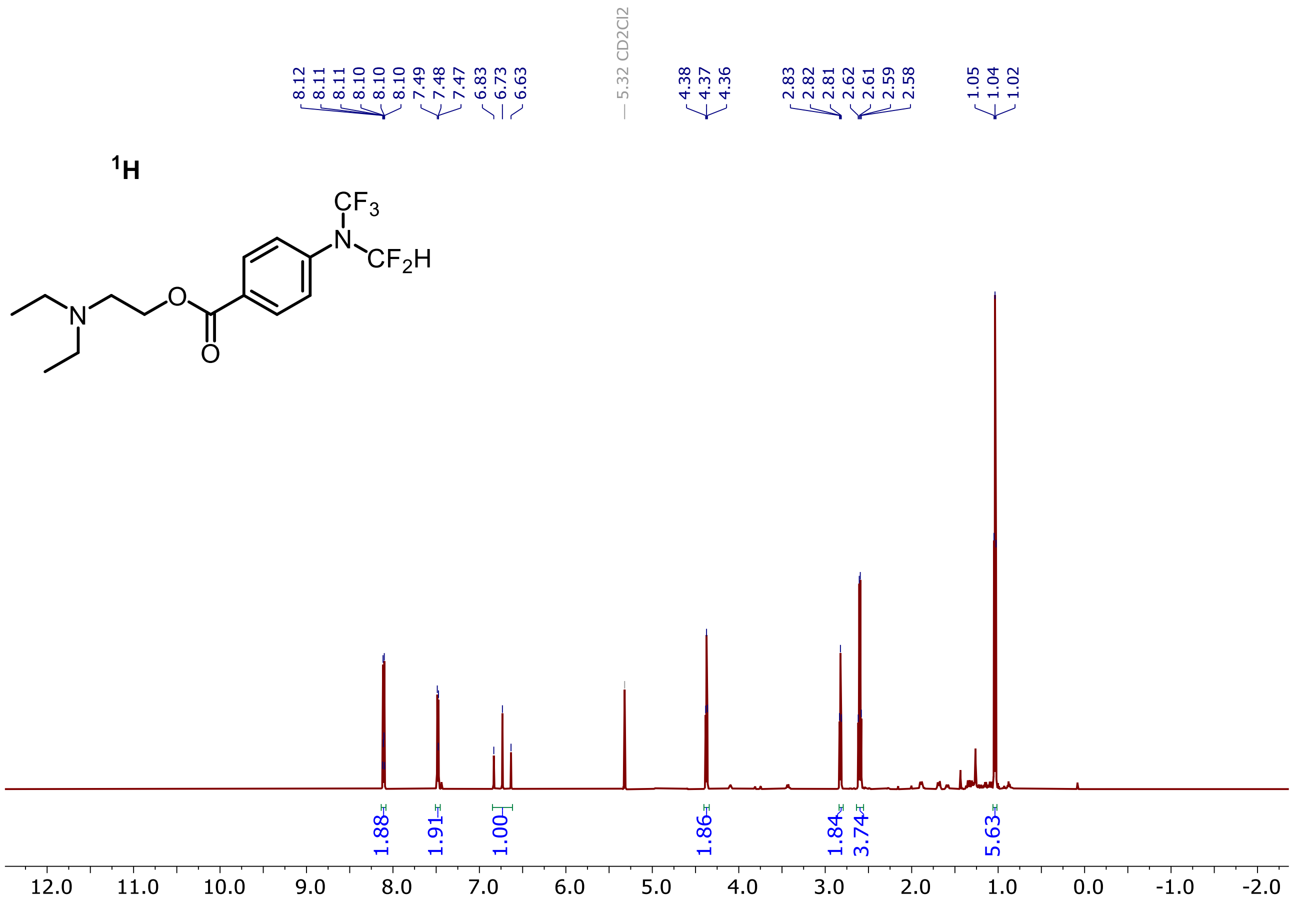

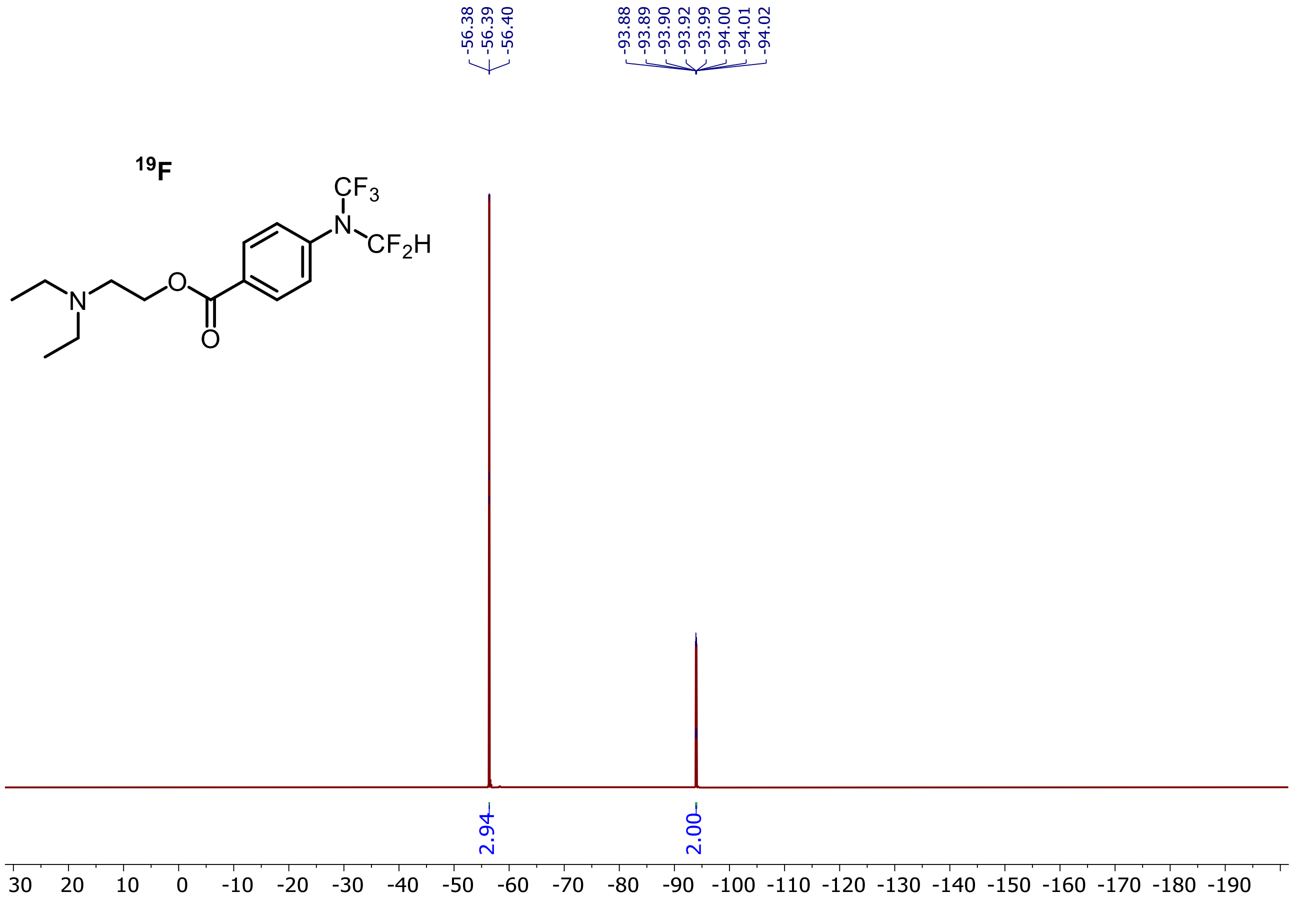
-56.38
-56.39
-56.40
-93.88
-93.89
-93.90
-93.92
-93.99
-94.00
-94.01
-94.02
19F
CF3
N
CF2H
N
O
O
2.94
2.00
30 20 10 0 -10 -20 -30 -40 -50 -60 -70 -80 -90 -100 -110 -120 -130 -140 -150 -160 -170 -180 -190

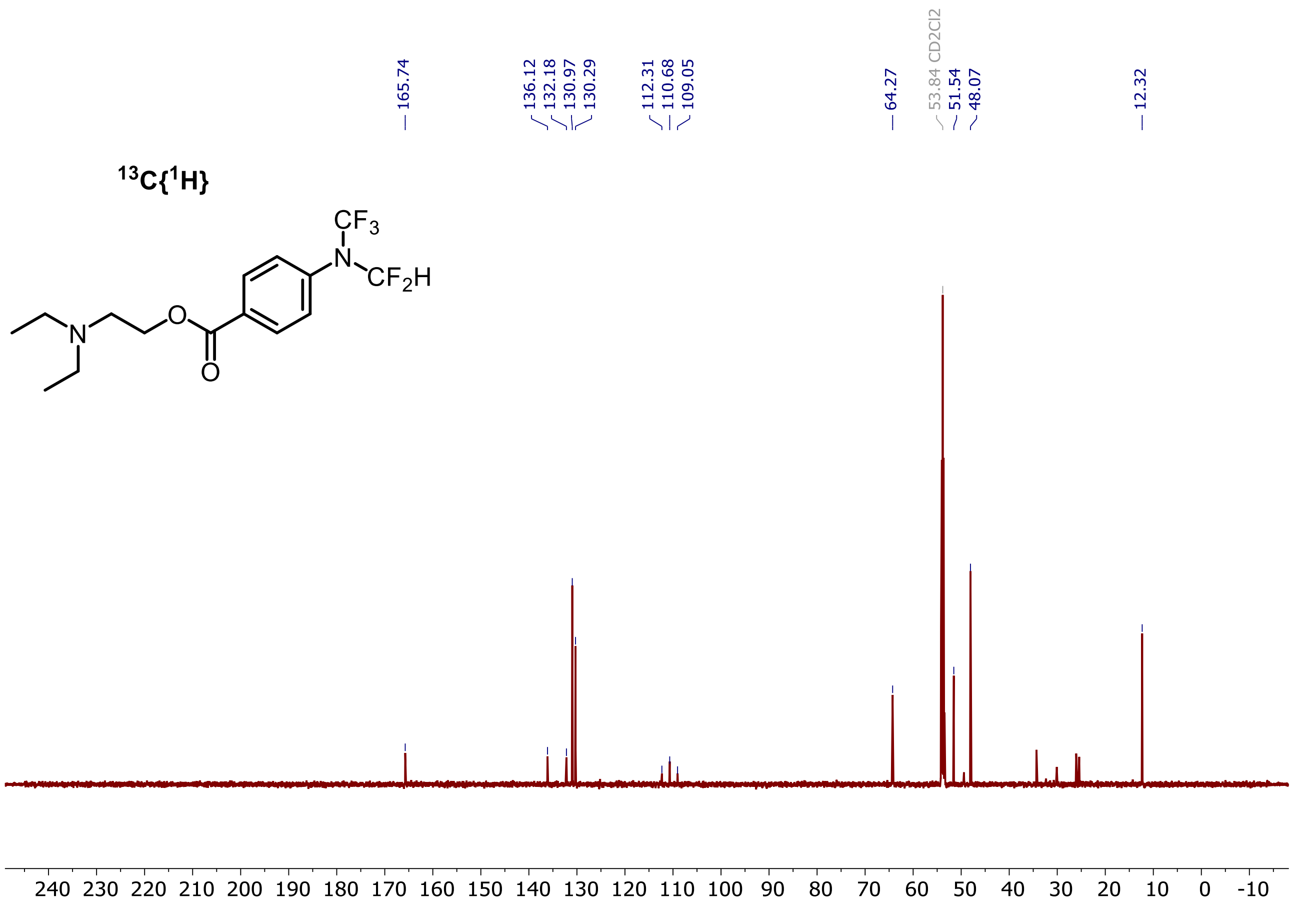
165.74
136.12
132.18
130.97
130.29
112.31
110.68
109.05
64.27
53.84 CD2Cl2
51.54
48.07
12.32
13C{1H}
CF3
N
CF2H
N
O
O
240 230 220 210 200 190 180 170 160 150 140 130 120 110 100 90 80 70 60 50 40 30 20 10 0 -10

12.2 Starting materials

12.2.1 *N*-CF_3 (deutero)thioformamides

N-([1,1'-biphenyl]-4-yl)-*N*-(trifluoromethyl)methanethioamide (*S*1)

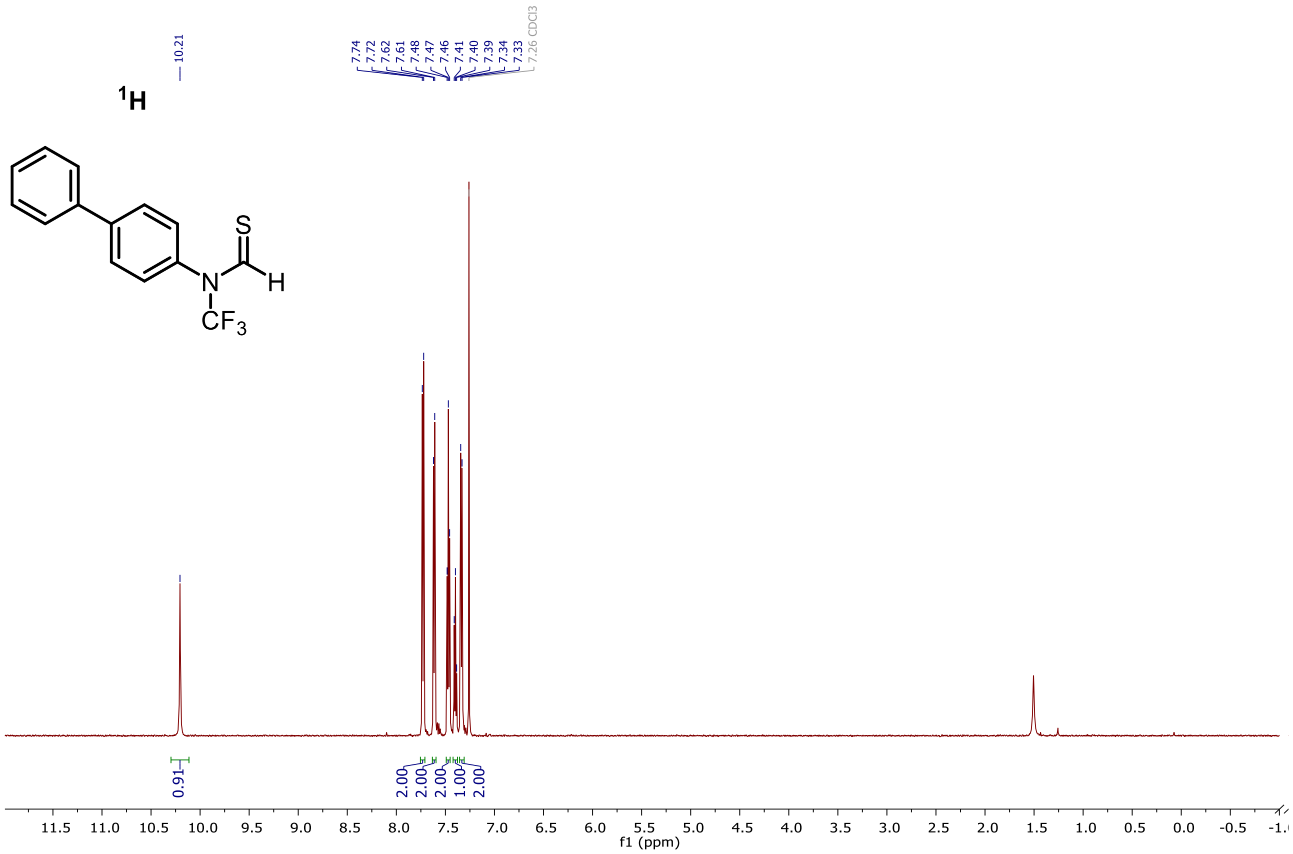

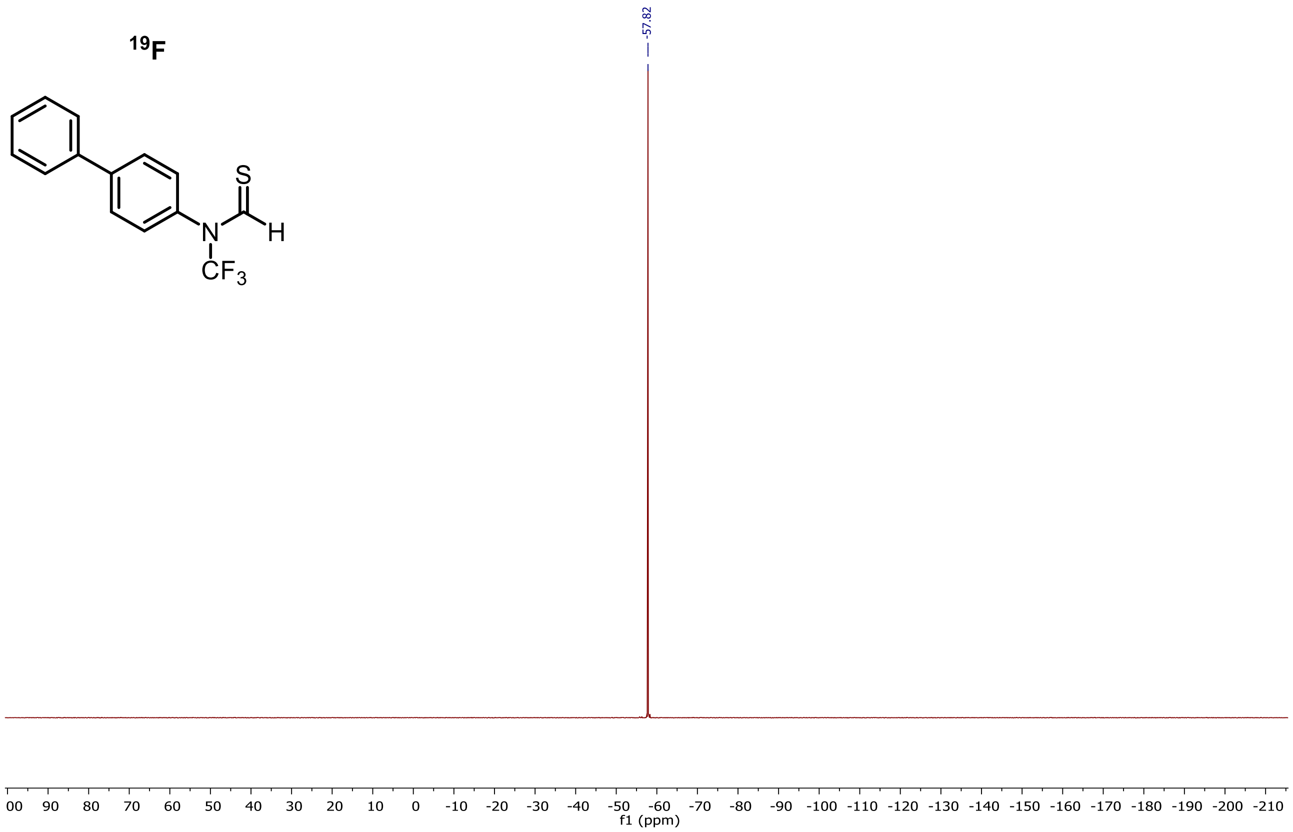

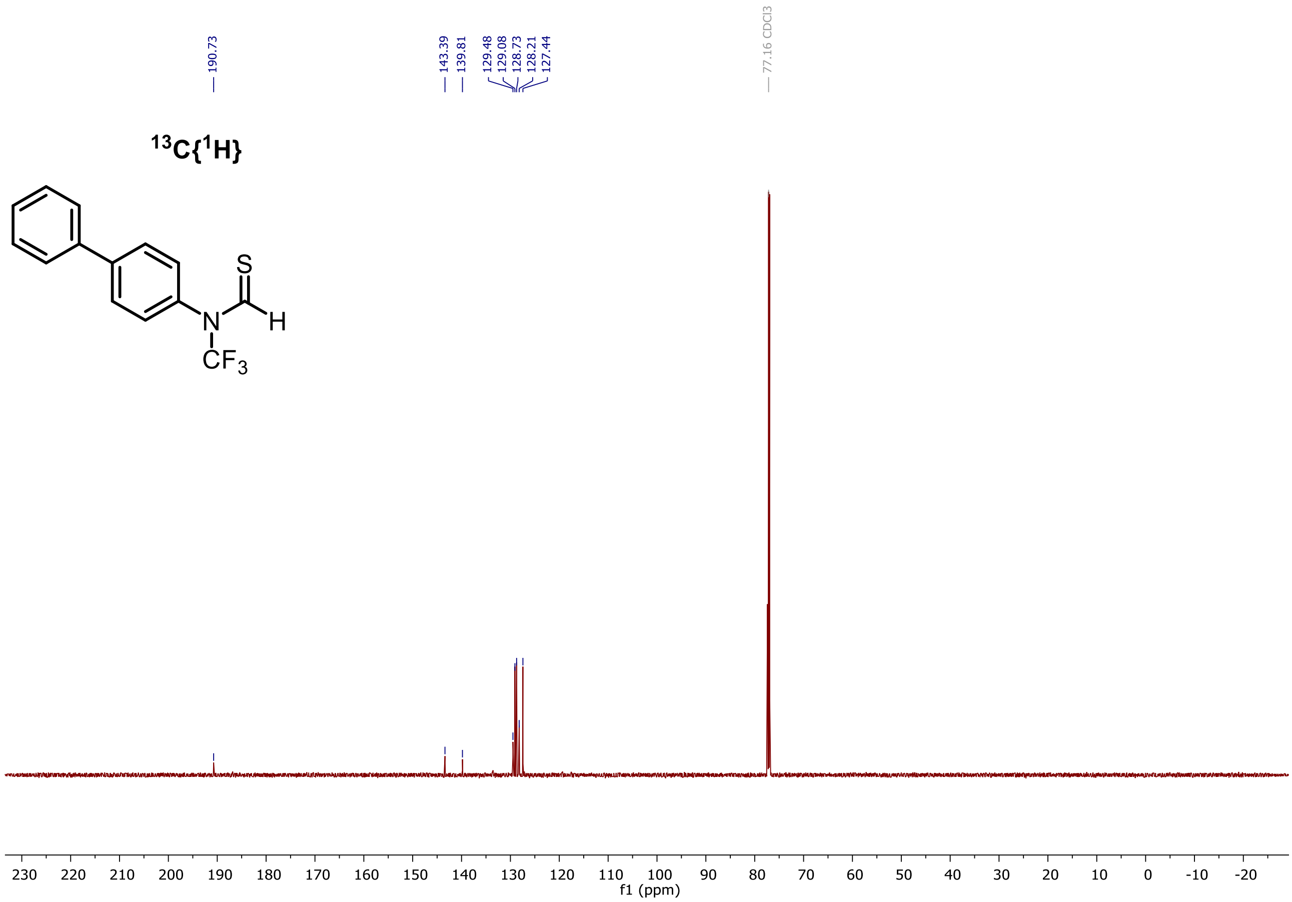

***N*-phenyl-*N*-(trifluoromethyl)methanethioamide (*S*2)**

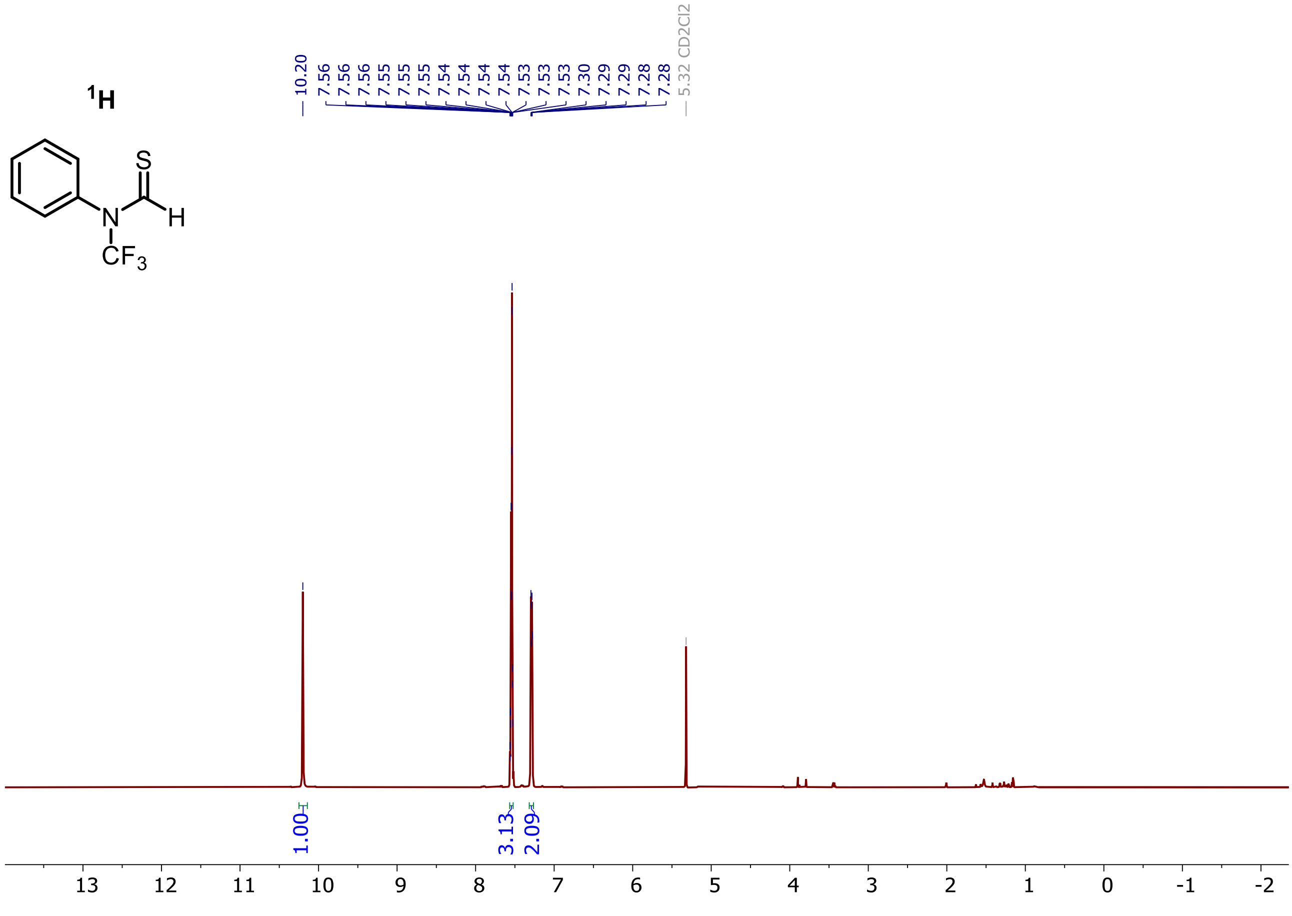

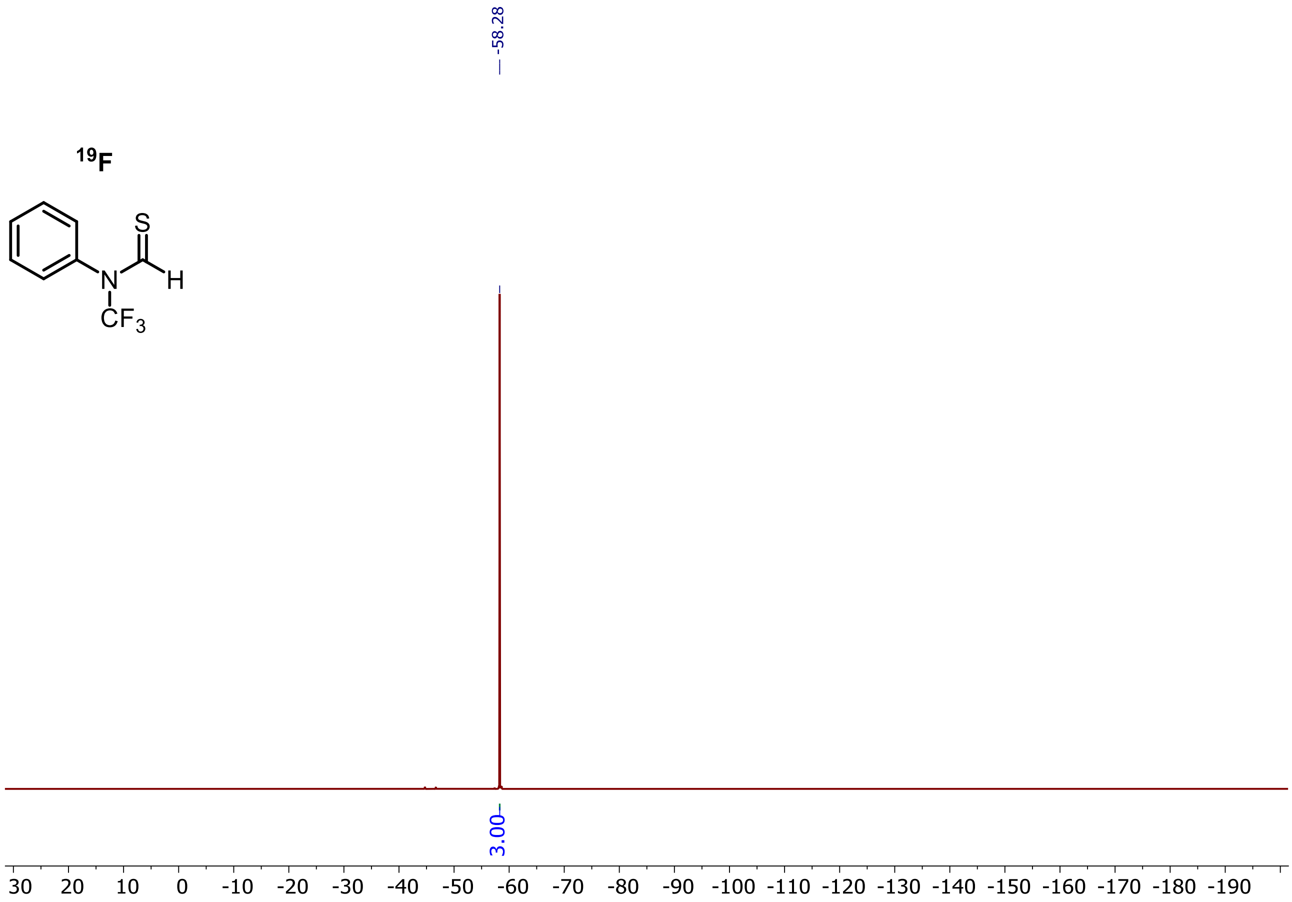
19F
-58.28
3.00
S
N
H
CF3
30 20 10 0 -10 -20 -30 -40 -50 -60 -70 -80 -90 -100 -110 -120 -130 -140 -150 -160 -170 -180 -190

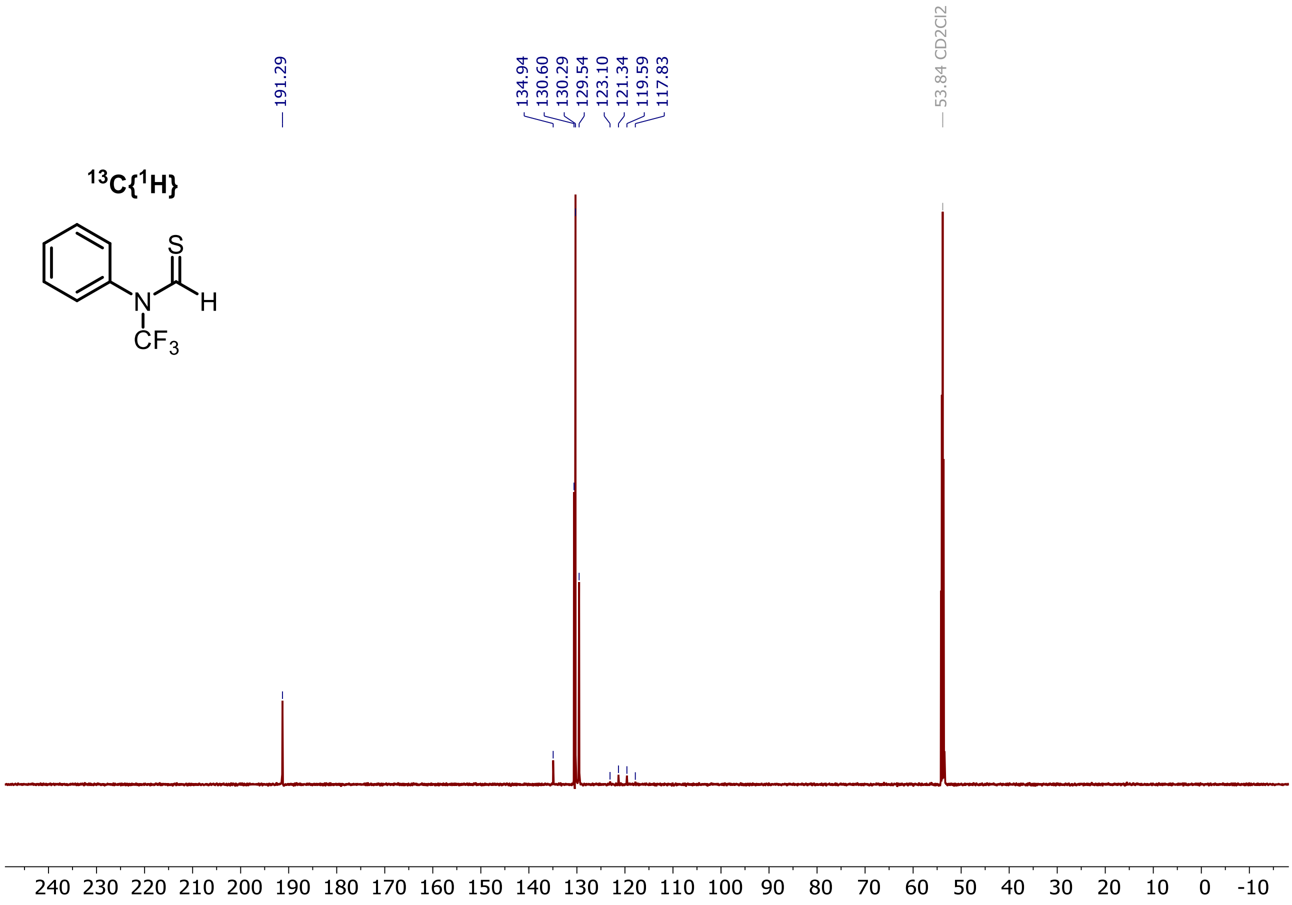
13C{1H}
191.29
134.94
130.60
130.29
129.54
123.10
121.34
119.59
117.83
53.84 CD2Cl2
S
N
H
CF3
240 230 220 210 200 190 180 170 160 150 140 130 120 110 100 90 80 70 60 50 40 30 20 10 0 -10

(4-cyclohexylphenyl)-*N*-(trifluoromethyl)methanethioamide (*S*3)

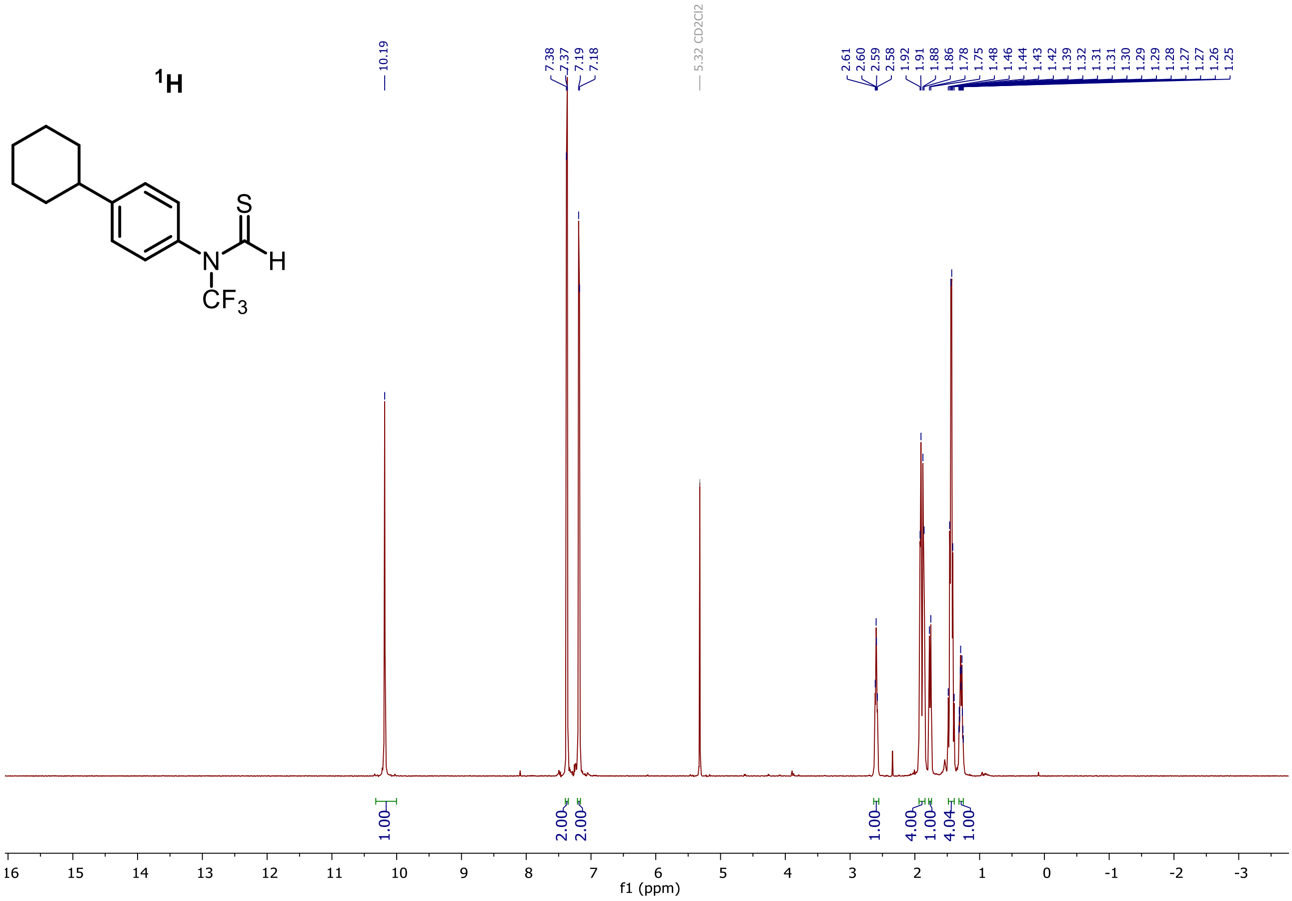

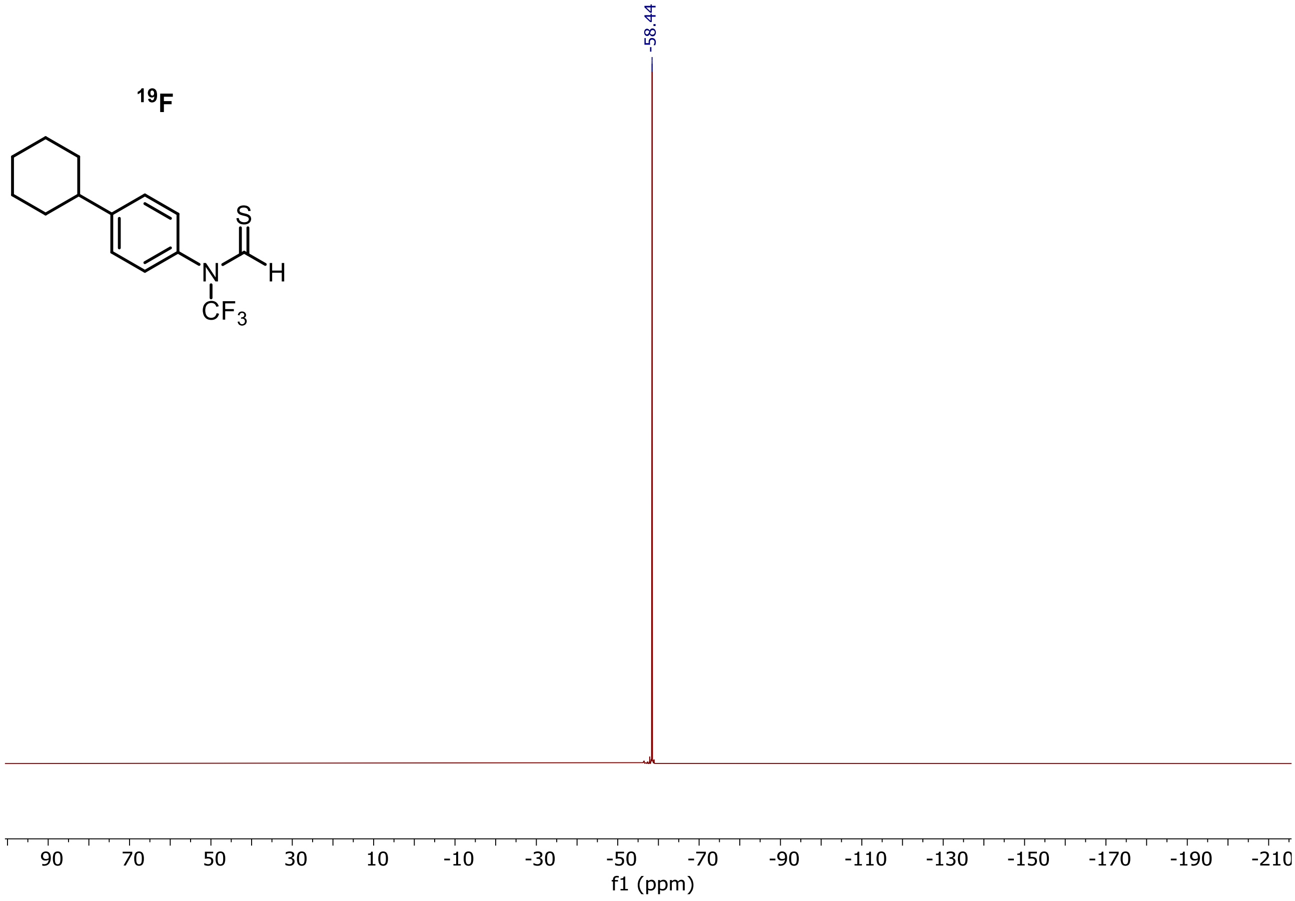

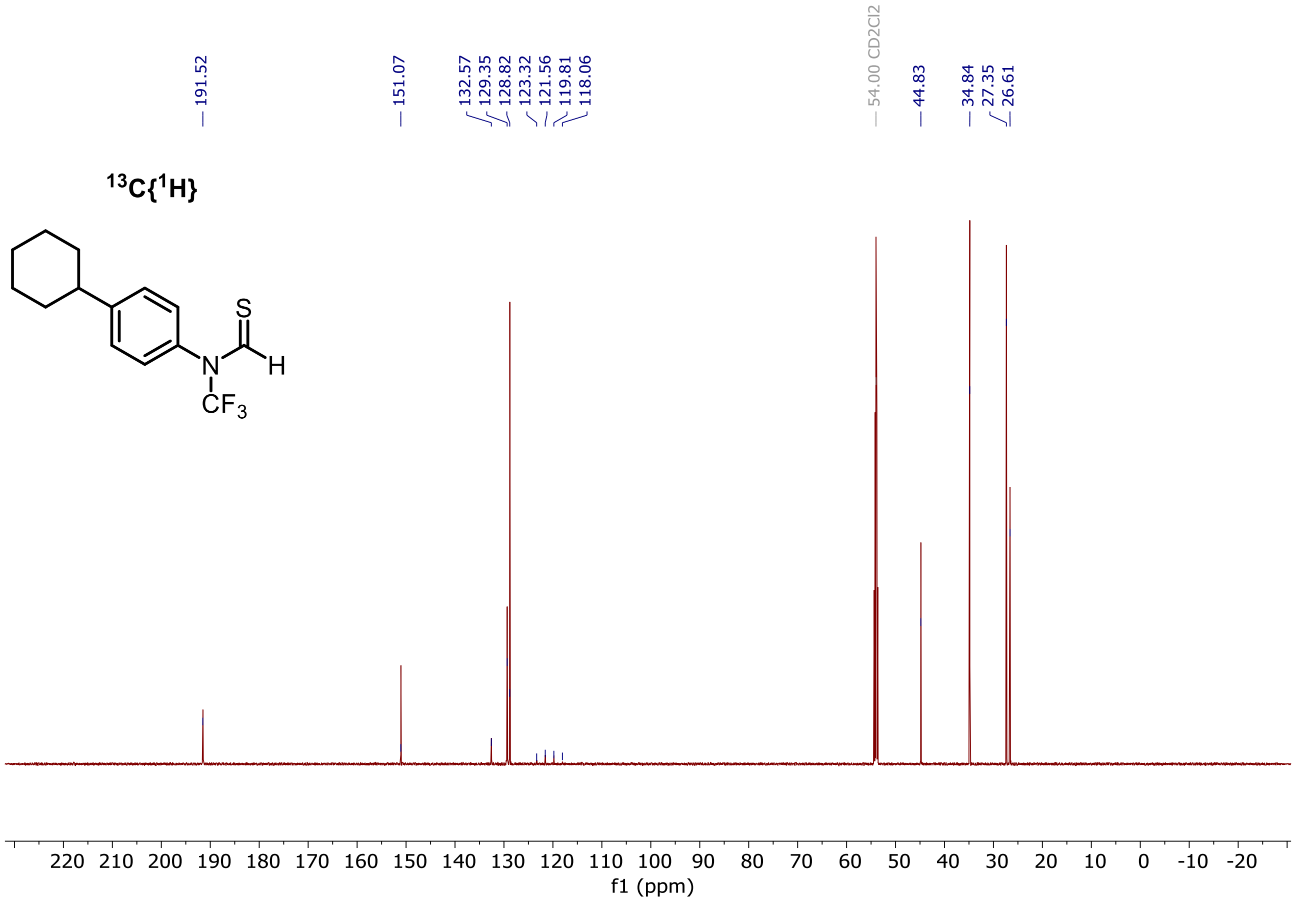

N-(trifluoromethyl)-*N*-(3,4,5-trimethoxyphenyl)methanethioamide (S4)

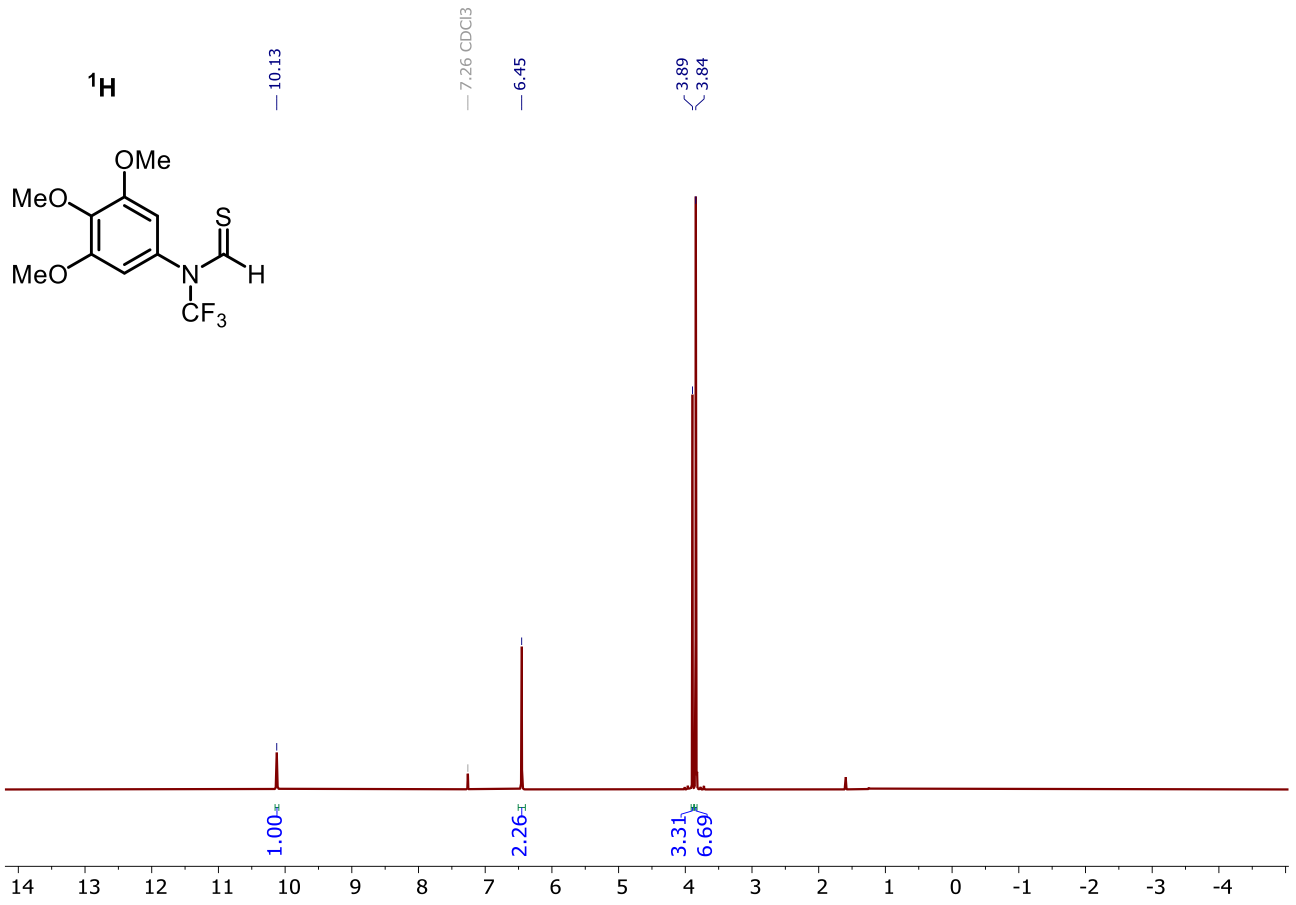

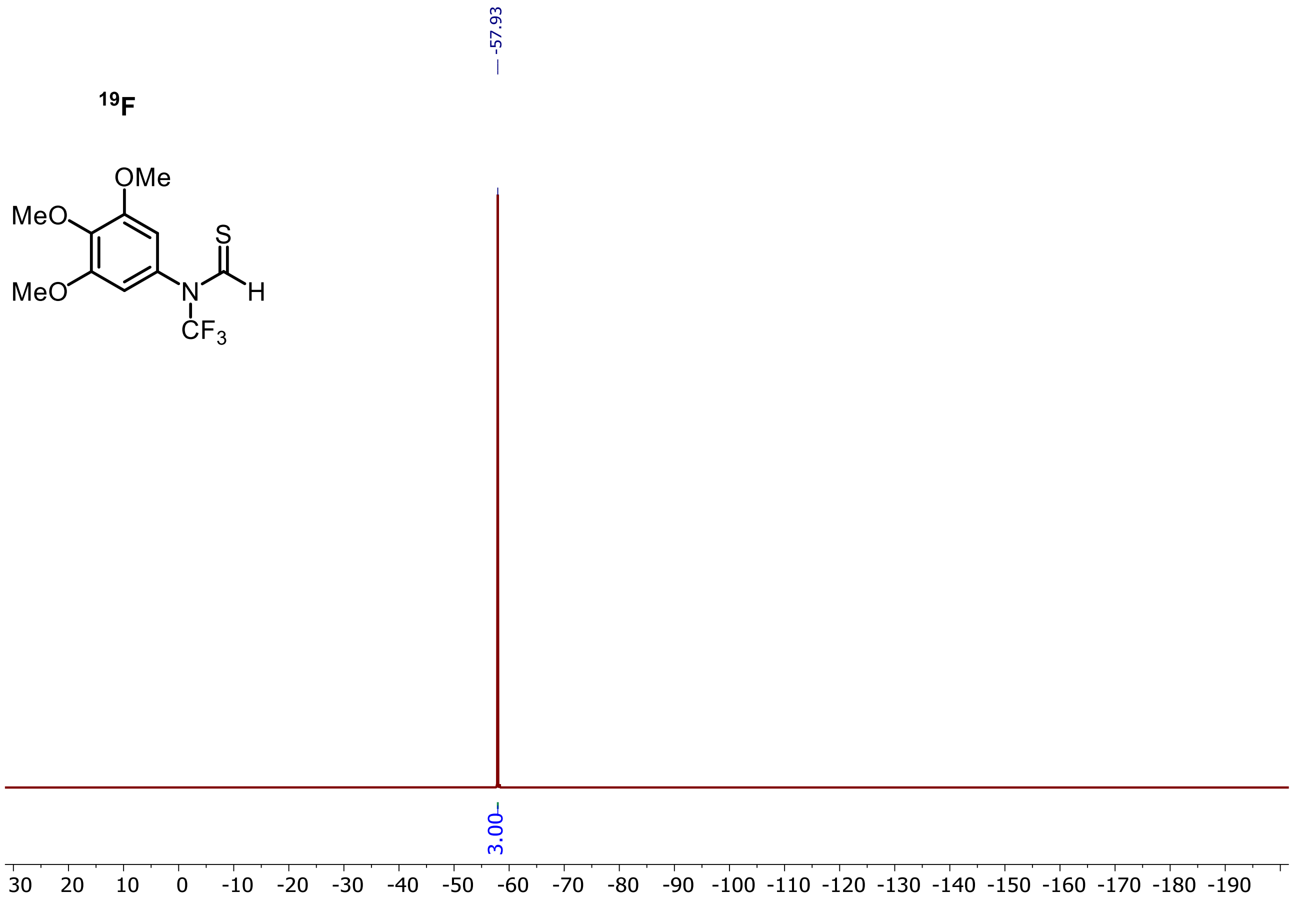

-57.93
19F
OMe
MeO
MeO
S
N
H
CF3
3.00
30 20 10 0 -10 -20 -30 -40 -50 -60 -70 -80 -90 -100 -110 -120 -130 -140 -150 -160 -170 -180 -190

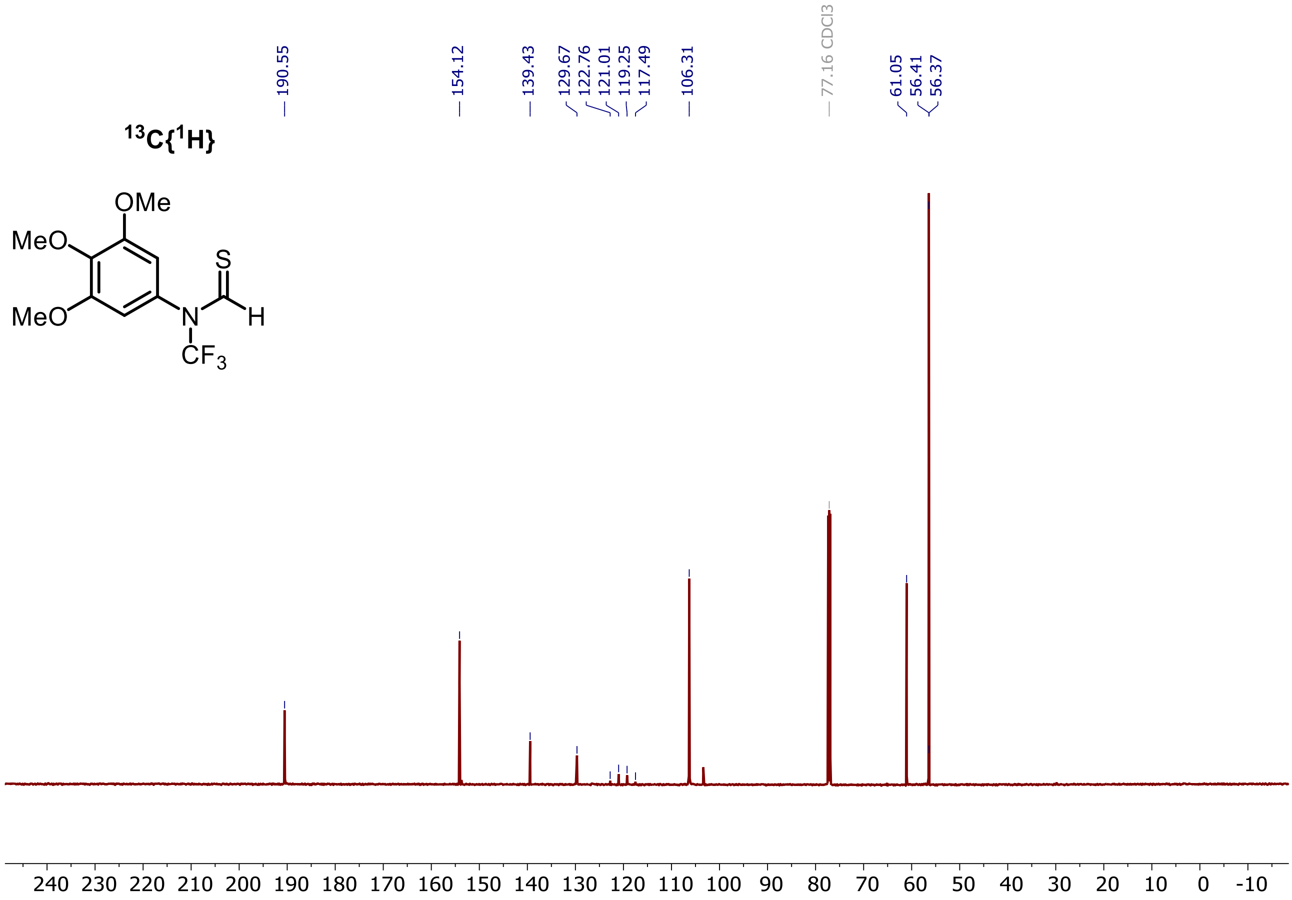

190.55
154.12
139.43
129.67
122.76
121.01
119.25
117.49
106.31
77.16 CDCl3
61.05
56.41
56.37
13C{1H}
OMe
MeO
MeO
S
N
H
CF3
240 230 220 210 200 190 180 170 160 150 140 130 120 110 100 90 80 70 60 50 40 30 20 10 0 -10

_N_-(4-bromo-2-chlorophenyl)-_N_-(trifluoromethyl)methanethioamide (_S_5)

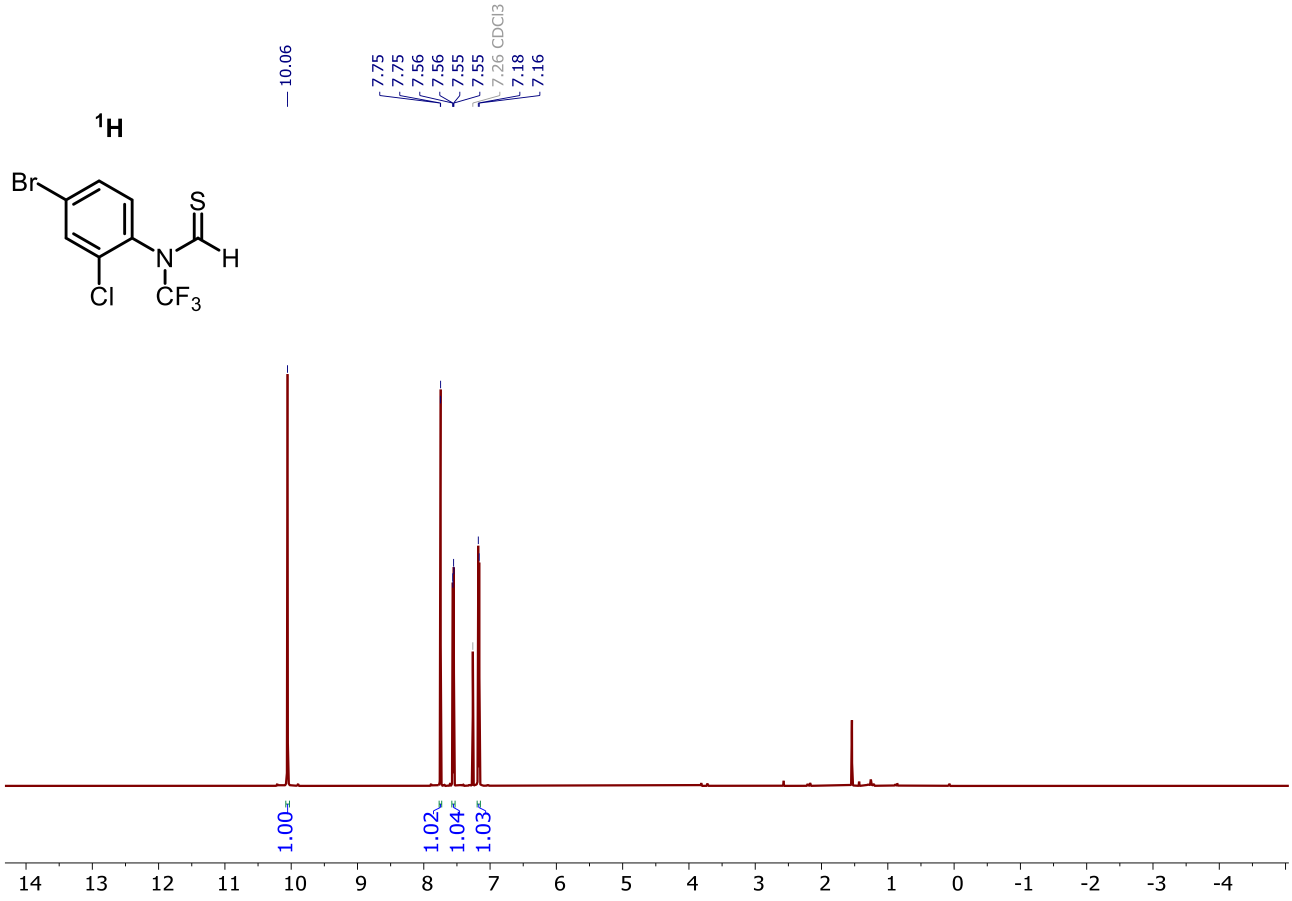

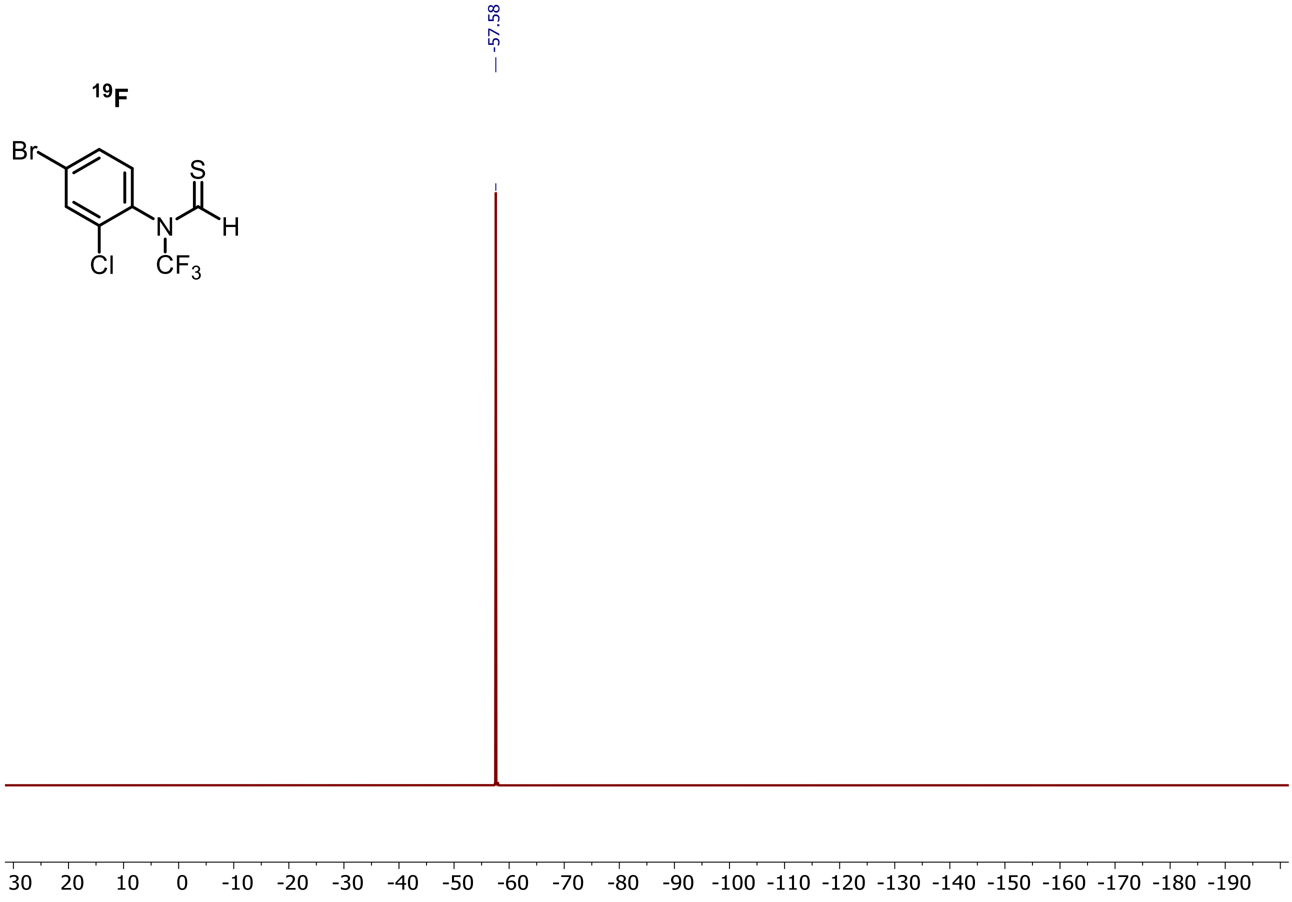

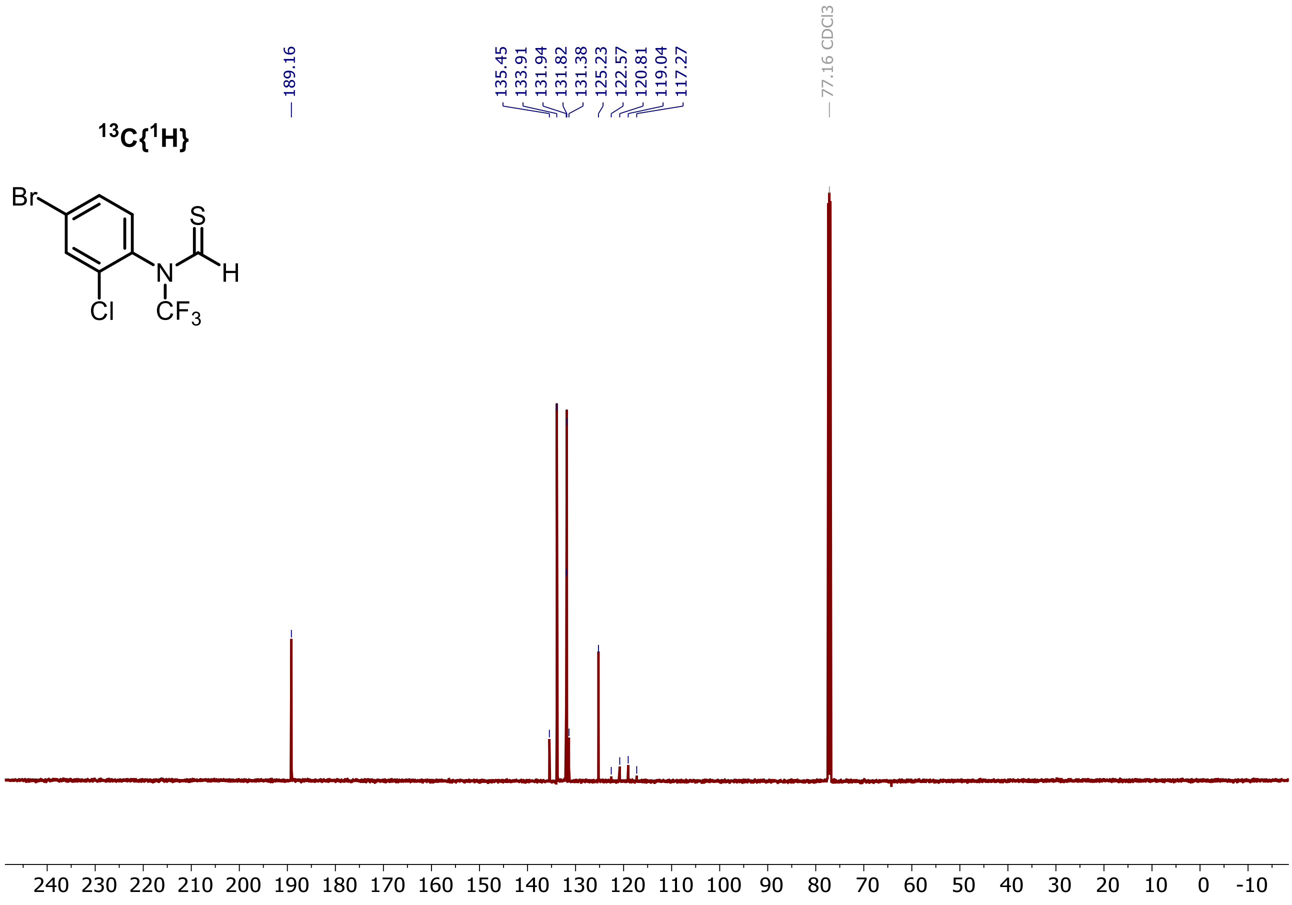

methyl 4-(*N*-(trifluoromethyl)methanethioamido)benzoate (*S6*)

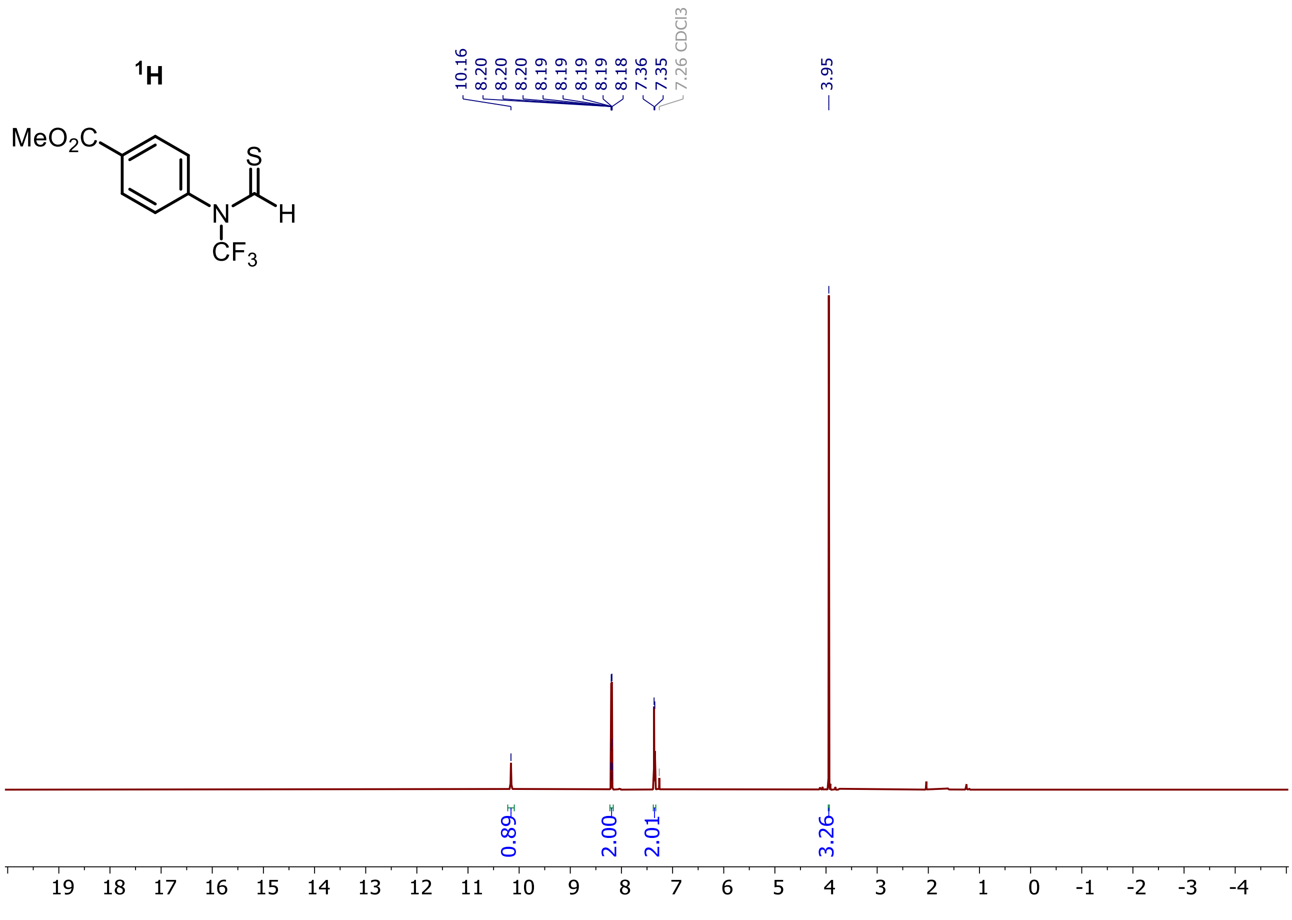

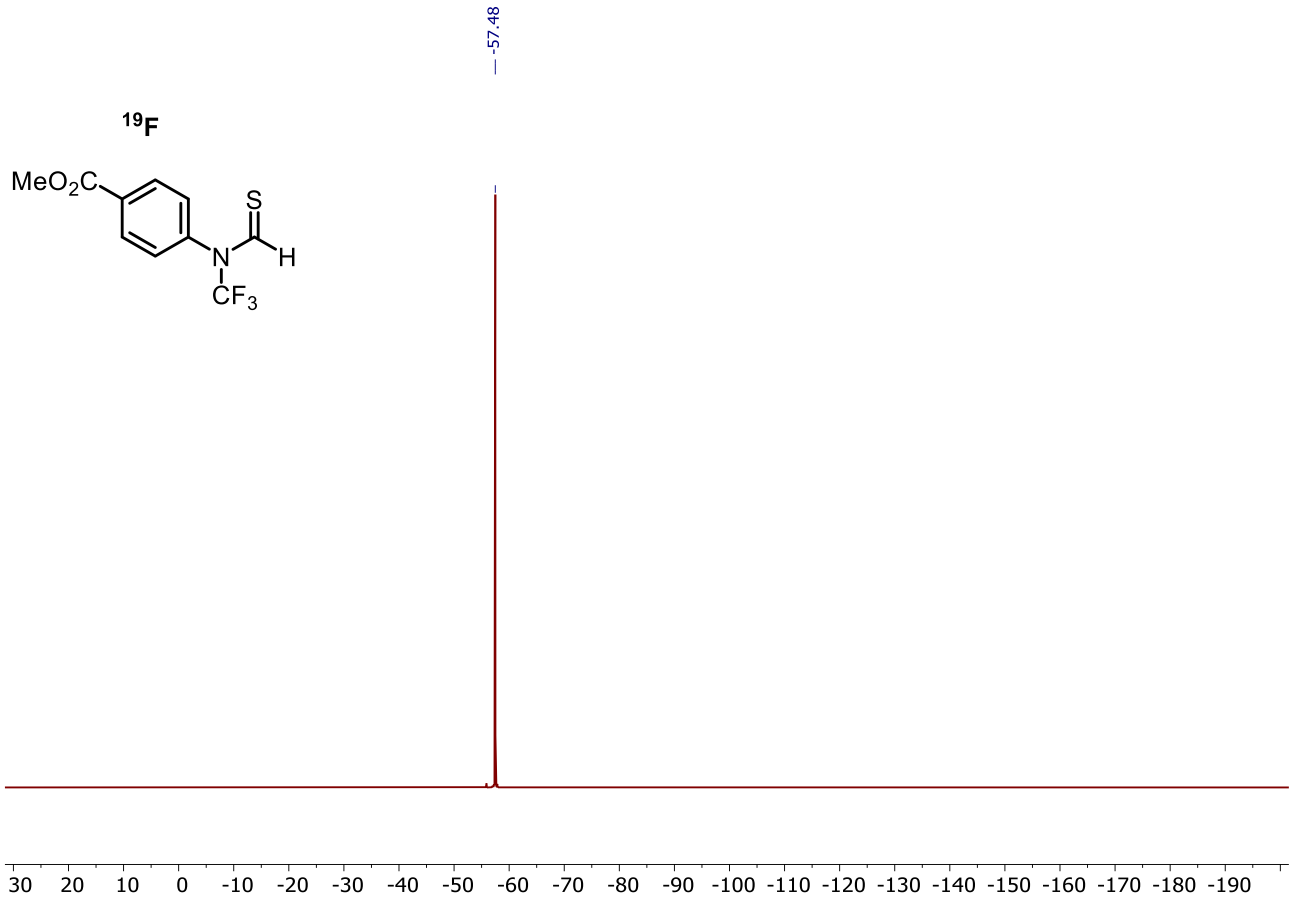
-57.48
19F
MeO2C
S
N
H
CF3
30 20 10 0 -10 -20 -30 -40 -50 -60 -70 -80 -90 -100 -110 -120 -130 -140 -150 -160 -170 -180 -190

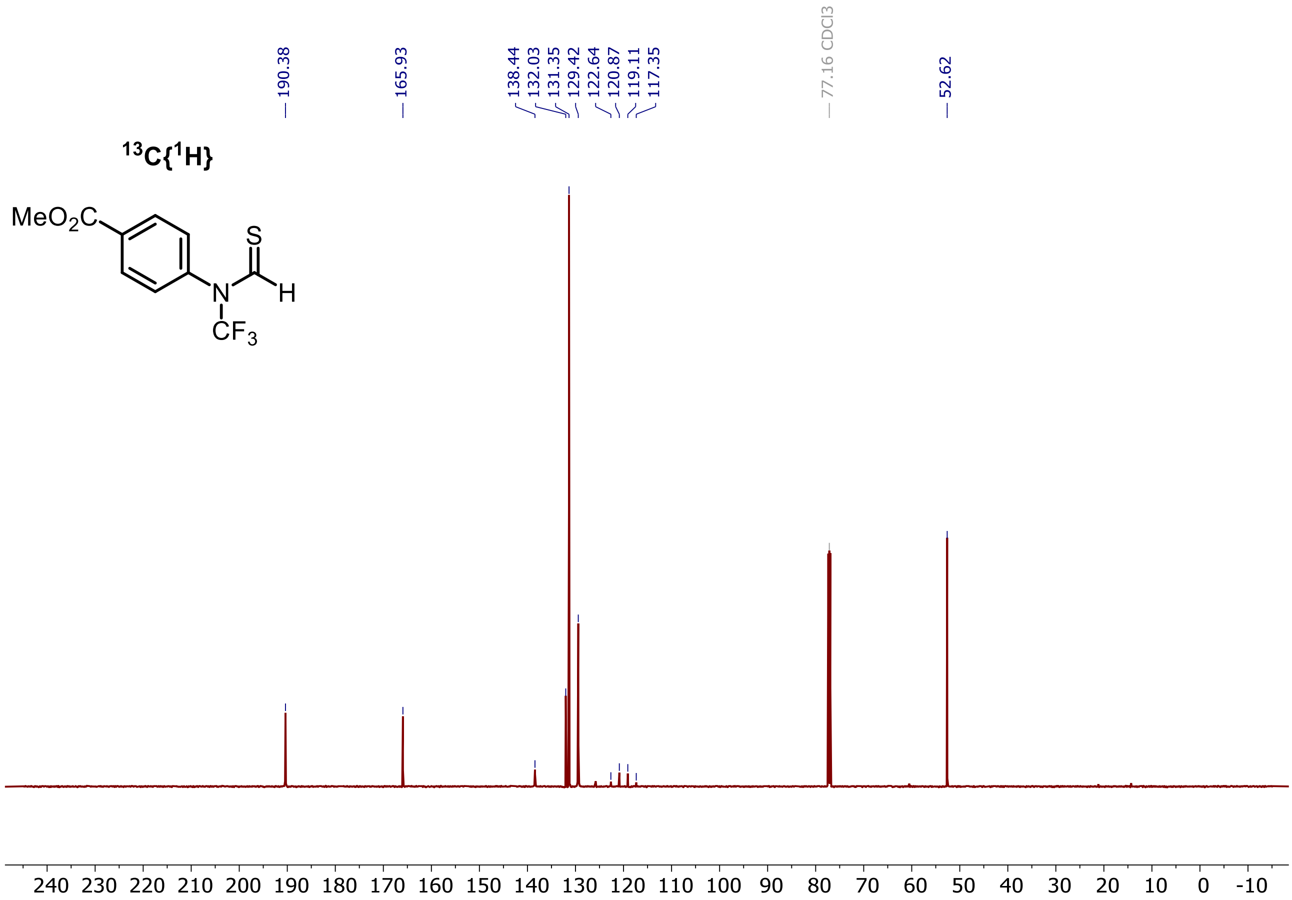
190.38
165.93
138.44
132.03
131.35
129.42
122.64
120.87
119.11
117.35
77.16 CDCl3
52.62
13C{1H}
MeO2C
S
N
H
CF3
240 230 220 210 200 190 180 170 160 150 140 130 120 110 100 90 80 70 60 50 40 30 20 10 0 -10

N-([1,1'-biphenyl]-4-yl)-*N*-(trifluoromethyl)methanethioamide-*d* (*S*7)

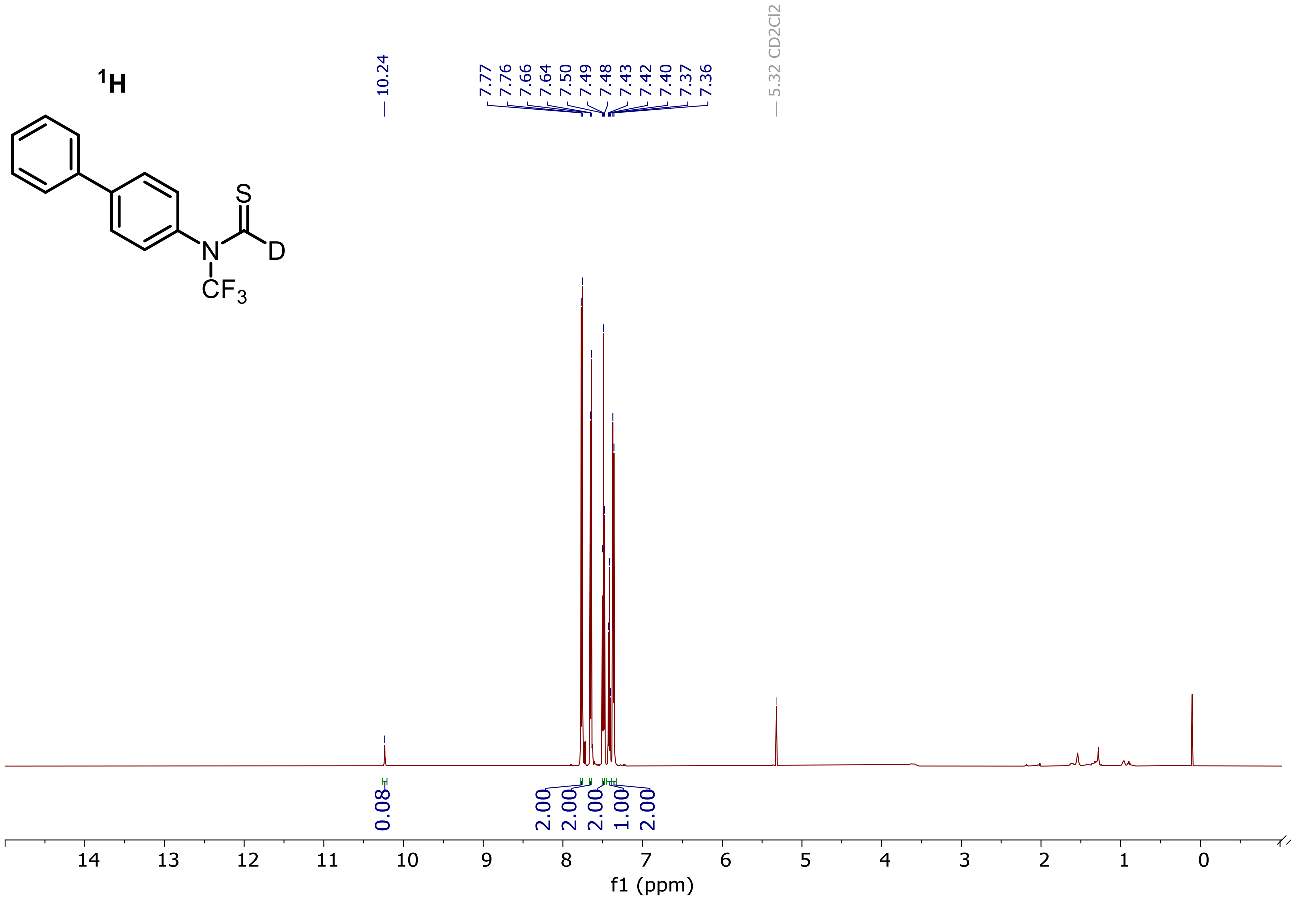

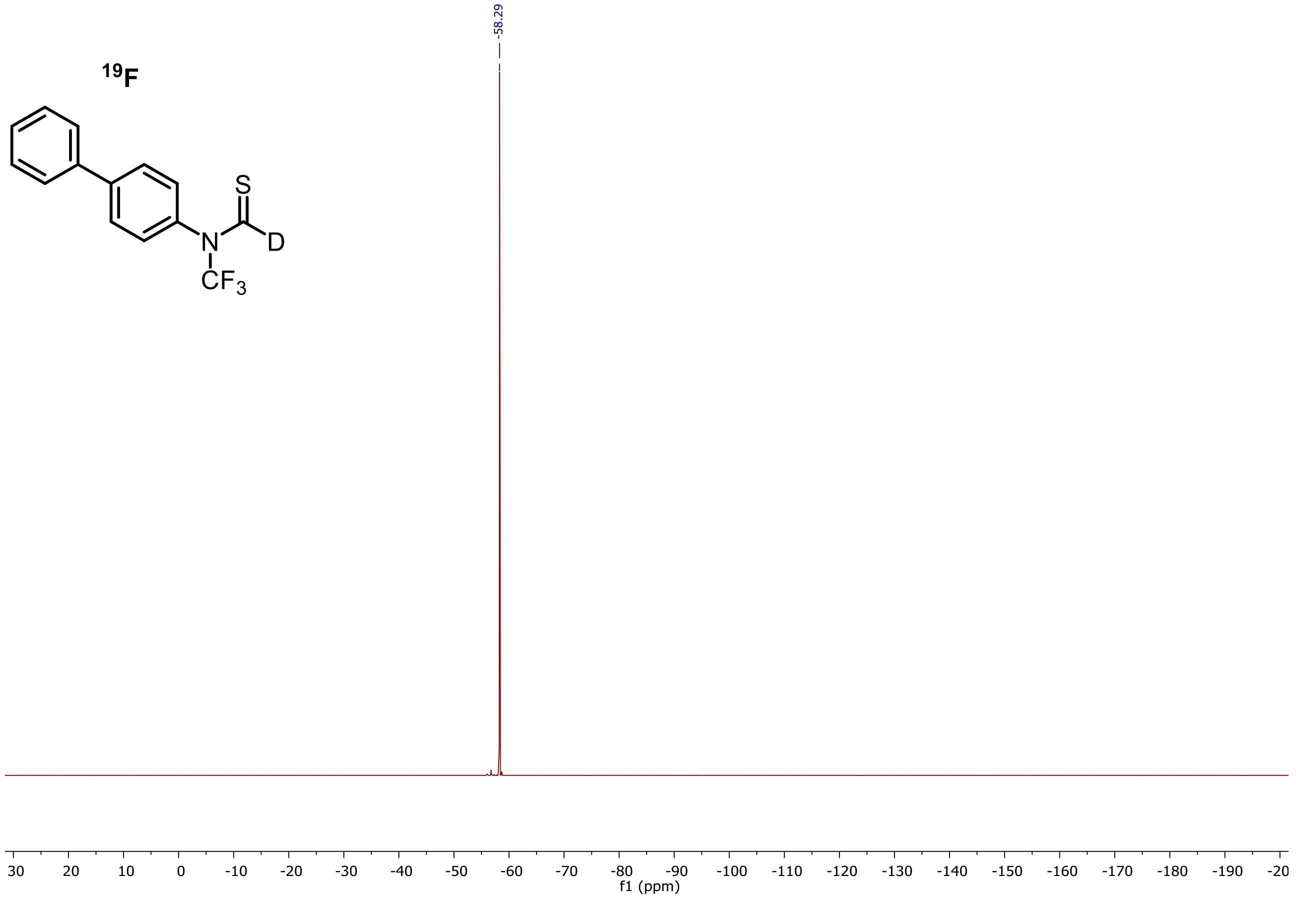

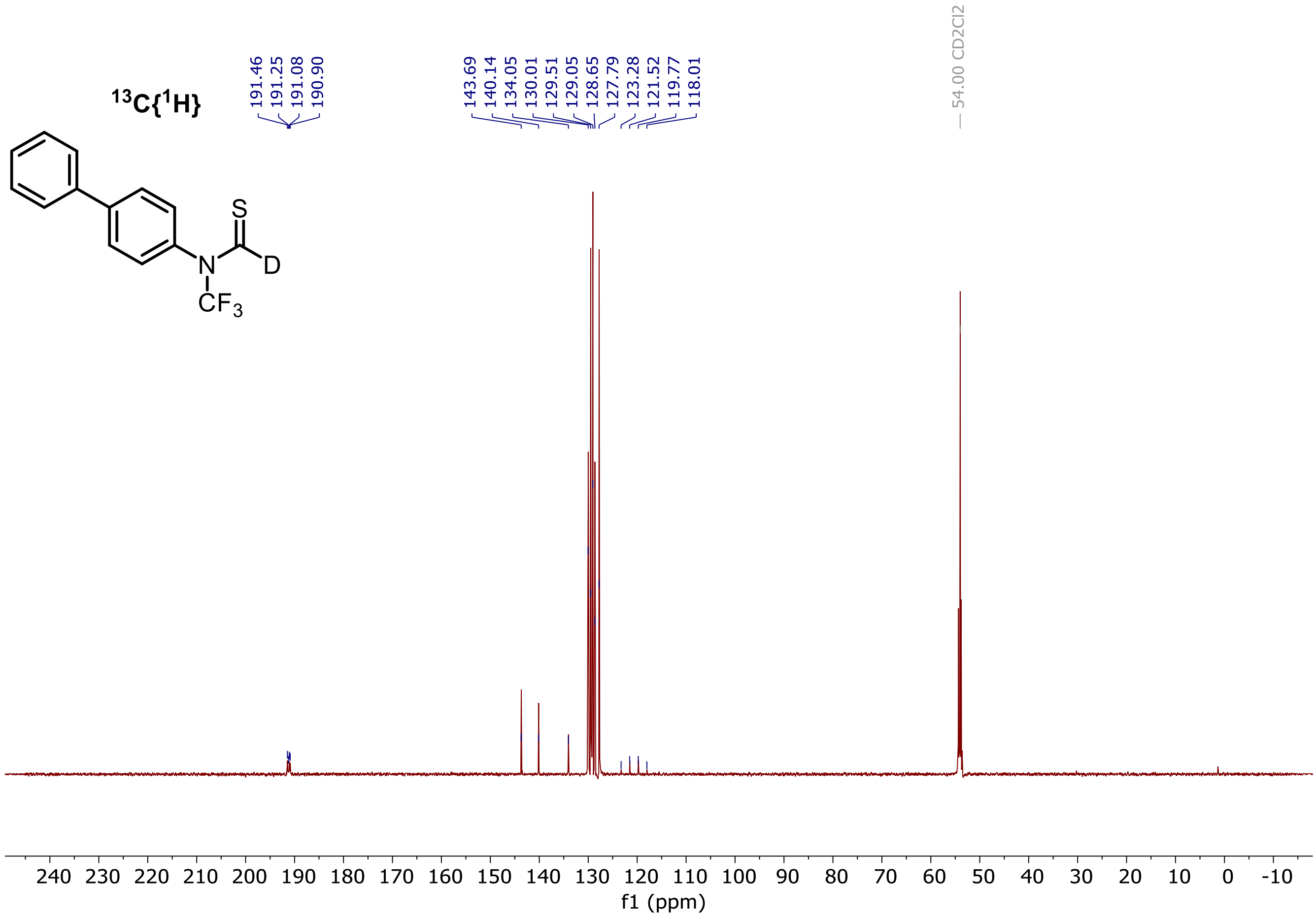

tert-butyl 4-(*N*-(trifluoromethyl)methanethioamido)piperidine-1-carboxylate (*S8*)

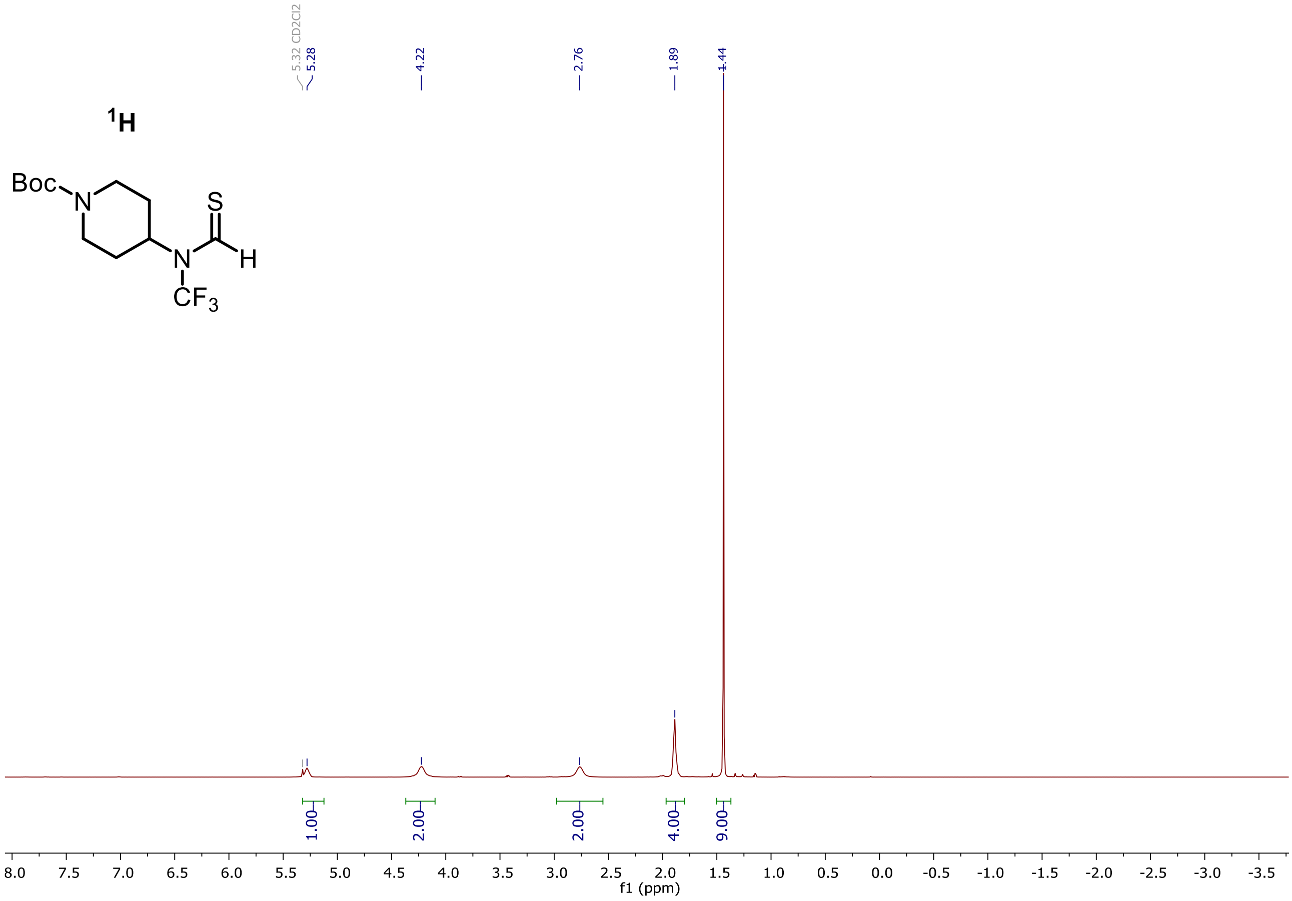

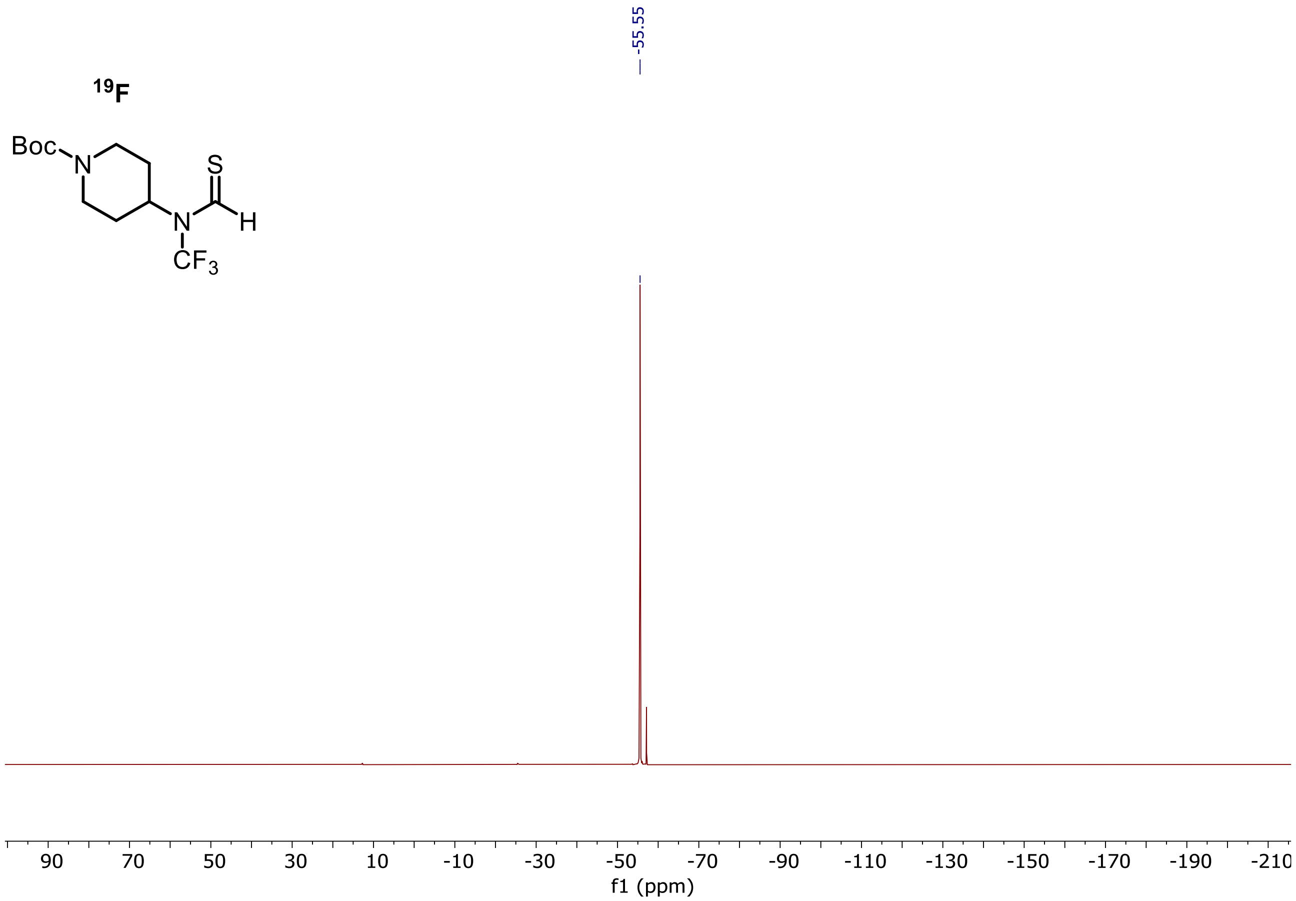

-55.55
19F
Boc
N
S
H
CF3
90
70
50
30
10
-10
-30
-50
-70
-90
-110
-130
-150
-170
-190
-210
f1 (ppm)

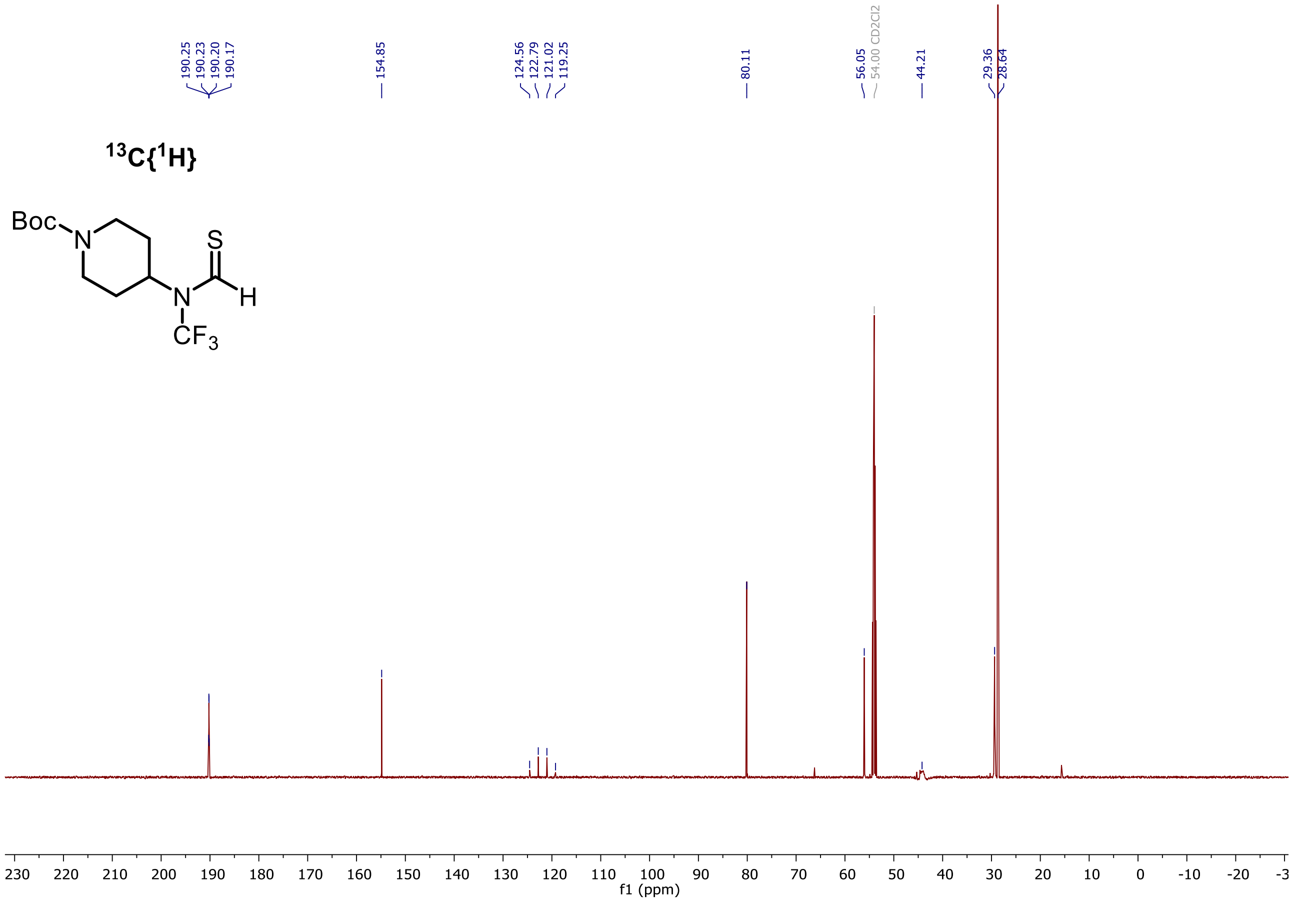

190.25
190.23
190.20
190.17
154.85
124.56
122.79
121.02
119.25
80.11
56.05
54.00 CD2Cl2
44.21
29.36
28.64
13C{1H}
Boc
N
S
H
CF3
230
220
210
200
190
180
170
160
150
140
130
120
110
100
90
80
70
60
50
40
30
20
10
0
-10
-20
f1 (ppm)

tert-butyl *N*-thioformyl-*N*-(trifluoromethyl)-*L*-phenylalaninate (*S*9)

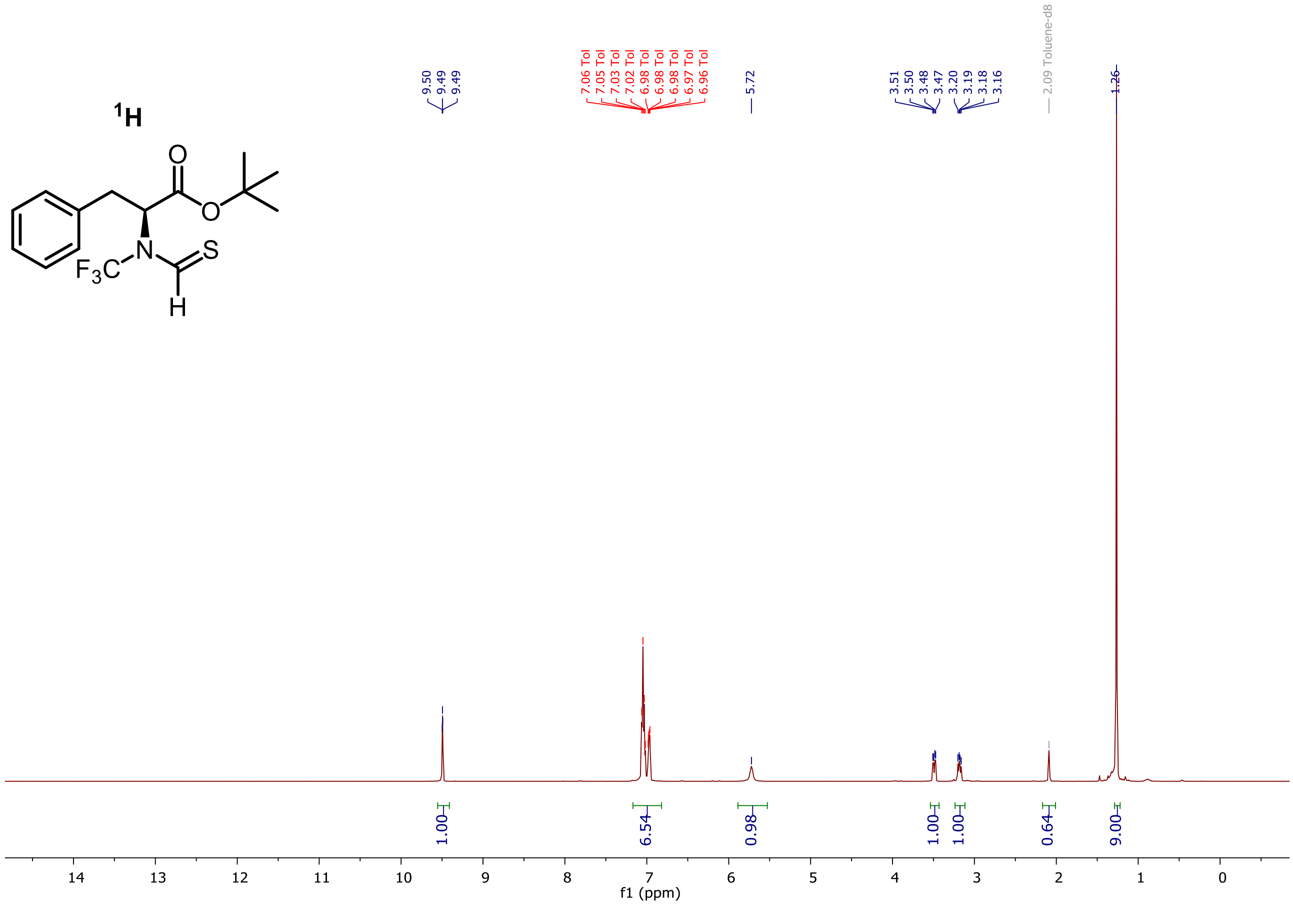

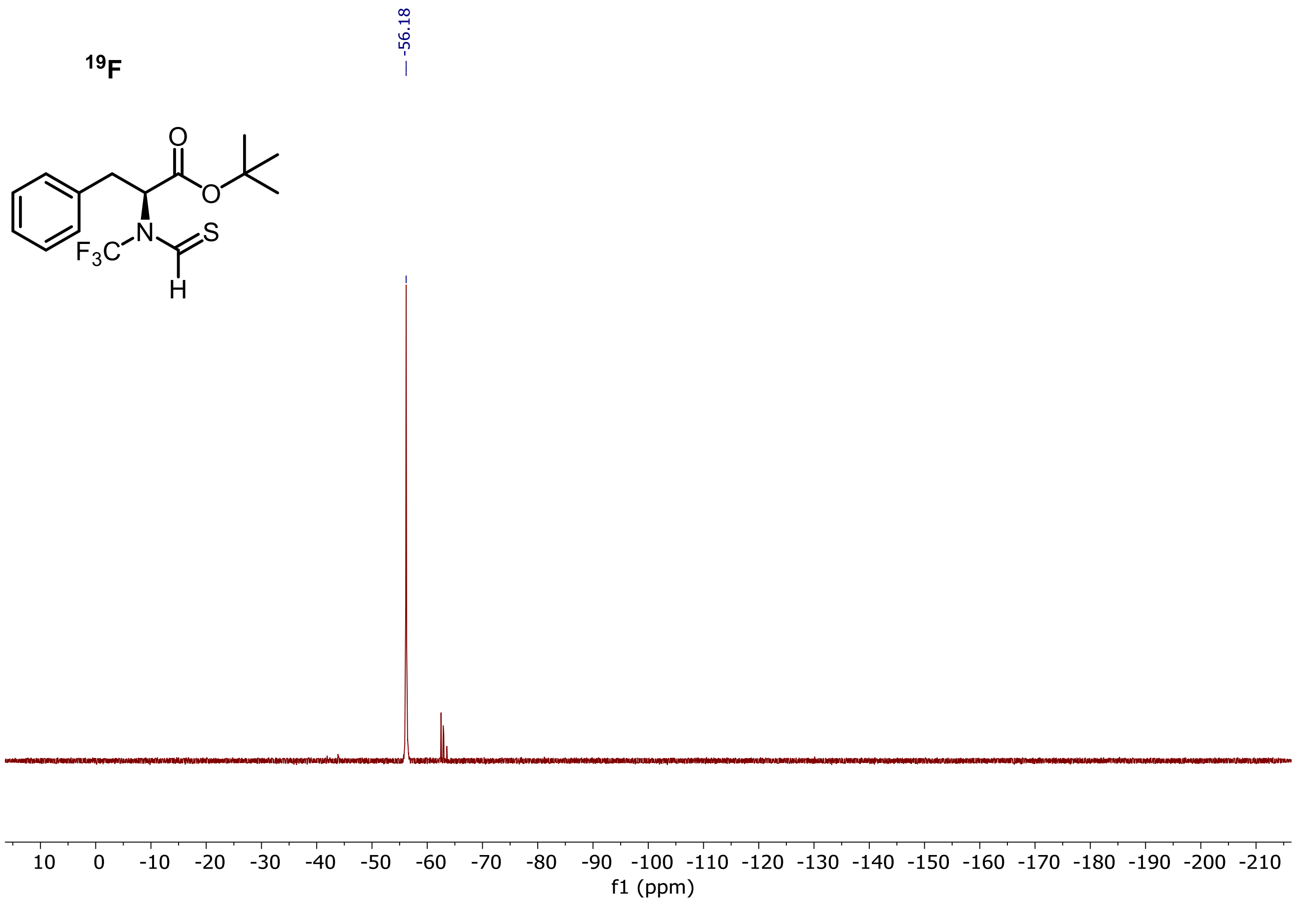

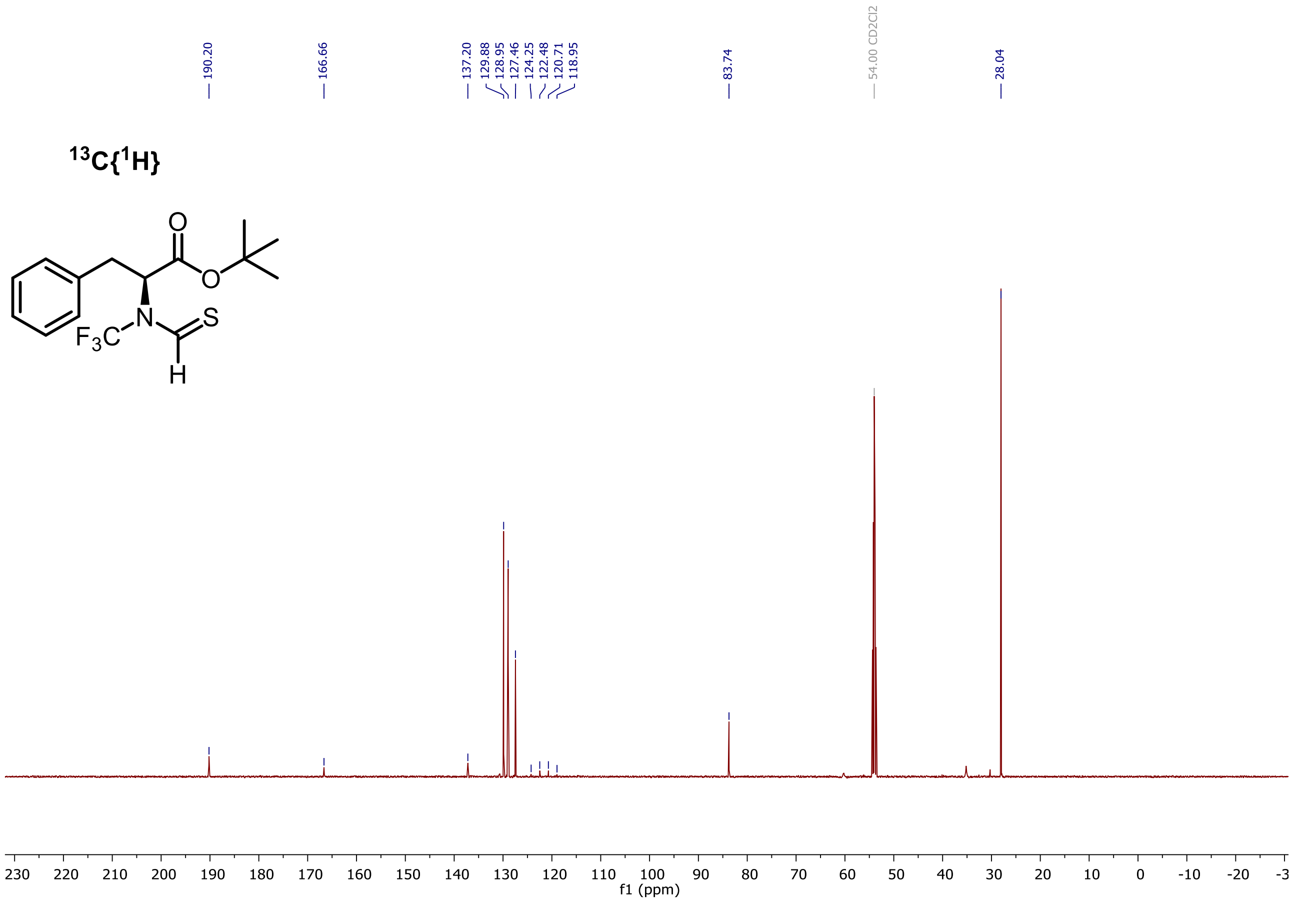

benzyl *N*-thioformyl-*N*-(trifluoromethyl)-*L*-leucinate (*S*10)

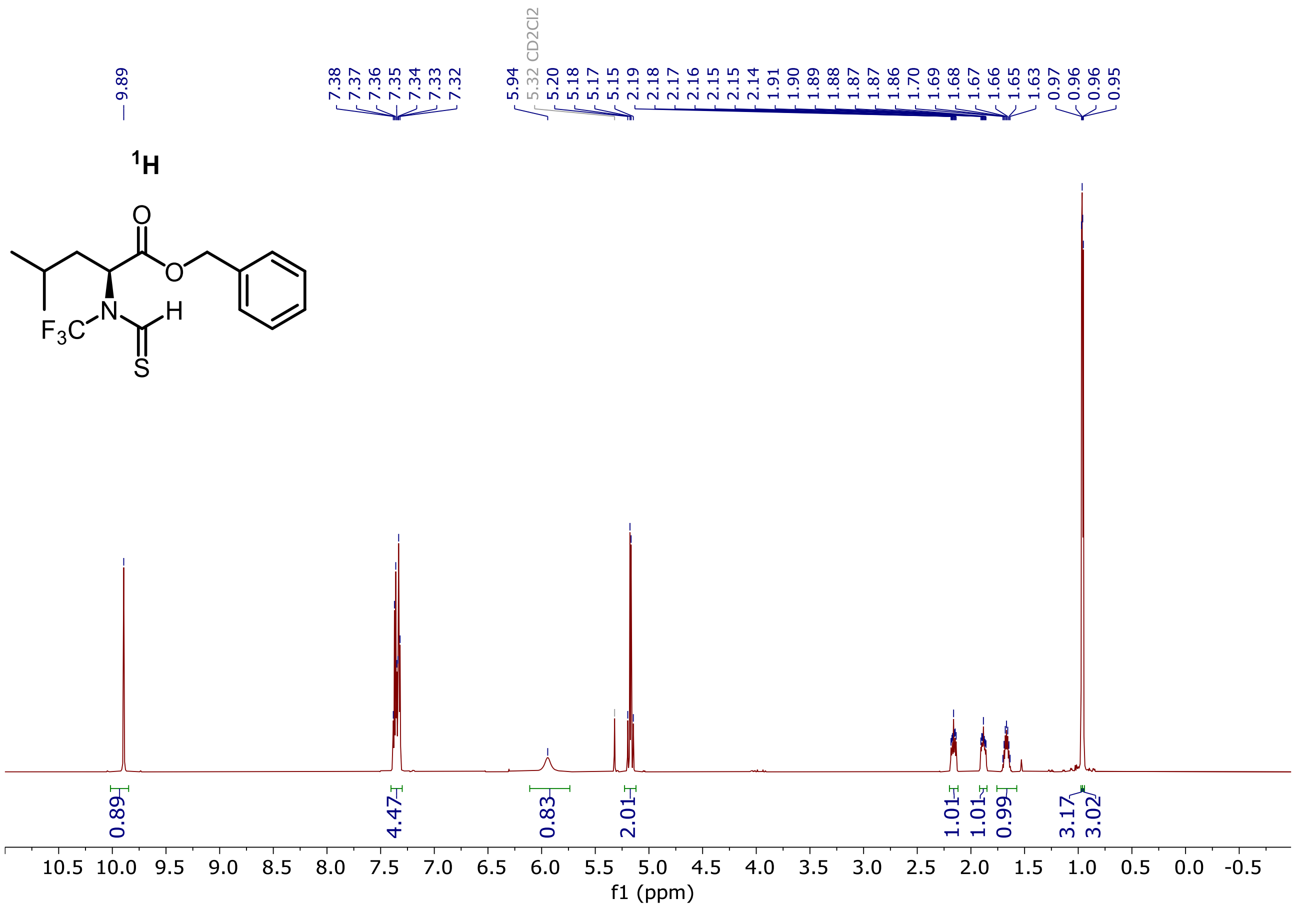

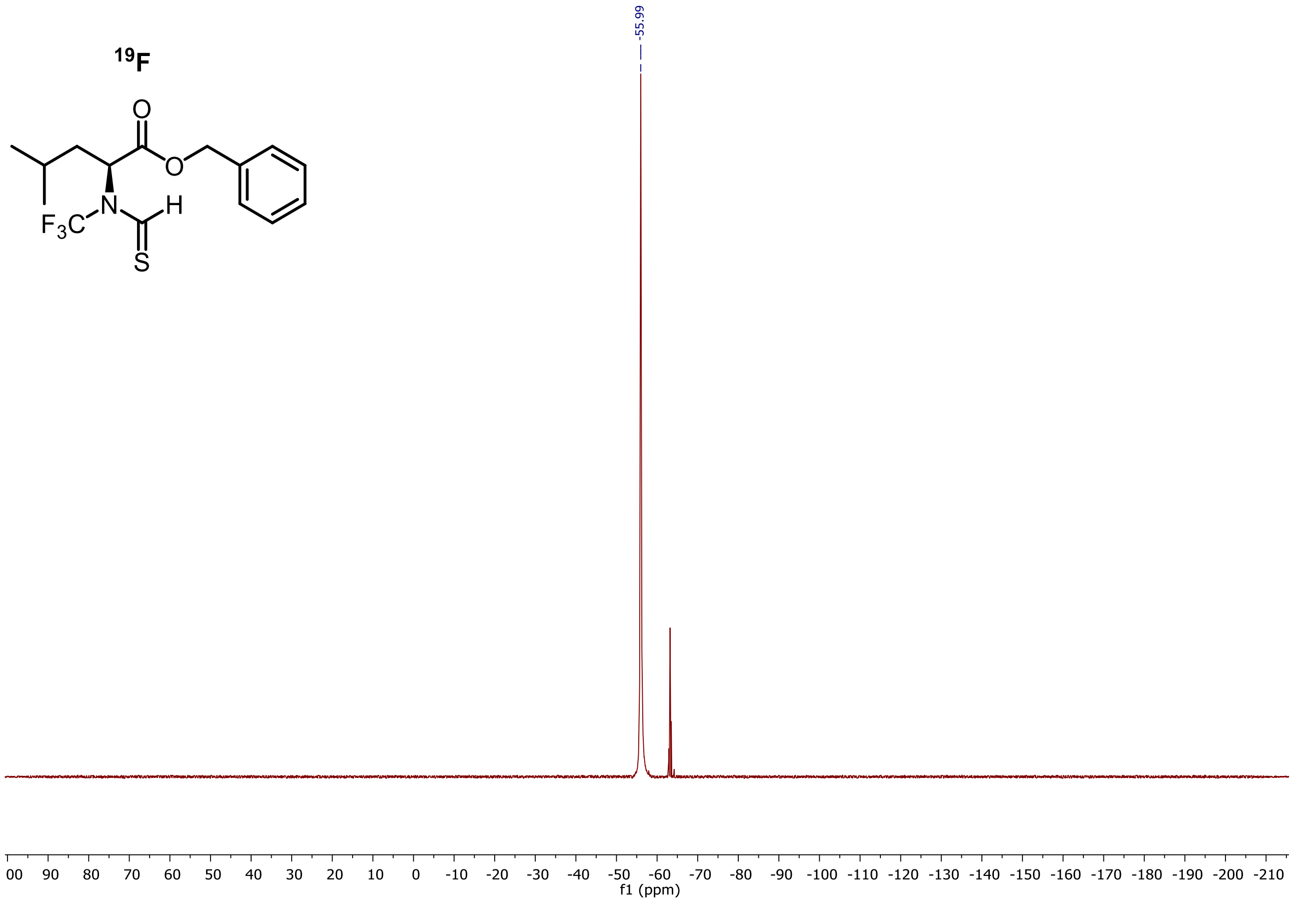
19F
O
O
N
H
F3C
S
-55.99
00 90 80 70 60 50 40 30 20 10 0 -10 -20 -30 -40 -50 -60 -70 -80 -90 -100 -110 -120 -130 -140 -150 -160 -170 -180 -190 -200 -210
f1 (ppm)

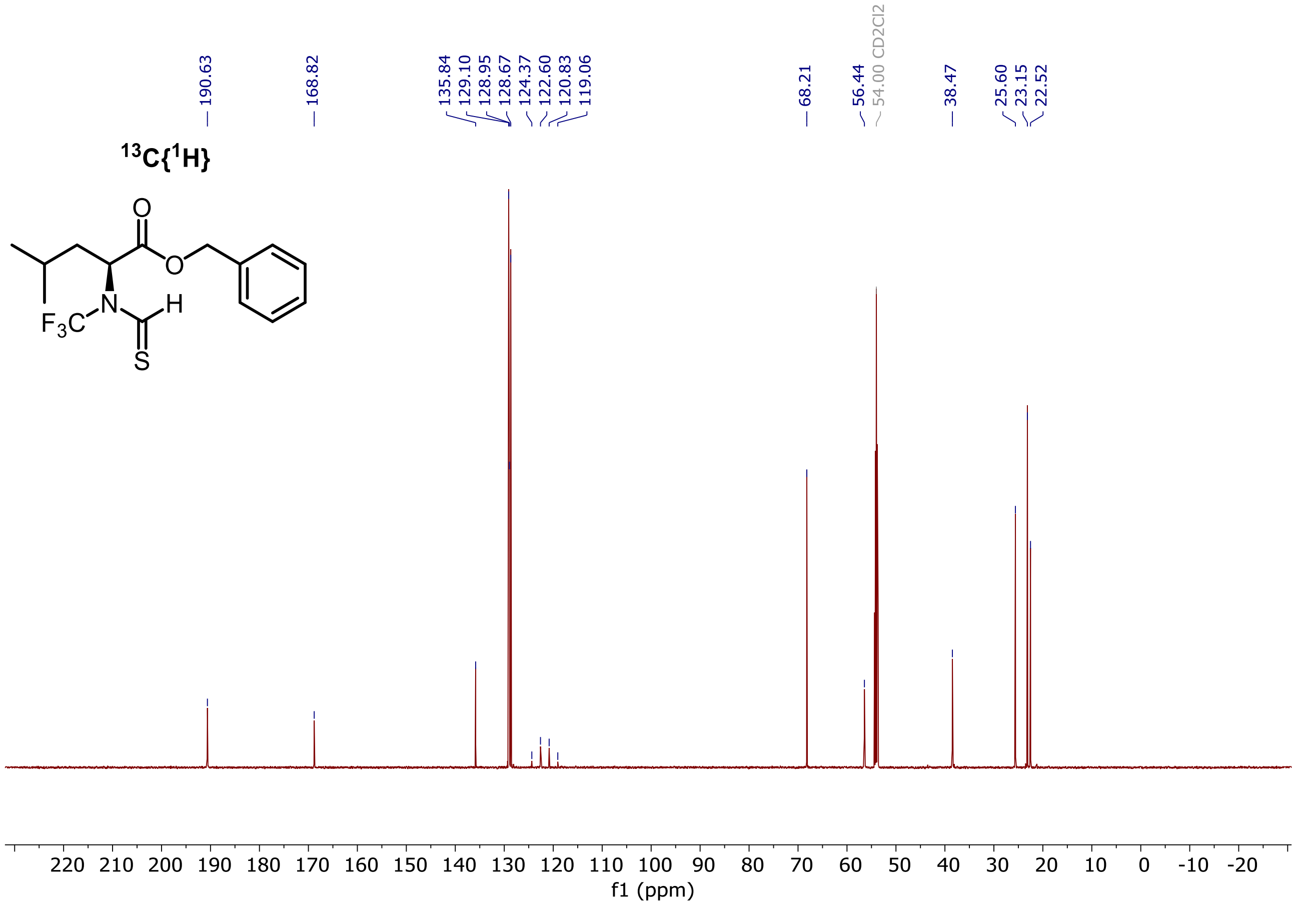
13C{1H}
O
O
N
H
F3C
S
190.63
168.82
135.84
129.10
128.95
128.67
124.37
122.60
120.83
119.06
68.21
56.44
54.00 CD2Cl2
38.47
25.60
23.15
22.52
220 210 200 190 180 170 160 150 140 130 120 110 100 90 80 70 60 50 40 30 20 10 0 -10 -20
f1 (ppm)

***N*-(4-bromobenzyl)-*N*-(trifluoromethyl)methanethioamide (*S*11)**

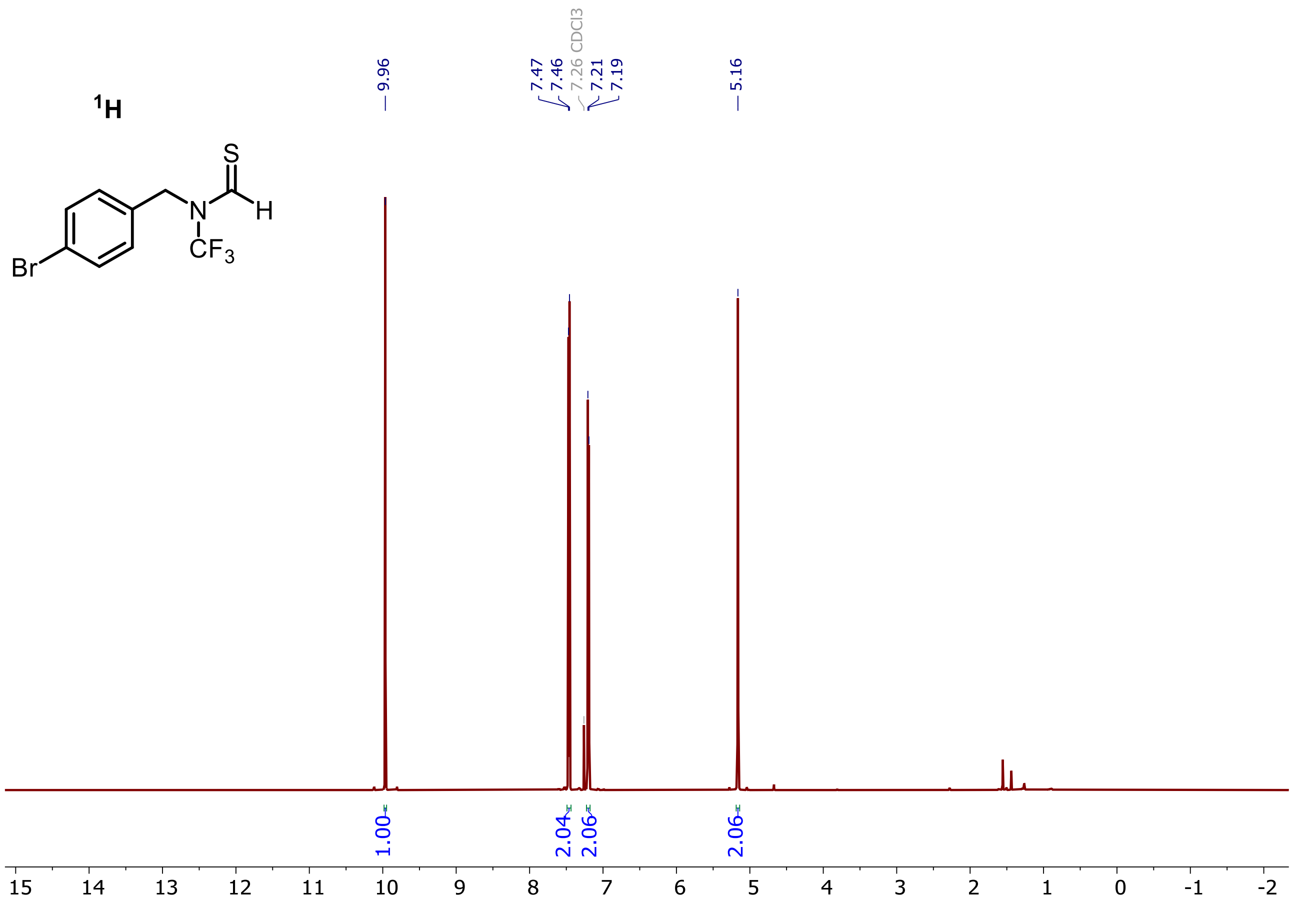

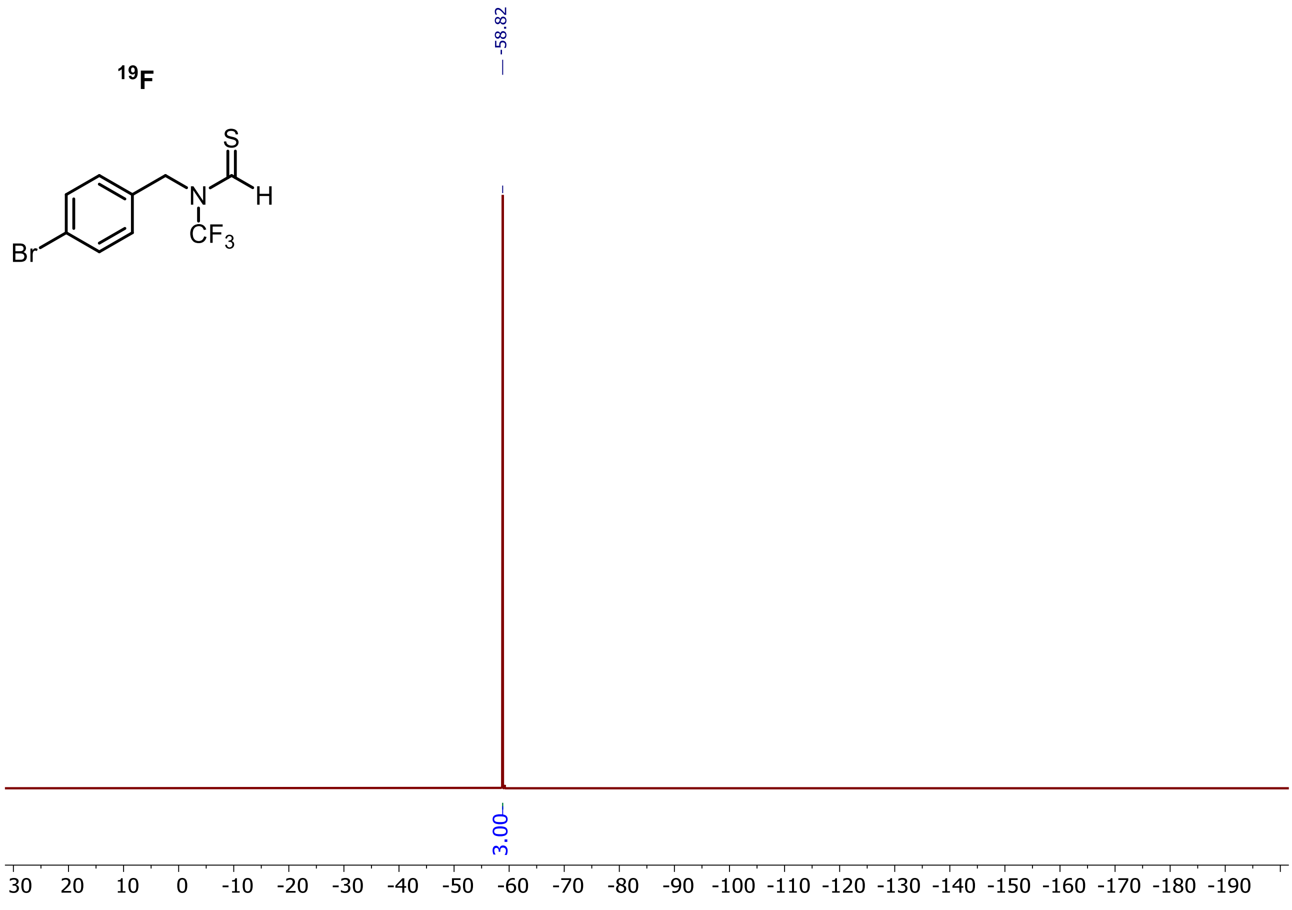

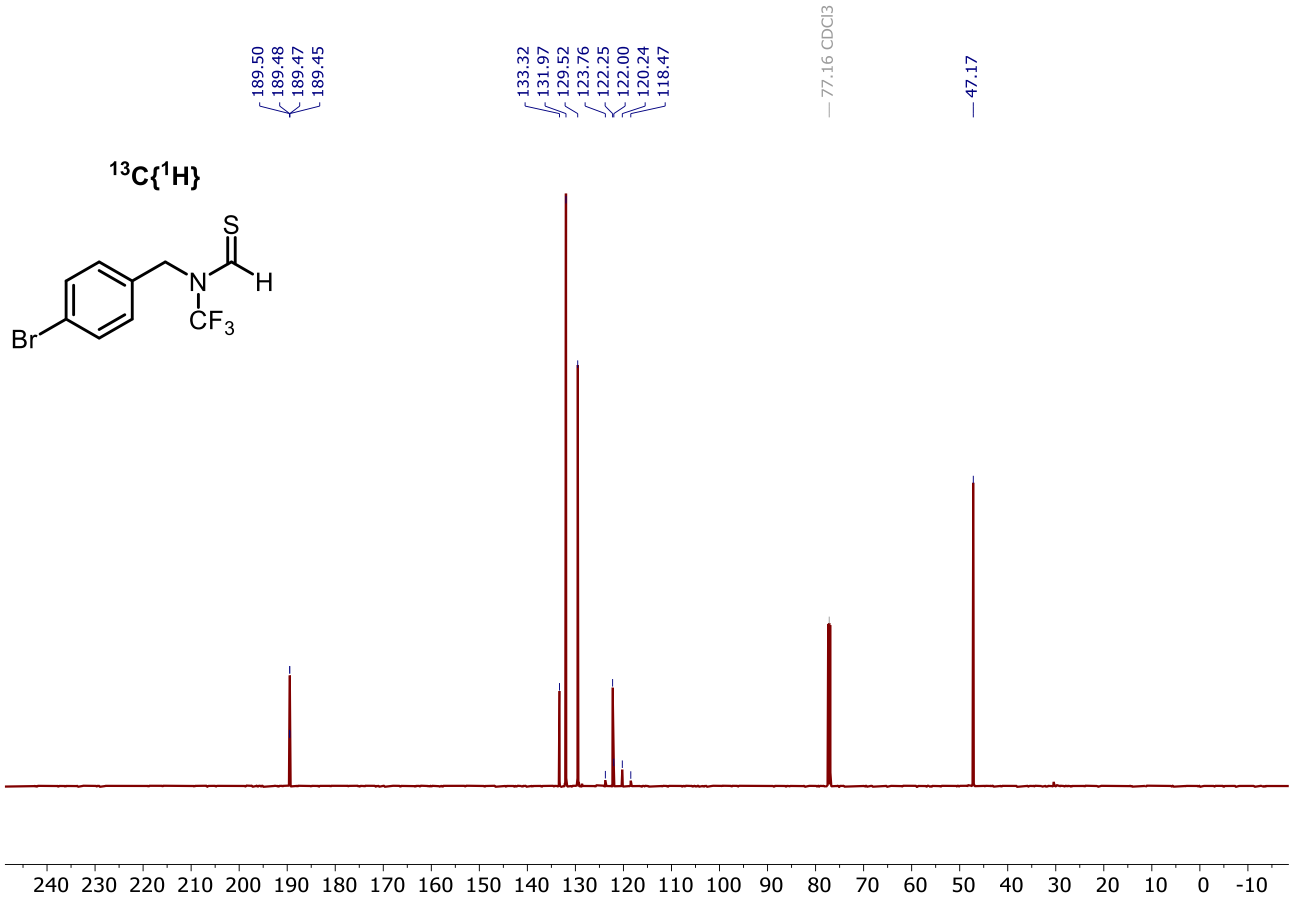

***tert*-butyl 4-(*N*-(trifluoromethyl)methanethioamido-*d*)piperidine-1-carboxylate (*S*12)**

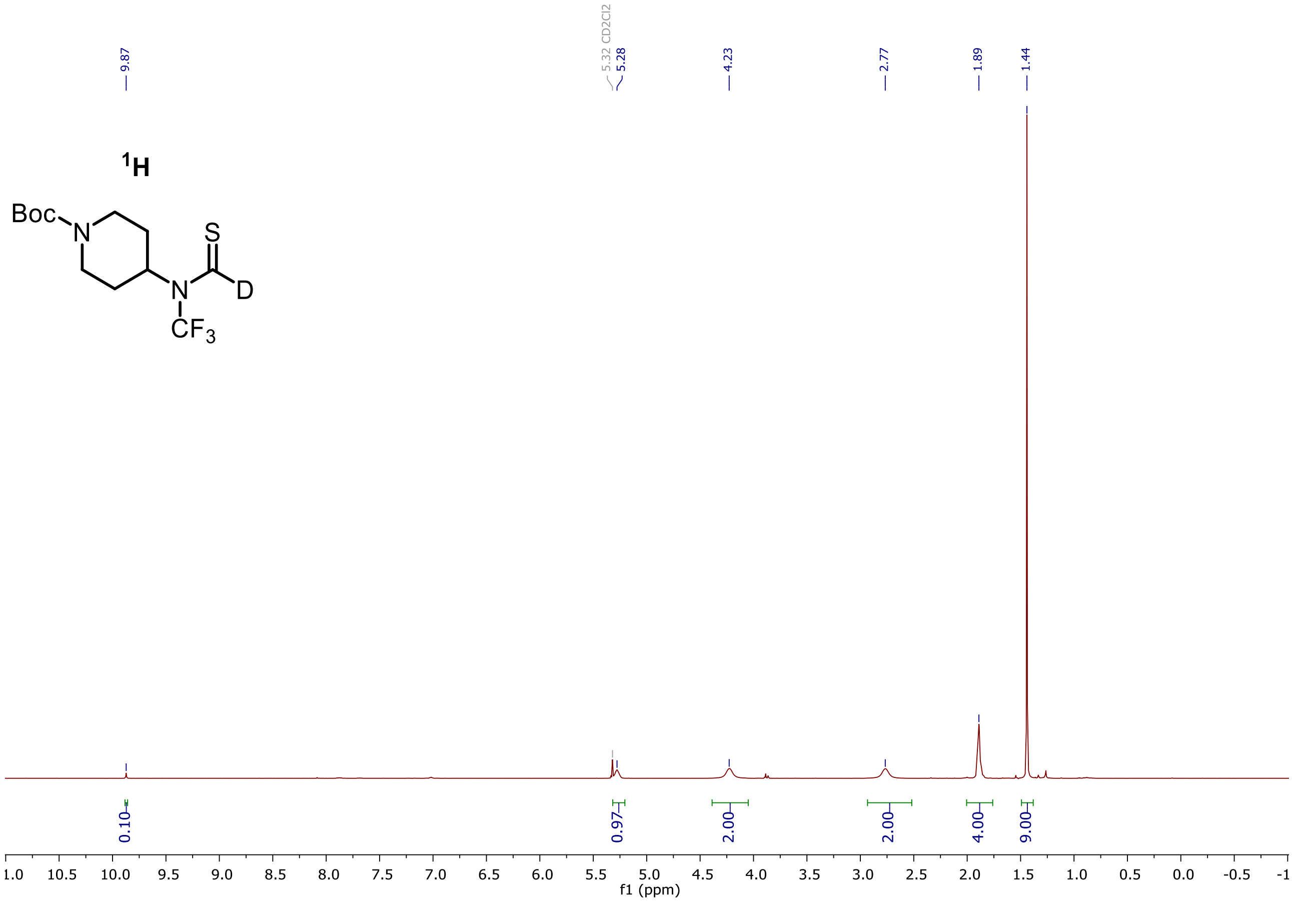

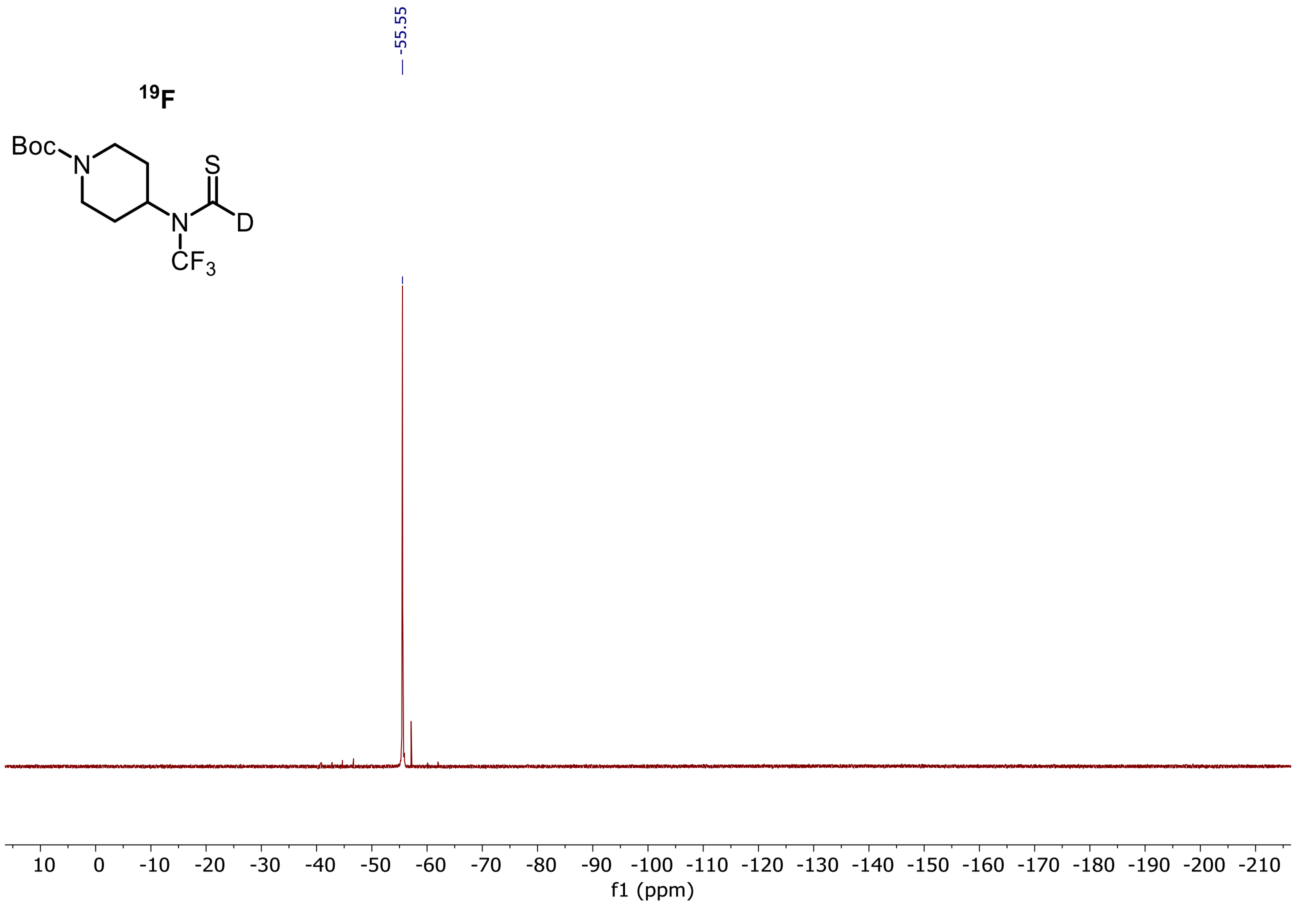
-55.55
19F
Boc
N
S
N
D
CF3
10 0 -10 -20 -30 -40 -50 -60 -70 -80 -90 -100 -110 -120 -130 -140 -150 -160 -170 -180 -190 -200 -210
f1 (ppm)

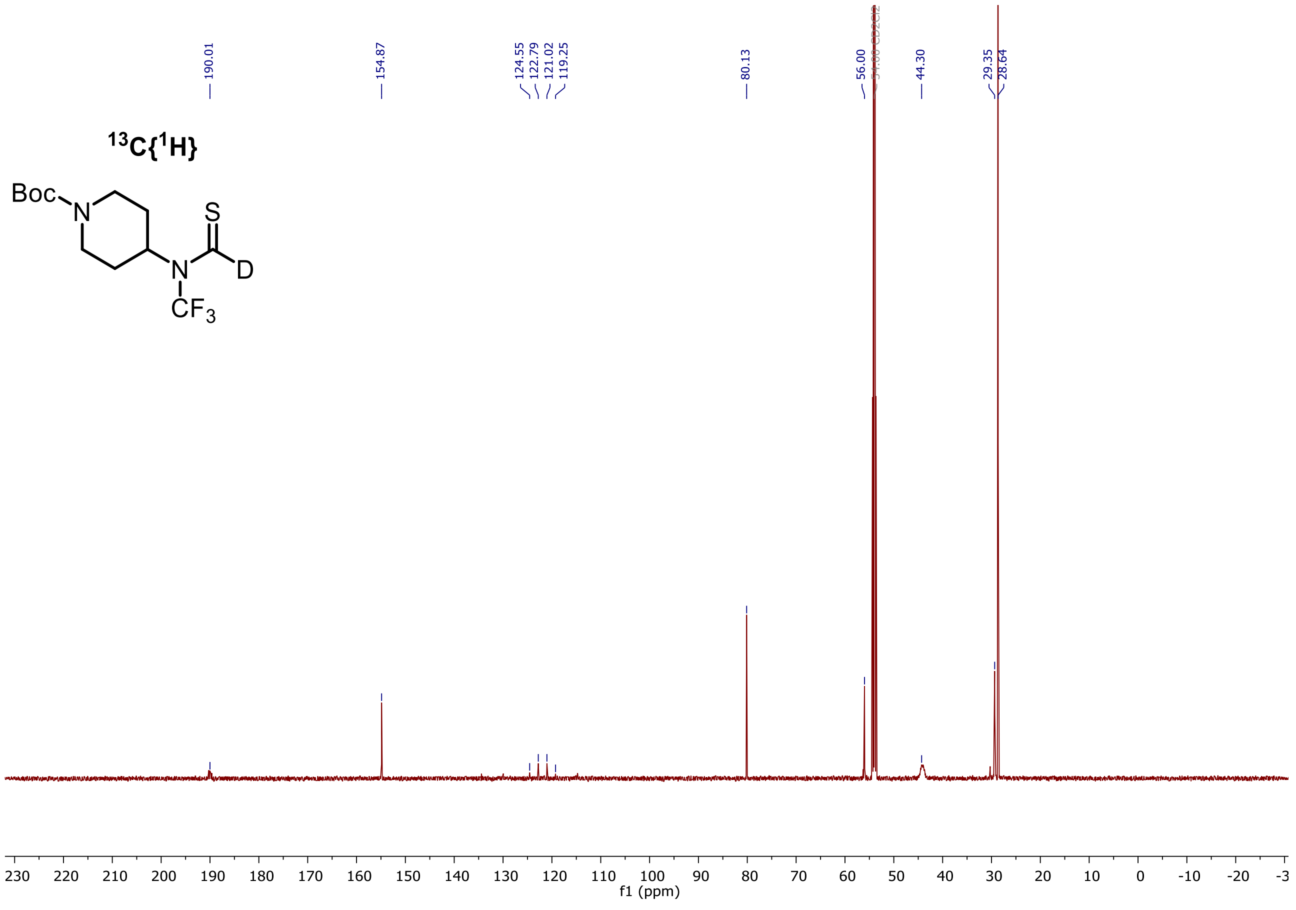
190.01
154.87
124.55
122.79
121.02
119.25
80.13
56.00
44.30
29.35
28.64
13C{1H}
Boc
N
S
N
D
CF3
230 220 210 200 190 180 170 160 150 140 130 120 110 100 90 80 70 60 50 40 30 20 10 0 -10 -20 -3
f1 (ppm)

N-(9-ethyl-9H-carbazol-3-yl)-N-(trifluoromethyl)methanethioamide (S13)

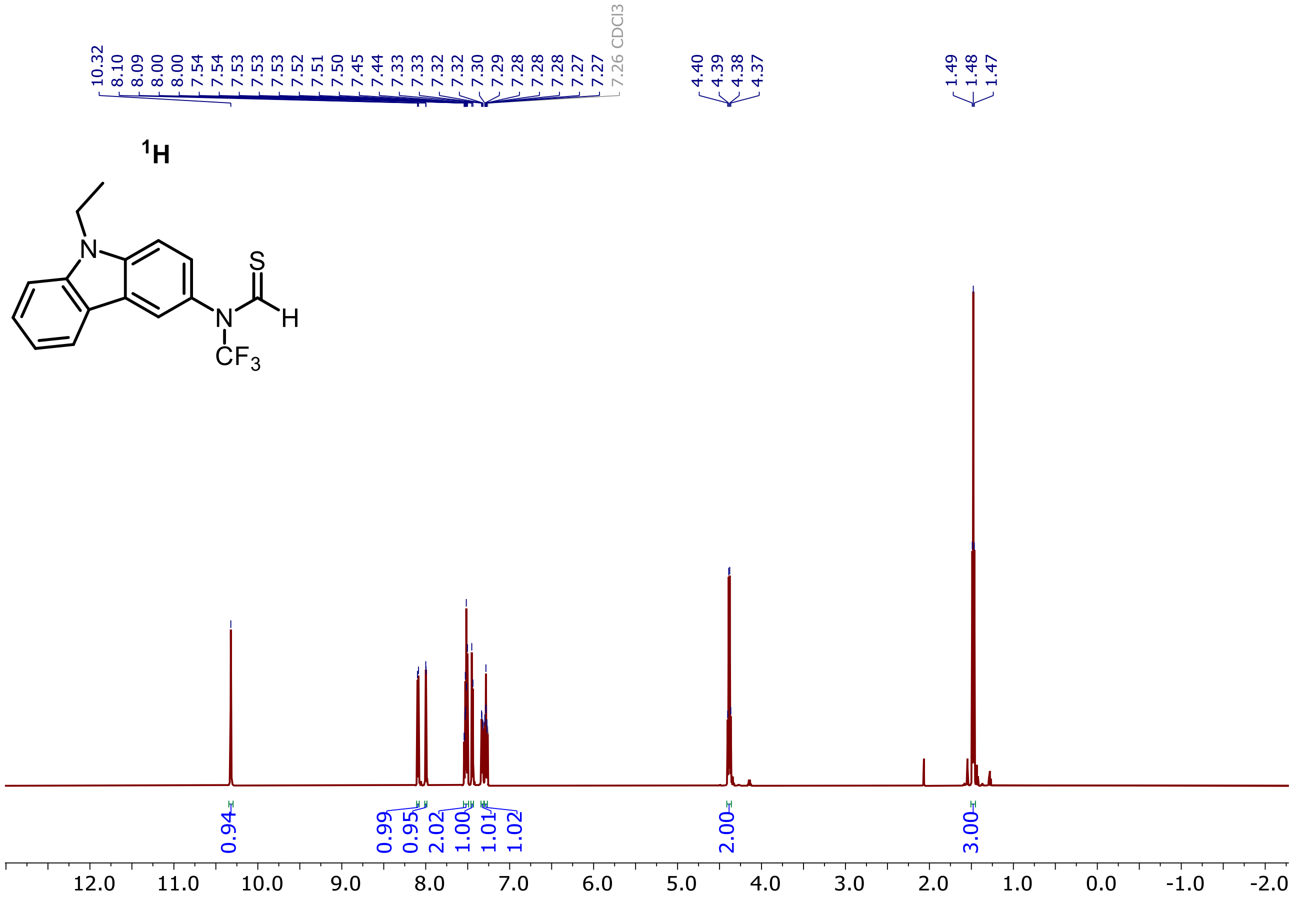

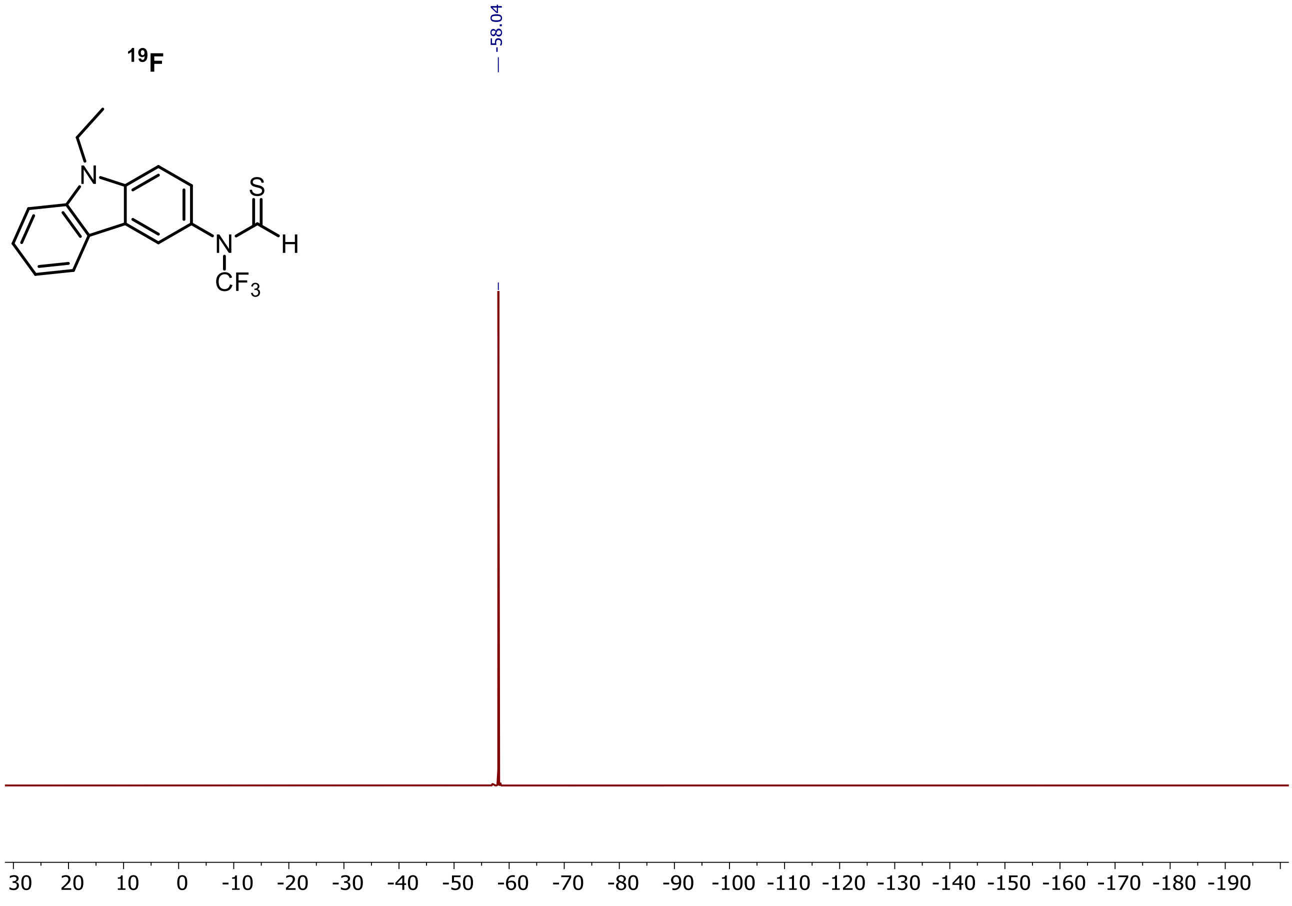

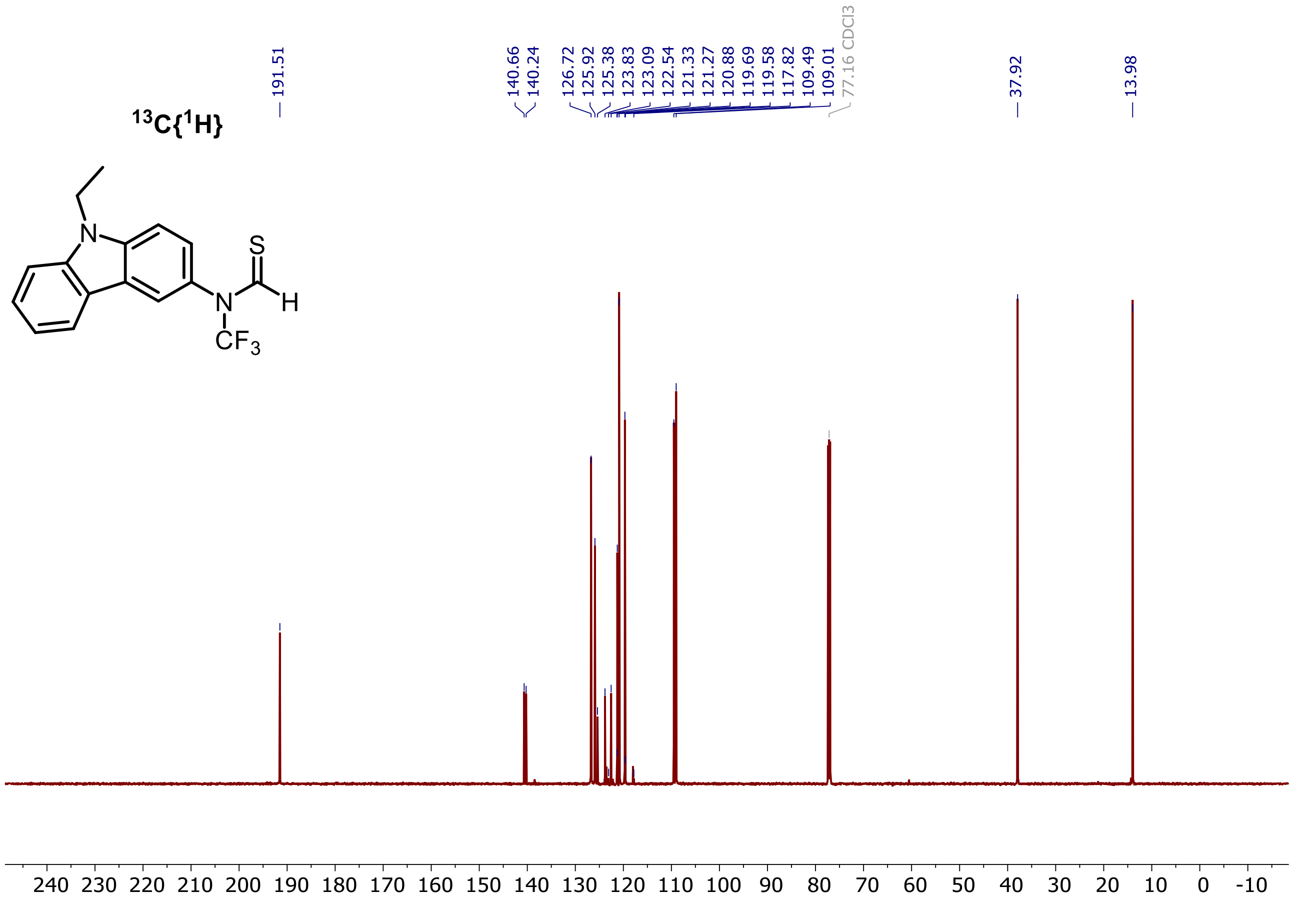

methyl 4-(*N*-(trifluoromethyl)methanethioamido)thiophene-2-carboxylate (*S*14)

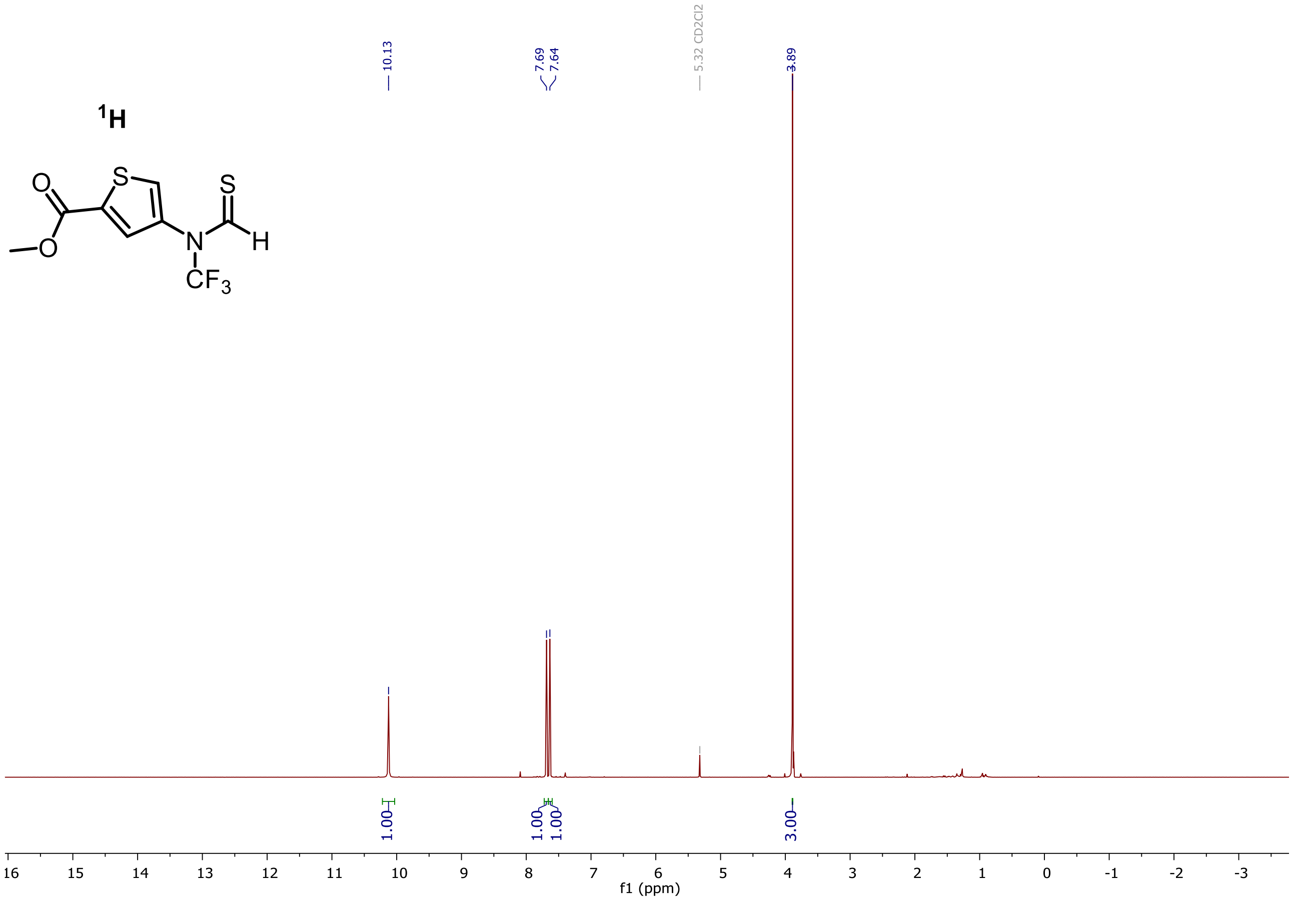

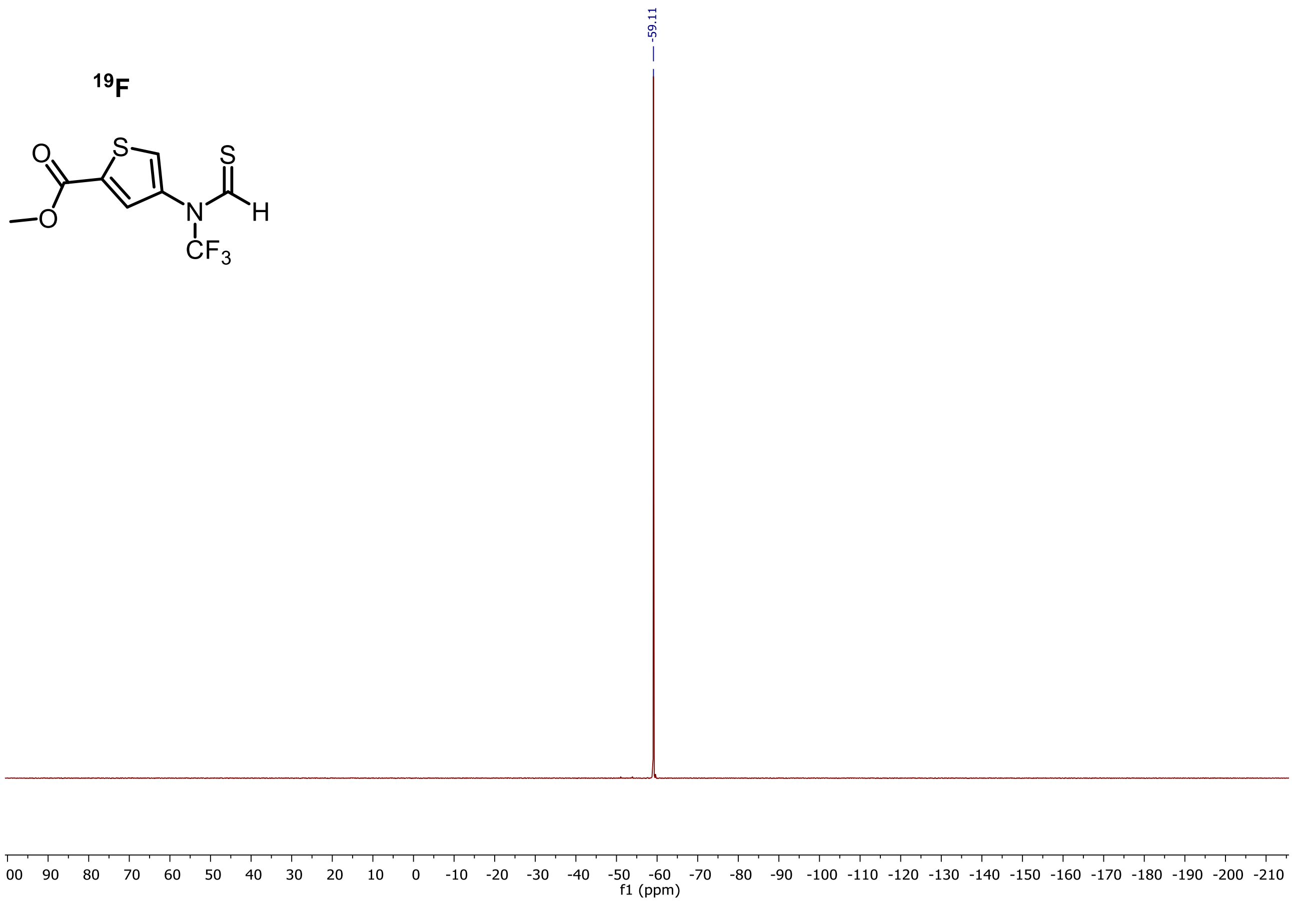
-59.11
19F
S
O
O
N
S
H
CF3
f1 (ppm)

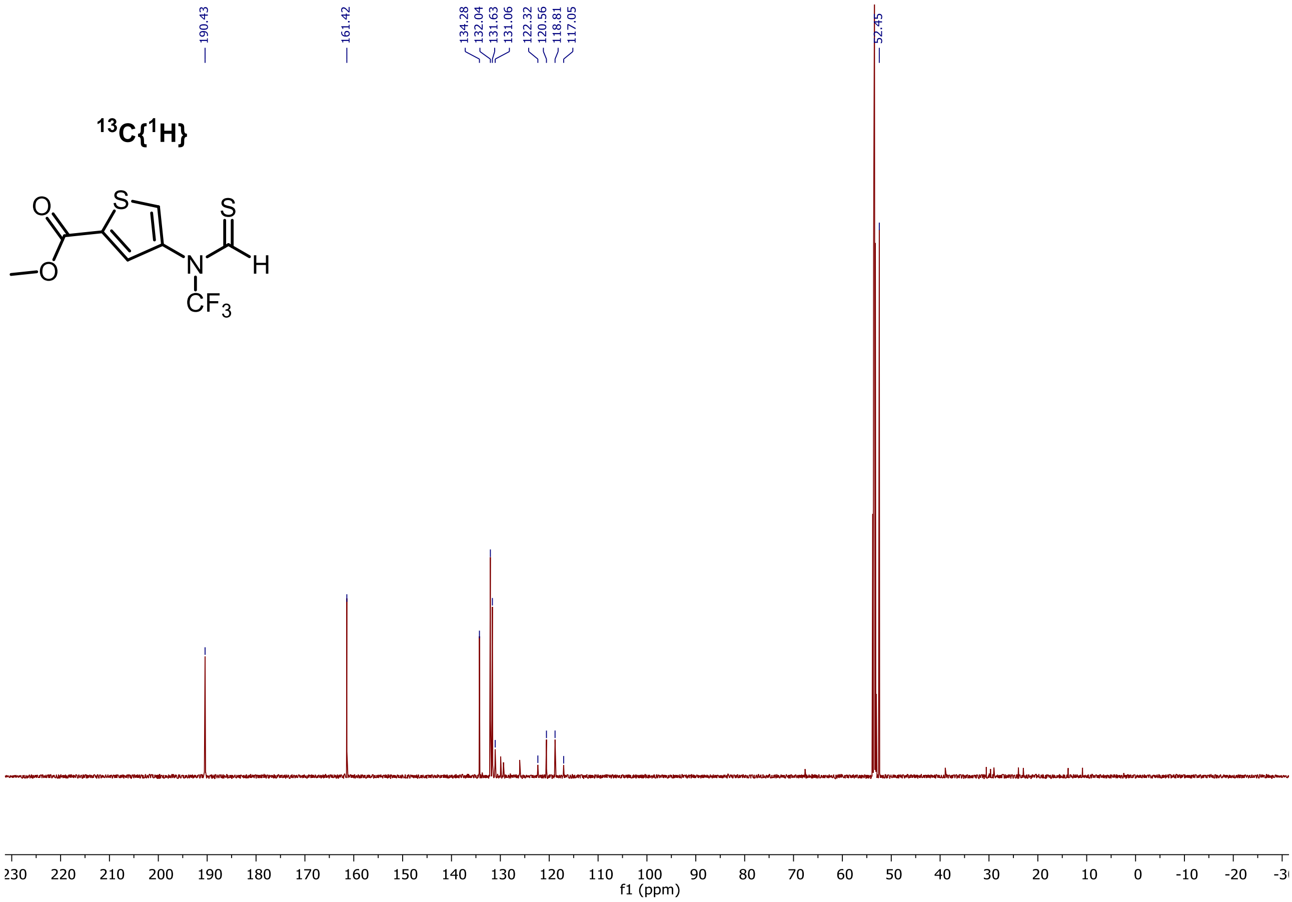
190.43
161.42
134.28
132.04
131.63
131.06
122.32
120.56
118.81
117.05
52.45
13C{1H}
S
O
O
N
S
H
CF3
f1 (ppm)

_N_-(_trans_-2-phenylcyclopropyl)-_N_-(trifluoromethyl)methanethioamide (S15)

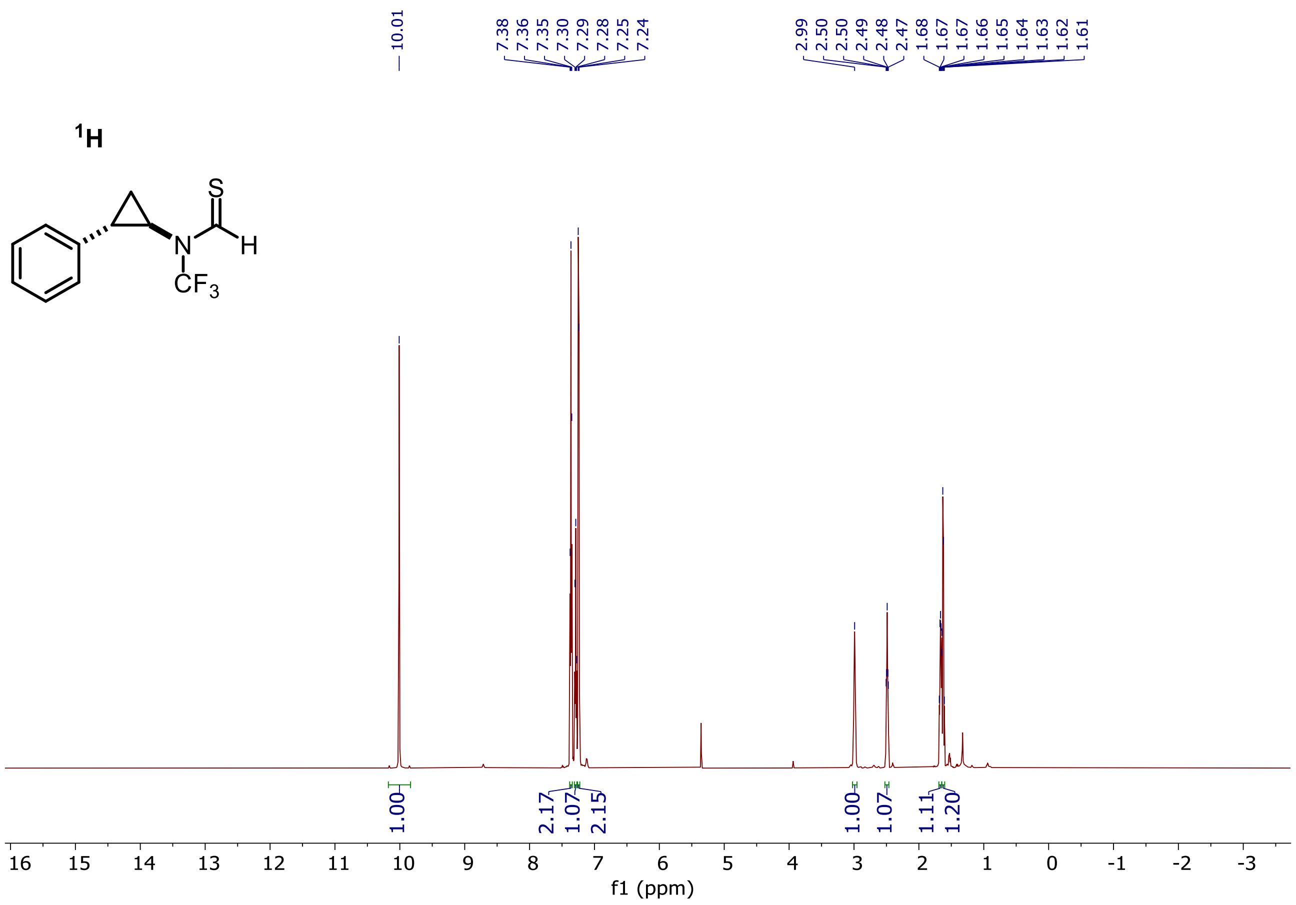

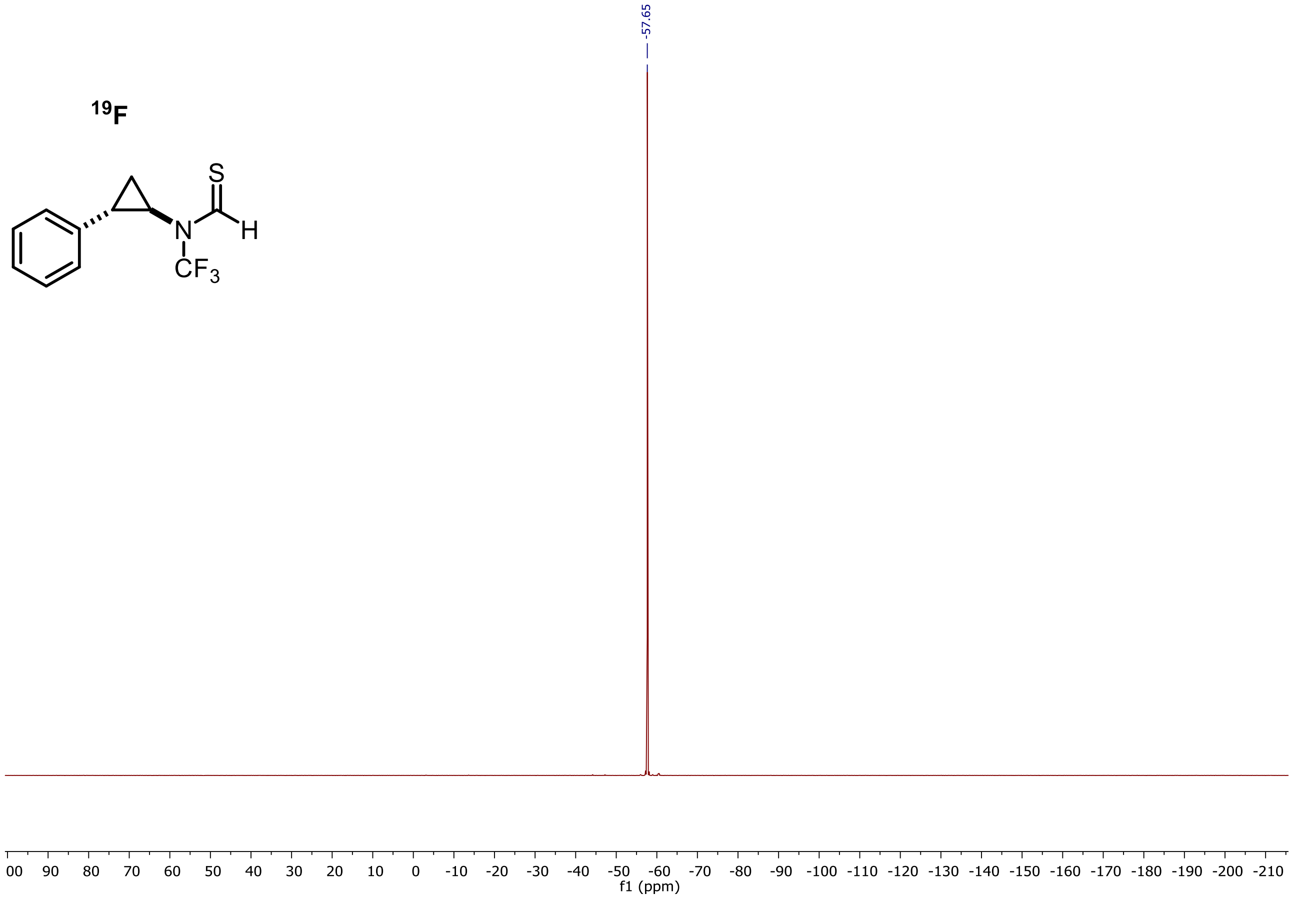

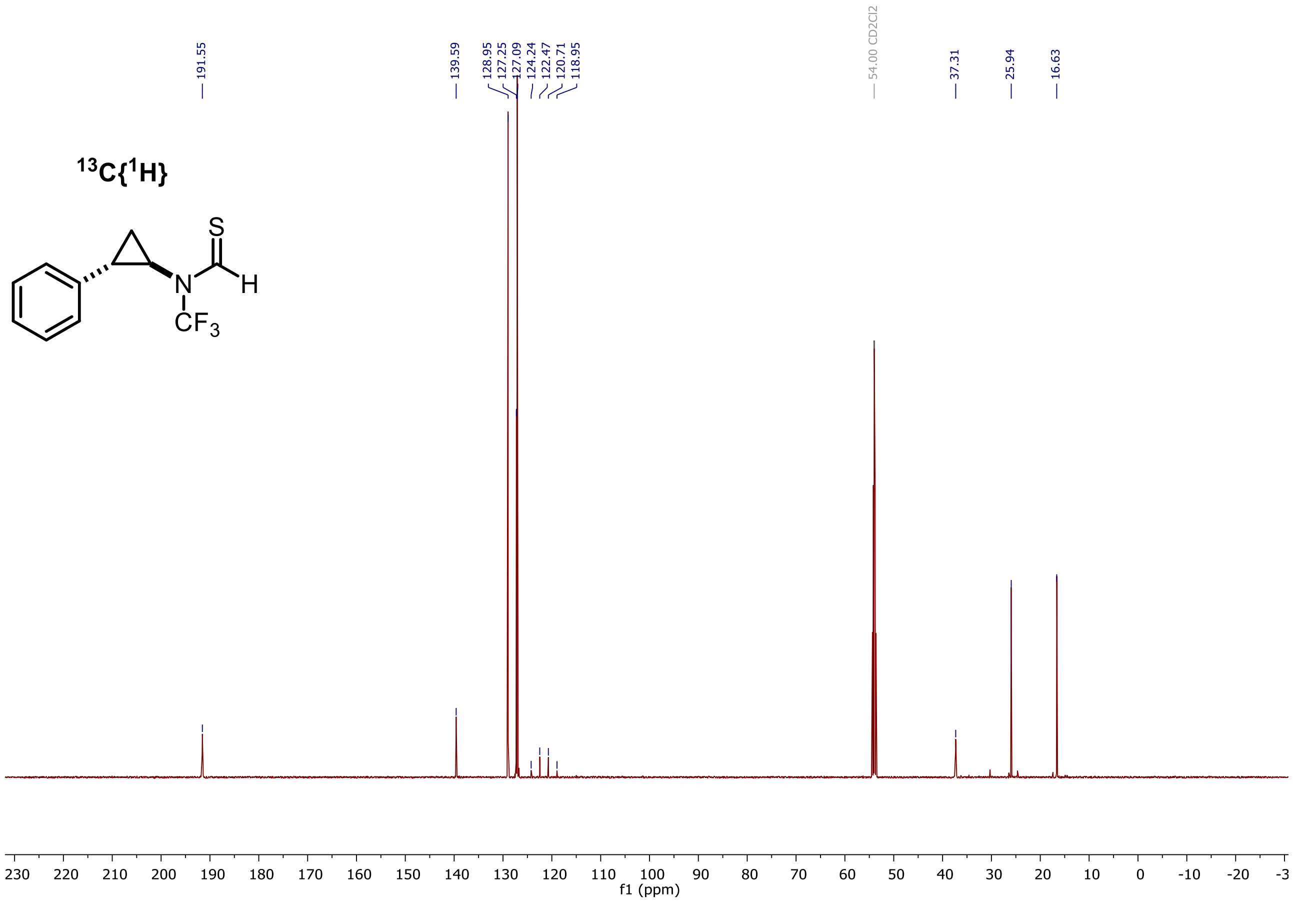

N-(1-(2,6-dimethylphenoxy)propan-2-yl)-*N*-(trifluoromethyl)methanethioamide (*S*16)

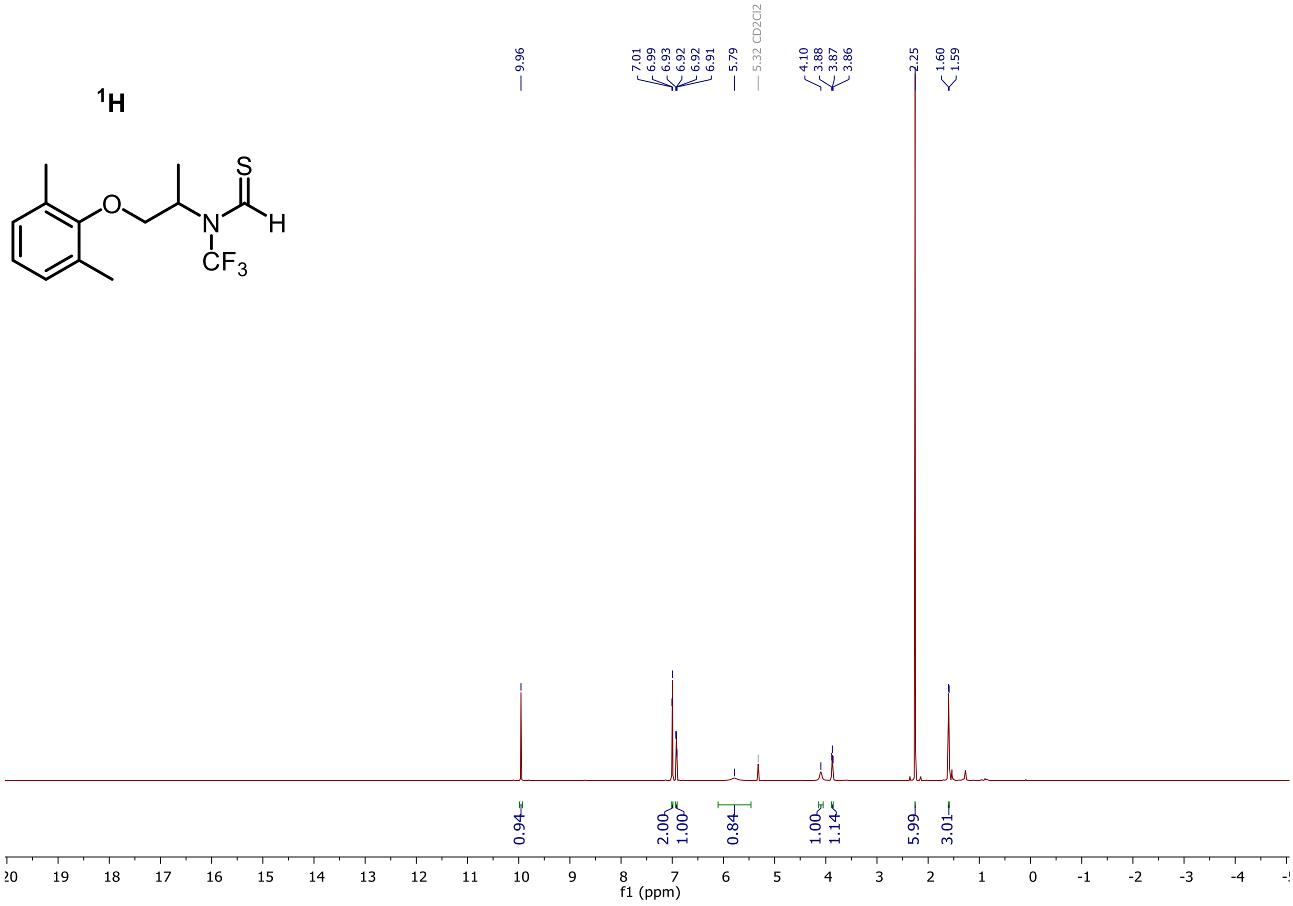

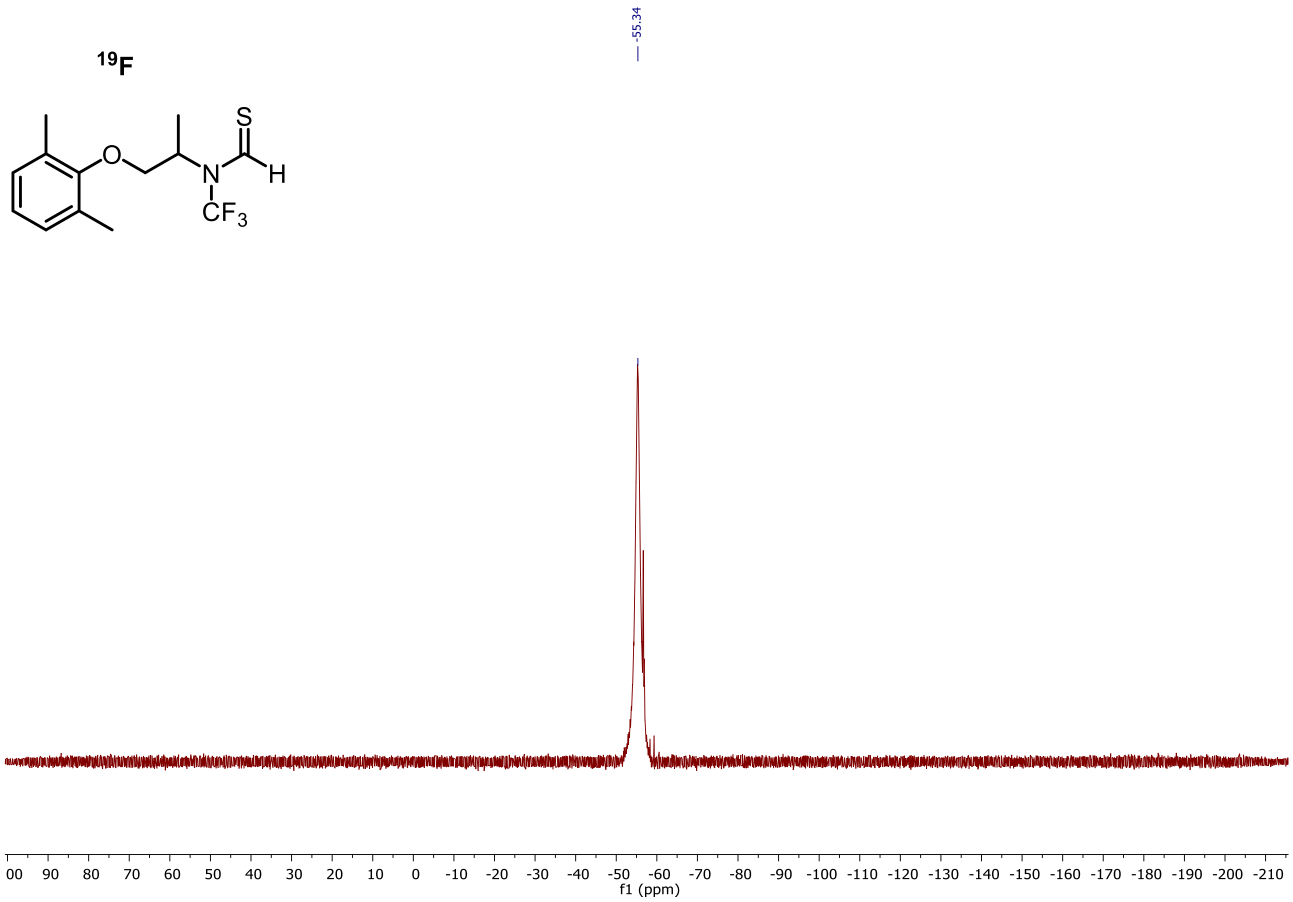
19F
S
O
N
H
CF3
-55.34
00 90 80 70 60 50 40 30 20 10 0 -10 -20 -30 -40 -50 -60 -70 -80 -90 -100 -110 -120 -130 -140 -150 -160 -170 -180 -190 -200 -210
f1 (ppm)

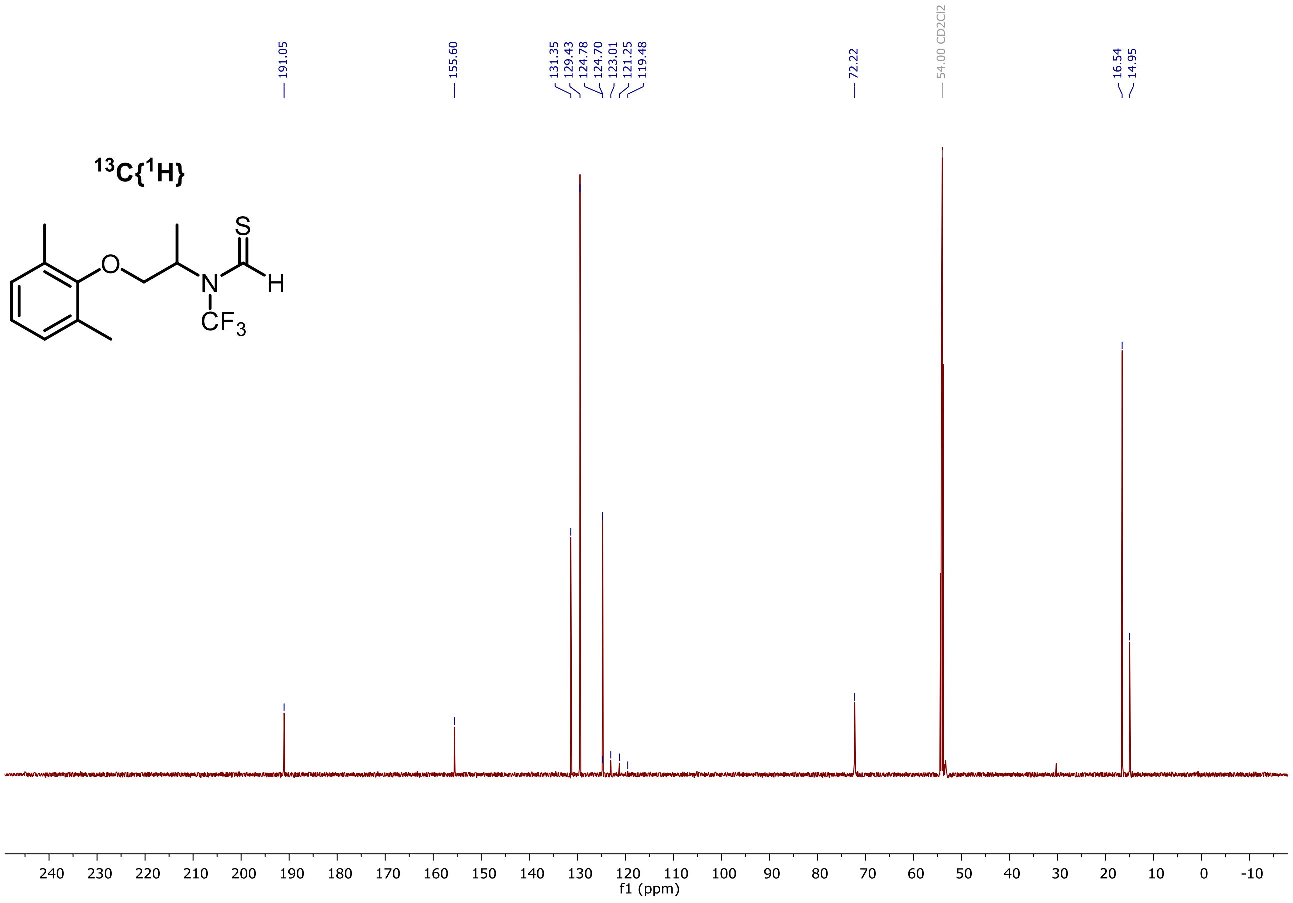
13C{1H}
S
O
N
H
CF3
191.05
155.60
131.35
129.43
124.78
124.70
123.01
121.25
119.48
72.22
54.00 CD2Cl2
16.54
14.95
240 230 220 210 200 190 180 170 160 150 140 130 120 110 100 90 80 70 60 50 40 30 20 10 0 -10
f1 (ppm)

(*R*)-*N*-(1-(3-(2-cyanobenzyl)-1-methyl-2-oxo-6-thioxo-1,2,3,6-tetrahydropyrimidin-4-yl)piperidin-3-yl)-*N*-(trifluoromethyl)methanethioamide (S17)

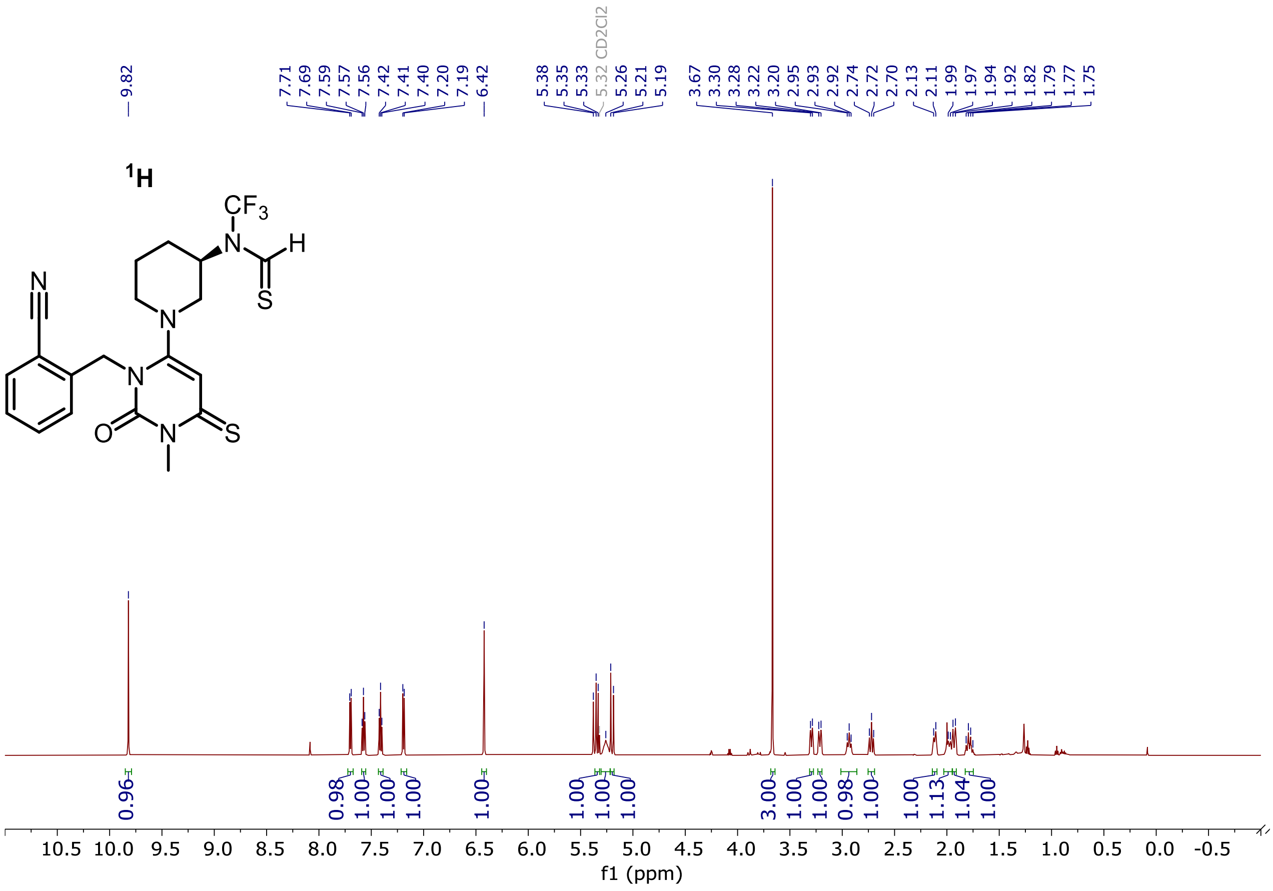

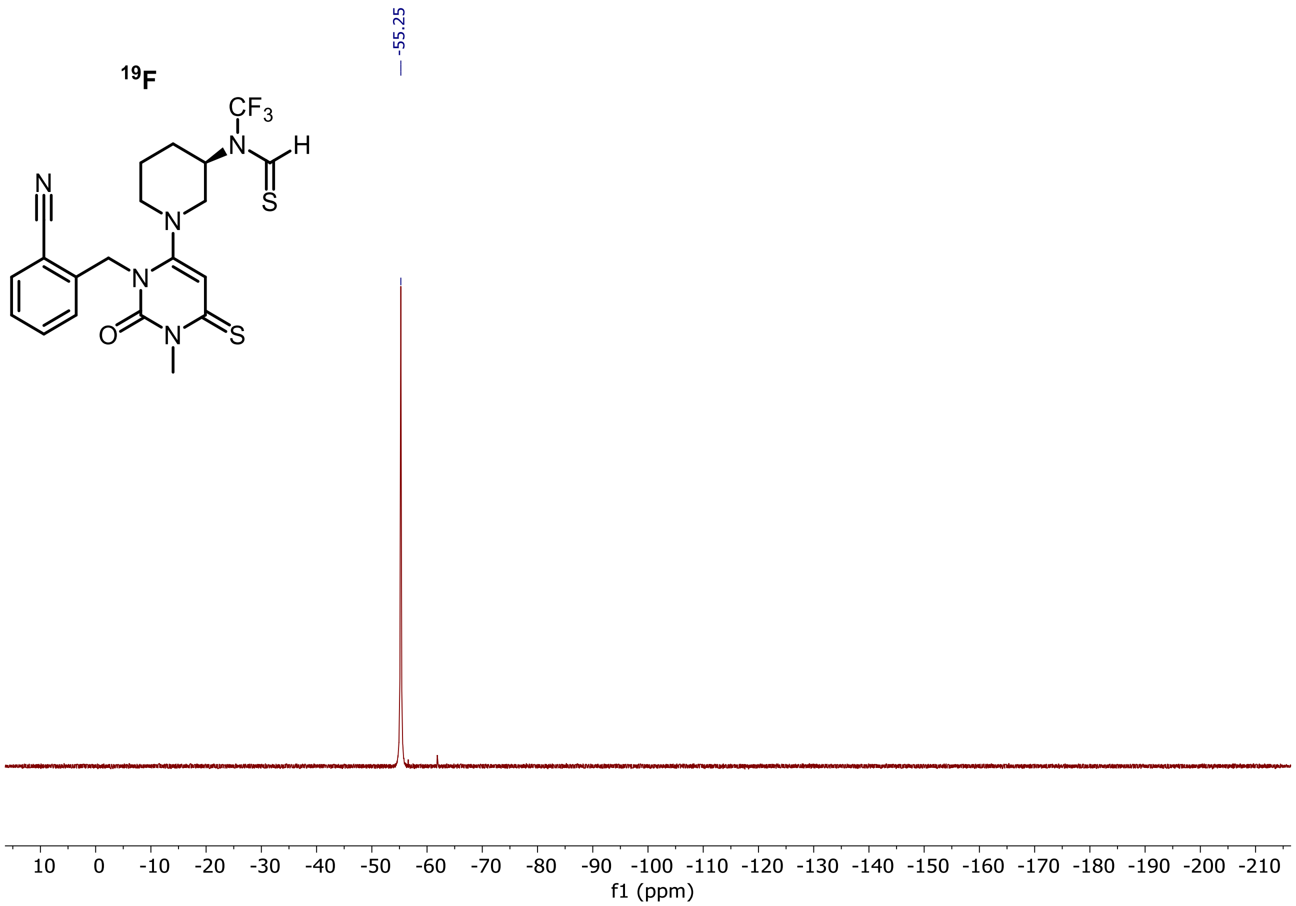

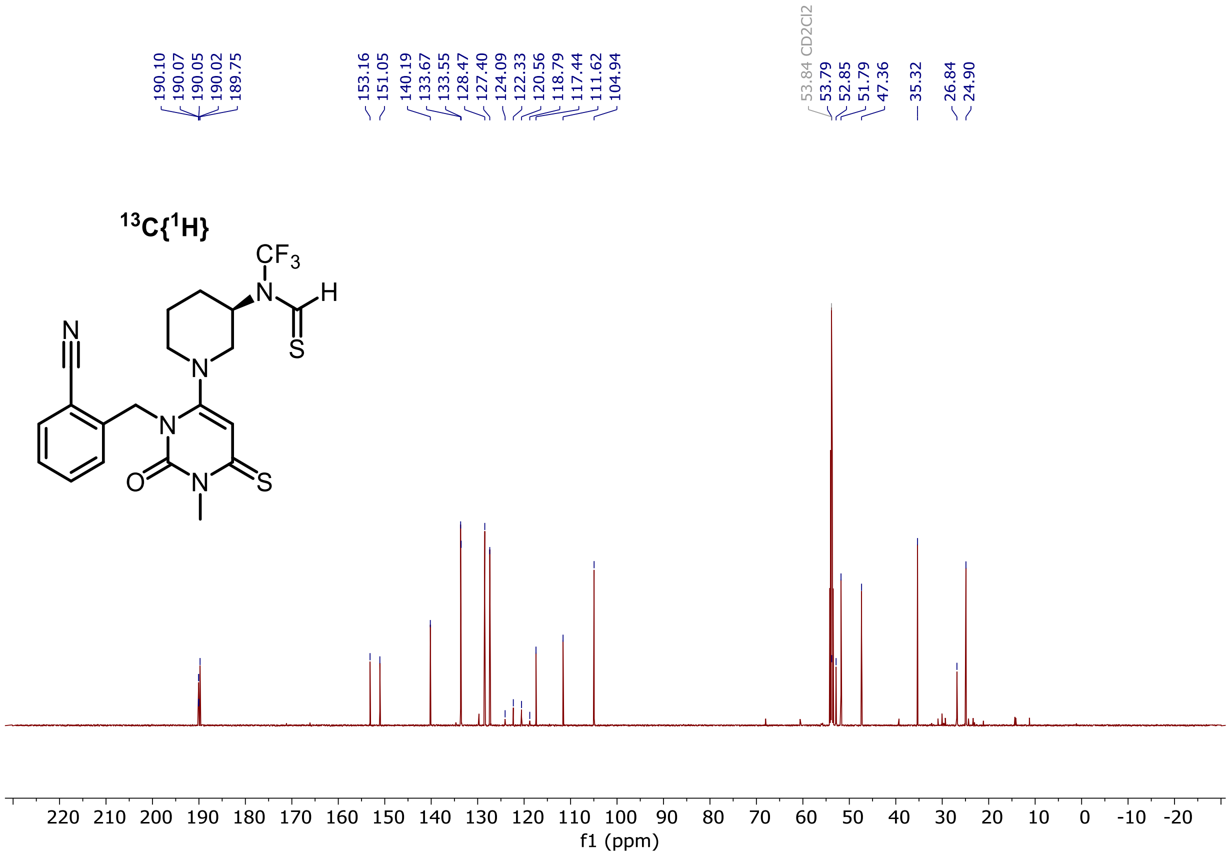
190.10
190.07
190.05
190.02
189.75
153.16
151.05
140.19
133.67
133.55
128.47
127.40
124.09
122.33
120.56
118.79
117.44
111.62
104.94
53.84 CD2Cl2
53.79
52.85
51.79
47.36
35.32
26.84
24.90
13C{1H}
CF3
N
H
S
N
N
N
N
O
S
220 210 200 190 180 170 160 150 140 130 120 110 100 90 80 70 60 50 40 30 20 10 0 -10 -20
f1 (ppm)

12.2.2 Isothiocyanates

Methyl 4-isothiocyanatothiophene-2-carboxylate (S18)

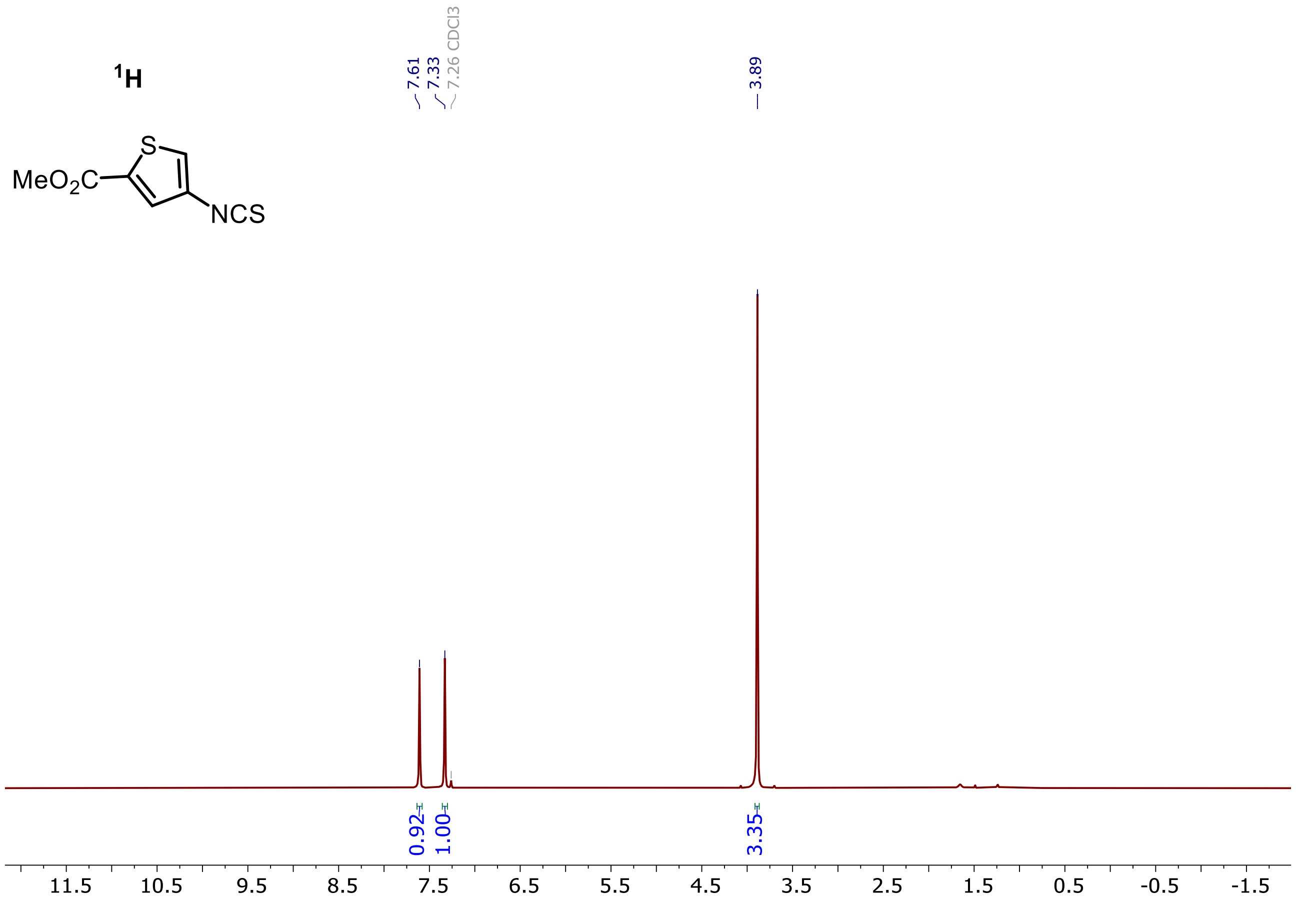

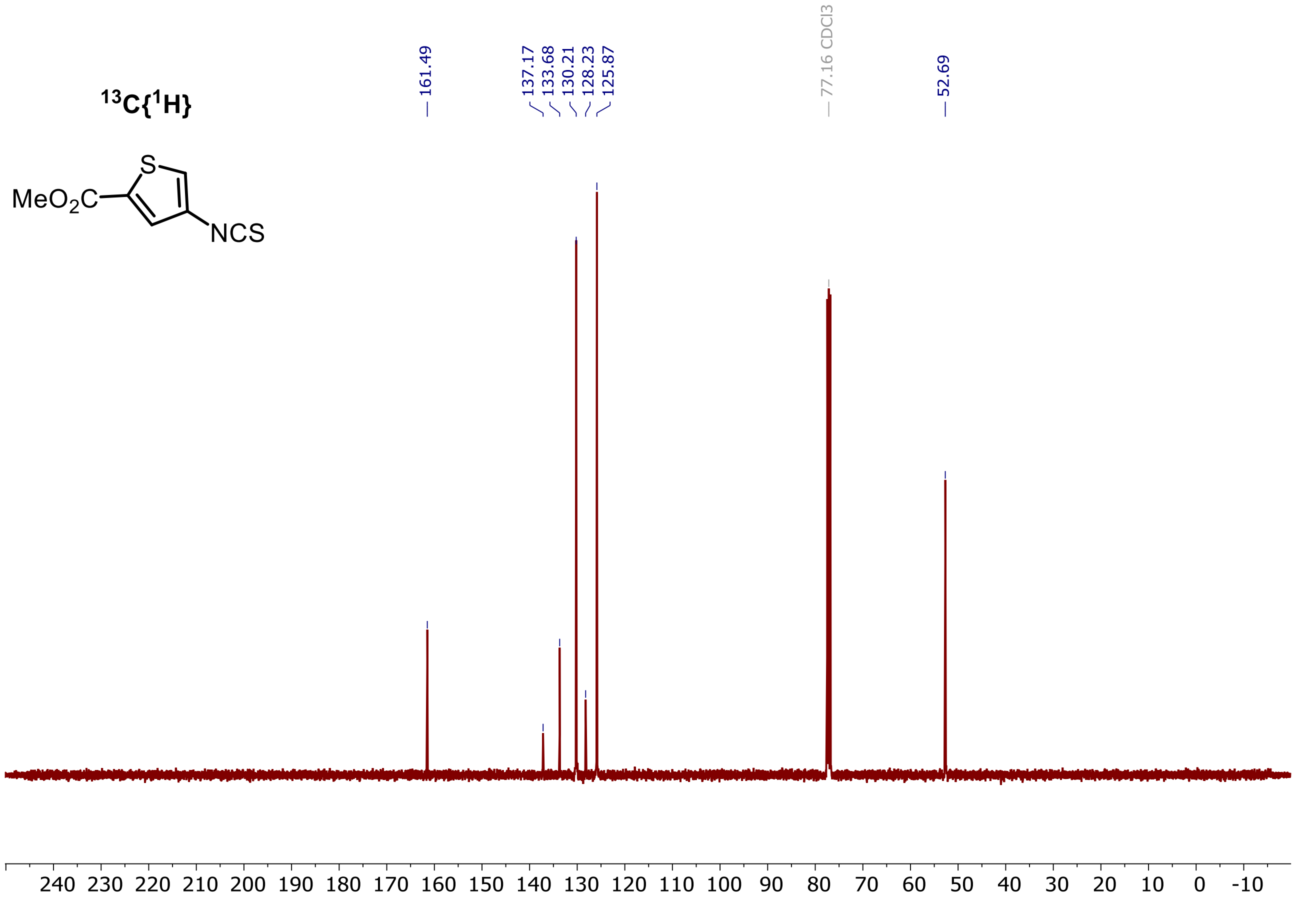

(*trans*-2-isothiocyanatocyclopropyl)benzene (S19)

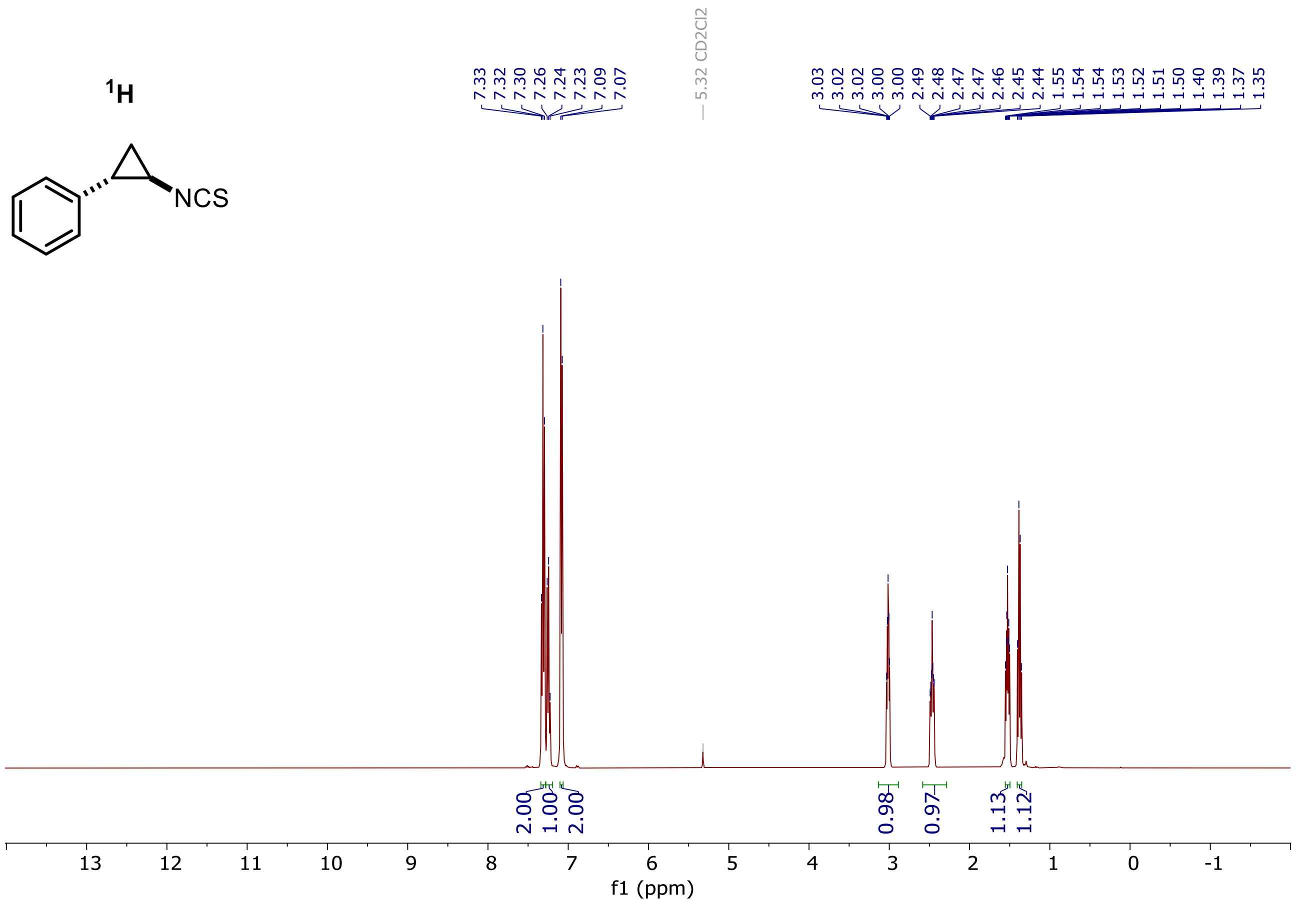

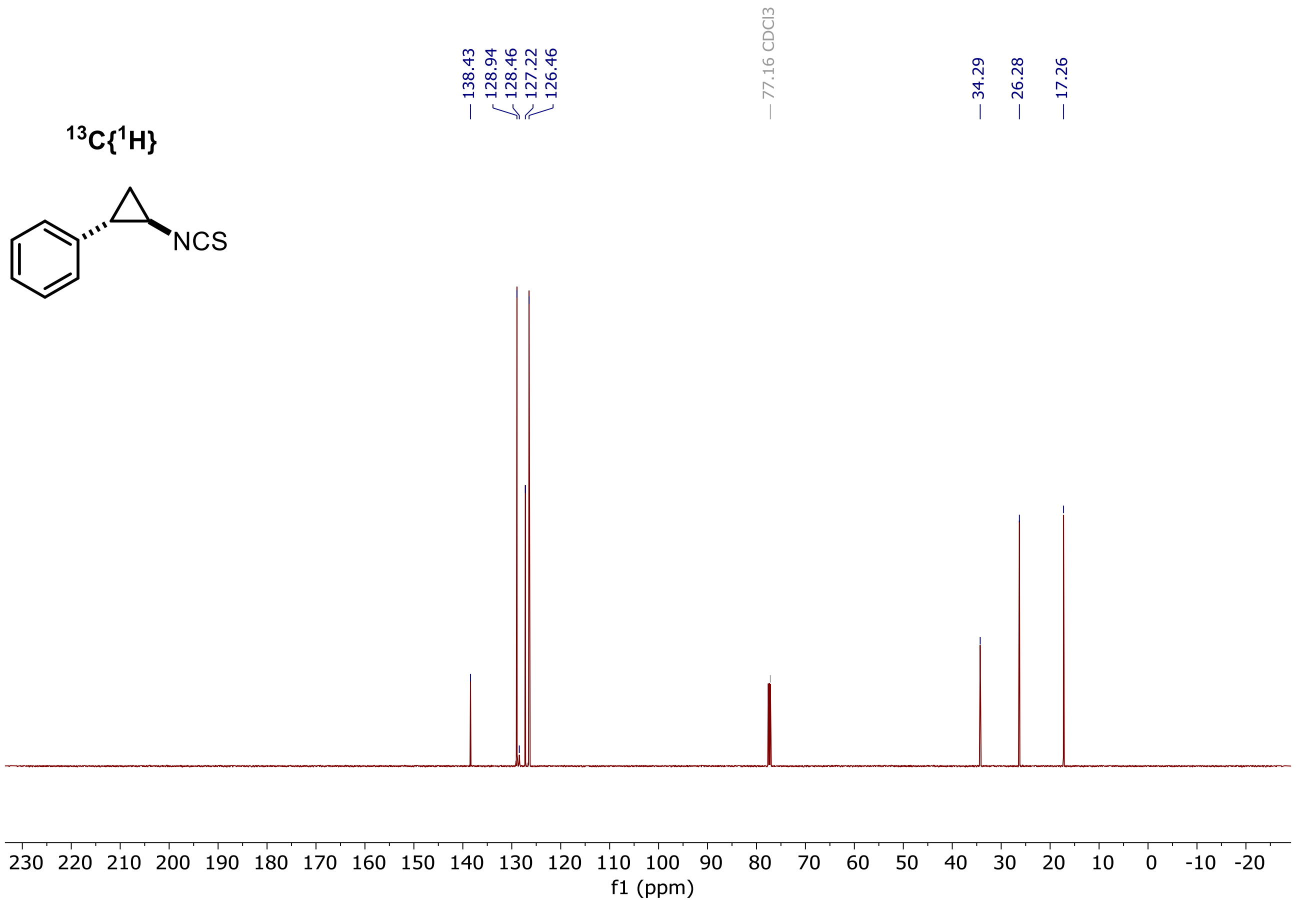

(*R*)-2-((6-(3-isothiocyanatopiperidin-1-yl)-3-methyl-2,4-dioxo-3,4-dihydropyrimidin-1(2*H*)-yl)methyl)benzonitrile (*S*20)

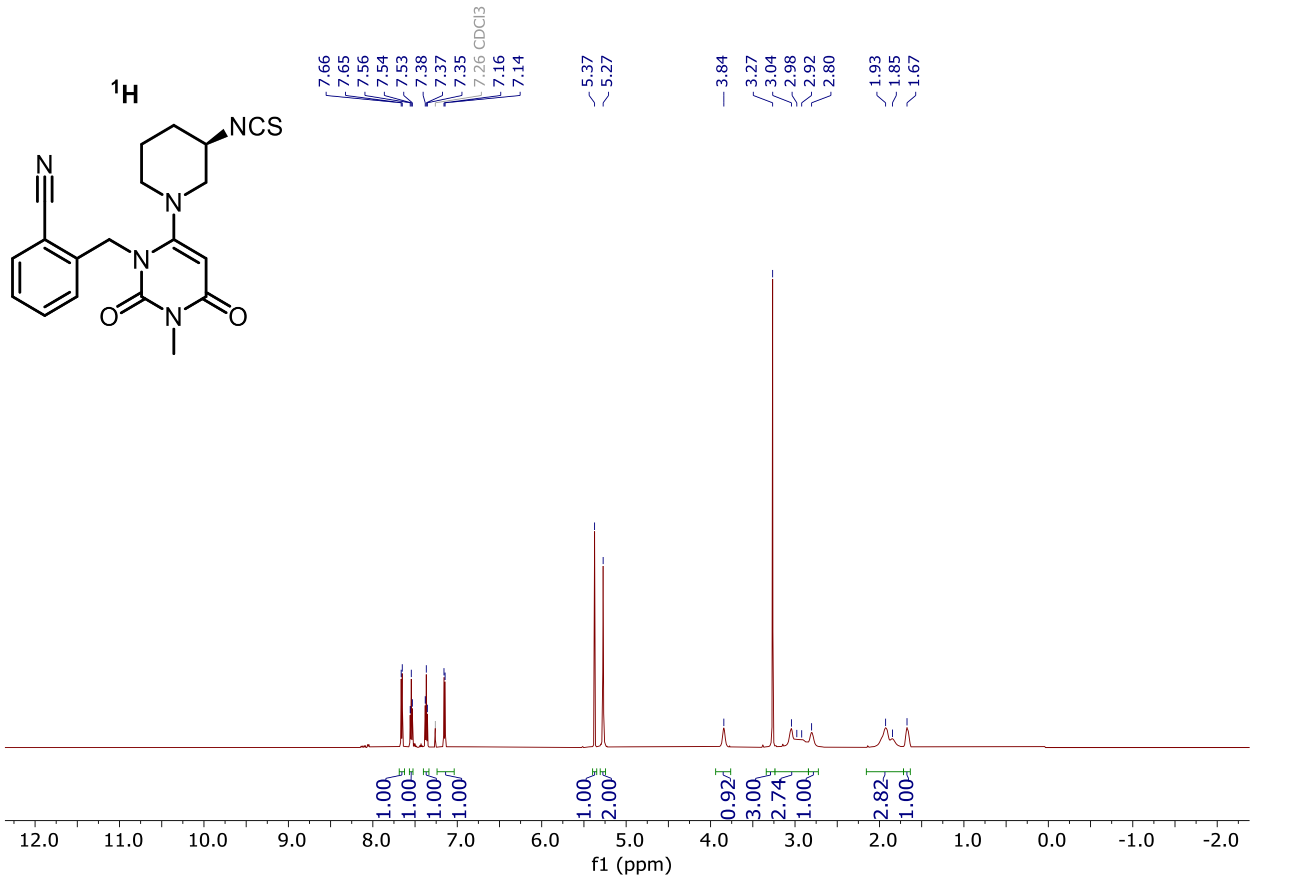

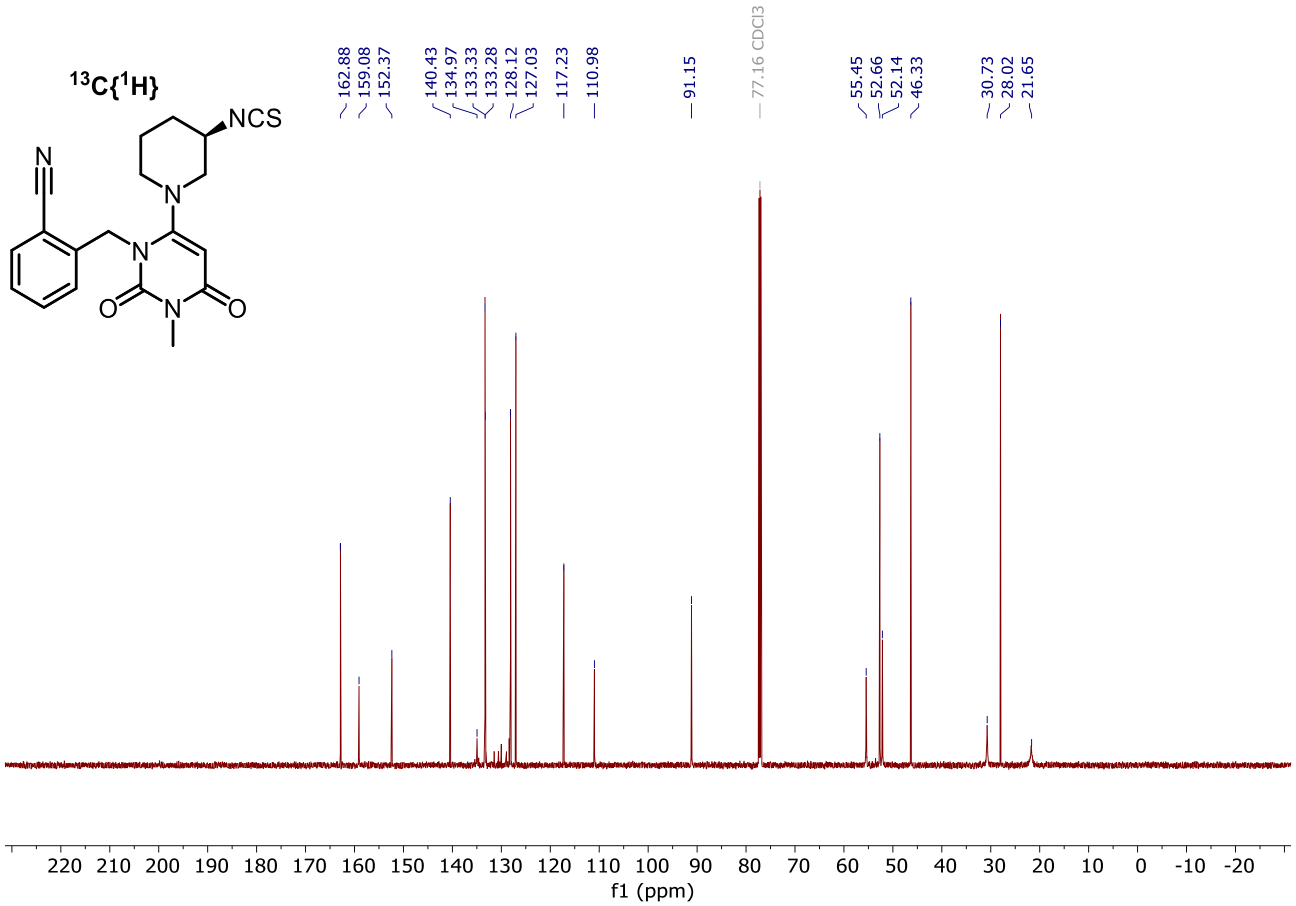

12.2.3 *N*-CF_3 carbamoyl fluoride

(4-cyclohexylphenyl)(trifluoromethyl)carbamoyl fluoride (S21)

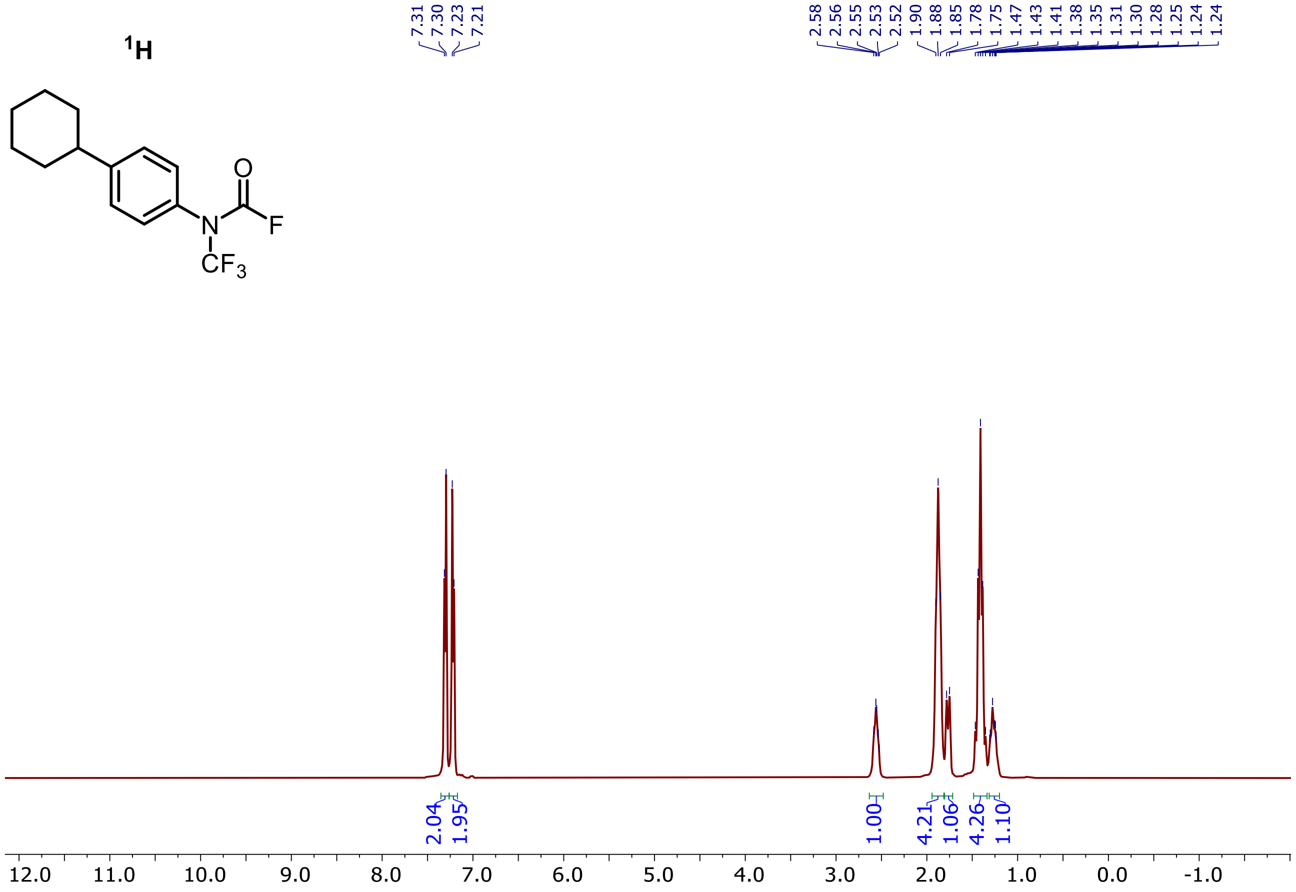

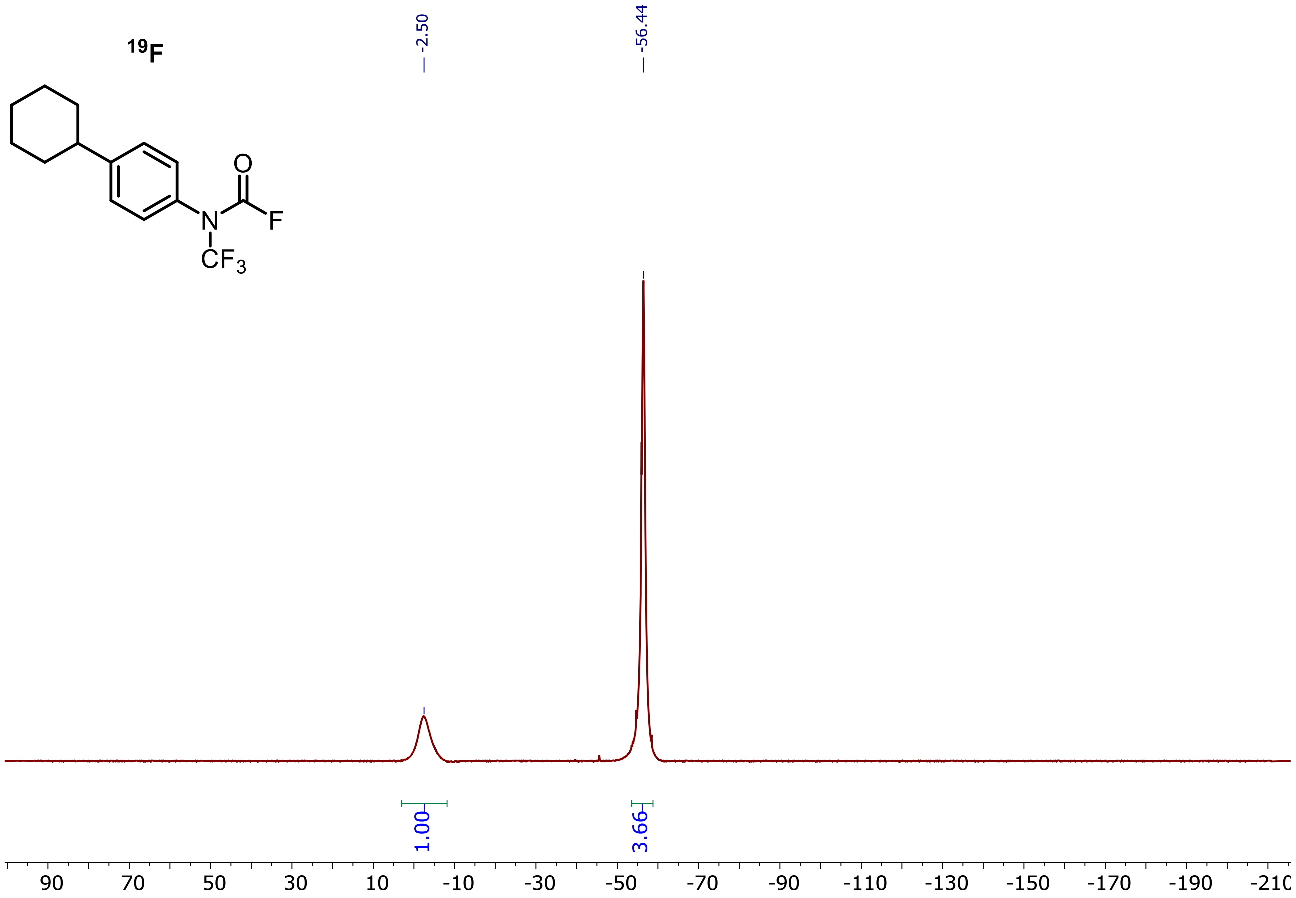

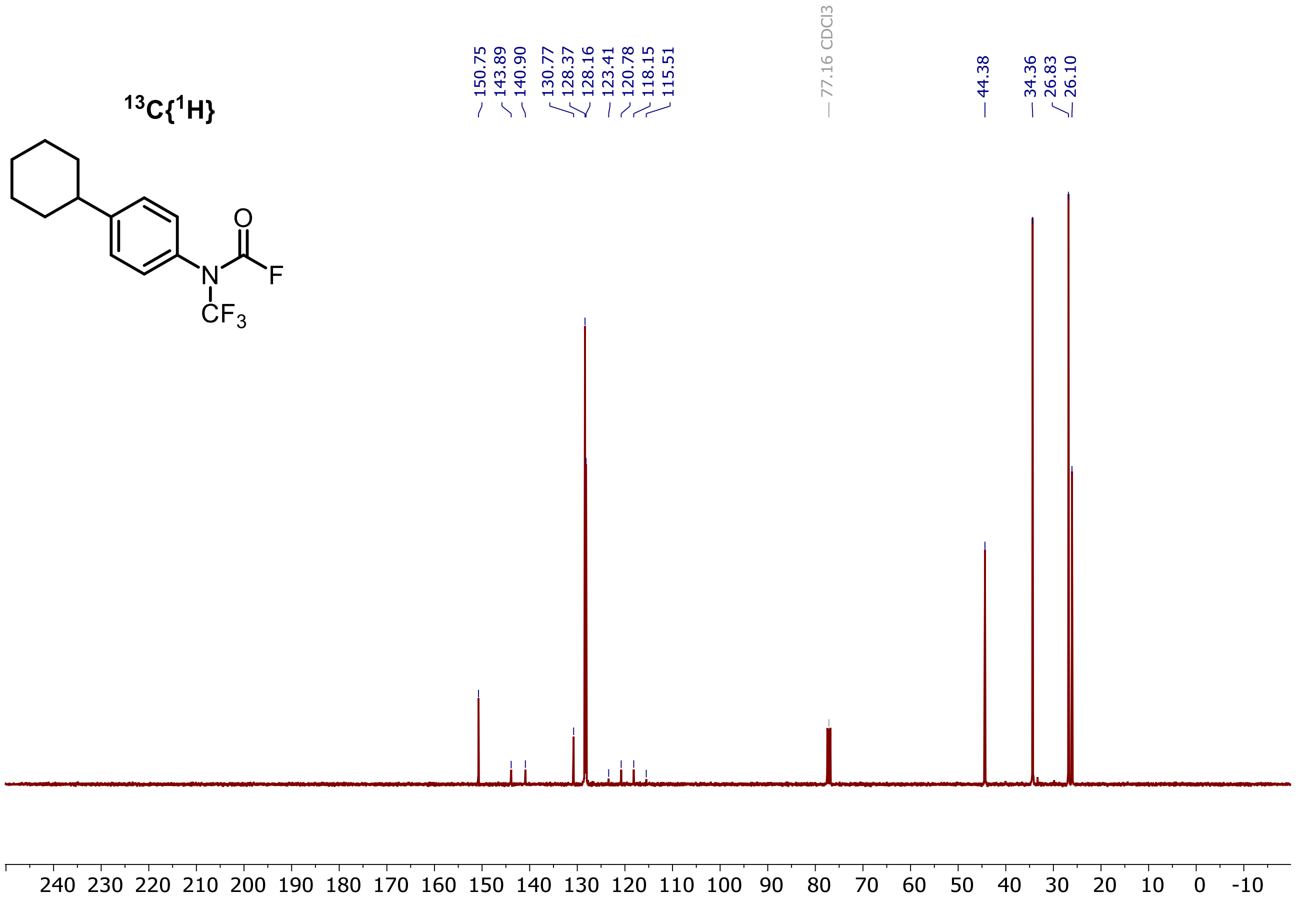

benzyl *N*-(fluorocarbonyl)-*N*-(trifluoromethyl)leucinate (*S*22)

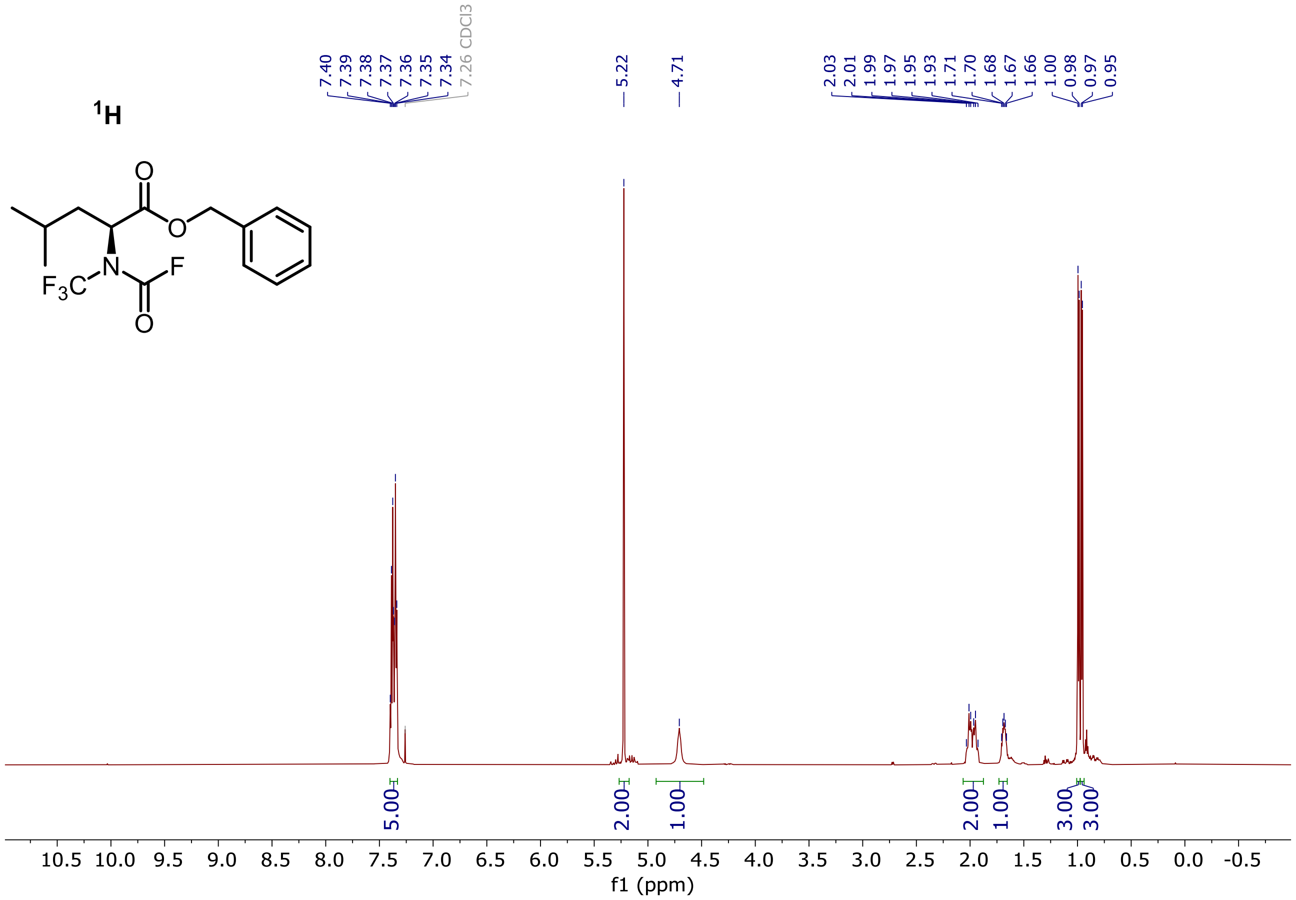

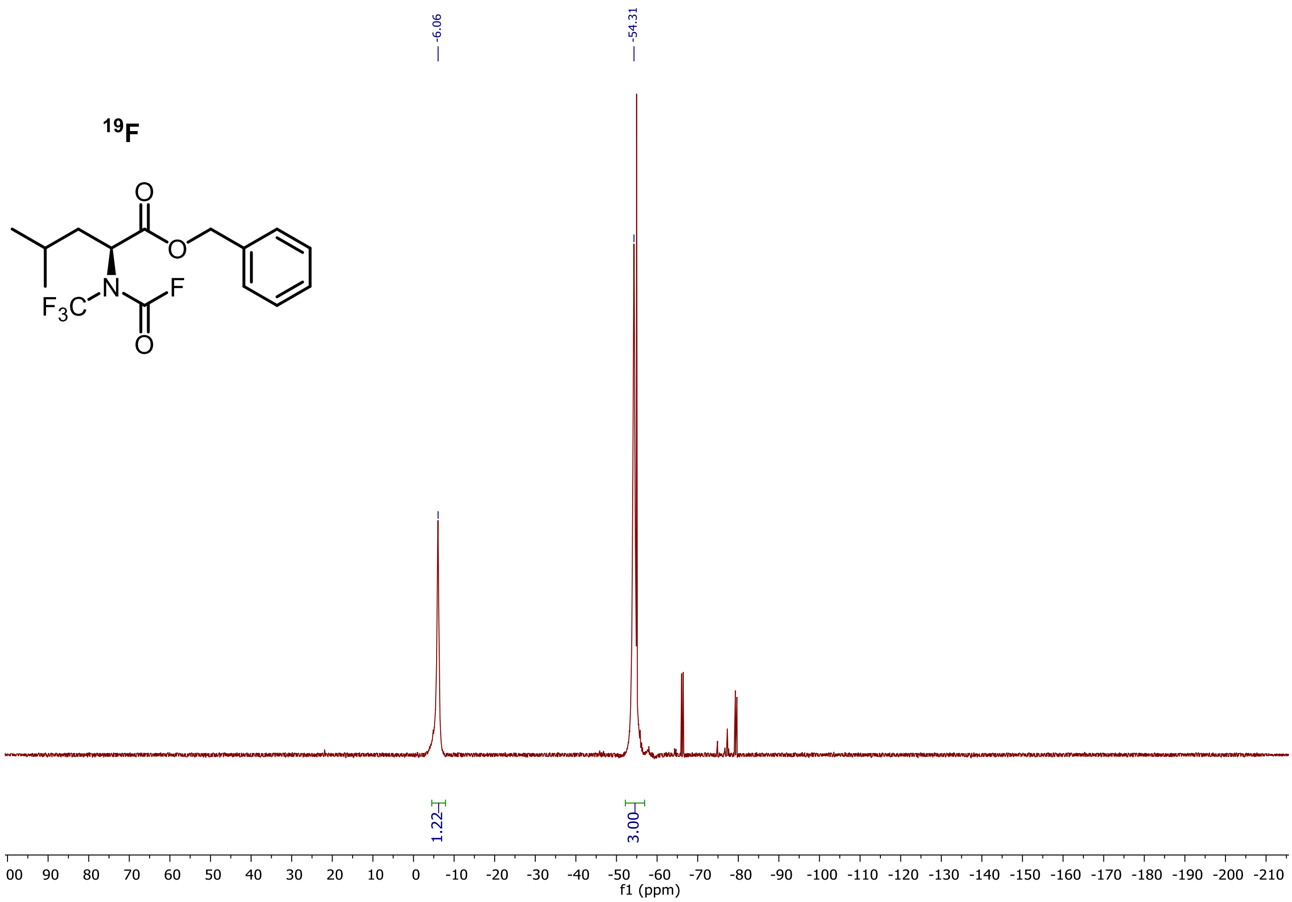

-6.06
-54.31
19F
F3C
N
F
O
O
O
1.22
3.00
00 90 80 70 60 50 40 30 20 10 0 -10 -20 -30 -40 -50 -60 -70 -80 -90 -100 -110 -120 -130 -140 -150 -160 -170 -180 -190 -200 -210
f1 (ppm)

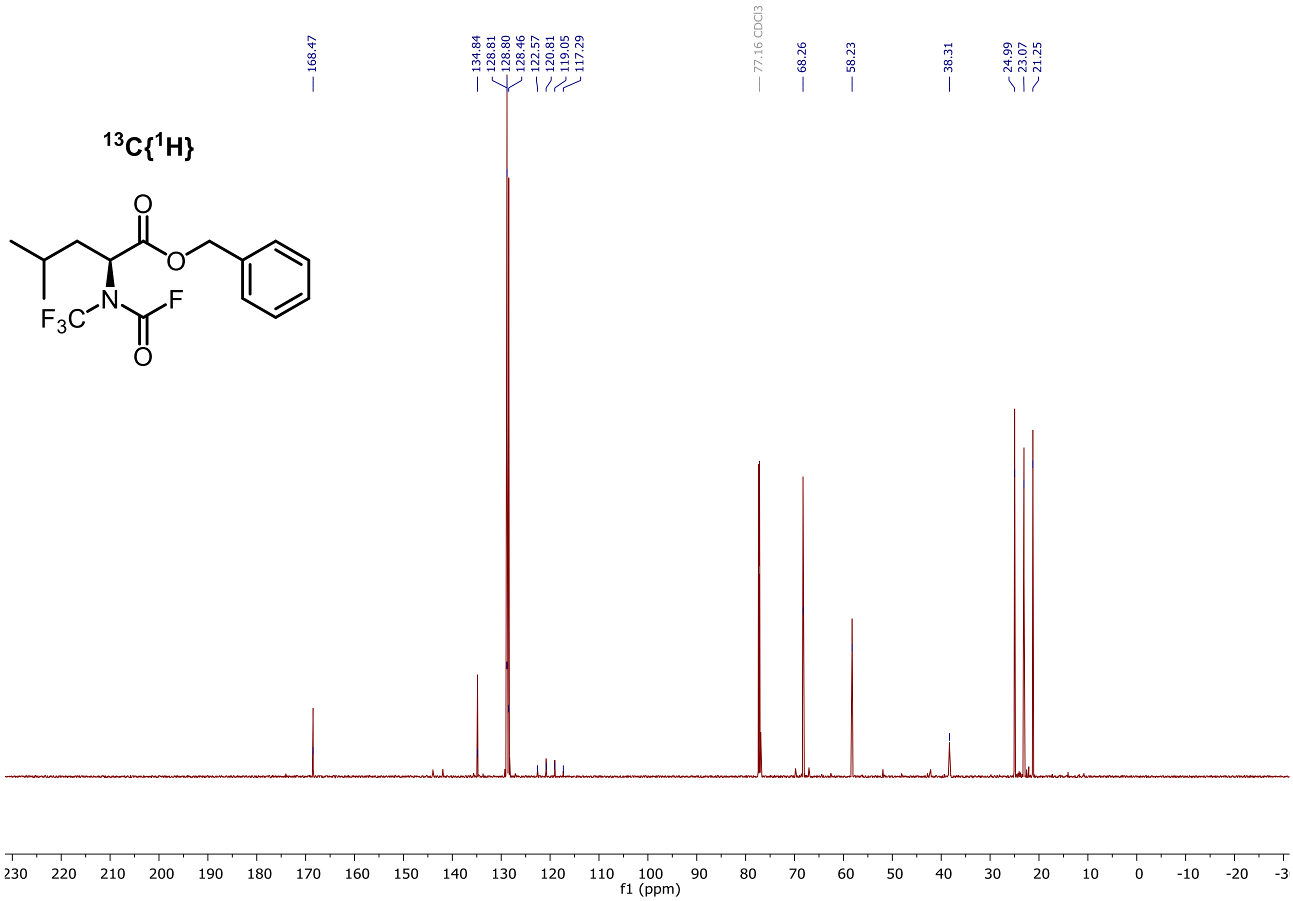

168.47
134.84
128.81
128.80
128.46
122.57
120.81
119.05
117.29
77.16 CDCl3
68.26
58.23
38.31
24.99
23.07
21.25
13C{1H}
F3C
N
F
O
O
O
230 220 210 200 190 180 170 160 150 140 130 120 110 100 90 80 70 60 50 40 30 20 10 0 -10 -20 -3
f1 (ppm)

methyl 4-((fluorocarbonyl)(trifluoromethyl)amino)thiophene-2-carboxylate (*S*23)

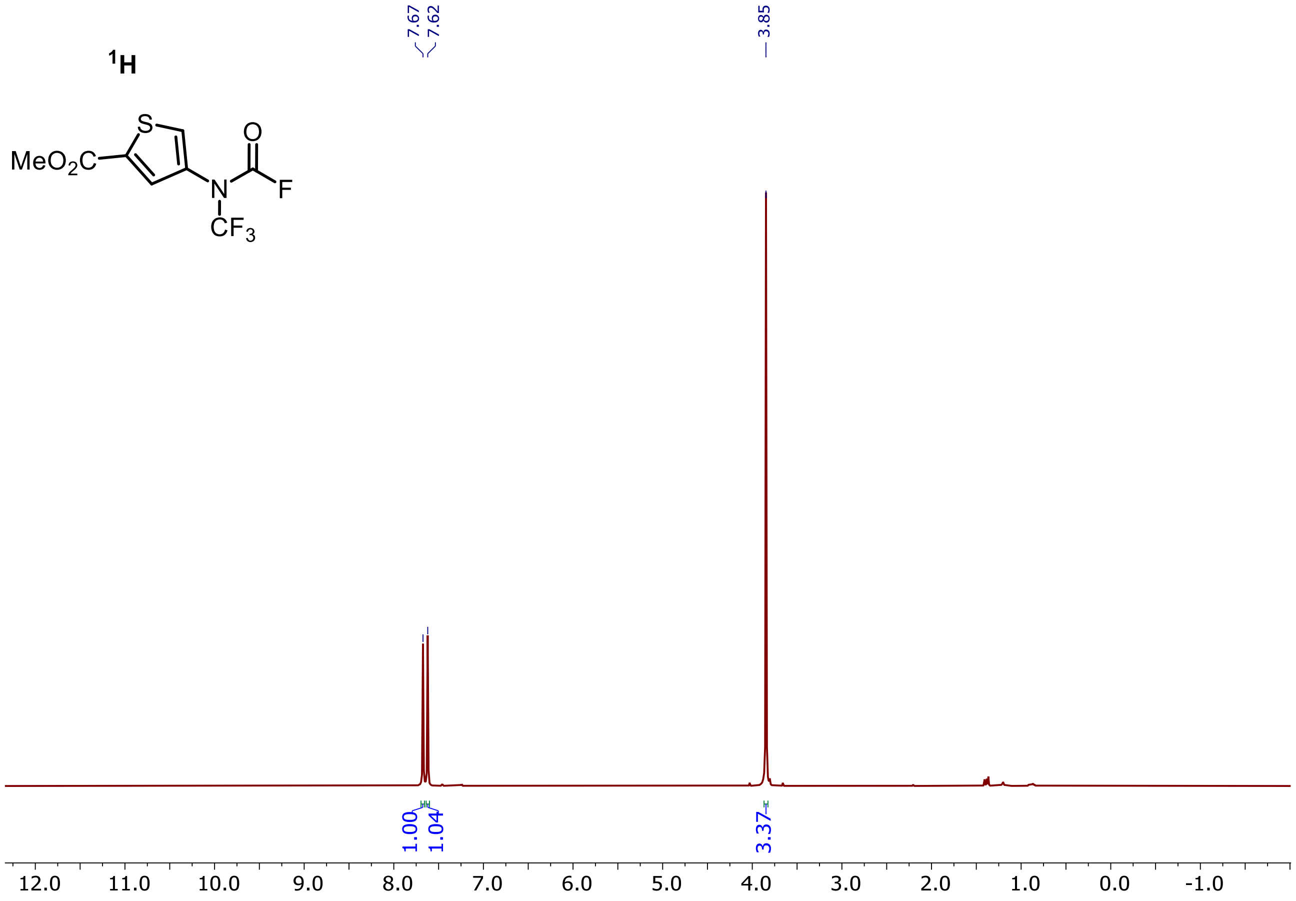

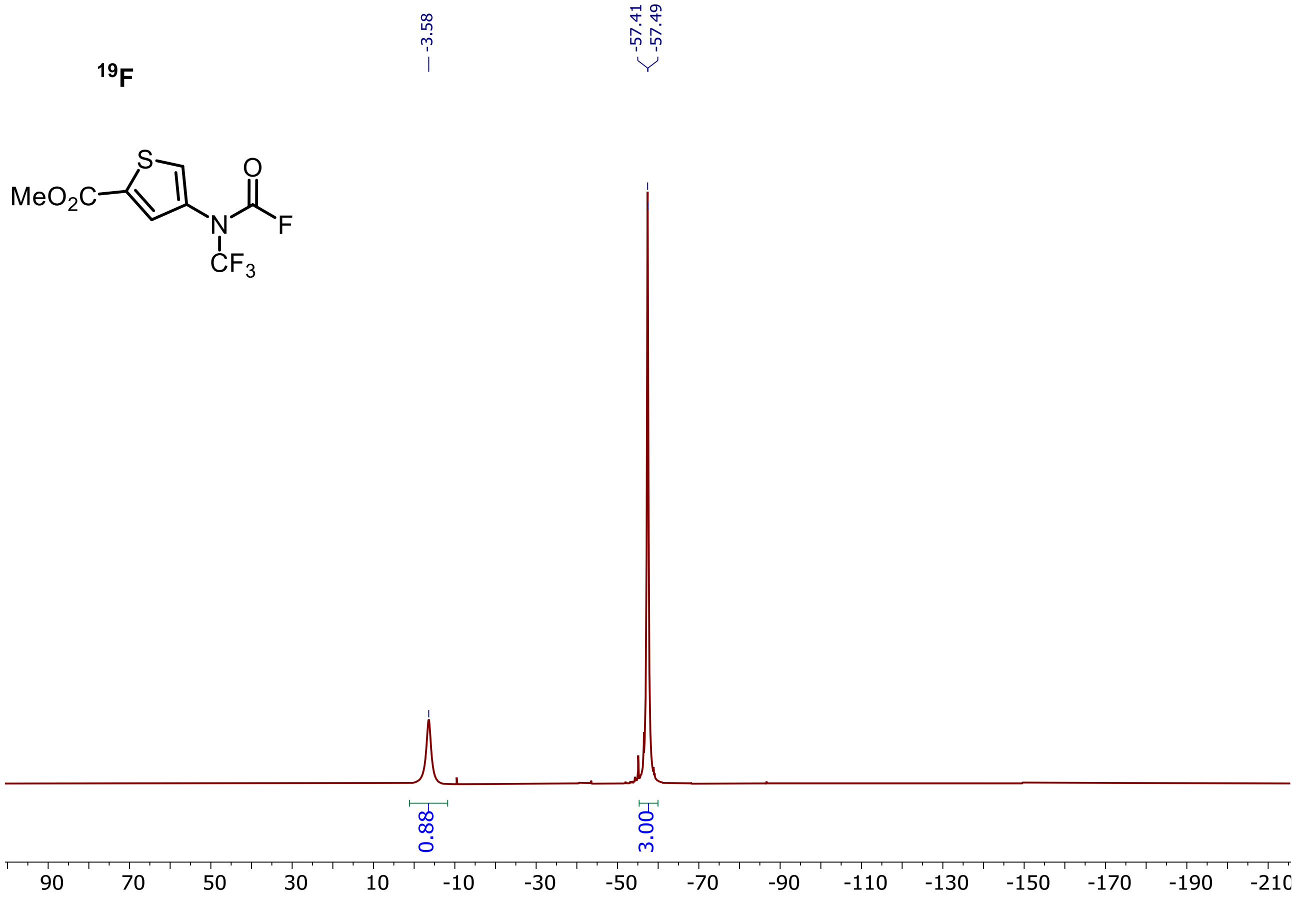

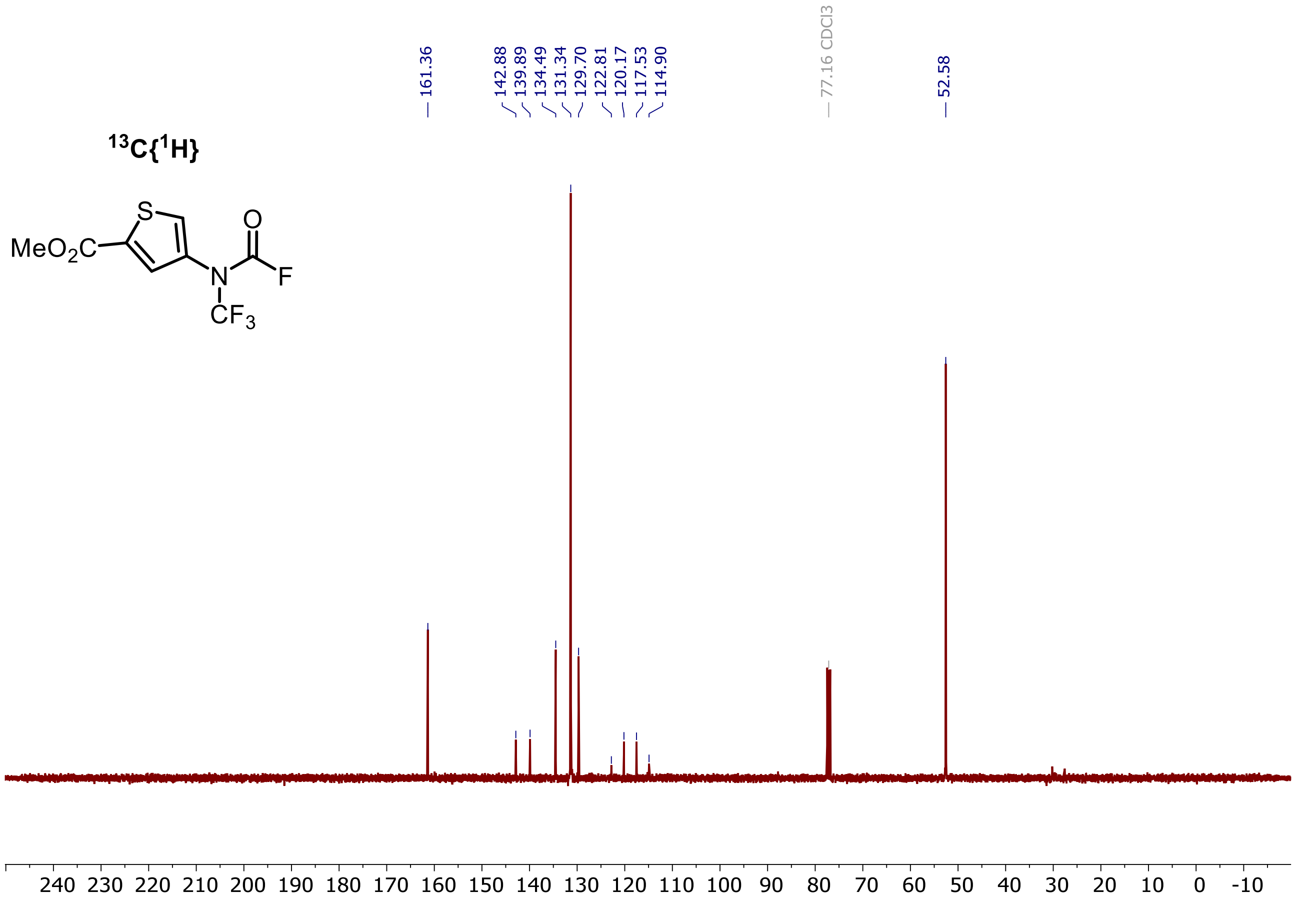

(*trans*-2-phenylcyclopropyl)(trifluoromethyl)carbamoyl fluoride (S24)

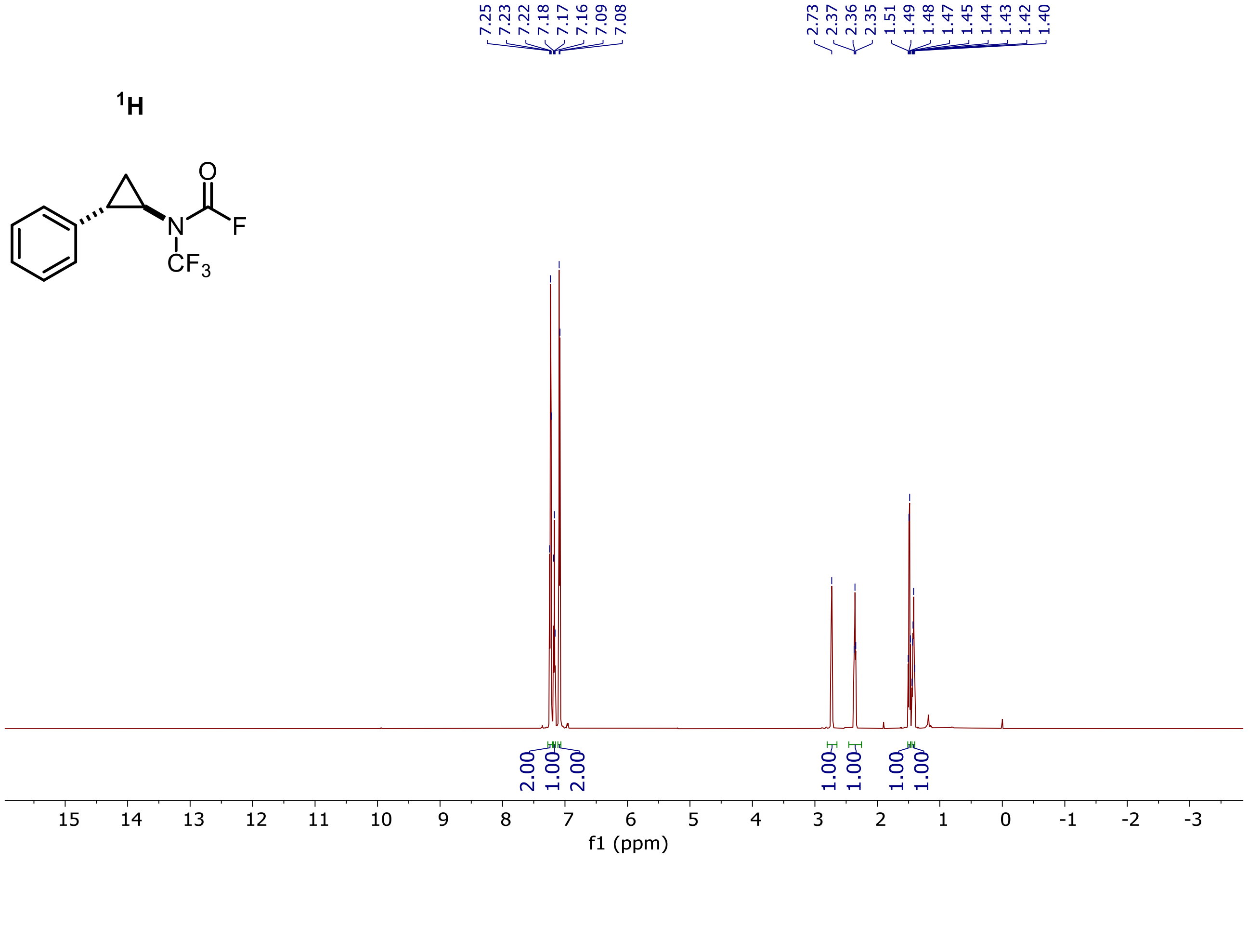

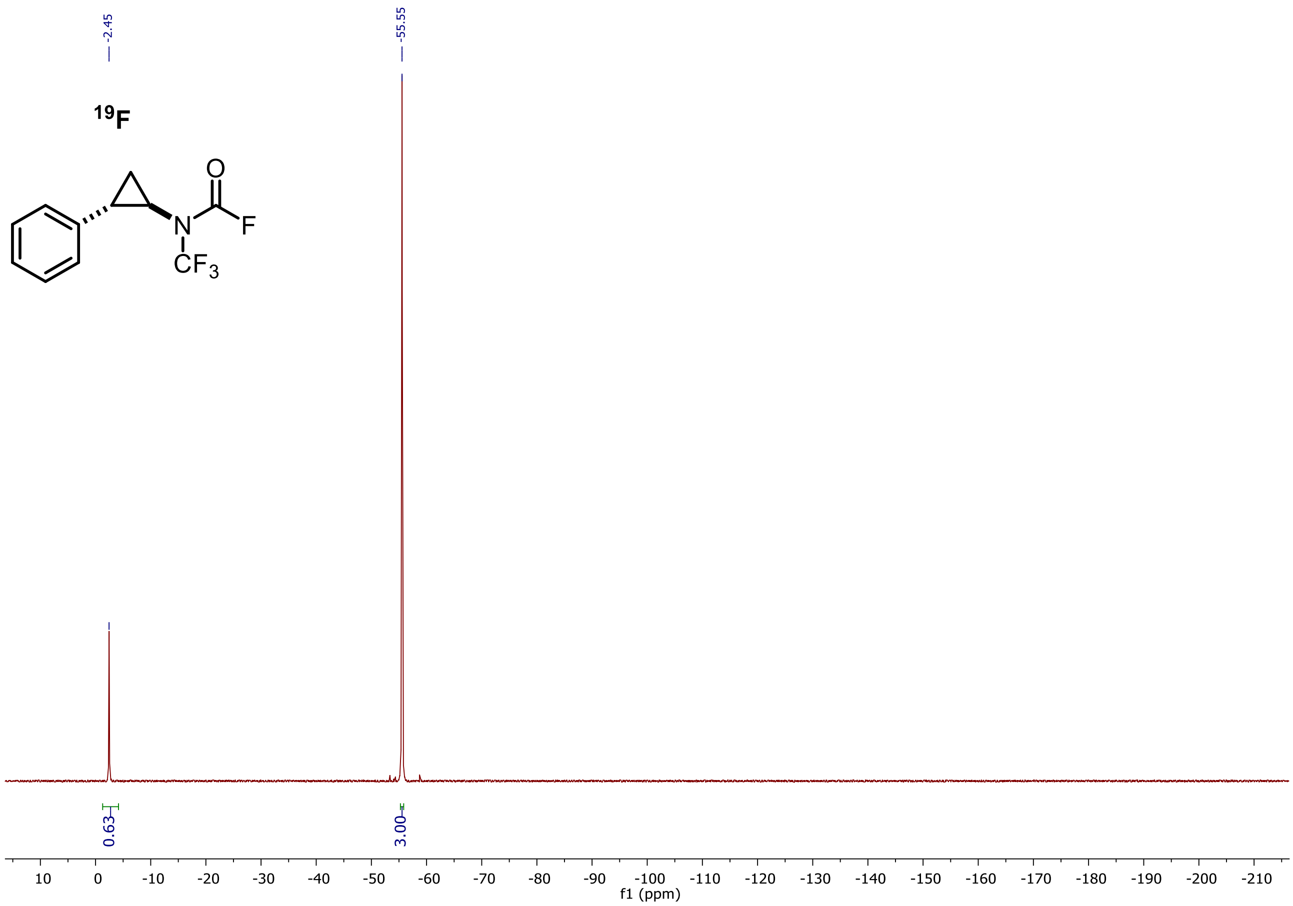
-2.45
-55.55
19F
O
N
F
CF3
0.63
3.00
10
0
-10
-20
-30
-40
-50
-60
-70
-80
-90
-100
-110
-120
-130
-140
-150
-160
-170
-180
-190
-200
-210
f1 (ppm)

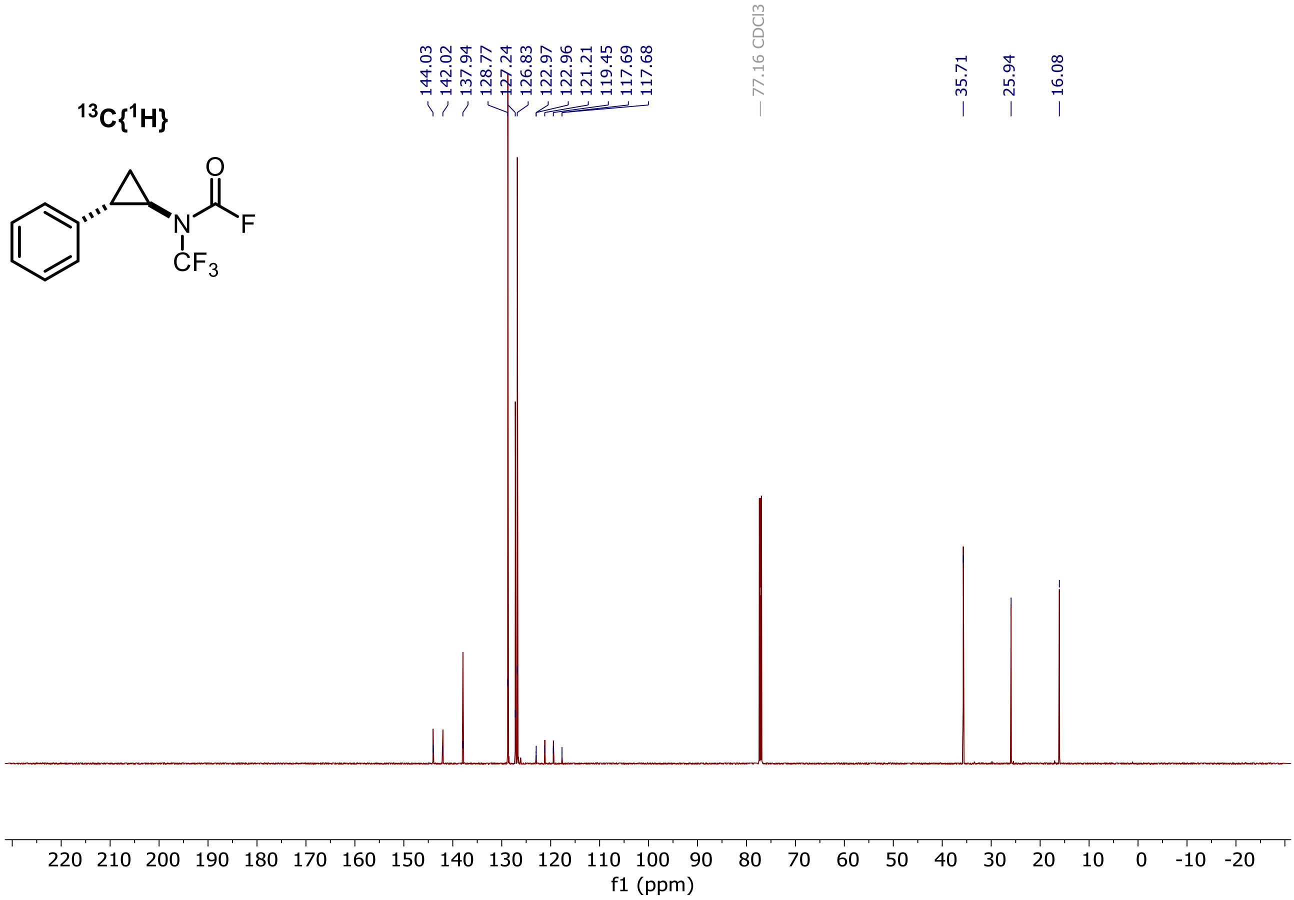
144.03
142.02
137.94
128.77
127.24
126.83
122.97
122.96
121.21
119.45
117.69
117.68
77.16 CDCl3
35.71
25.94
16.08
13C{1H}
O
N
F
CF3
220
210
200
190
180
170
160
150
140
130
120
110
100
90
80
70
60
50
40
30
20
10
0
-10
-20
f1 (ppm)

(*R*)-(1-(3-(2-cyanobenzyl)-1-methyl-2,6-dioxo-1,2,3,6-tetrahydropyrimidin-4-yl)piperidin-3-yl)(trifluoromethyl)carbamoyl fluoride (*S*25)

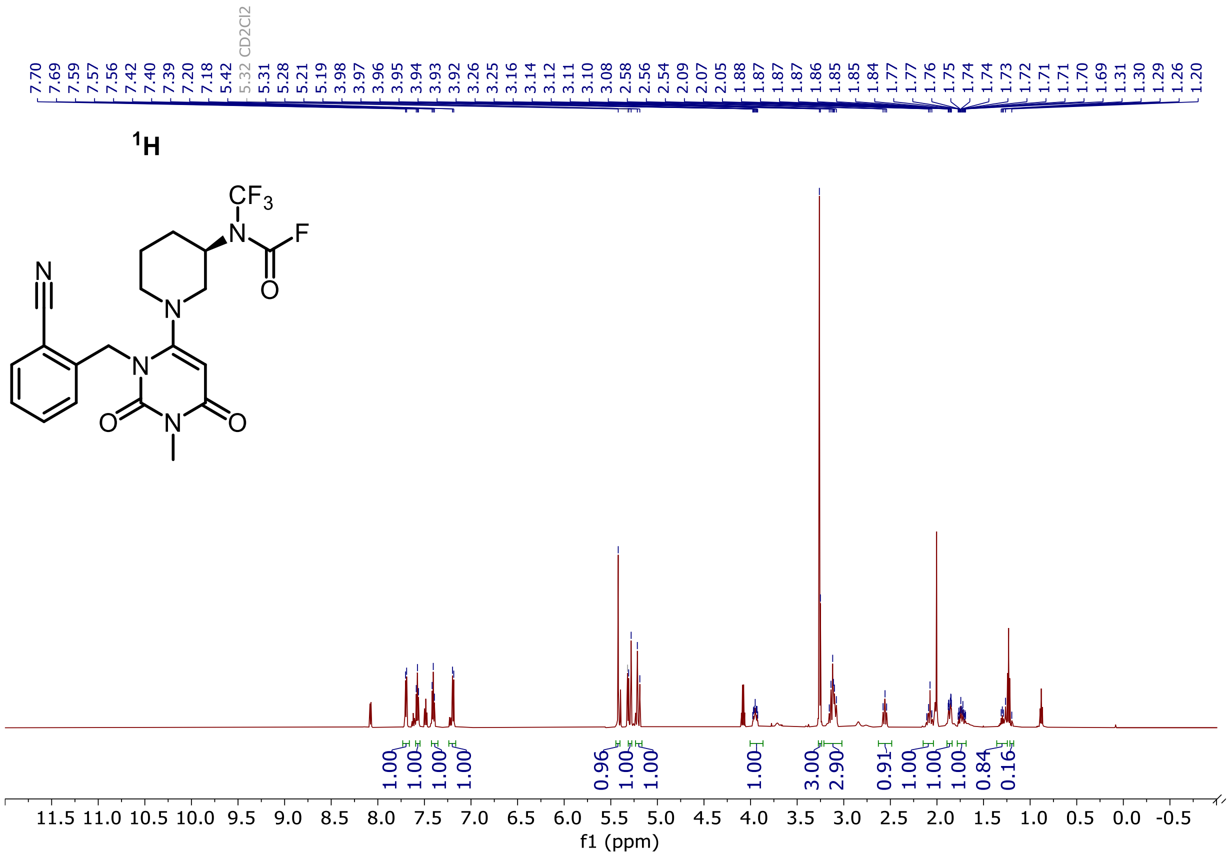

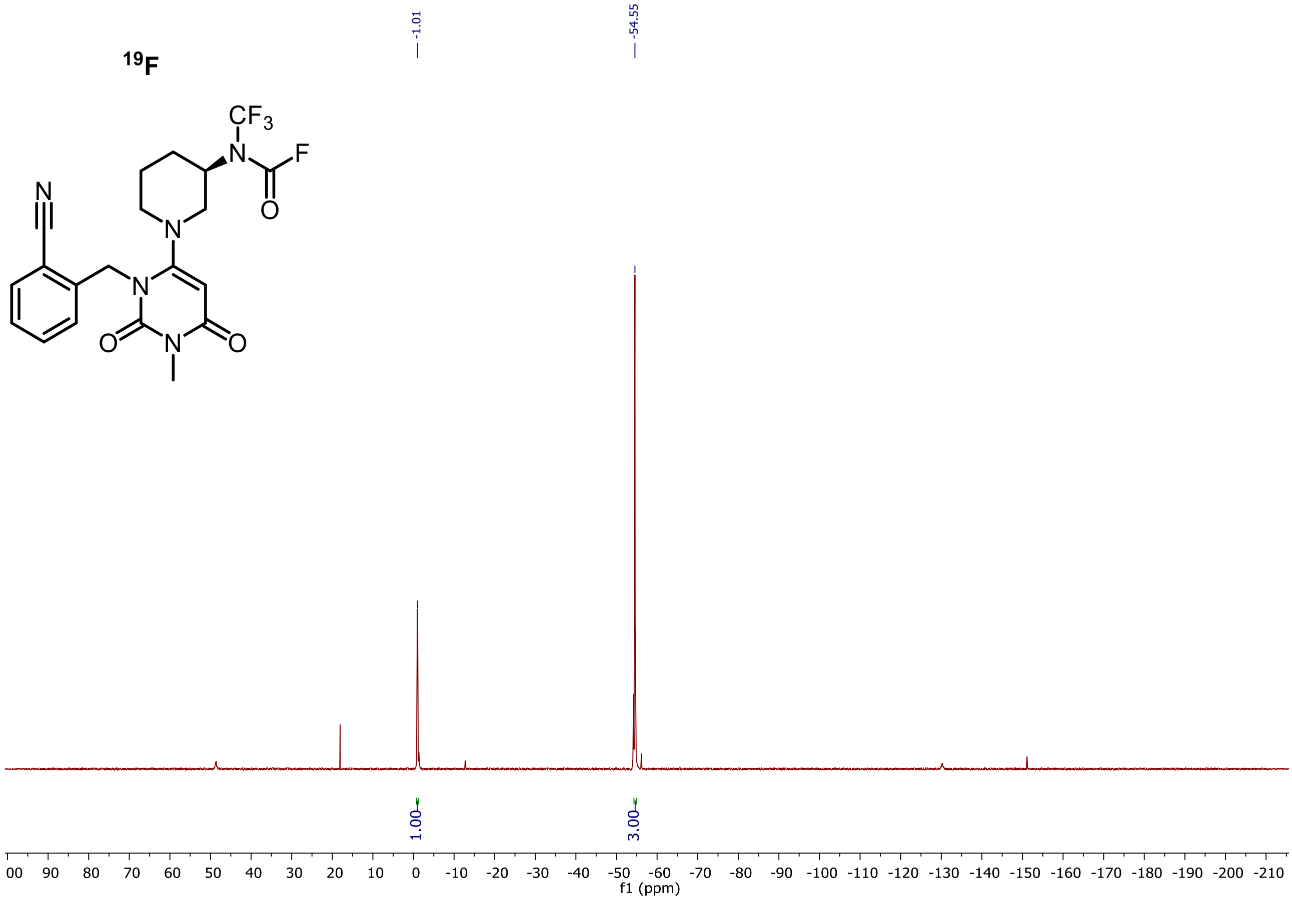

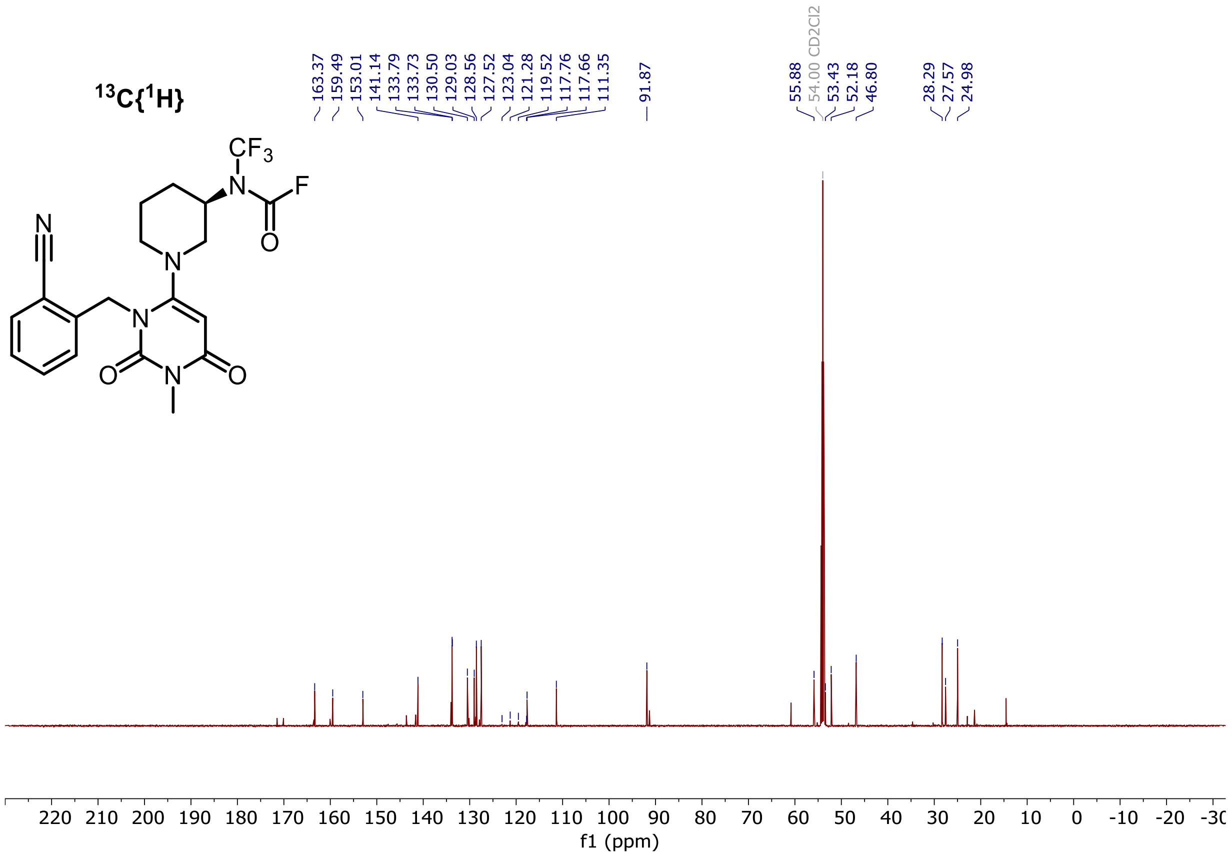
13C{1H}
CF3
N
F
O
N
N
N
O
N
O
163.37
159.49
153.01
141.14
133.79
133.73
130.50
129.03
128.56
127.52
123.04
121.28
119.52
117.76
117.66
111.35
91.87
55.88
54.00 CD2Cl2
53.43
52.18
46.80
28.29
27.57
24.98
220 210 200 190 180 170 160 150 140 130 120 110 100 90 80 70 60 50 40 30 20 10 0 -10 -20 -30
f1 (ppm)

12.2.4 *N*-CF_3 (deutero)formamides

N-phenyl-*N*-(trifluoromethyl)formamide (S26)

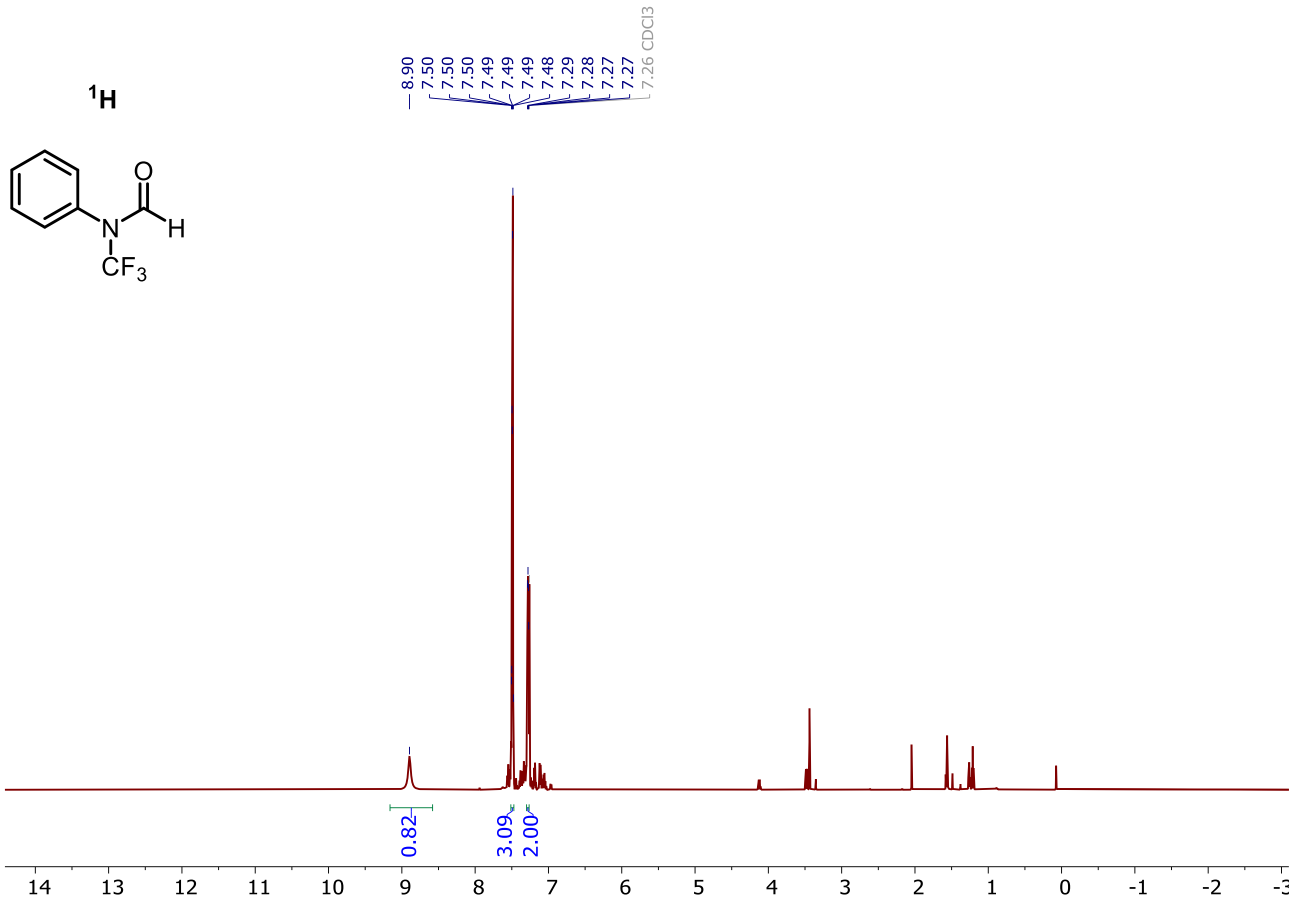

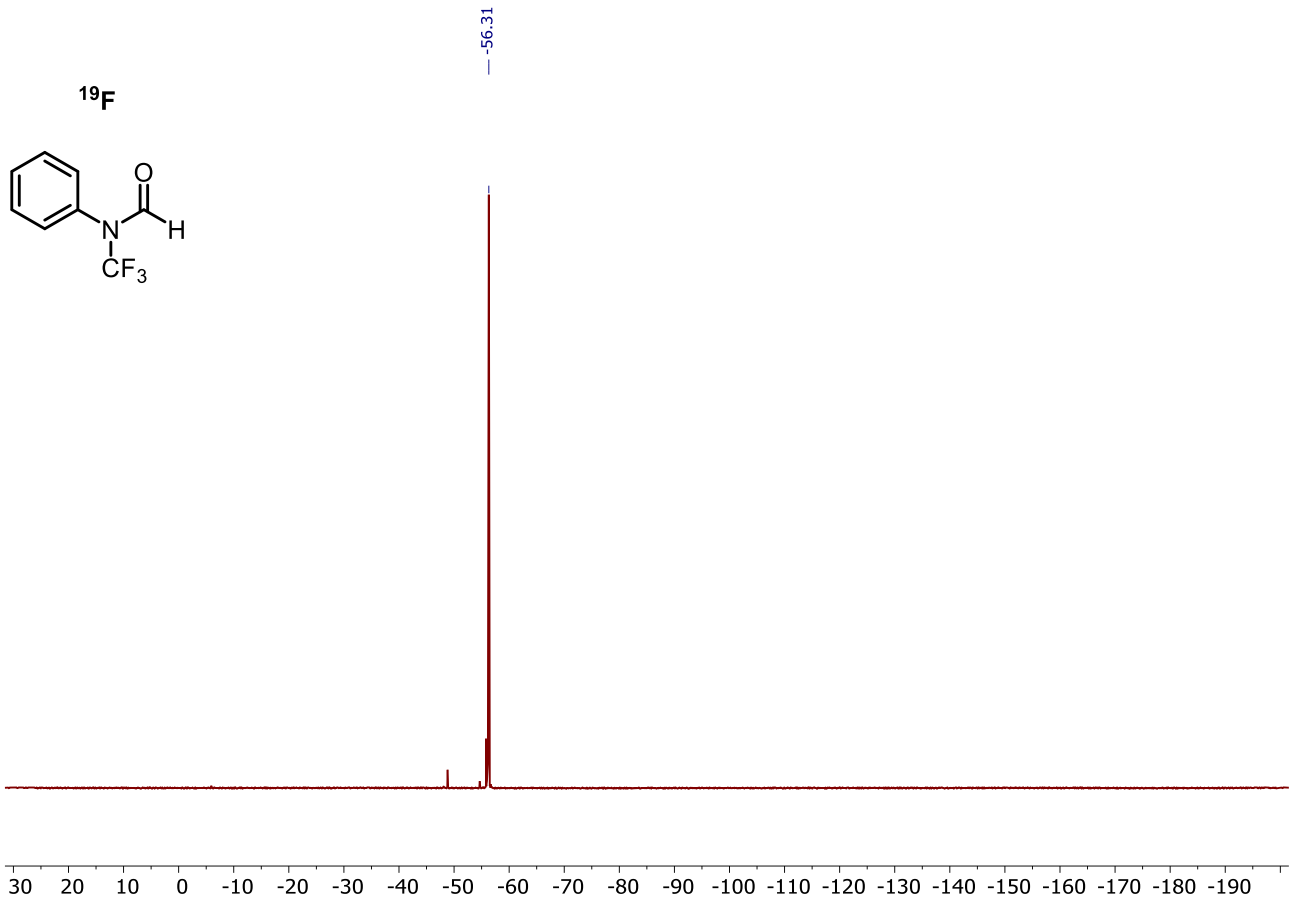

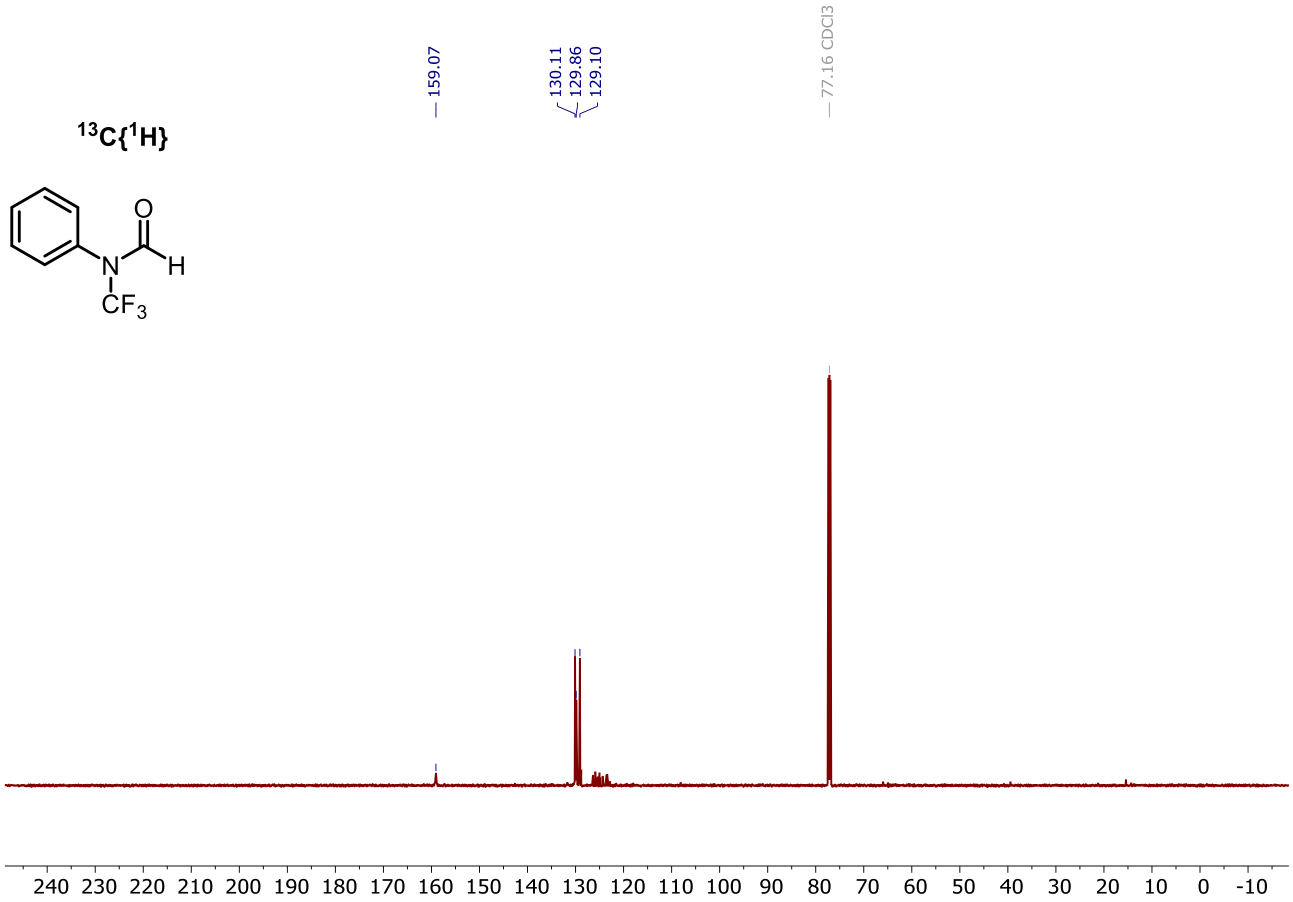

N-(4-cyclohexylphenyl)-N-(trifluoromethyl)formamide (S27)

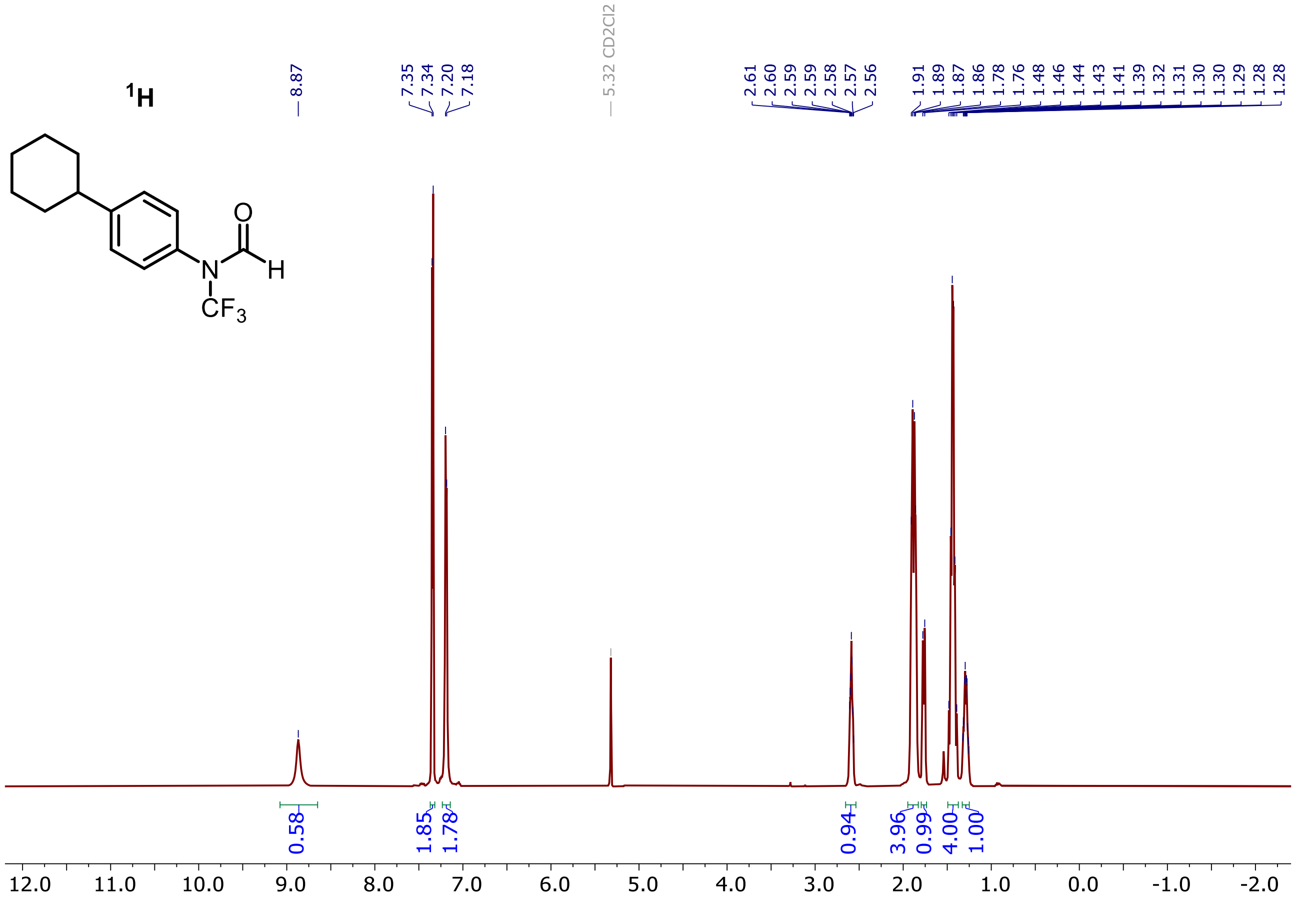

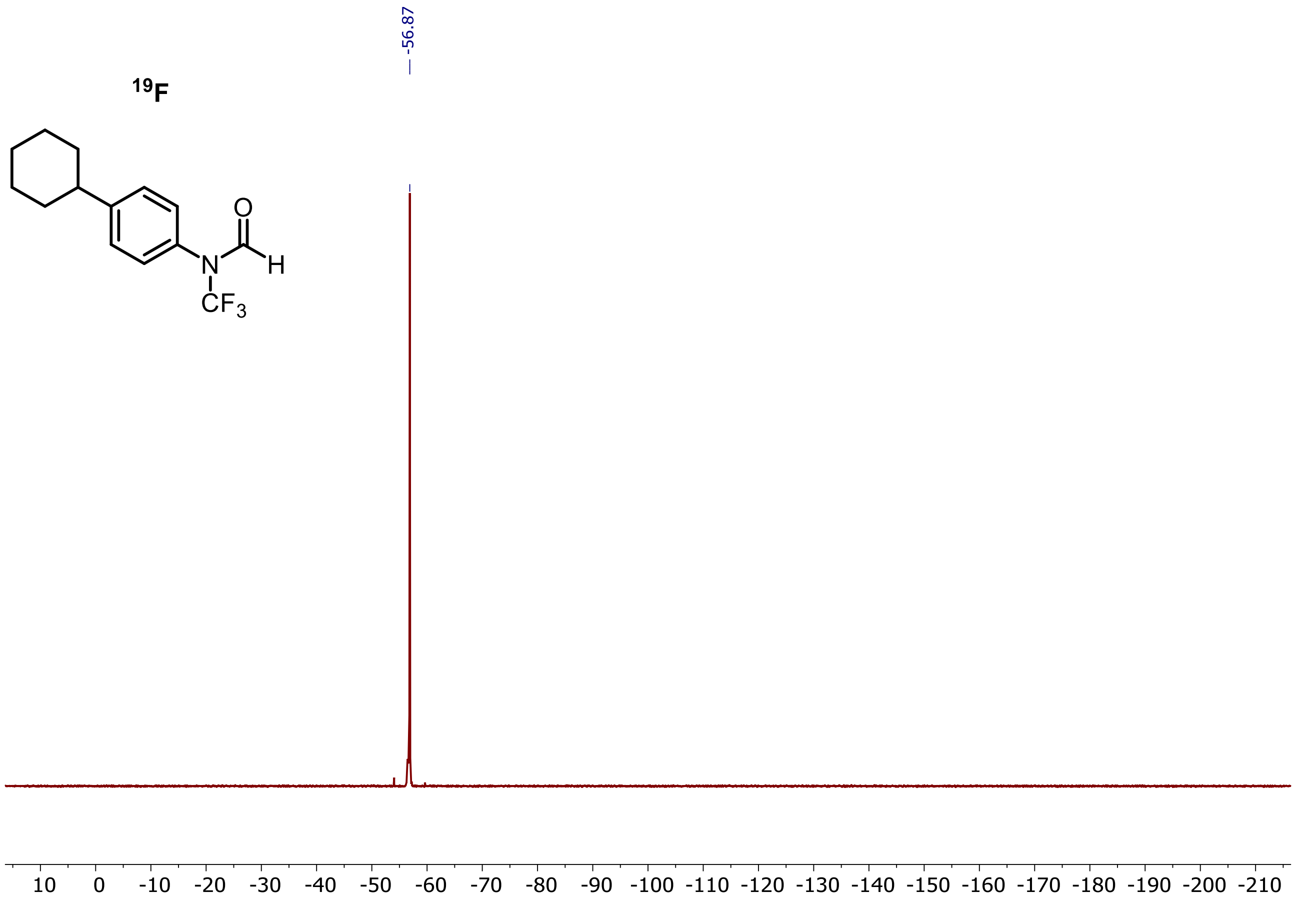

19F
O
N
H
CF3
-56.87
10 0 -10 -20 -30 -40 -50 -60 -70 -80 -90 -100 -110 -120 -130 -140 -150 -160 -170 -180 -190 -200 -210

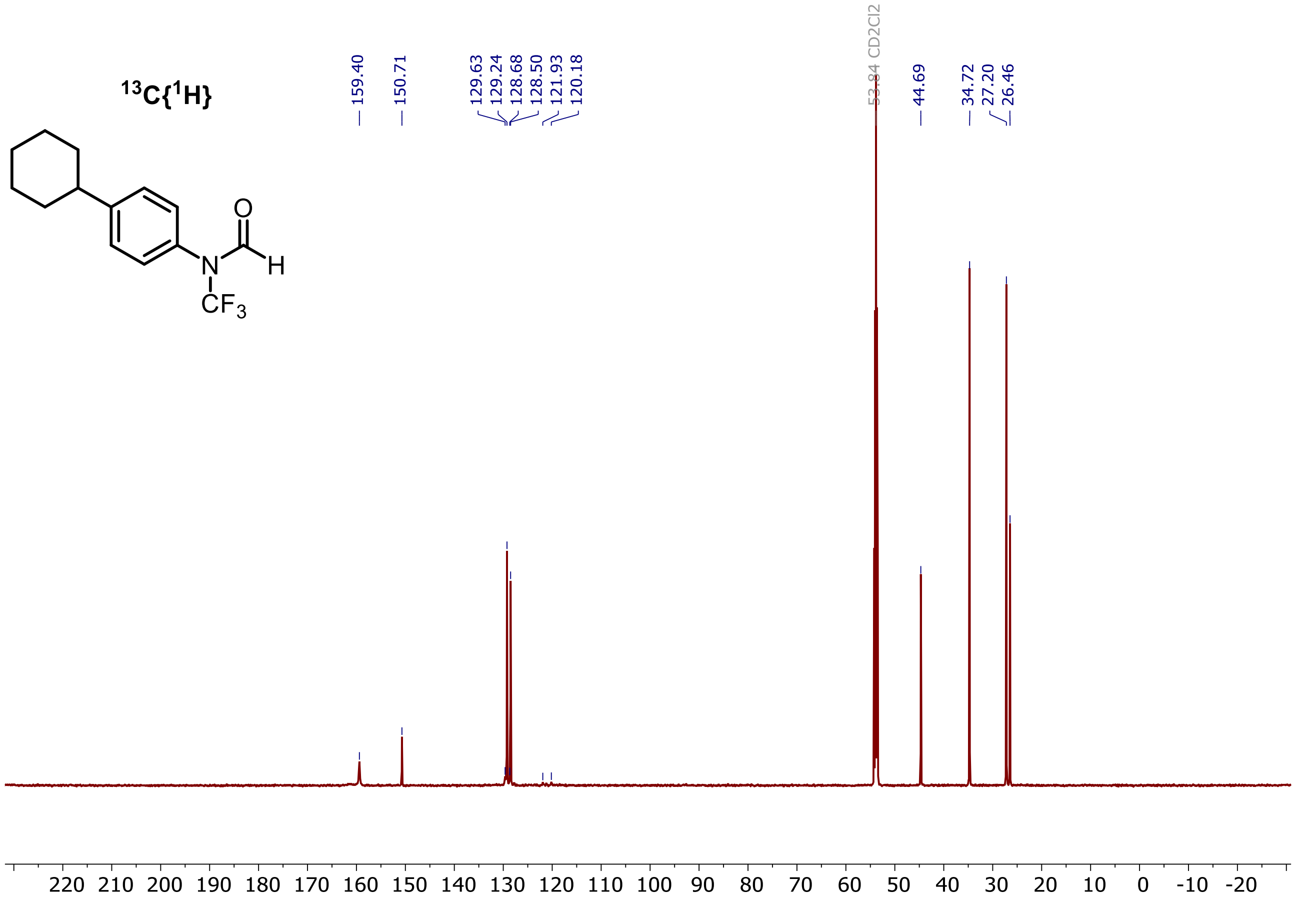

13C{1H}
O
N
H
CF3
159.40
150.71
129.63
129.24
128.68
128.50
121.93
120.18
53.84 CD2Cl2
44.69
34.72
27.20
26.46
220 210 200 190 180 170 160 150 140 130 120 110 100 90 80 70 60 50 40 30 20 10 0 -10 -20

N-(4-bromo-2-chlorophenyl)-*N*-(trifluoromethyl)formamide (*S*28)

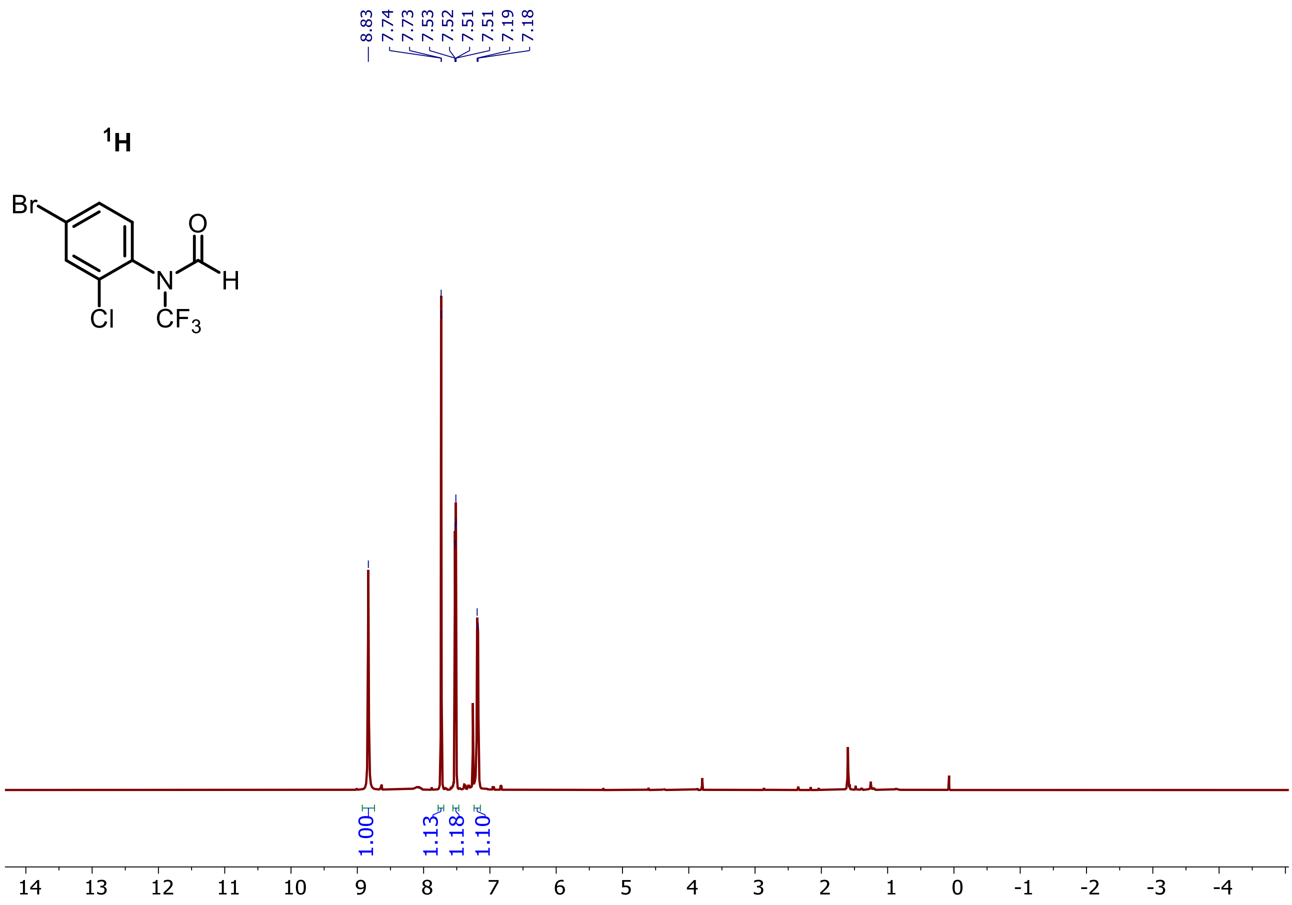

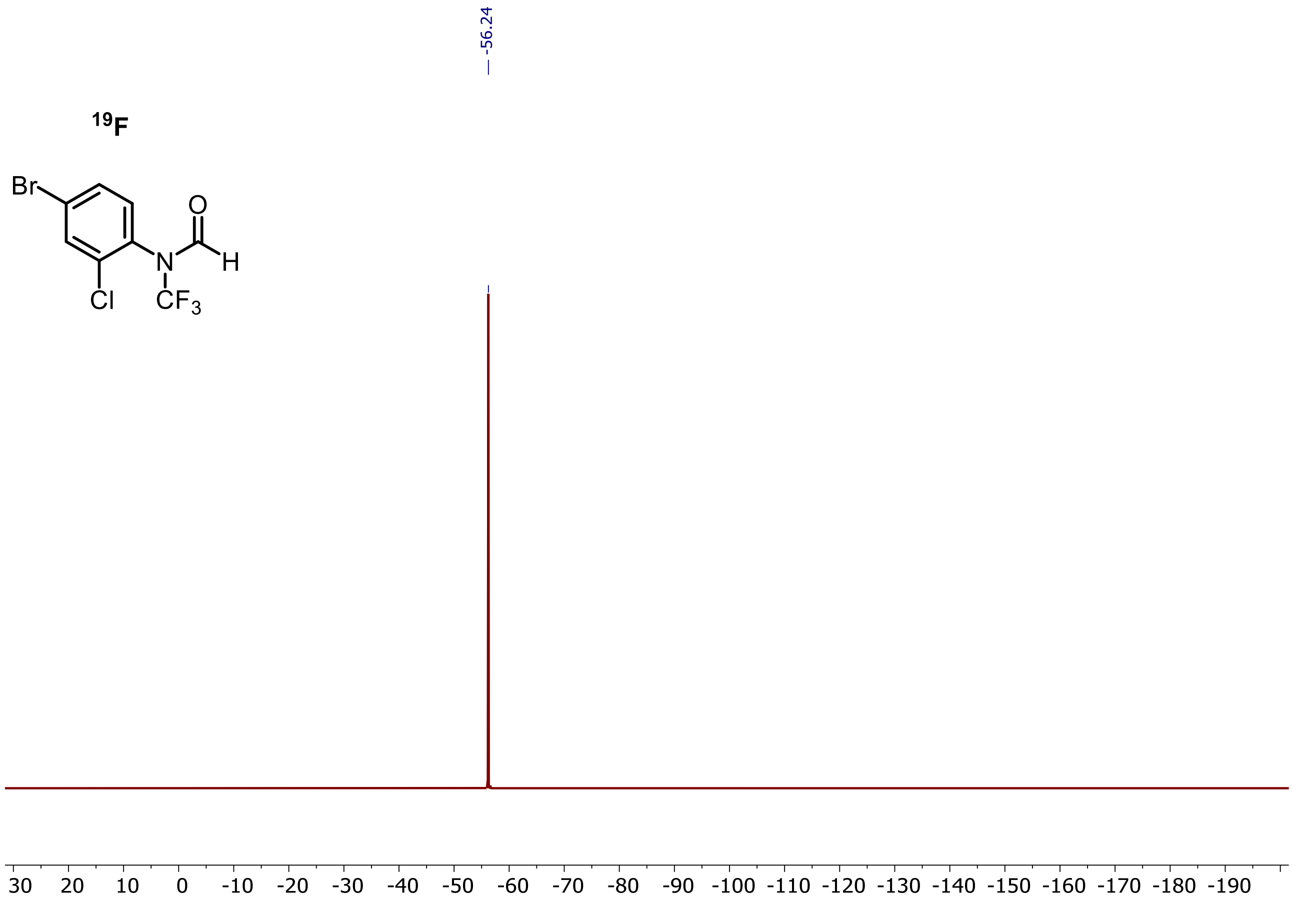

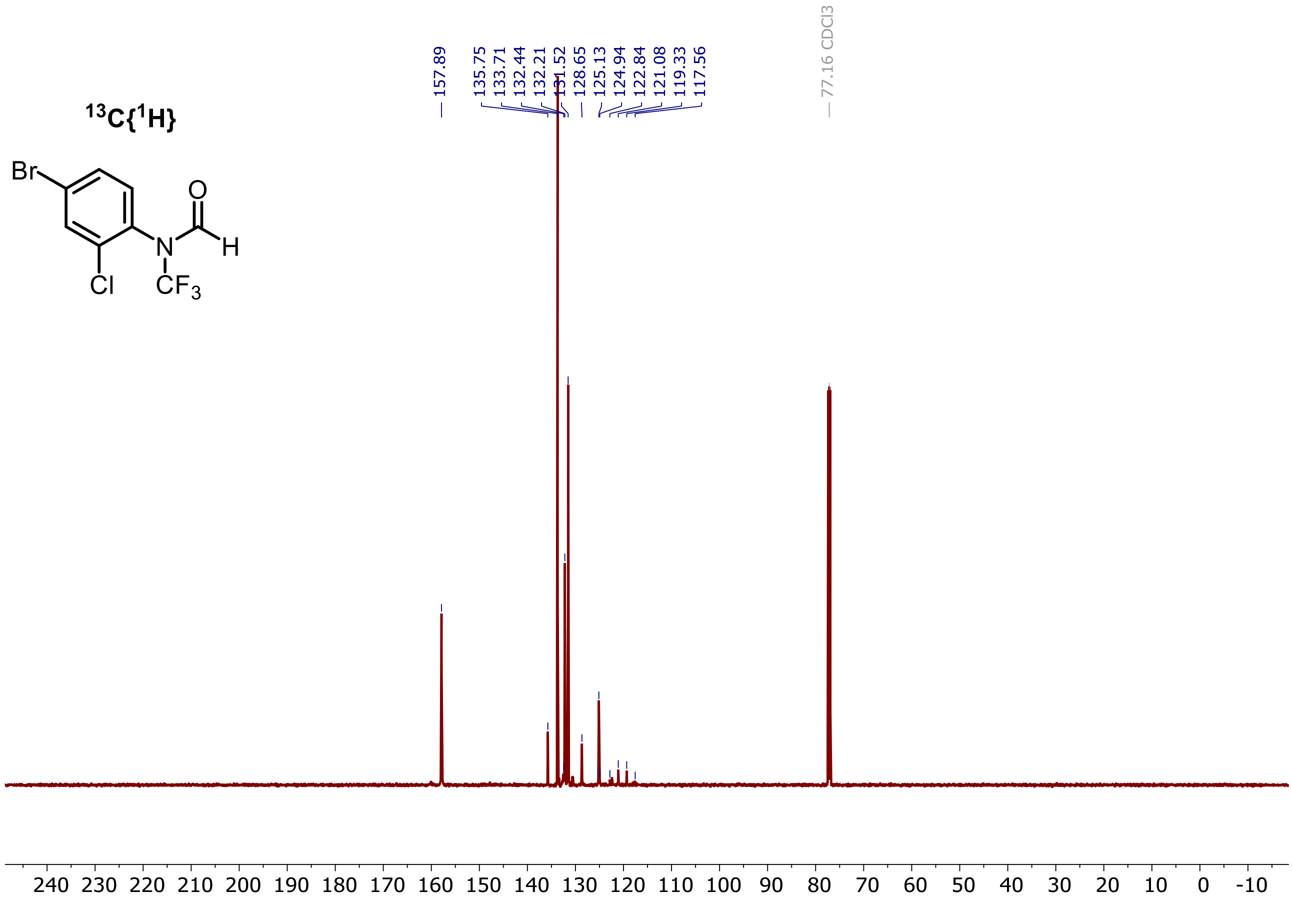

methyl 4-((difluoromethyl)(trifluoromethyl)amino)benzoate (S29)

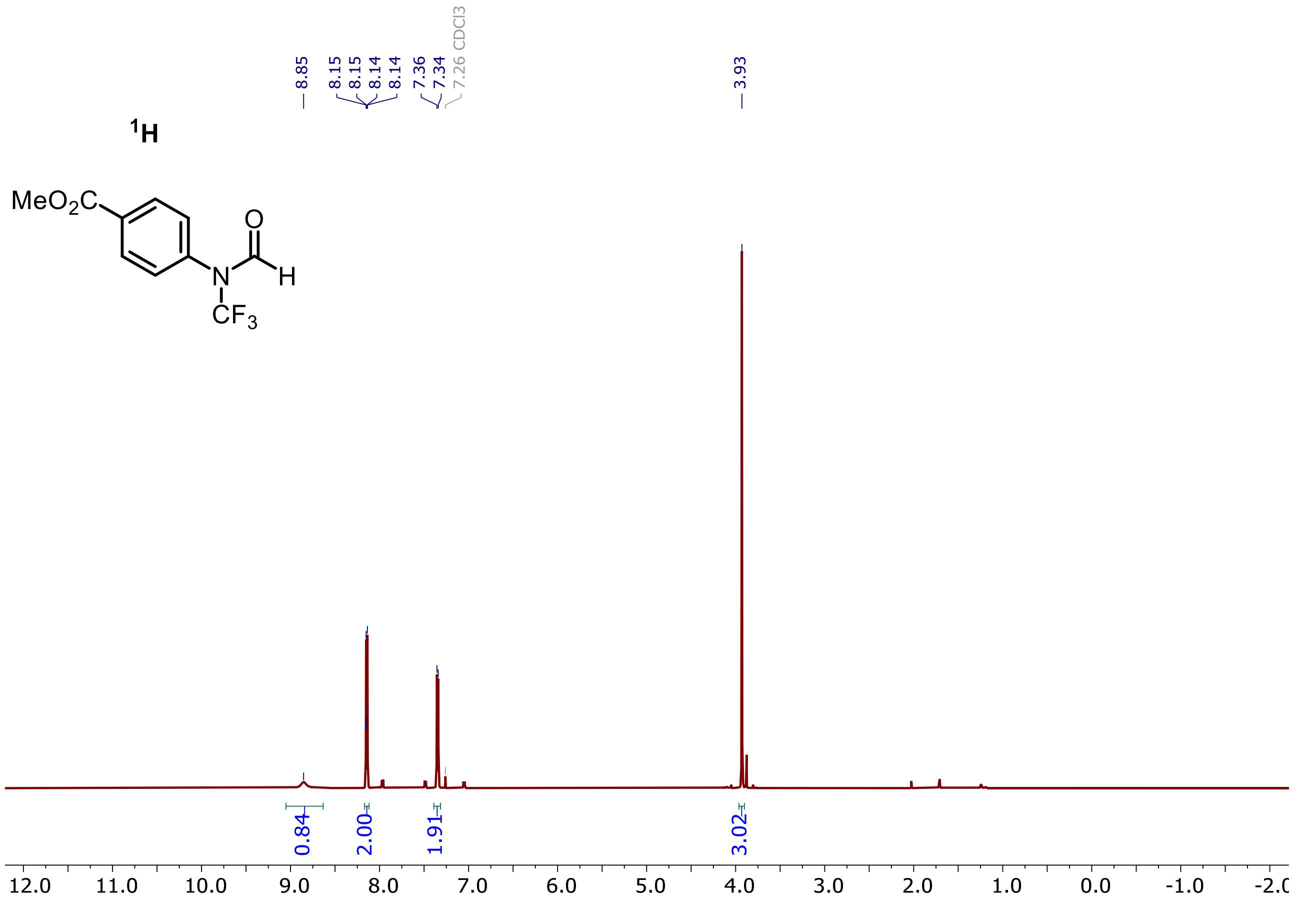

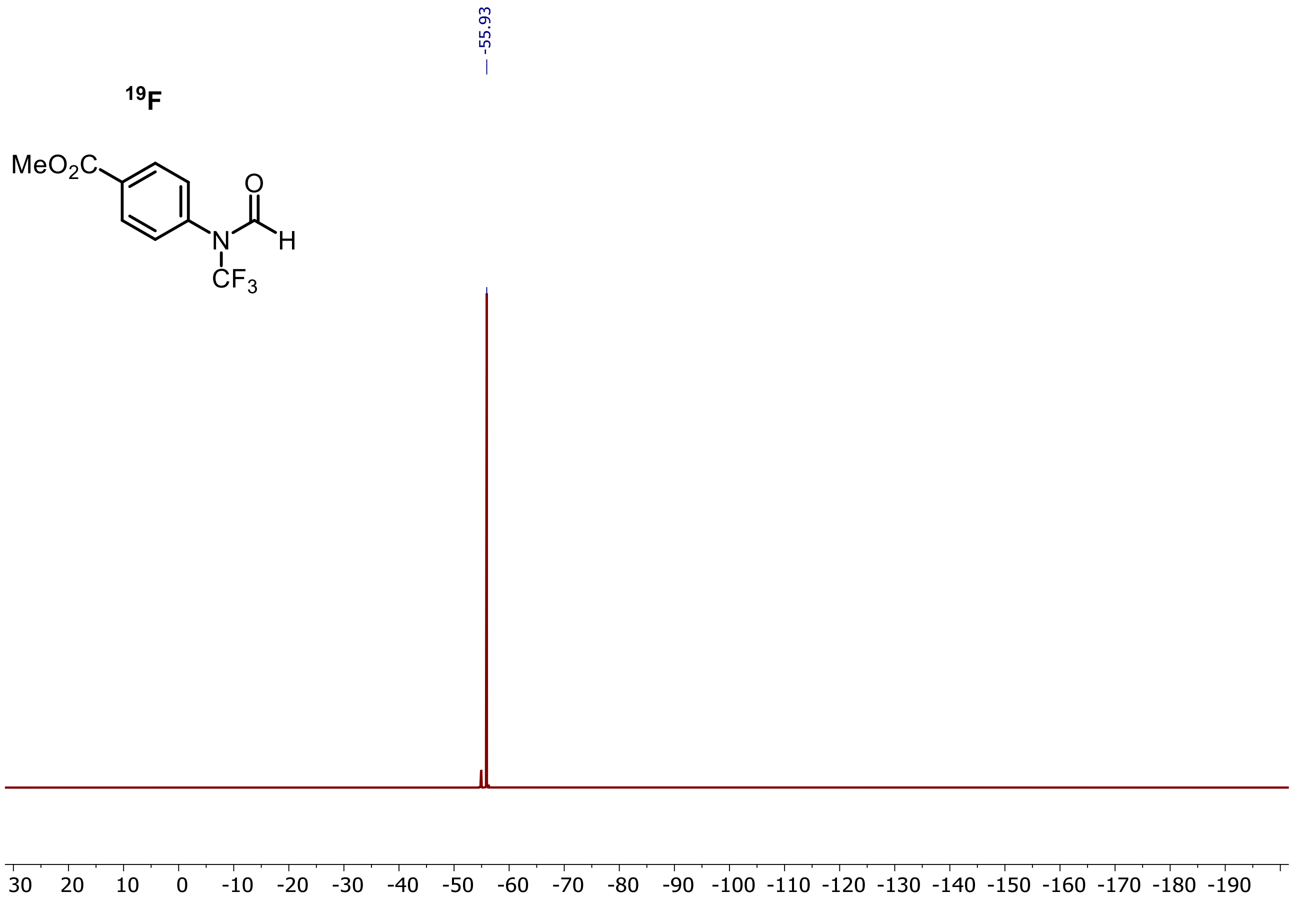

-55.93
19F
MeO2C
O
H
N
CF3
30 20 10 0 -10 -20 -30 -40 -50 -60 -70 -80 -90 -100 -110 -120 -130 -140 -150 -160 -170 -180 -190

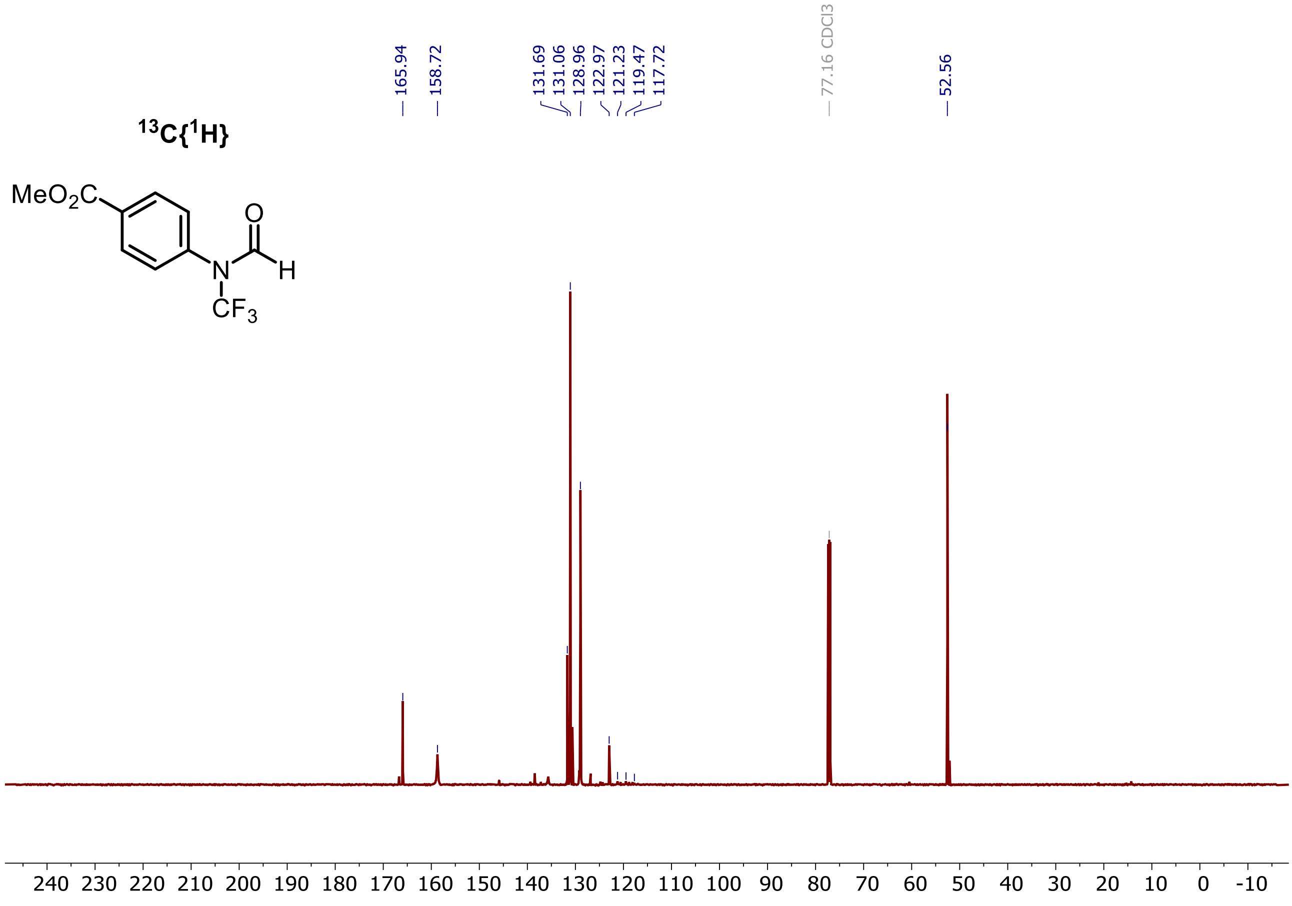

165.94
158.72
131.69
131.06
128.96
122.97
121.23
119.47
117.72
77.16 CDCl3
52.56
13C{1H}
MeO2C
O
H
N
CF3
240 230 220 210 200 190 180 170 160 150 140 130 120 110 100 90 80 70 60 50 40 30 20 10 0 -10

benzyl *N*-formyl-*N*-(trifluoromethyl)-*L*-leucinate (*S*30)

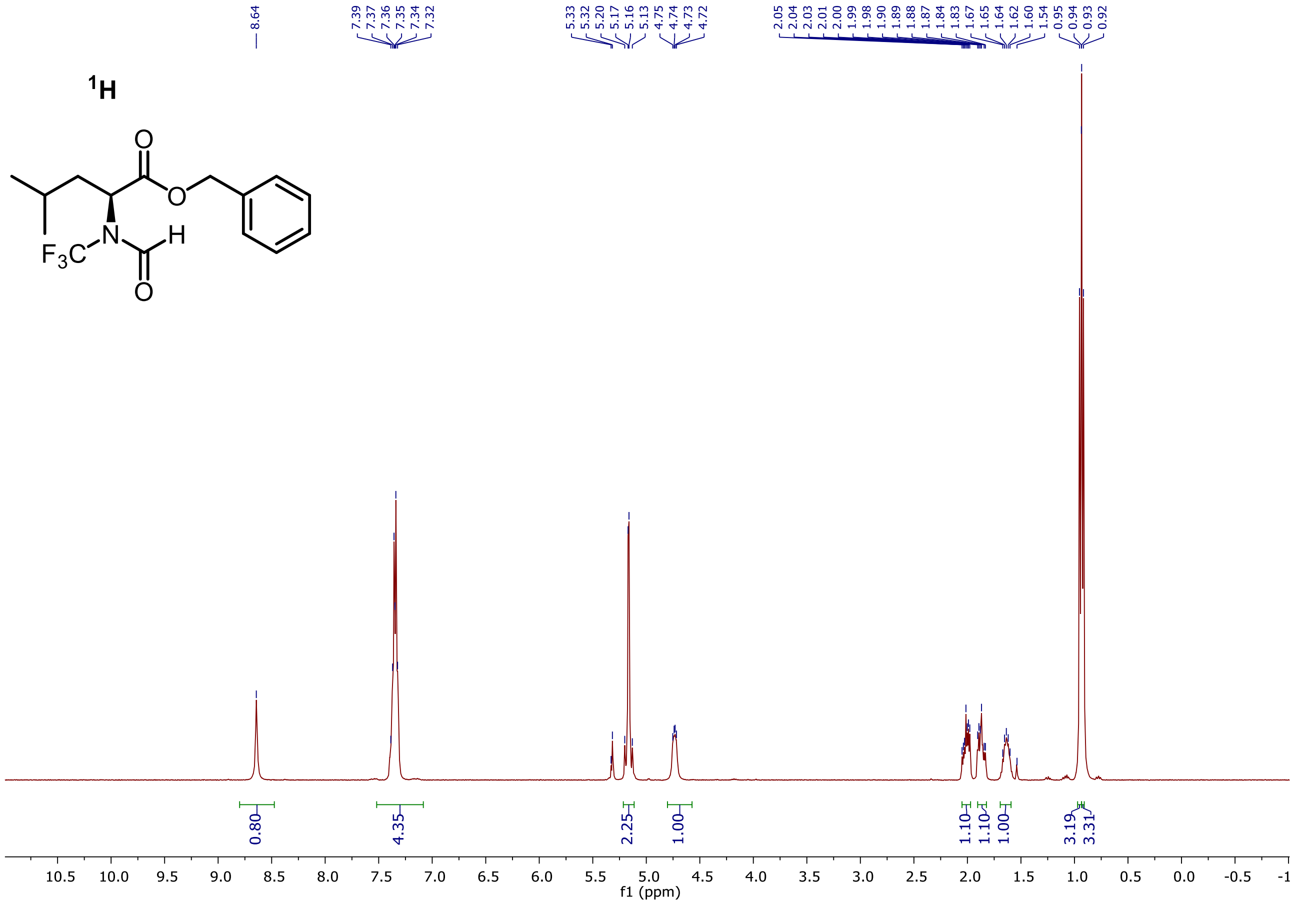

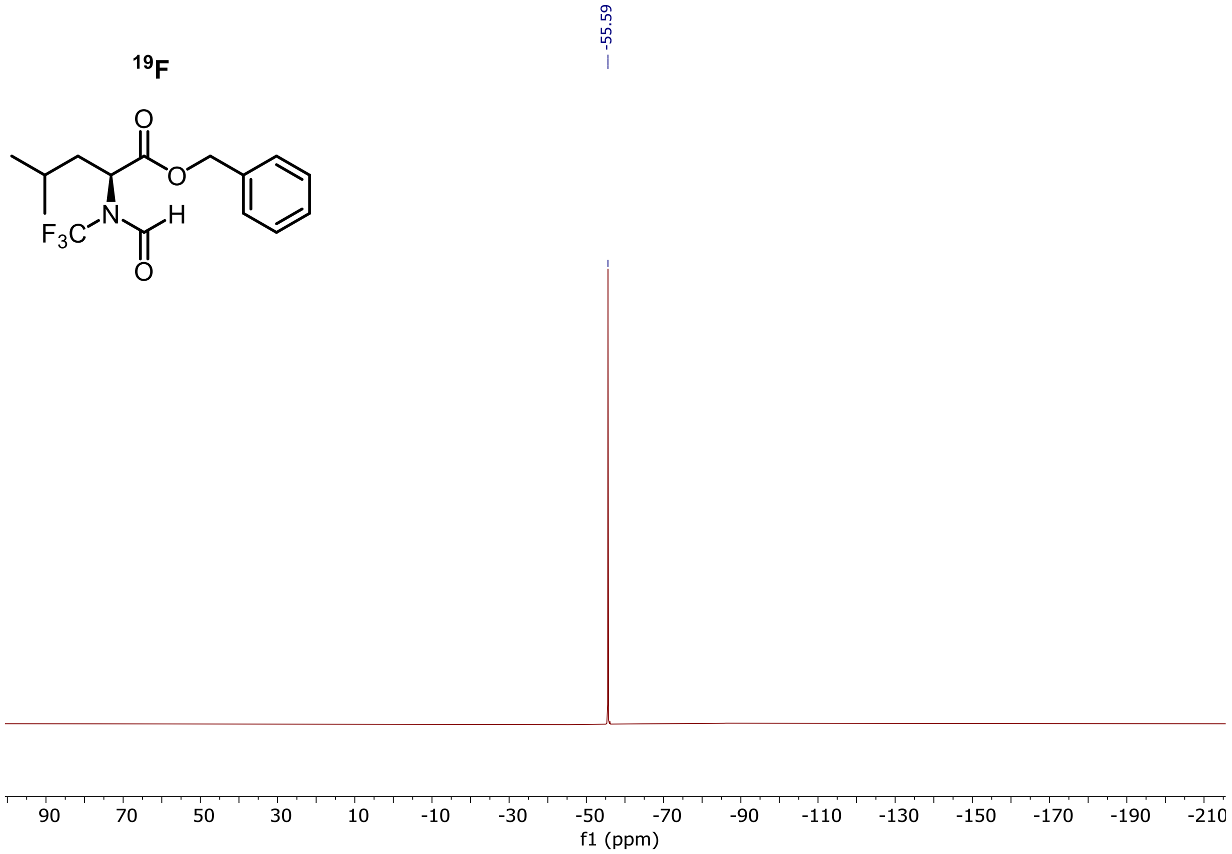

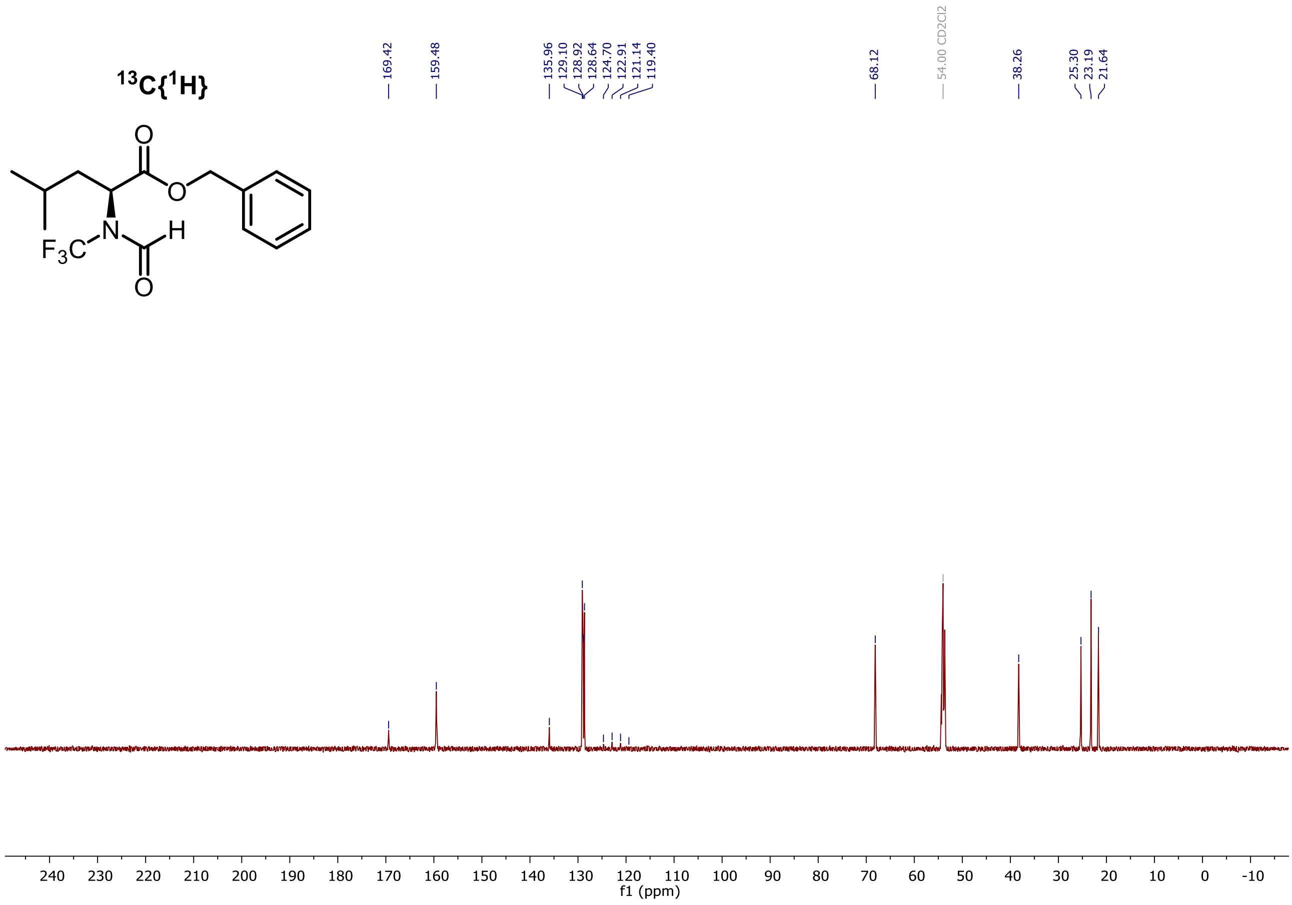

tert-butyl 4-(*N*-(trifluoromethyl)formamido-*d*)piperidine-1-carboxylate (*S*31)

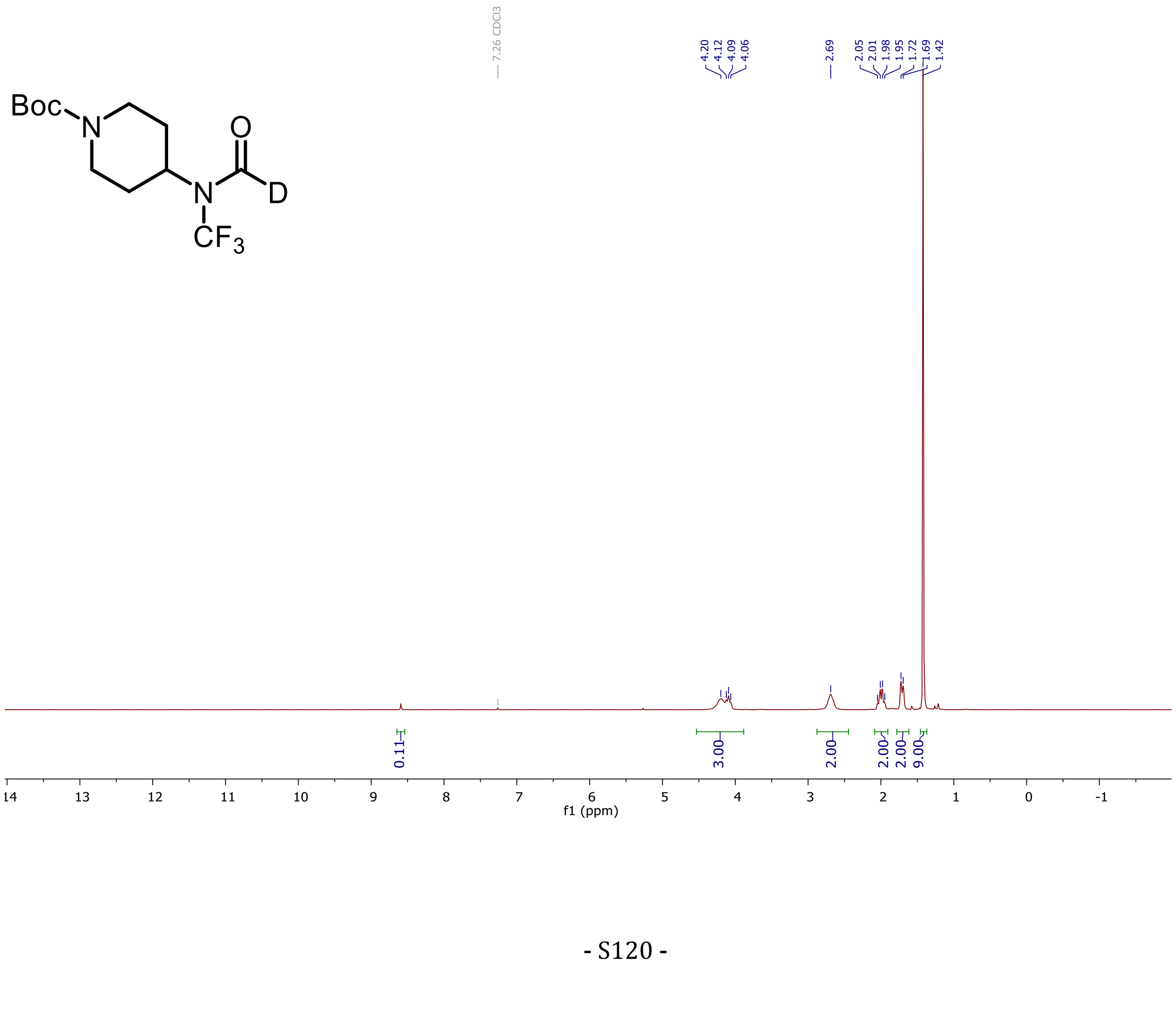

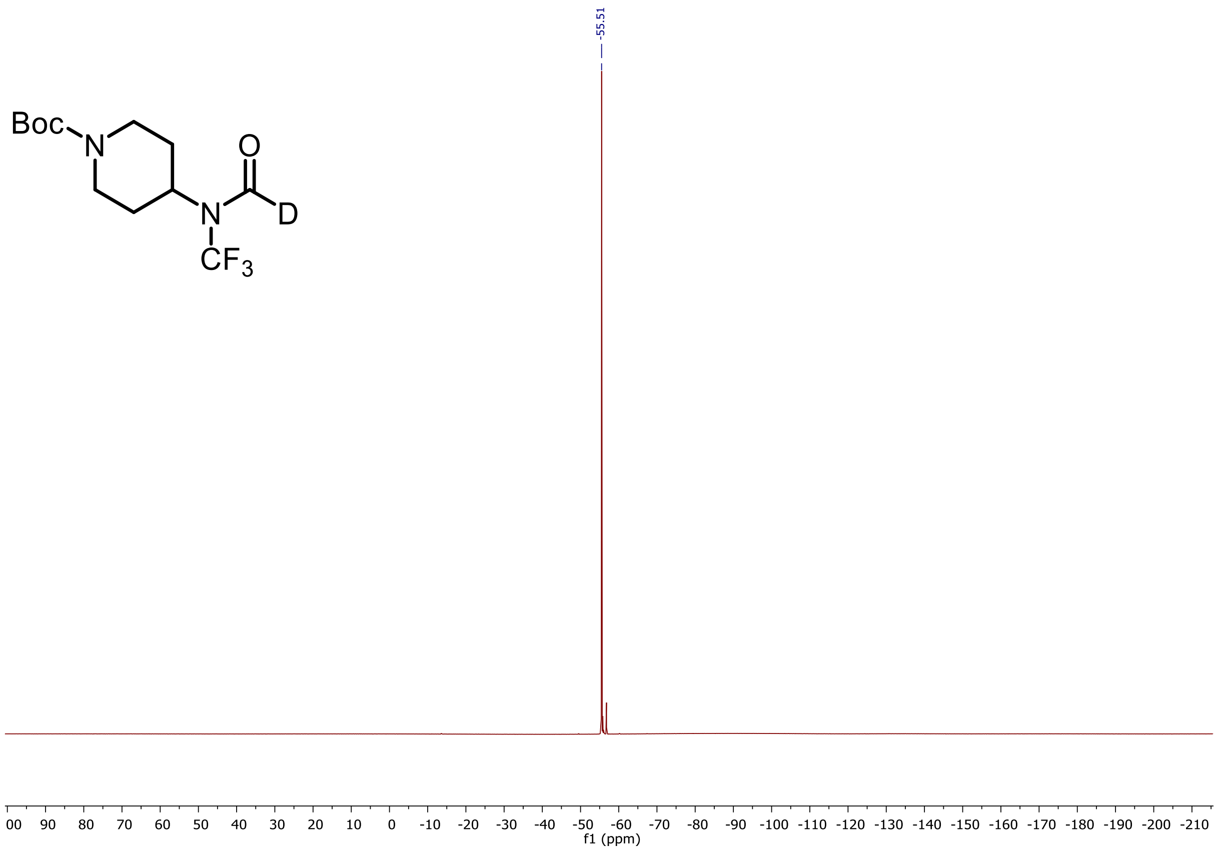
Boc
N
O
D
N
CF3
-55.51
f1 (ppm)

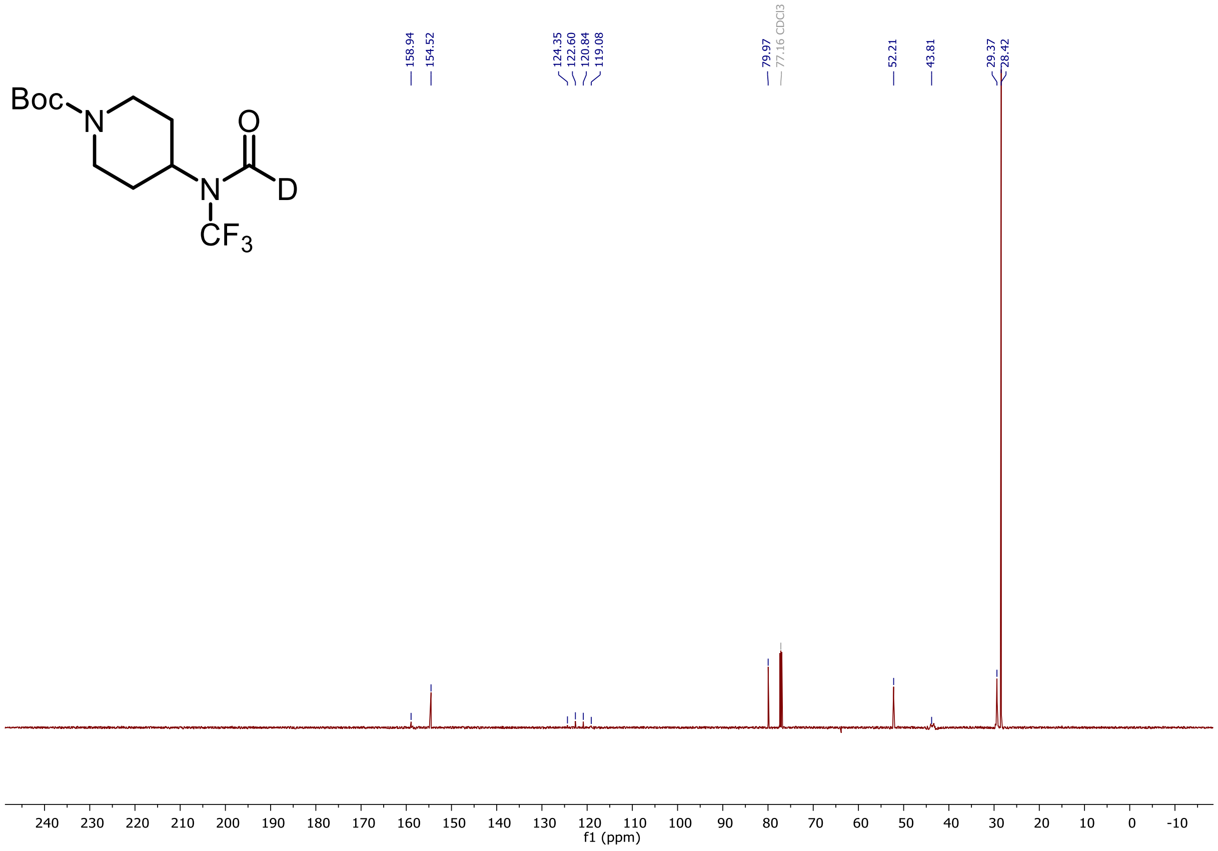
Boc
N
O
D
N
CF3
158.94
154.52
124.35
122.60
120.84
119.08
79.97
77.16 CDCl3
52.21
43.81
29.37
28.42
f1 (ppm)

N-(9-ethyl-9H-carbazol-3-yl)-N-(trifluoromethyl)formamide (S32)

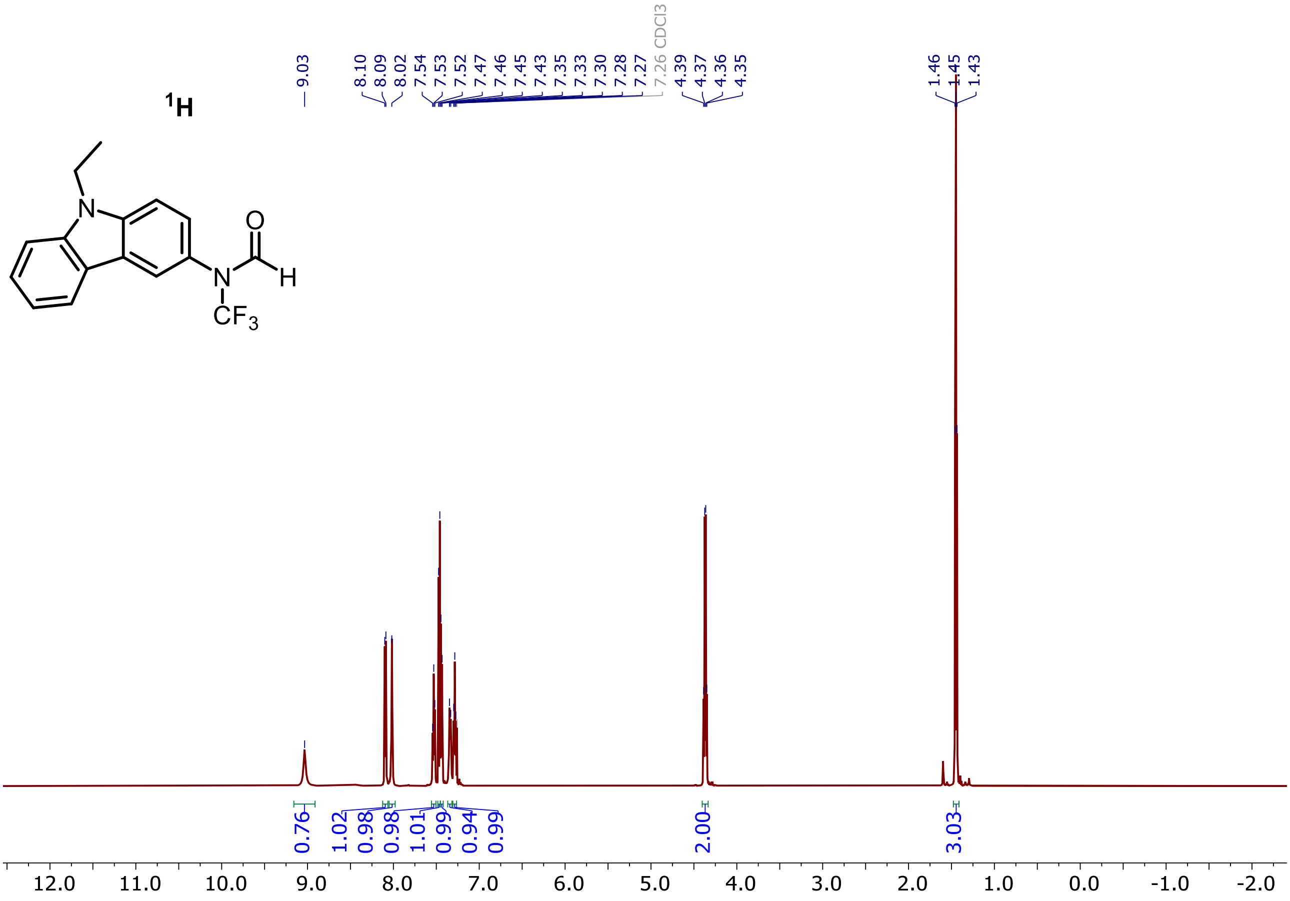

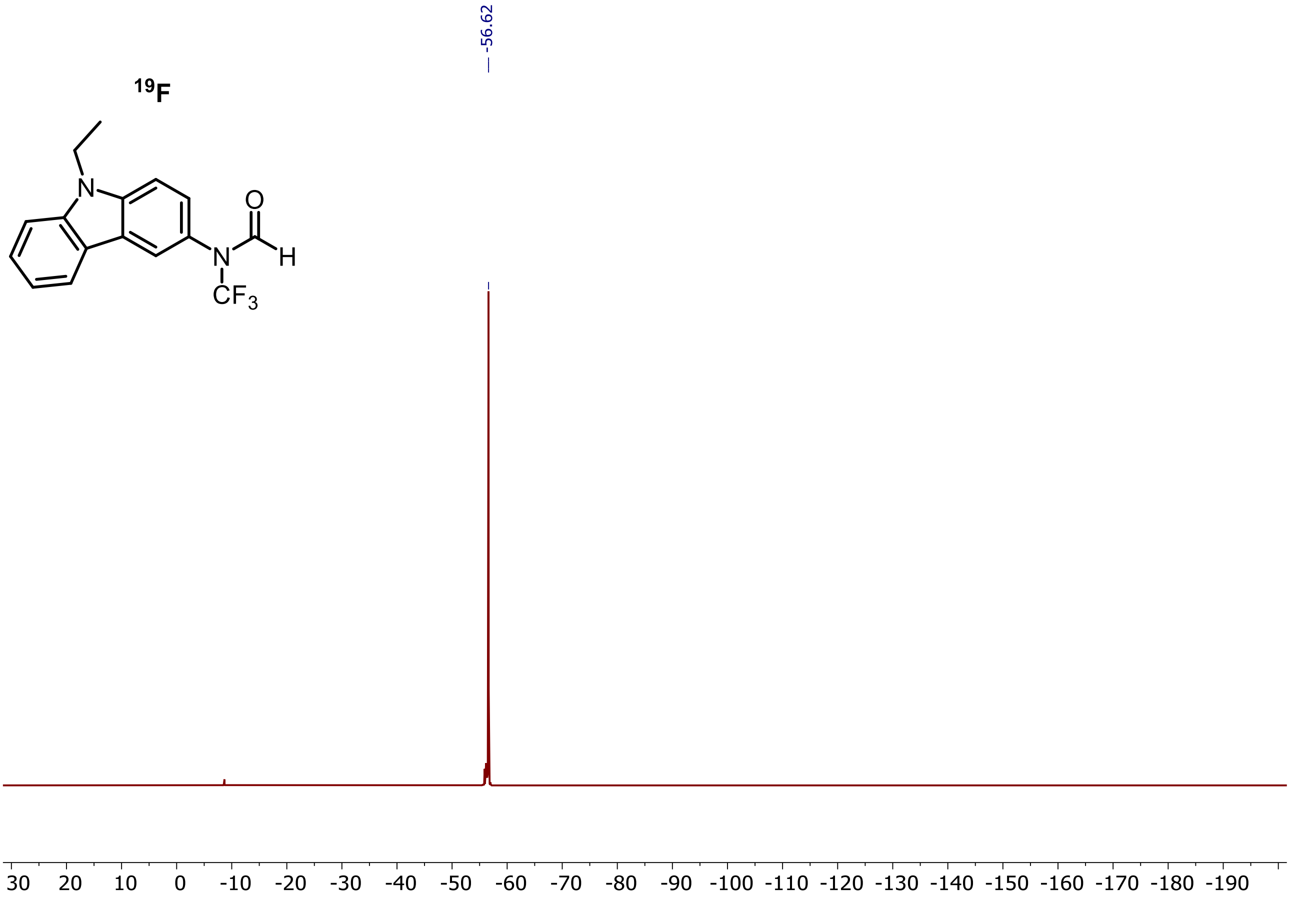

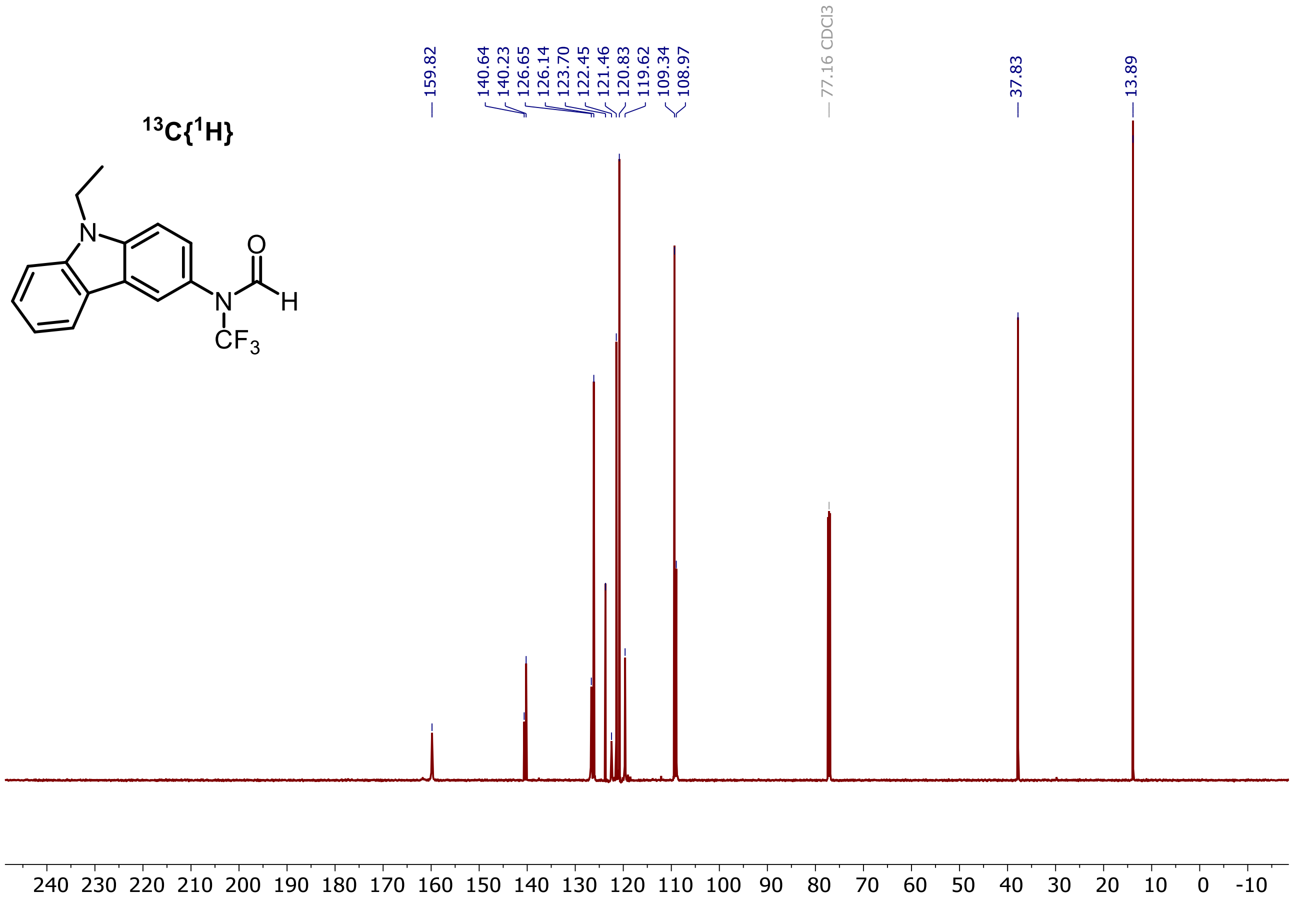

methyl 4-(*N*-(trifluoromethyl)formamido)thiophene-2-carboxylate (S33)

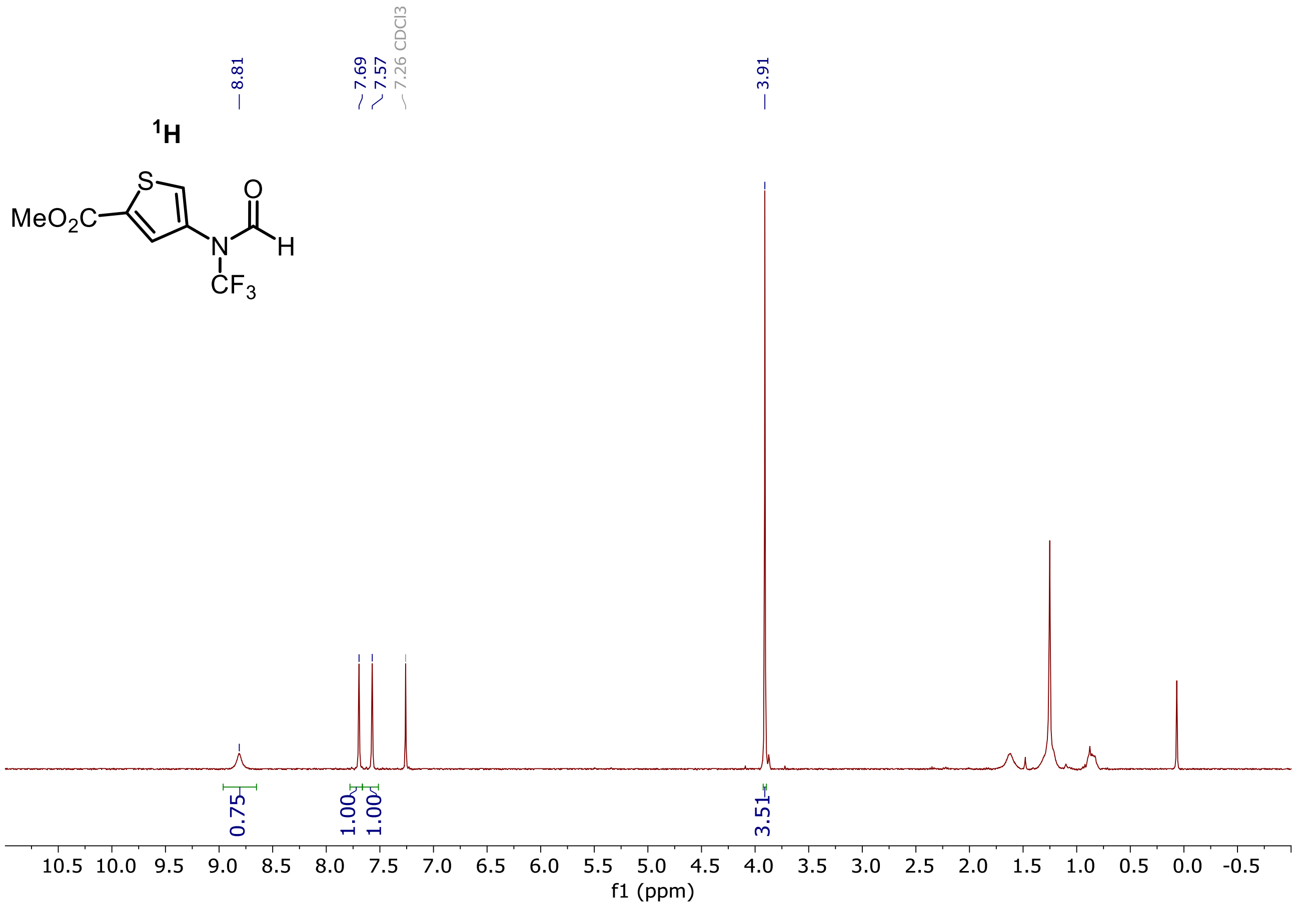

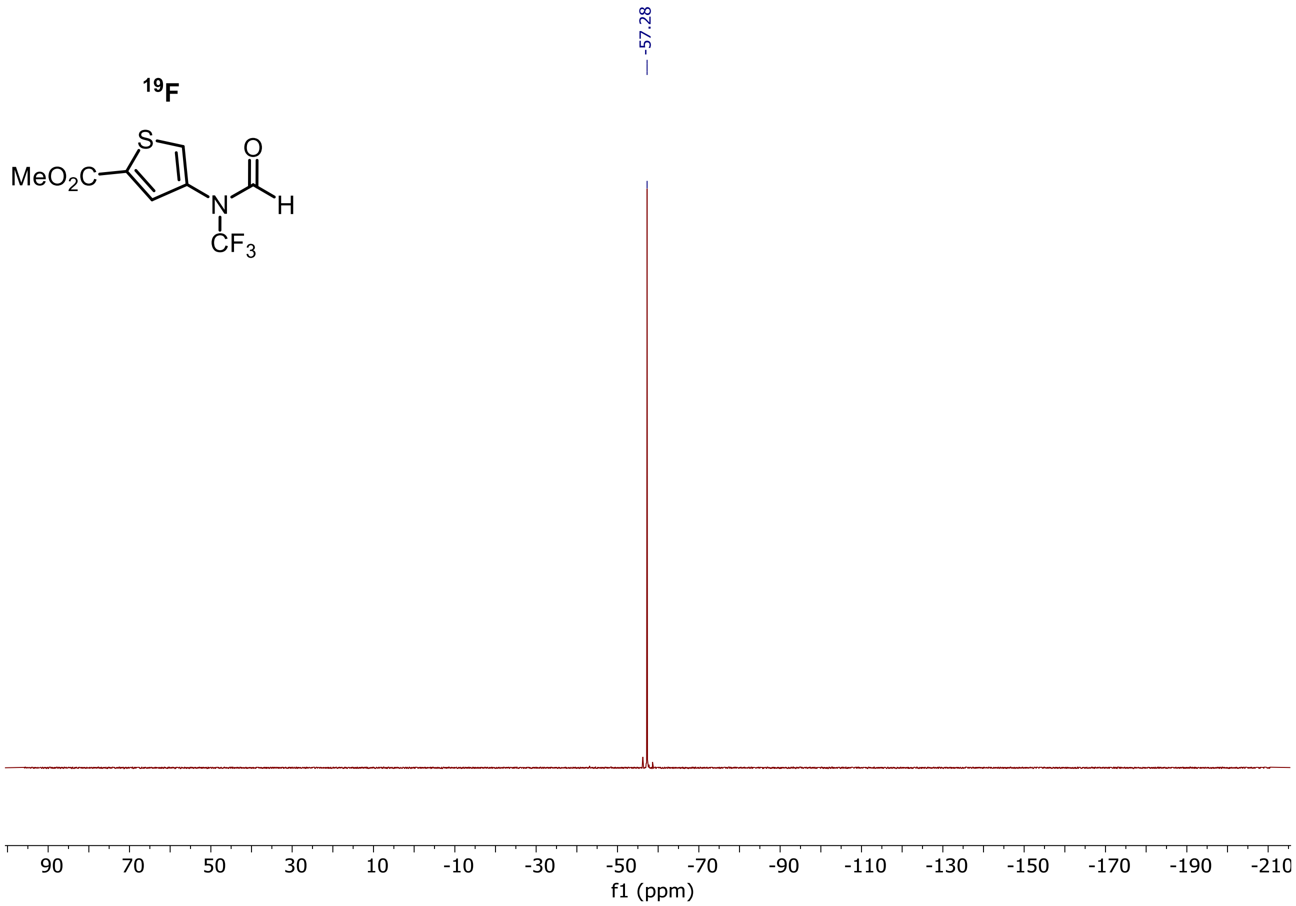
19F
MeO2C
S
O
N
H
CF3
-57.28
90
70
50
30
10
-10
-30
-50
-70
-90
-110
-130
-150
-170
-190
-210
f1 (ppm)

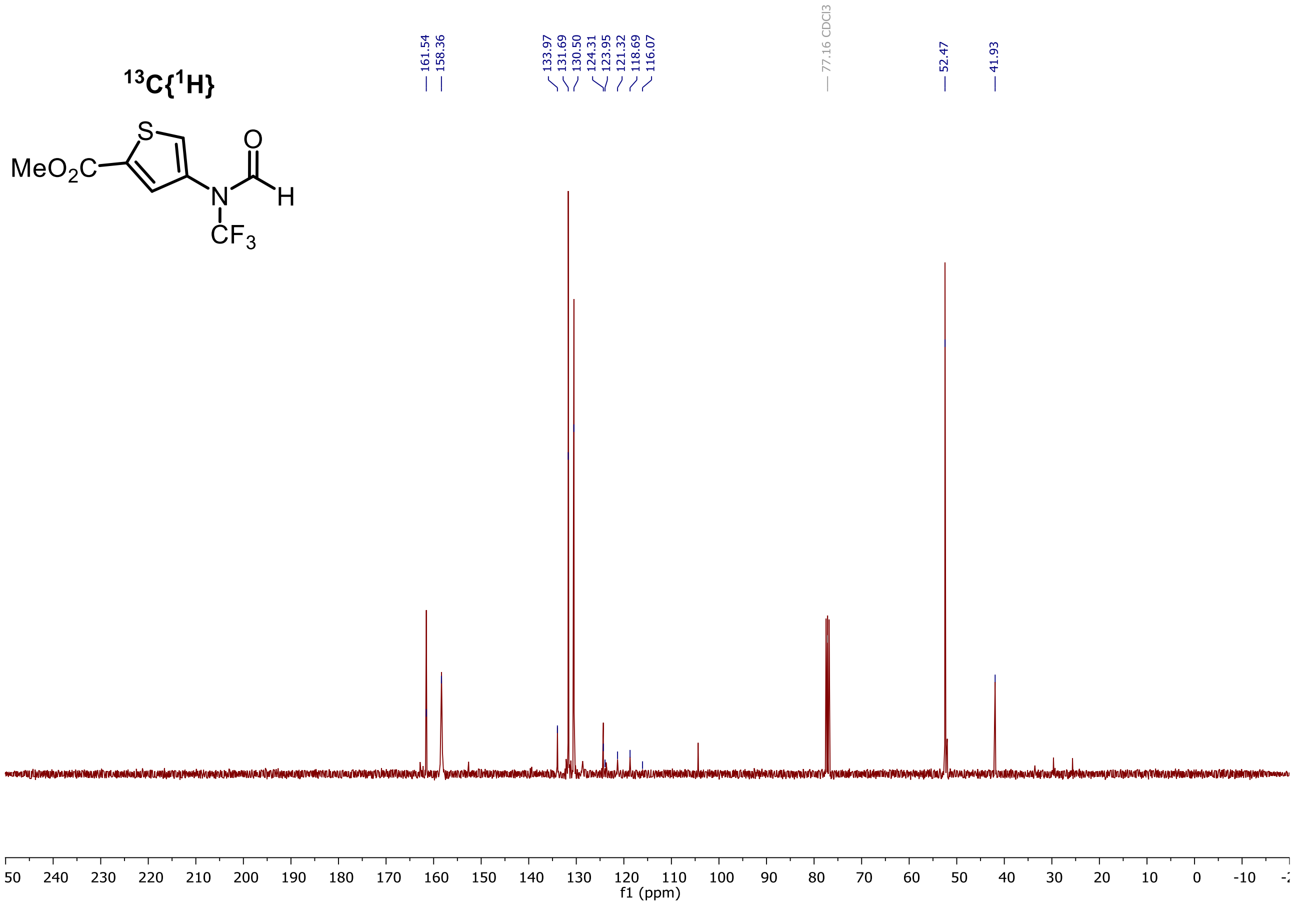
13C{1H}
MeO2C
S
O
N
H
CF3
161.54
158.36
133.97
131.69
130.50
124.31
123.95
121.32
118.69
116.07
77.16 CDCl3
52.47
41.93
240
230
220
210
200
190
180
170
160
150
140
130
120
110
100
90
80
70
60
50
40
30
20
10
0
-10
f1 (ppm)

***N*-(*trans*-2-phenylcyclopropyl)-*N*-(trifluoromethyl)formamide (S34)**

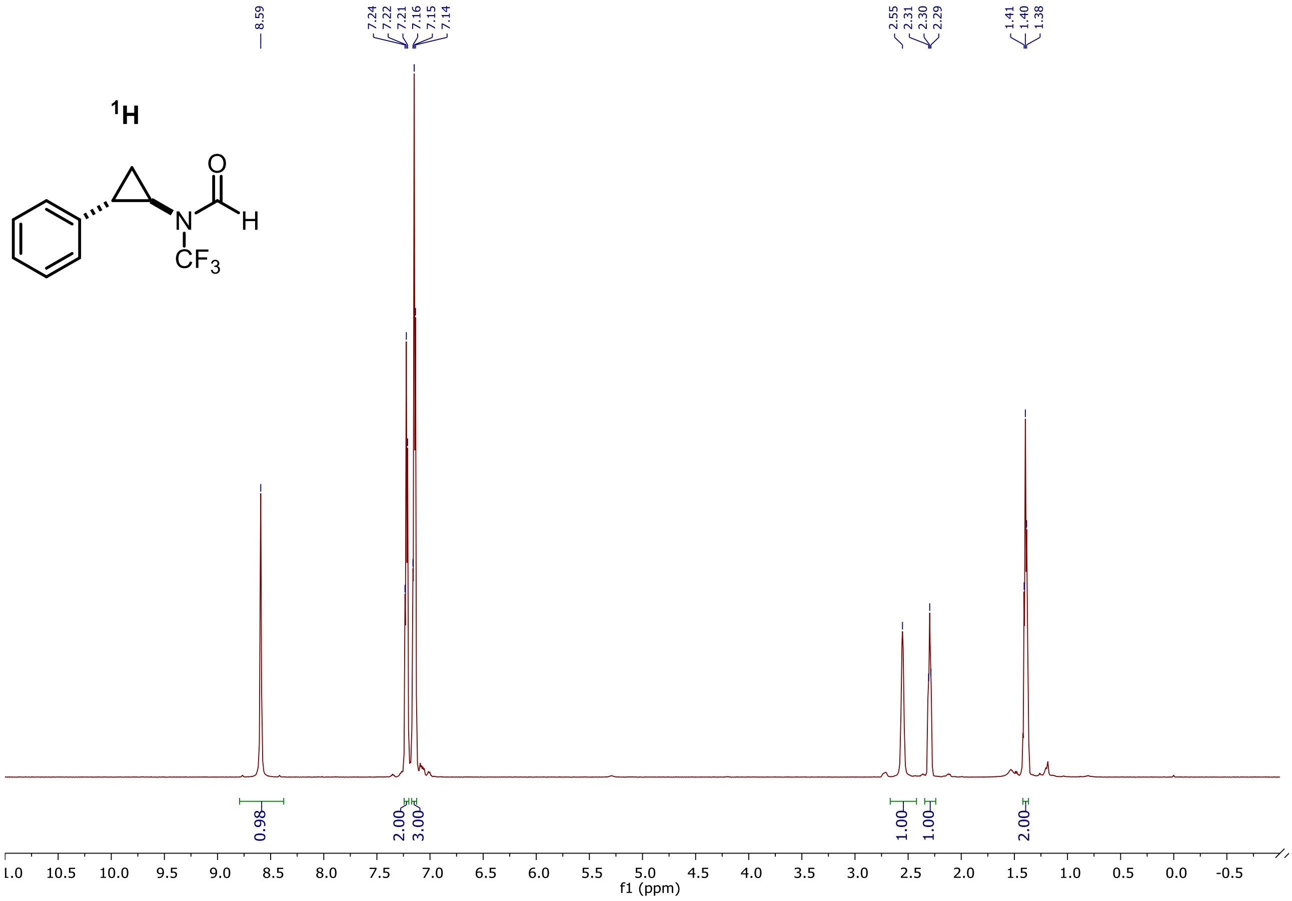

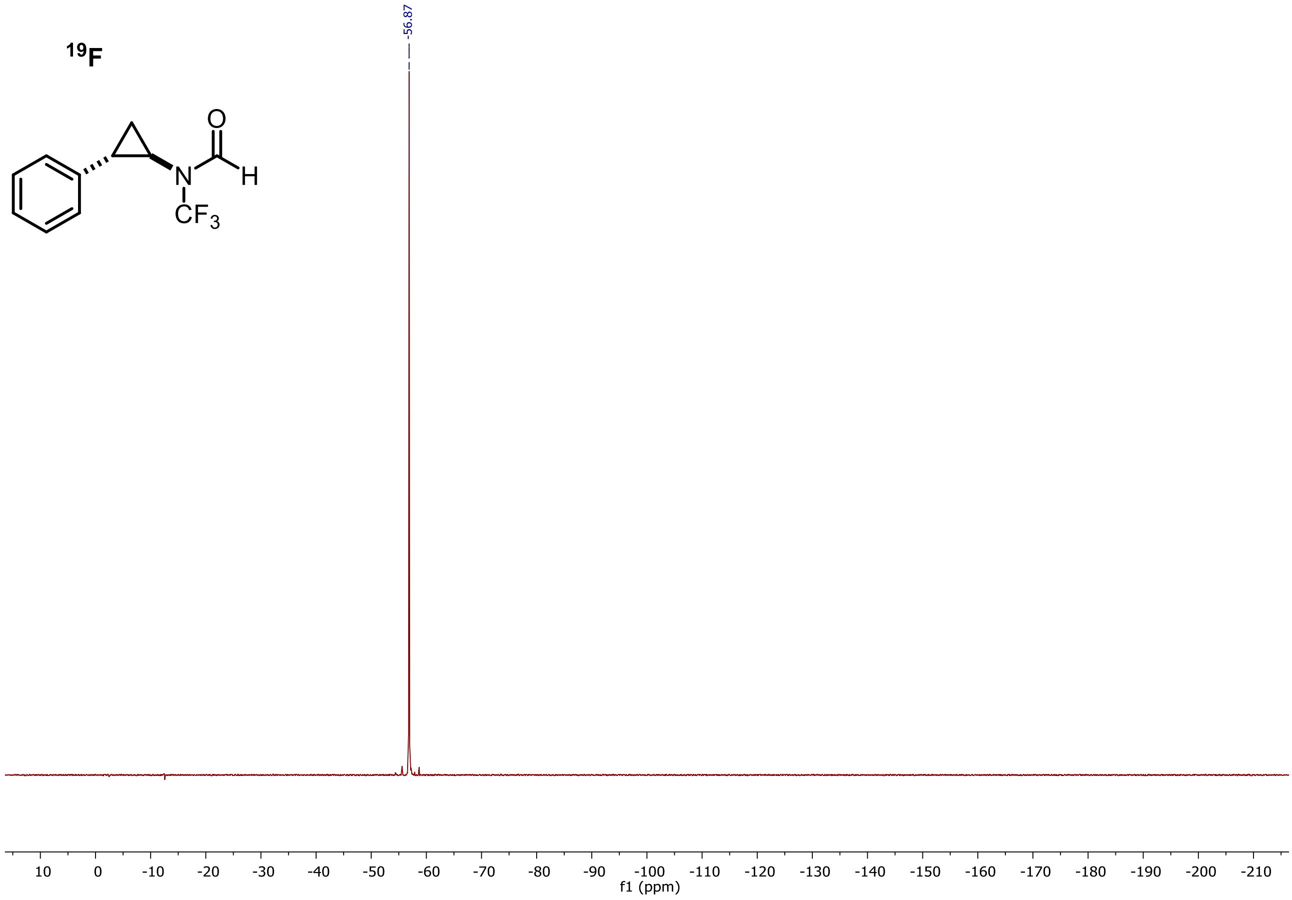

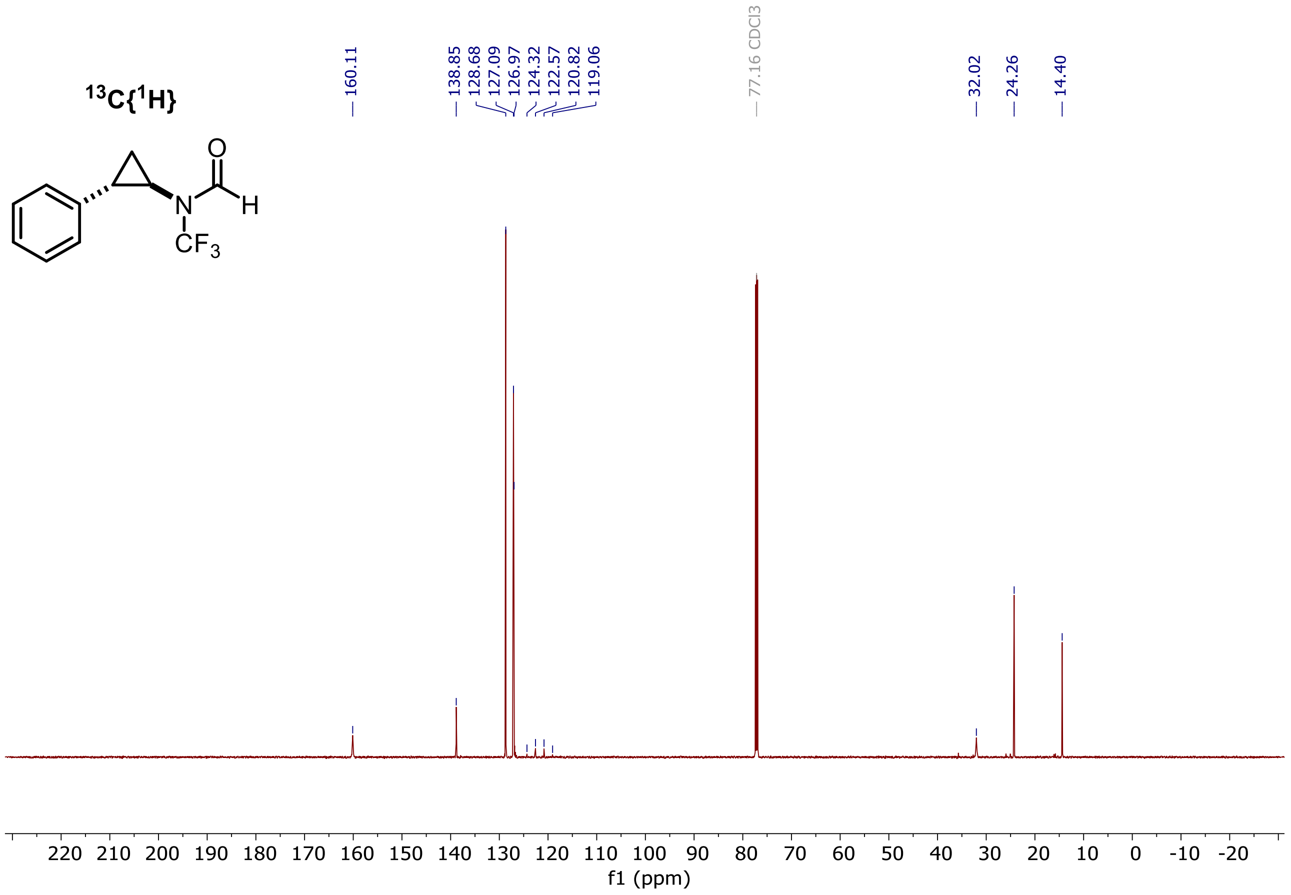

(*R*)-*N*-(1-(3-(2-cyanobenzyl)-1-methyl-2,6-dioxo-1,2,3,6-tetrahydropyrimidin-4-yl)piperidin-3-yl)-*N*-(trifluoromethyl)formamide (*S*35)

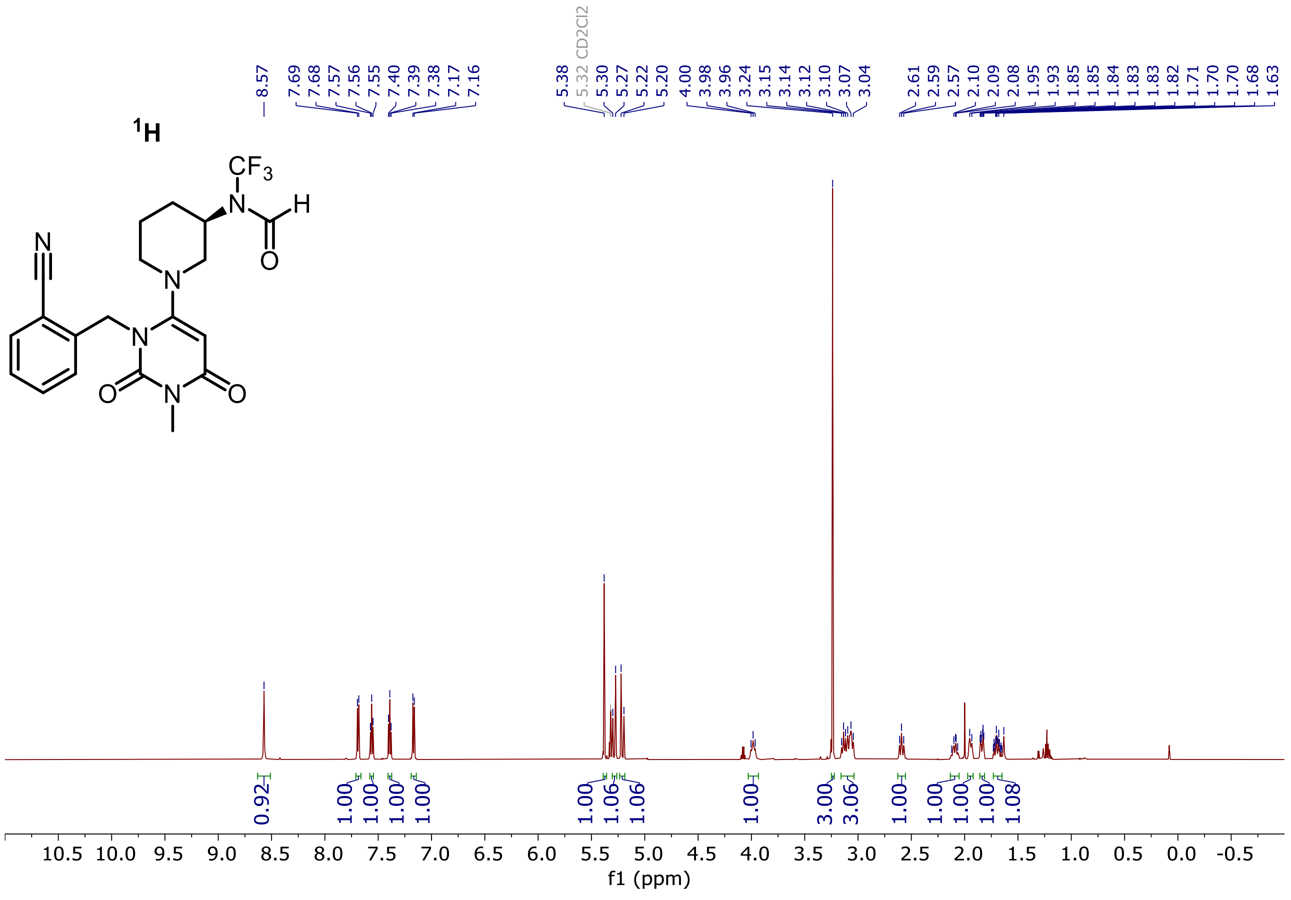

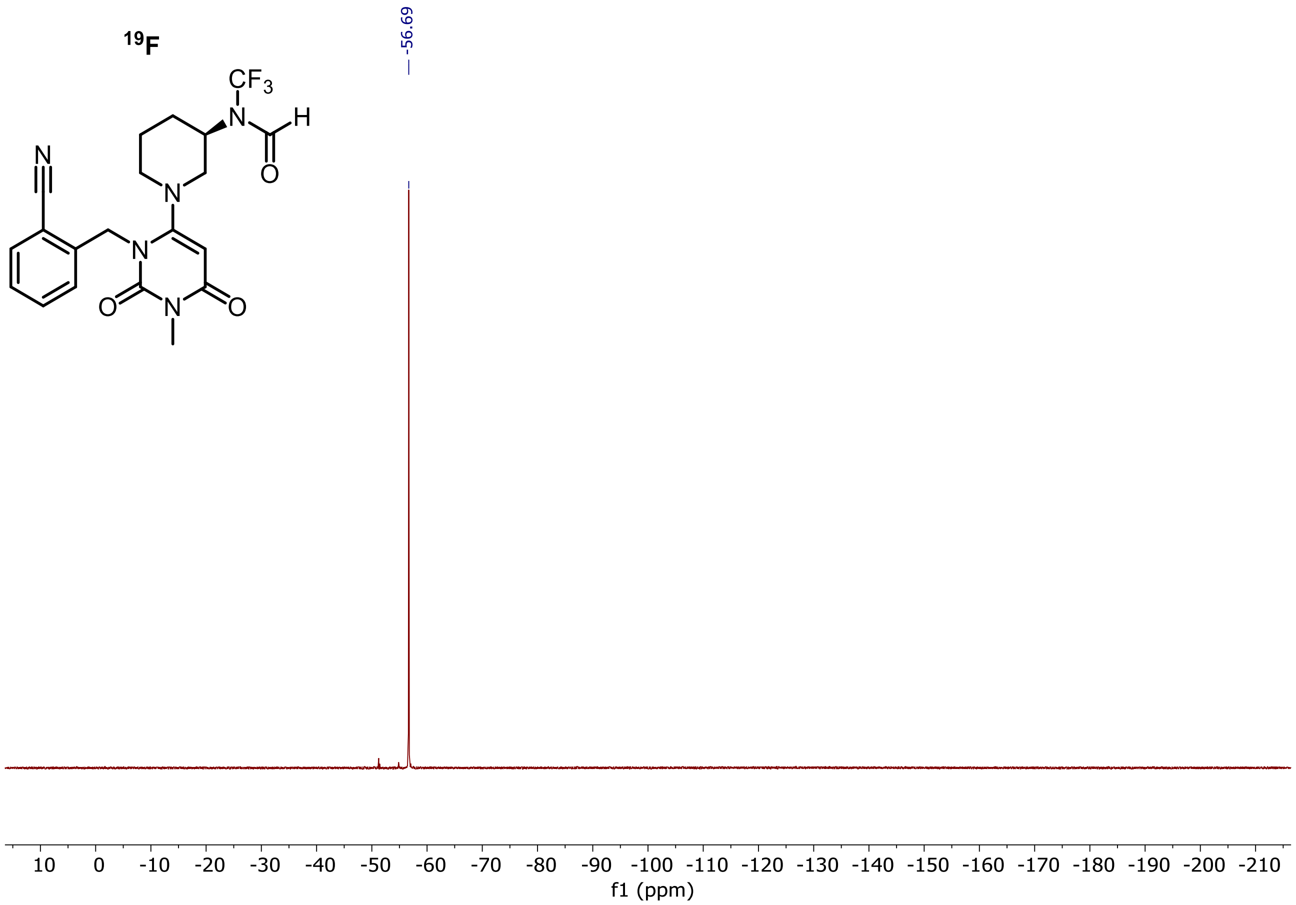
19F
CF3
N
H
O
N
N
N
N
O
O
-56.69
10 0 -10 -20 -30 -40 -50 -60 -70 -80 -90 -100 -110 -120 -130 -140 -150 -160 -170 -180 -190 -200 -210
f1 (ppm)

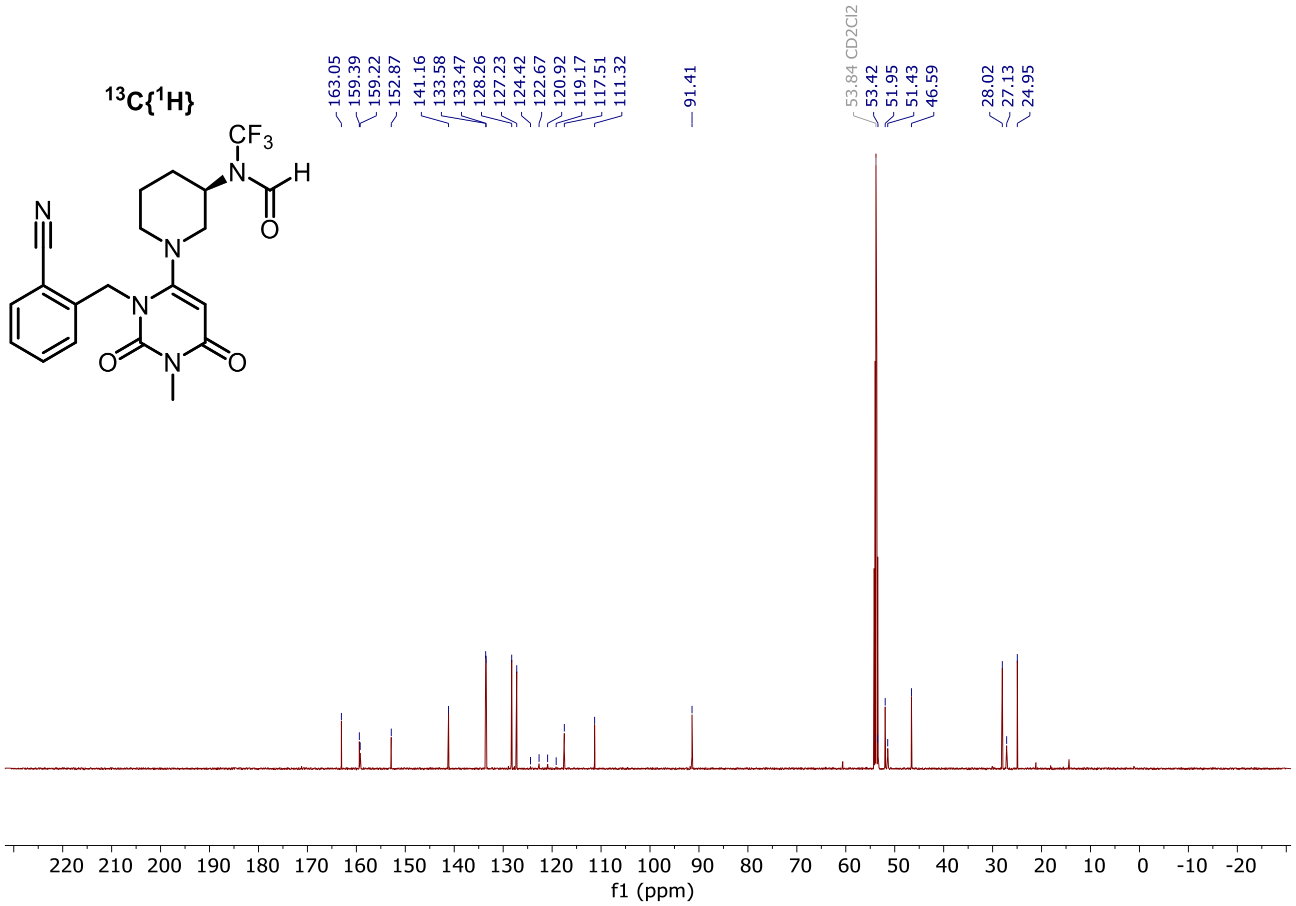
13C{1H}
CF3
N
H
O
N
N
N
N
O
O
163.05
159.39
159.22
152.87
141.16
133.58
133.47
128.26
127.23
124.42
122.67
120.92
119.17
117.51
111.32
91.41
53.84 CD2Cl2
53.42
51.95
51.43
46.59
28.02
27.13
24.95
220 210 200 190 180 170 160 150 140 130 120 110 100 90 80 70 60 50 40 30 20 10 0 -10 -20
f1 (ppm)

12.2.5 *N*-methyl-*N*-(trifluoromethyl)-[1,1'-biphenyl]-4-amine (1-Me)

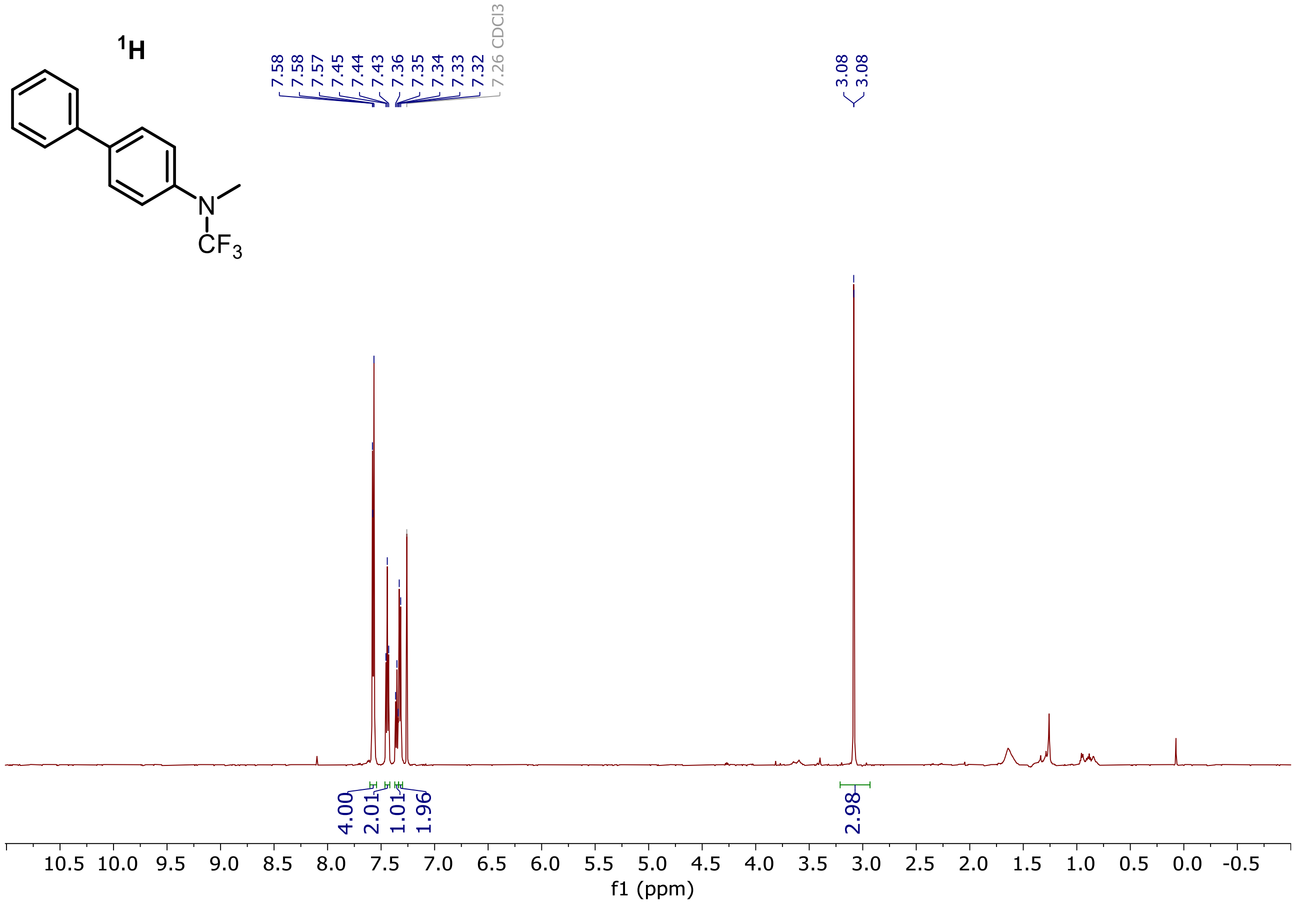

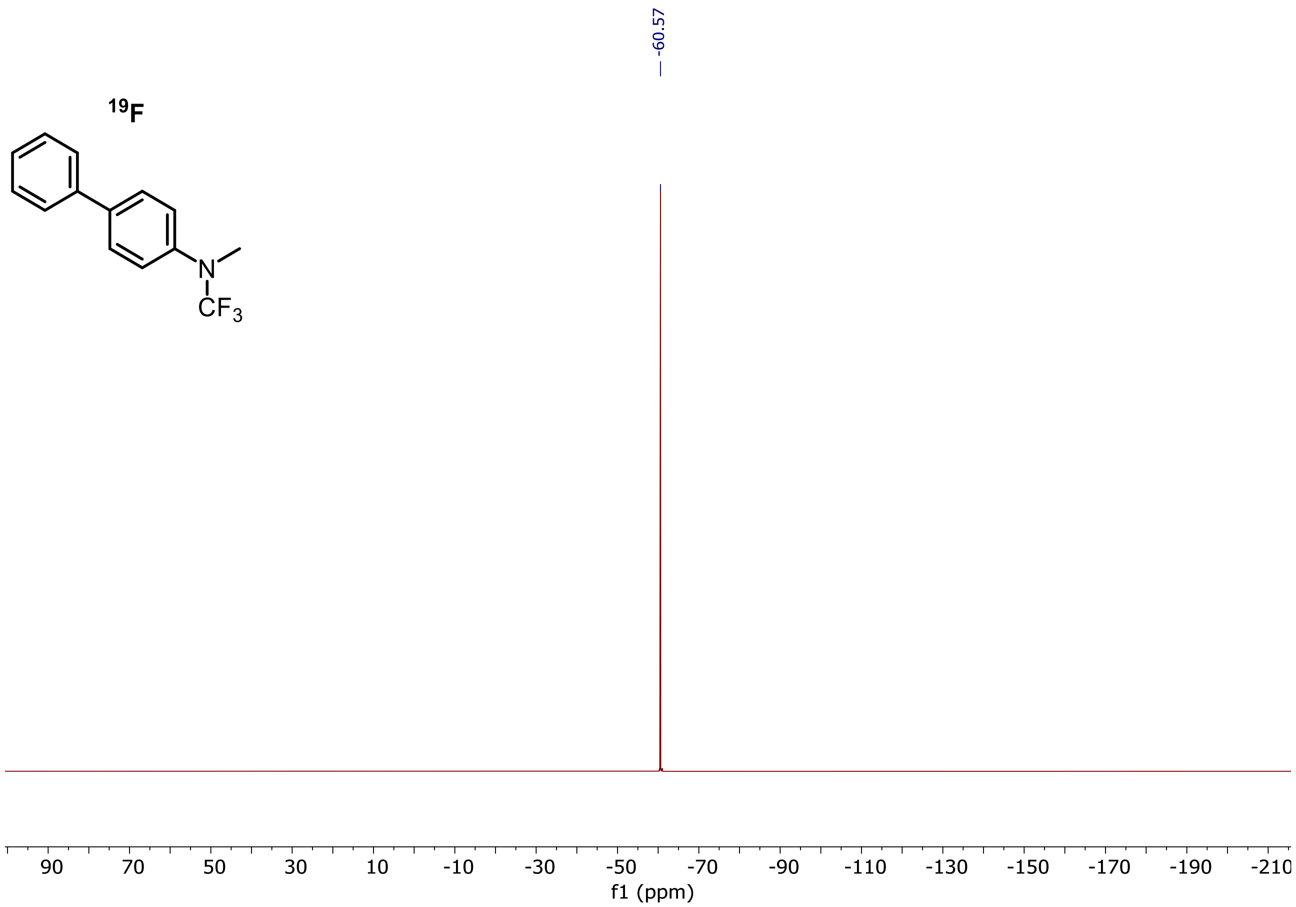

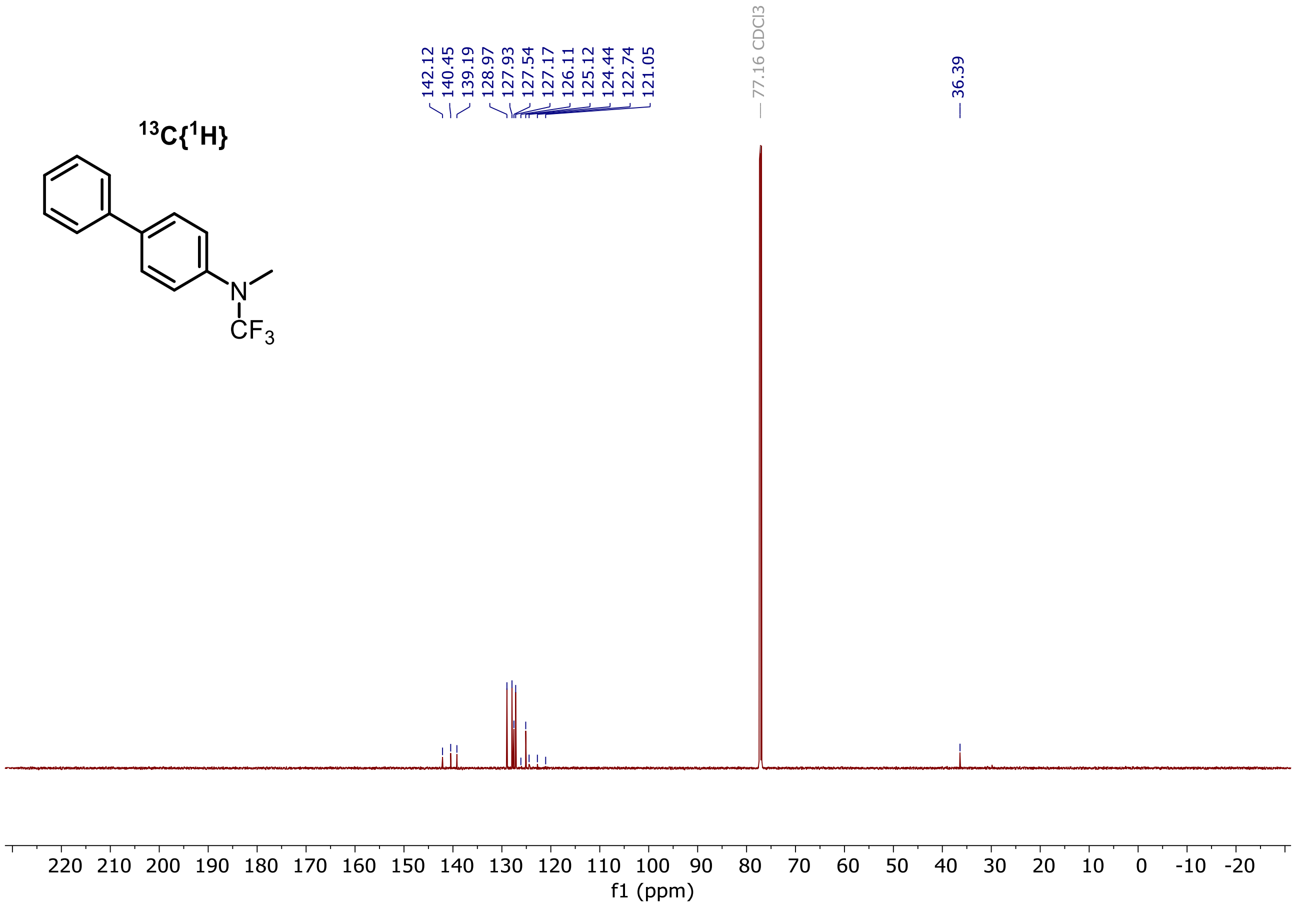

13C{1H}
N
CF3
142.12
140.45
139.19
128.97
127.93
127.54
127.17
126.11
125.12
124.44
122.74
121.05
77.16 CDCl3
36.39
220 210 200 190 180 170 160 150 140 130 120 110 100 90 80 70 60 50 40 30 20 10 0 -10 -20
f1 (ppm)

13. References

[1] T. Scattolin, S. Bouayad-Gervais, F. Schoenebeck, *Nature* **2019**, *573*, 102-107.

[2] F. G. Zivkovic, C. D.-T. Nielsen, F. Schoenebeck, *Angew. Chem. Int. Ed.* **2022**, *61*, e202213829.

[3] S. Bouayad-Gervais, T. Scattolin, F. Schoenebeck, *Angew. Chem. Int. Ed.* **2020**, *59*, 11908-11912.

[4] K. Grollier, A. De Zordo-Banliat, F. Bourdreux, B. Pegot, G. Dagousset, E. Magnier, T. Billard, *Chem. Eur. J.* **2021**, *27*, 6028-6033.

[5] M. H. Abraham, R. J. Abraham, W. E. Acree, Jr., A. E. Aliev, A. J. Leo, W. L. Whaley, *J. Org. Chem.* **2014**, *79*, 11075-11083.

[6] a) *BIOVIA COSMOtherm 21.0,* **2021**, Dassult Systèmes; b) F. Eckert, A. Klamt*, COSMOtherm, Version C3.0, Release 17.01,* **2016**, COSMOlogic GmbH & Co. KG, Leverkusen (Germany), (http://www.cosmologic.de).

[7] *TURBOMOLE V7.5.1 2021, a development of University of Karlsruhe and Forschungszentrum Karlsruhe GmbH (1989-2007),* TURBOMOLE GmbH (since 2007), (https://www.turbomole.org).

[8] a) C. Bannwarth, S. Ehlert, S. Grimme, *J. Chem. Theory Comput.* **2019**, *15*, 1652-1671; b) S. Grimme, *J. Chem. Theory Comput.* **2019**, *15*, 2847-2862; c) S. Grimme, C. Bannwarth, P. Shushkov, *J. Chem. Theory Comput.* **2017**, *13*, 1989-2009.

[9] P. Pracht, F. Bohle, S. Grimme, *Phys. Chem. Chem. Phys.* **2020**, *22*, 7169-7192.

[10] M. J. Frisch*, et al., Gaussian 16, Revision A.03,* **2016**, Gaussian, Inc., Wallingford, CT.
